# The microbiome in cancer

**DOI:** 10.1002/imt2.70070

**Published:** 2025-08-30

**Authors:** Anqi Lin, Minying Xiong, Aimin Jiang, Lihaoyun Huang, Hank Z. H. Wong, Suyin Feng, Chunyan Zhang, Yu Li, Li Chen, Hao Chi, Pengpeng Zhang, Bicheng Ye, Hengguo Zhang, Nan Zhang, Lingxuan Zhu, Weiming Mou, Junyi Shen, Kailai Li, Wentao Xu, Haoxuan Ying, Cangang Zhang, Dongqiang Zeng, Jindong Xie, Xinpei Deng, Qi Wang, Jianying Xu, Wenjie Shi, Chang Qi, Chunrun Qu, Xufeng Huang, András Hajdu, Chaoqun Li, Changmin Peng, Xuanye Cao, Guangsheng Pei, Lin Zhang, Yujia Huo, Jiabao Xu, Antonino Glaviano, Attila Gábor Szöllősi, Sicheng Bian, Zhengrui Li, Hailin Tang, Bufu Tang, Zaoqu Liu, Jian Zhang, Kai Miao, Quan Cheng, Ting Wei, Shuofeng Yuan, Peng Luo

**Affiliations:** ^1^ Donghai County People's Hospital (Affiliated Kangda College of Nanjing Medical University) Lianyungang China; ^2^ Department of Oncology Zhujiang Hospital, Southern Medical University Guangzhou China; ^3^ Department of Urology Changhai hospital, Naval Medical University (Second Military Medical University) Shanghai China; ^4^ Li Ka Shing Faculty of Medicine The University of Hong Kong Hong Kong China; ^5^ Cancer Centre and Institute of Translational Medicine, Faculty of Health Sciences University of Macau Macau China; ^6^ Western (Chongqing) Institut for Digital‐Intelligent Medicine Chongqing China; ^7^ Clinical Medical College Southwest Medical University Luzhou China; ^8^ Department of Lung Cancer Tianjin Lung Cancer Center, National Clinical Research Center for Cancer, Key Laboratory of Cancer Prevention and Therapy, Tianjin's Clinical Research Center for Cancer, Tianjin Medical University Cancer Institute and Hospital Tianjin China; ^9^ Liver Disease Center of Integrated Traditional Chinese and Western Medicine, Department of Radiology Zhongda Hospital, Medical School, Southeast University, Nurturing Center of Jiangsu Province for State Laboratory of AI Imaging & Interventional Radiology (Southeast University) Nanjing China; ^10^ Key Laboratory of Oral Diseases Research of Anhui Province College & Hospital of Stomatology, Anhui Medical University Hefei China; ^11^ College of Life Science and Technology Huazhong University of Science and Technology Hubei China; ^12^ Department of Pathogenic Microbiology and Immunology School of Basic Medical Sciences, Xi'an Jiaotong University Xi'an China; ^13^ Department of Oncology Nanfang Hospital, Southern Medical University Guangzhou China; ^14^ Cancer Center, the Sixth Affiliated Hospital, School of Medicine, South China University of Technology Foshan China; ^15^ State Key Laboratory of Oncology in South China Guangdong Provincial Clinical Research Center for Cancer, Sun Yat‐sen University Cancer Center Guangzhou China; ^16^ Department of Urology, State Key Laboratory of Oncology in Southern China Sun Yat‐sen University Cancer Center, Guangdong Provincial Clinical Research Center for Cancer Guangzhou China; ^17^ Department of Oncology Ruijin Hospital, Shanghai Jiao Tong University School of Medicine Shanghai China; ^18^ Department of Medicine II LMU University Hospital Munich Germany; ^19^ Molecular and Experimental Surgery, Clinic for General‐, Visceral‐, Vascular‐ and Transplantation Surgery, Medical Faculty and University Hospital Magdeburg Otto‐von‐Guericke University Magdeburg Germany; ^20^ Institute of Logic and Computation Vienna University of Technology Vienna Austria; ^21^ Ludwig Institute for Cancer Research, Nuffield Department of Medicine University of Oxford Oxford UK; ^22^ Department of Data Visualization, Faculty of Informatics University of Debrecen Debrecen Hungary; ^23^ Faculty of Dentistry University of Debrecen Debrecen Hungary; ^24^ Hillman Cancer Center UPMC Pittsburgh Pennsylvania USA; ^25^ Department of Biochemistry & Molecular Medicine, GW Cancer Center George Washington University Washington DC USA; ^26^ Department of Molecular and Cellular Biology Baylor College of Medicine Houston Texas USA; ^27^ Center for Precision Health, McWilliams School of Biomedical Informatics The University of Texas Health Science Center at Houston Houston Texas USA; ^28^ The School of Public Health and Preventive Medicine Monash University Melbourne Victoria Australia; ^29^ Division of Biomedical Engineering James Watt School of Engineering, University of Glasgow Glasgow UK; ^30^ Department of Biological Chemical and Pharmaceutical Sciences and Technologies, University of Palermo Palermo Italy; ^31^ Department of Immunology, Faculty of Medicine University of Debrecen Debrecen Hungary; ^32^ Department of Medicine The MetroHealth System, Case Western Reserve University Cleveland Ohio USA; ^33^ Department of Oral and Cranio‐Maxillofacial Surgery Shanghai Ninth People's Hospital, College of Stomatology, Shanghai Jiao Tong University School of Medicine, National Clinical Research Center for Oral Diseases, Shanghai Key Laboratory of Stomatology and Shanghai Research Institute of Stomatology Shanghai China; ^34^ Department of Radiation Oncology Zhongshan Hospital Affiliated to Fudan University Shanghai China; ^35^ Institute of Basic Medical Sciences Chinese Academy of Medical Sciences and Peking Union Medical College Beijing China; ^36^ MoE Frontiers Science Center for Precision Oncology University of Macau Macau China; ^37^ Department of Neurosurgery Xiangya Hospital, Central South University Changsha China; ^38^ National Clinical Research Center for Geriatric Disorders Xiangya Hospital, Central South University Changsha China; ^39^ Department of Infectious Disease and Microbiology The University of Hong Kong‐Shenzhen Hospital Shenzhen China; ^40^ Department of Microbiology State Key Laboratory of Emerging Infectious Diseases, Carol Yu Centre for Infection, School of Clinical Medicine, Li Ka Shing Faculty of Medicine, The University of Hong Kong Hong Kong China

**Keywords:** cancer, microbiome, precision oncology, treatment, tumor microenvironment

## Abstract

The human microbiome is now recognized as a central regulator of cancer biology, intricately shaping tumor development, immune dynamics, and therapeutic response. This comprehensive review delineates the multifaceted roles of bacteria, viruses, and fungi in modulating the tumor microenvironment and systemic immunity across diverse cancer types. We synthesize current evidence on how microbial dysbiosis promotes carcinogenesis via chronic inflammation, metabolic reprogramming, genotoxic stress, immune evasion, and epigenetic remodeling. This review emphasizes organ‐specific microbiome signatures and highlights their potential as non‐invasive biomarkers for early detection, treatment stratification, and prognosis. Furthermore, we explore the impact of intratumoral microbiota on cancer therapies, uncovering how microbial metabolites and host–microbe interactions shape therapeutic efficacy and resistance. Finally, advances in microbiome‐targeted strategies, such as probiotics, fecal microbiota transplantation, and engineered microbes offer new avenues for adjunctive cancer therapy. This review provides a roadmap for future investigation and underscores the transformative promise of microbiome modulation in cancer prevention and treatment.

## Highlights

- Microbiome dysbiosis drives carcinogenesis via multiple mechanisms.
- Microbiome‐targeted strategies offer novel therapeutic avenues.
- Organ‐specific microbiome signatures serve as non‐invasive biomarkers.
- Intratumoral microbiota modulate therapy efficacy and resistance.
- Microbial metabolites reprogram the tumor microenvironment.

## Graphical abstract

This comprehensive review elucidates the transformative role of the human microbiome in cancer biology, revealing innovative mechanistic insights into the intricate tumor–microbe interactions across diverse organ systems. This work highlights the clinical and translational value of novel microbiome‐based biomarkers for early detection, prognosis, and therapeutic response prediction. Furthermore, this review examines breakthrough insights into how the microbiome modulates the tumor microenvironment and influences the efficacy of major cancer treatments, including chemotherapy, radiotherapy, immunotherapy, and targeted therapies. By integrating these findings, this review contributes to an advanced understanding of personalized, effective, and less‐toxic cancer management strategies.

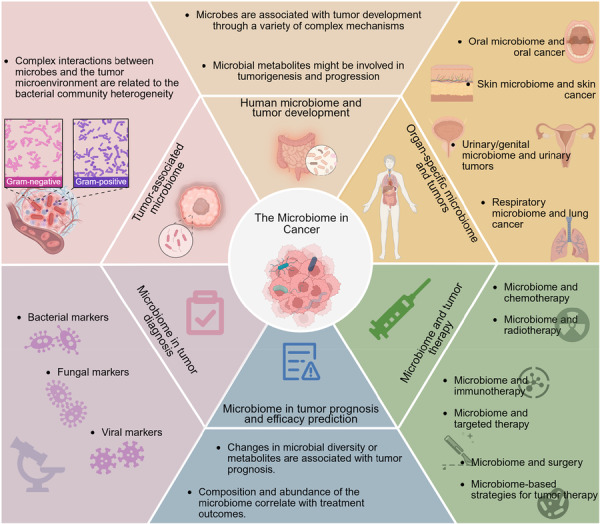

## INTRODUCTION

Microorganisms comprise diverse microscopic entities, including bacteria, fungi, viruses, archaea, and protists. These microorganisms, including 10^13^–10^14^ bacteria colonizing human skin, intestines, and respiratory tract, establish complex symbiotic relationships with the host and are collectively referred to as the body's “second genome” [1, 2, 3, 4]. Symbiotic microbes maintain host health through complex metabolic reciprocity [5]. In contrast, pathogenic microorganisms pose a threat to the host's health. Notably, microorganisms can have dual (beneficial and pathogenic) roles. When host immune function is compromised, commensal microbes may convert to conditionally opportunistic pathogens, potentially disrupting host health [6]. Clinical studies have confirmed that the composition of the gut microbiome can be modulated through fecal microbiota transplantation (FMT) and the administration of probiotic preparations, influencing immune cell infiltration, immune factor release, and inflammatory responses in the intestine and surrounding tumor tissues, thereby improving the immune status of the tumor microenvironment (TME) [7, 8, 9]. Consequently, the microbiome serves not only as a biomarker for disease diagnosis but also as a promising target for therapeutic intervention [10, 11, 12].

While research on gut microbes dates back to the 18th century, comprehensive investigations into the gut microbes expanded exponentially in the 21st century. A study in 1972 established that gut microbes play a critical role in drug transformation [13]. This seminal work laid the groundwork for understanding the microbiome as a key modulator of pharmacology, a concept fundamental to modern microbiome–drug interaction studies. Since 2006, several pivotal studies have begun to emphasize the significant impact of diet on gut microbial composition and host metabolism [14, 15, 16, 17, 18, 19]. In 2007, the National Institutes of Health (NIH) launched the Human Microbiome Project (HMP), the first large‐scale collaborative program to study the human gut microbiome [20]. The Metagenomics of the Human Intestinal Tract (MetaHIT) project, initiated by the European Union in 2008, represents another landmark effort in the comprehensive and systematic study of gut microbiota. This initiative systematically characterized the genomic architecture of intestinal microbial communities and established foundational frameworks for investigating microbiome–disease associations, significantly advancing the development of precision medicine approaches [21]. Subsequently, Yatsunenko et al. conducted a large‐scale comparative analysis demonstrating that human populations across distinct geographic regions have significantly different compositions and functional characteristics of gut microbes [22]. The American Gut Project (AGP), launched in 2012, was further expanded into a global microbiome research program to explore the composition and diversity of microbes in geographically diverse populations [23]. Notably, the study of gut microbes–host interactions has gradually extended to the field of disease treatment. A groundbreaking study in 2018 demonstrated that gut microbial signatures can predict immunotherapeutic response in patients with melanoma, non‐small cell lung cancer, and renal cell carcinoma [24]. This landmark discovery provided the first robust clinical evidence establishing a causal relationship between the gut microbiome composition and the efficacy of cutting‐edge cancer immunotherapies, thereby creating a paradigm‐shifting theoretical framework for understanding the microbiome's pivotal role in developing therapeutic intervention. These findings establish a crucial theoretical framework for understanding the pivotal role of tumor‐associated microbes in cancer initiation, progression, and therapeutic intervention.

Investigations into intratumoral microbes began in the 19th century, but significant advances remained limited until recent decades [25]. In 2020, through a large‐scale multi‐omic analysis of diverse primary human malignancies, researchers established the presence of highly specific microbiome profiles and functional characteristics across different tumor types [26]. This study provides comprehensive evidence for the existence of distinct tumor‐type‐specific intratumoral microbiomes. In recent years, the prevalence of microbes within tumors has been further confirmed by increasing amounts of reliable evidence obtained through various advanced technological approaches [27, 28]. Studies have demonstrated that the species composition and abundance distribution of intratumoral microbes in different cancer types show marked heterogeneity [29, 30, 31]. As investigations into tumor‐associated microbiota intensify, the precise origins and molecular mechanisms governing microbial colonization remain fundamental unresolved questions in the field. Recent investigations have characterized three potential routes of microbial access to tumor tissues: disruption of epithelial barrier integrity, migration from adjacent microenvironmental niches, and systemic dissemination [29, 32]. Substantial evidence indicates that intratumoral microbes are involved in the regulation of tumorigenesis, progression, and therapeutic response through multiple molecular mechanisms, including DNA damage induction, oncogenic signaling pathway activation, and alteration of epigenetic regulation [33]. Additionally, intratumoral microbes significantly affect the clinical efficacy of immunotherapy and chemotherapy by remodeling the tumor immune microenvironment (TIME). As intratumoral microbiome research advances, microbiome‐targeted tumor precision diagnosis and individualized treatment strategies have emerged as an international research frontier. This recognition underscores the immense translational potential of the intratumoral microbiome and its associated metabolites, positioning them as critical targets for the next generation of diagnostics and personalized therapeutic interventions, particularly within the TME and immunomodulation approaches. However, methodological challenges still exist, including sample acquisition, microbial detection, and functional validation [34, 35, 36, 37]. The study of intratumoral microbiota holds profound clinical translational potential and is positioned to constitute a significant breakthrough and promising research direction in anti‐tumor therapy.

The microbiome plays a critical role in multiple aspects of tumor growth, proliferation, diagnosis, treatment, and prognosis. Microorganisms and malignant cells engage in complex bidirectional interactions [38]. Microorganisms can promote tumorigenesis and development through direct genotoxic mechanisms or indirect immunomodulatory effects [39, 40]. Certain beneficial bacteria are also associated with anti‐cancer effects. Probiotics maintain intestinal barrier homeostasis, regulate anti‐inflammatory cytokine levels with anti‐cancer properties, and enhance immune surveillance through activation of phagocytic cells. Germ‐free murine models demonstrate compromised immunosurveillance and increased susceptibility to chemical carcinogenesis [41]. *Lactobacillus* and *Bifidobacterium* have also demonstrated potential to bind and degrade carcinogenic compounds [42]. Importantly, the microbial community serves as a diagnostic and prognostic biomarker [43, 44, 45]. Furthermore, the microbiome influences therapeutic effects by metabolizing drugs and dynamically modulating the immune microenvironment [46]. In immunotherapy, dysbiosis of the intestinal microbes (such as reduction of *Bifidobacteria*) exacerbates anti‐CTLA‐4 therapy‐induced colitis, whereas probiotic supplementation both mitigates toxicity and enhances therapeutic efficacy [47]. As research exploring microbiome–tumor interactions intensifies, microbe‐targeted therapies are gradually being recognized for their anti‐tumor effects. For example, probiotics help maintain intestinal homeostasis and inhibit proliferation of malignant cells [48]. FMT therapy contributes to immunomodulation of the TME, resulting in a tumor‐preventive effect at an early stage [8, 49]. The microbiome, as an emerging biomarker and potential therapeutic target, has broad applications in tumor diagnosis and treatment [50, 51, 52, 53]. Consequently, microbial dysbiosis and intervention emerge as critical factors throughout carcinogenesis and disease progression. Targeted microbial therapy is emerging as a promising approach for integrative oncological strategies [54, 55, 56, 57, 58, 59].

This review comprehensively synthesizes recent advances in understanding the complex interactions between the microbiome and cancer biology. Initially, we examine the intrinsic relationships between the intestinal microbiota (encompassing bacteria, viruses, fungi, and their metabolites) and the intestinal barrier in carcinogenesis, characterize microbial signatures within tumor tissues, and assess their organ‐specific manifestations and diagnostic implications across diverse anatomical sites including the oral cavity, integumentary system, urogenital tract, and respiratory tract. We subsequently focus on the diagnostic and prognostic implications of microbiome profiles, emphasizing the potential applications of site‐specific and kingdom‐specific microbial signatures as clinically actionable biomarkers for early cancer detection, therapeutic response prediction, and outcome assessment. Building upon the above studies, we further elucidate the synergistic effects of microbiome regulation in chemotherapy, radiotherapy, immunotherapy, targeted therapy, and surgery. Additionally, we comprehensively summarize various innovative microbiome‐based therapeutic strategies, including probiotic/prebiotic interventions, FMT, engineered bacteria therapy, and targeted antimicrobial approaches. Studies have shown that the microbiome not only influences tumor initiation and progression but also offers promising new targets for tumor prevention and treatment. Looking forward, continued technological innovation in microbiome research methodologies, elucidation of molecular mechanisms underlying microbiome–host interactions, development of precision microbiome‐targeted therapeutic strategies, and advancement of robust clinical translational studies will collectively deliver more personalized, effective, and safer therapeutic interventions for cancer patients.

## GUT MICROBIOME AND TUMOR DEVELOPMENT

### Association of bacteria with tumorigenesis

Gut microbes contribute to tumor development through a variety of complex mechanisms, predominantly promoting neoplastic progression via induction of chronic inflammatory responses, disruption of epithelial barrier integrity, and comprehensive remodeling of the TIME [60, 61, 62, 63]. The relationship between microbiota composition and carcinogenesis demonstrates pronounced species‐specificity, with pro‐tumorigenic effects predominantly characterized by depletion of beneficial commensal taxa, enrichment of pathogenic bacteria, and dysregulated expansion of opportunistic bacteria [64, 65, 66]. However, some investigations have reported heterogeneous findings [67, 68]. Table 1 summarizes the associations between different types of bacteria (probiotics and pathogenic bacteria) and various tumors, delineating their proposed mechanistic contributions to carcinogenesis or tumor suppression, thereby providing a comprehensive framework for understanding the diverse roles of the microbiome in cancer pathogenesis.

**Table 1:** Associations and potential mechanisms between different bacterial types and various tumors.

Bacterial type	Representative strains	Associated cancer types	Mechanism	Specificity	References
Probiotics	*Lactobacillus*	GC CRC Liver cancer BCa	• Regulate the TME pH and bile acid metabolism; • Degrade carcinogens.	Particular case: elevated *Lactobacillus* abundance in intrahepatic cholangiocarcinoma	[58, 67, 69, 70, 71, 72, 73]
	*Bifidobacterium*	GC CRC Liver cancer	• Competitively inhibit pathogen colonization; • Regulate the TME pH and bile acid metabolism; • Maintain intestinal barrier integrity.	Enriched in PC	[58, 67, 73]
Pathogens	*Helicobacter pylori*	GC CRC PC HCC	• Induce DNA damage; • Disrupt DNA repair; • Activate STAT3 signaling; • Decrease Treg cells; • Increase CD3 cells.	Reduces Barrett's esophagus, EAC and IBD risk	[64, 68, 74, 75, 76, 77, 78]
	*Fusobacterium nucleatum*	CRC PC Oral cancer Gliomas	• Activate pro‐cancer pathways; • Induce DNA methylation; • Suppress immune cell anti‐tumor function.	Promotes tumorigenesis in PC	[40, 79, 80, 81]
	*Pseudomonas*	PC	• *Pseudoxanthomonas*: recruits CD8^+^ T cells (anti‐tumor).	Improve the OS of PC	[81]
	*Enterotoxigenic Bacteroides fragilis*	CRC	• Highly inducible to inflammatory stimuli.		[82]
Microbial synergy	*Helicobacter pylori + phage*	CRC	• Altering the enterovirus composition.	Synergism promotes CRC progression	[75]
	*Ruminococcaceae + Bacteroides*	HCC	• Induce hepatic steatosis; • Accumulate toxic metabolites (such as ammonia).	Alters liver metabolic microenvironment	[66]

Abbreviations: BCa, bladder cancer; CRC, colorectal cancer; EAC, esophageal adenocarcinoma; GC, gastric cancer; HCC, hepatocellular carcinoma; IBD, inflammatory bowel disease; OS, overall survival; PC, pancreatic cancer; pH, potential of hydrogen; STAT3, signal transducer and activator of transcription 3; TME, tumor microenvironment.

#### Probiotics and tumorigenesis

Intestinal probiotics, beneficial bacteria colonizing the human gastrointestinal tract, play a crucial role in promoting nutrient absorption and maintaining gastrointestinal health by orchestrating gut microbial balance and calibrating intestinal mucosa and systemic immune function through multiple mechanisms [69, 70, 71]. Common intestinal probiotics primarily include *Bifidobacterium*, *Lactobacillus*, and *Bacillus* [72, 73, 83, 84]. *Lactobacillus* and *Bifidobacterium* demonstrate significant anti‐tumor activity, inhibiting not only digestive malignancies, such as esophageal, gastric, colon, and liver cancer, but also bladder cancer (BCa), through mechanisms including regulation of the pH of the TME, modulation of bile acid metabolism, and degradation of potential carcinogens and their metabolites [66, 85, 86, 87, 88]. Additionally, tumor metastasis in patients with gastric cardia adenocarcinoma considerably correlates with decreased *Lactobacillus* counts in the stomach [89]. Patients with hepatocellular carcinoma (HCC) exhibit significantly altered gut microbial composition, with hepatitis B virus (HBV)‐associated HCC displaying significant enrichment of *Bacteroides* and *Lachnospiraceae incertae sedis*, while non‐HBV‐associated HCC shows decreased abundance of *Faecalibacterium*, *Ruminococcus*, and *Ruminoclostridium* [90, 91]. Notably, patients with intrahepatic cholangiocarcinoma had a significantly higher abundance of *Lactobacillus* in fecal samples compared to healthy controls [67]. Comprehensive analyses have established significantly reduced duodenal microbial diversity in patients with pancreatic cancer (PC), with enrichment of *Bifidobacterium spp*. and *Rothia* bacteria [67]. Furthermore, multiple microorganisms, including seven *Bacteroidales* species, significantly enhanced anti‐tumor immune responses mediated by CD8^+^ T cells in mice through synergistic effects [92]. Although *Bifidobacterium* and *Lactobacillus* are widely recognized as probiotics within the intestinal ecosystems, their functions in a specific TME may diverge substantially from their canonical probiotic activities. This effect is influenced by multiple factors, including tumor histology, metabolic state, and the dominant bacterial strain. Therefore, “probiotics” cannot be simplistically equated with “anti‐tumor,” necessitating that future microbiome‐targeted therapies carefully consider strain‐specific functions within particular TME.

#### Pathogenic bacteria and tumorigenesis

Pathogenic bacteria are disease‐causing microorganisms. These bacteria can damage host cells either directly or indirectly by releasing toxins or inducing immune responses. Intestinal pathogenic bacteria can induce acute or chronic inflammatory responses in the host, several of which directly contribute to tumor development and progression [93, 94, 95, 96, 97, 98]. Common intestinal pathogenic bacteria include *Salmonella*, *Shigella*, *enterohemorrhagic E.coli*, *Staphylococcus aureus* (*S. aureus*), and *Clostridium difficile* [99, 100, 101, 102, 103, 104]. Gastrointestinal tumor development closely relates to the dynamics of various intestinal microbiota, including *enterotoxigenic Bacteroides fragilis*, *Porphyromonas*, *Flavonifractor plauti* [105], *Campylobacter spp*. [106, 107], and *Fusobacterium nucleatum* (*F. nucleatum*) [66]. *Bacteroides* and *Ruminococcaceae* can contribute to HCC development through promoting inflammatory responses, facilitating toxic substances, and inducing hepatic steatosis [66]. Significant enrichment of *Actinomyces spp*. has been identified in extrahepatic cholangiocarcinoma tissues [108], while chronic *Salmonella typhi* infection significantly correlates with gallbladder cancer development [109]. PC tissues harbor significant *Elizabethkingia* enrichment [110], and *Hungatella hathewayi* can promote cancer by reprogramming host DNA methylation patterns [79]. Beyond digestive system tumors, gut microbiome dysregulation exhibits associations with malignancies in other organ systems. Breast cancer (BC) patients' fecal samples show significantly lower relative abundance of *Bacteroidetes*, *Firmicutes*, and *Faecalibacterium prausnitzii*, with higher levels of *Proteobacteria*, *Actinobacteria*, *Verrucomicrobia*, and *Firmicutes/Bacteroidetes* ratio [111, 112]. Specific *Clostridium* species and *Ruminococcaceae* family members increase BC risk by altering estrogen metabolic pathways [113, 114]. Lung cancer patients show significantly upregulated *Ruminococci* (*R. gnavus*) abundance in fecal microbiome, with squamous cell carcinoma (SCC) patients having higher *Proteobacteria*, *Gammaproteobacteria*, *Bacteroides*, and *Enterobacteriaceae* abundance, while adenocarcinoma patients' feces contain higher *Fusicatenibacter* and *Roseburia* abundance [115]. Prostate cancer (PCa) studies demonstrate a significant correlation between cancer development and relative abundance of *Alphaproteobacteria* and *Bacteroides massiliensis* [116].

Among microorganisms closely associated with gastric tumors, *Helicobacter pylori* (*H. pylori*) is the most extensively documented. *H. pylori* is the most common pathogenic bacterium in gastric tissues, promoting tumorigenesis and progression by inducing DNA damage, interfering with DNA repair mechanisms, and activating oncogenic pathways [64]. Numerous studies confirm *H. pylori*'s significant positive association with gastric, colorectal, and PC risks, leading to its classification as a class I carcinogen by the World Health Organization [117, 118, 119]. Cross‐sectional studies demonstrate that *H. pylori* infection significantly correlates with colorectal cancer (CRC) risk (OR = 1.9), surpassing the associations observed with established risk factors including body mass index (BMI), smoking, and alcohol consumption [120]. Research indicates that both *H. pylori*‐infected CRC patients and mouse models exhibit activation of pro‐carcinogenic signal transducer and activator of transcription 3 (STAT3) signaling, loss of intestinal epithelial goblet cells, reduction in Treg cells, and significant increases in CD3 cells (pro‐inflammatory T cells) [74]. These findings suggest that *H. pylori* functions not merely as a risk indicator for CRC but also establishes a pro‐carcinogenic microenvironment within the colon. Additionally, animal experiments demonstrate that *H. pylori* can increase the abundance of CRC‐associated intestinal bacteriophages, thereby altering the intestinal virome composition, which is associated with CRC development [75]. Cohort studies reveal that serological responses to the *H. pylori* virulence factor VacA exhibit positive correlations with CRC risk [76]. A recent cohort study demonstrated that *H. pylori* has significant positive associations with both CRC incidence and mortality. Individuals with untreated *H. pylori* infection exhibited significantly higher incidence and mortality rates compared to those who received treatment [121]. *Helicobacter hepaticus* has been identified in hepatitis C virus (HCV)‐associated HCC tissue specimens [122, 123], although subsequent research challenged its pathogenic role in HBV‐associated HCC [122]. Mouse model studies have shown that *Helicobacter hepaticus* can inhibit HCC progression by suppressing intrinsic immunity's recognition and clearance functions of tumor cells [124]. The detection of *Helicobacter bilis*, *Helicobacter hepaticus*, or *H. pylori* in biliary tract cancer tissues demonstrates significant correlations with increased cancer risk [109, 125, 126, 127]. Various intestinal microorganisms, including *Bacteroidetes*, *Proteobacteria*, and *Streptococcaceae*, show synergistic effects with *H. pylori* in promoting gastrointestinal tumorigenesis [128]. Interestingly, *H. pylori* exhibits protective effects against Barrett's esophagus, esophageal adenocarcinoma (EAC), and inflammatory bowel disease (IBD) [69, 77, 78]. Multiple studies have demonstrated a negative correlation between *H. pylori* infection and EAC and Barrett's esophagus [129]. A case‐control study established that *H. pylori* infection exhibits strong negative associations with both erosive esophagitis and Barrett's esophagus [68]. A previous community‐based study reached the same conclusion, showing that *H. pylori* infection and CagA^+^ status are negatively associated with newly diagnosed Barrett's esophagus [130]. A subsequent meta‐analysis including 10 studies revealed that among Asian populations, IBD patients had significantly lower H. pylori infection rates compared to non‐IBD patients [78]. The negative association between H. pylori infection and IBD is independent of ethnicity, age, H. pylori detection methods, and previous medication use, while antibiotic use influenced this association [131, 132]. Research suggests that the *H. pylori* genome contains immunomodulatory elements that may explain its protective effect against IBD through suppression of inflammatory responses via downregulation of dendritic cell (DC)‐produced IL‐12 and type I interferon levels [133, 134]. Case‐control studies show that both gastric atrophy and *H. pylori* seropositivity are associated with reduced risk of EAC, Barrett's esophagus, and reflux esophagitis, with this negative correlation persisting in patients without gastric atrophy [135, 136]. A recent study postulates that *H. pylori* eradication may disrupt the inflammatory microenvironment, thereby inhibiting the proliferation of other bacteria and reducing EAC risk [137]. However, some cohort studies have not observed increased EAC risk following *H. pylori* eradication [138].

Conditional pathogens are bacteria that may cause disease when there is an imbalance in the host microbiota homeostasis [139, 140], and they are associated with impaired immune systems. These bacteria can elevate cancer risk and facilitate tumorigenesis and progression through direct DNA damage or by inducing microbiota dysbiosis [6, 141, 142]. *Veillonella* and *Prevotella* are commensal in the gut most of the time, but can also cause disease in the context of the dysregulated immune microenvironment. *Veillonella spp*. and specific species of *Prevotella* are associated with gastric cancer (GC) risk [143]. Elevated levels of *Bacteroides fragilis* [144, 145, 146] and *Streptococcus gallolyticus* (*S. gallolyticus*) [147, 148] were detected in tumor tissues of patients with CRC, and fecal specimens from these patients demonstrated significantly higher prevalence of *Enterococcus faecalis* [149]. Additionally, *enterotoxigenic Bacteroides fragili* (*ETBF*) is highly responsive to inflammatory stimuli and considered a risk factor for CRC [82]. *Enterococcus faecalis* potentially promotes chromosomal instability associated with both sporadic adenomatous polyps and CRC [150].

Hepatobiliary tumors have also been associated with various conditionally pathogenic bacteria. Enrichment of *Fusobacterium*, *Prevotella*, and *Novosphingobium* was observed in extrahepatic cholangiocarcinoma tissues [108]. *Fusobacteria* enrichment was observed in duodenal fluids from PC patients, especially those with short‐term survival [151]. The presence of *Pseudoxanthomonas* in PC tissues correlated with increased density of CD8^+^ T cells and improved overall survival (OS) [152]. In contrast, animal experiments showed *B. pseudolongum* migrating from intestine to pancreas and enriched in PC tissues, obstructing immunity mediated by T cells, and suppressing immune responses to pancreatic tumors [110]. Additionally, large amounts of *Fusobacterium* in PC tumor tissues often indicate poor prognosis [153].

*F. nucleatum* is a common opportunistic pathogen residing in the gastrointestinal tract and oral cavity, associated with multiple cancers, including CRC, PC, oral cancer, and gliomas [40, 80, 110, 154]. This microorganism is significantly enriched in neoplastic tissues compared to healthy mucosa across these diverse cancer types. *F. nucleatum* contributes to tumor development and progression through diverse mechanisms: it reprograms host DNA methylation patterns, potentially facilitating the initiation of PC [79]; enhances CRC metastasis by suppressing anti‐tumor immunity, promoting immune evasion, and modulating the E‐cadherin/β‐catenin signaling pathway via FadA adhesin [81, 154, 155, 156]; recruits tumor‐associated macrophages (TAMs) to the TME via CCL20 upregulation, thereby accelerating the progression of CRC [157]; promotes M2 macrophage polarization and activation [158]; and stimulates cancer cell invasion in PC through autocrine/paracrine pathways [159]. Within the TME, intratumoral *F. nucleatum* modulates immune responses by inducing T helper 17 (Th17) cell enrichment and promoting secretion of IL‐17 family cytokines via the metabolite‐sensing receptor FFAR2, thereby establishing pro‐inflammatory microenvironmental conditions [160]. Clinically, *F. nucleatum* serves as a specific diagnostic and prognostic biomarker in CRC, GC, oral cancer, PC, and lung cancer, with its intratumoral abundance demonstrating a significant negative correlation with OS in CRC patients [40, 161, 162, 163, 164]. Furthermore, *F. nucleatum* induces chemoresistance in CRC, particularly to oxaliplatin, by inhibiting caspase‐mediated cascades via autophagy modulation and BIRC3 upregulation [165, 166, 167]. As previously described, *F. nucleatum* can regulate the expression of β‐catenin [156]. Studies have shown that the β‐catenin signaling pathway, which can be activated through multiple pathways, is associated with the development of resistance to lenvatinib in HCC [168, 169, 170]. Therefore, the modulation of β‐catenin expression by *F. nucleatum* may influence the sensitivity of tumor cells to lenvatinib, although the precision molecular mechanisms and clinical relevance of this association require further rigorous investigation. Conversely, the development of targeted therapeutic approaches, such as employing butyrate derivatives to inhibit bacterial proliferation and adhesion, utilizing engineered bacterial outer membrane vesicles to specifically target *F. nucleatum*, or leveraging its presence to implement immunostimulatory strategies, collectively demonstrates promising therapeutic potential, particularly when integrated with established treatment modalities [165, 171].

### Association of viruses with tumorigenesis

Viruses contribute to tumor development through both direct oncogenic mechanisms and modulation of the host immune microenvironment [172, 173, 174]. The molecular mechanisms span multiple pathways, including oncogenic virus‐induced genetic material damage, microbiota dysregulation, and phage‐mediated metabolic network regulation [175, 176, 177]. Among these, oncogenic viruses function as primary drivers of tumorigenesis, exerting critical regulatory influences on cancer development. Table 2 summarizes the oncogenic mechanisms of major viral types in different tumors and their clinical significance, providing a systematic reference for understanding virus‐mediated tumorigenesis.

**Table 2:** Oncogenic mechanisms of different virus types in different tumors and their clinical significance.

Virus type	Representative viruses	Associated cancer types	Primary carcinogenic mechanisms	Clinical significance	References
Carcinogenic viruses	HBV HCV	HCC	• Induce chronic inflammation and fibrosis; • Regulate cell cycle; • Promote EMT.	HBV/HCV vaccination and antiviral therapy significantly reduce HCC risk.	[175, 178]
	EBV	NPC GC B‐cell lymphoma	• Encode oncoproteins (such as LMP1 and EBNA2); • Modulate gut microbiota metabolites.	EBV DNA detection for early screening of NPC.	[179, 180]
	HPV	CC	• Suppress immune surveillance; • Inhibit lymphocyte cytotoxicity; • Regulate TIME.	The HPV vaccine can prevent CC and other related cancers.	[181]
	HIV	Oral cancer Anal cancer	• Alter local microbiota composition; • Chronic immunosuppression promotes carcinogenesis.	HIV‐infected individuals require intensified cancer screening.	[182]
Phages	*Siphoviridae* *Myoviridae*	CRC	• Regulate gut microbiota composition; • Release bacterial antigens to induce inflammation; • Indirectly promote carcinogenic bacterial proliferation.	Phage community analysis as a potential early diagnostic marker for CRC.	[176, 183, 184]
	*Caudovirales*	CRC	Bidirectional role: • Inhibit carcinogen colonization (anti‐cancer); • Exacerbate inflammation (pro‐cancer) upon overgrowth.	Phage therapy requires precise regulation to avoid inflammatory aggravation.	[185, 186]
Other viruses	EV71	Advanced CRC	• Recruit Th17 cells to promote TME inflammation; • Persistent viral antigen stimulation drives cell proliferation.	EV71‐persistent infection requires enhanced CRC monitoring.	[177]
	MMTV BLV	BC	• Genomic integration activates host oncogenes; • Evade immune clearance.	MMTV/BLV antibody detection as a potential BC risk prediction tool.	[187]
	HCMV	BC Glioma	• Encode oncogenes; • Suppress tumor suppressor proteins.	HCMV‐targeted therapy may enhance chemo/radiotherapy sensitivity.	[188, 189]
	HERV‐K	OC PC HCC	• Retrotransposition induces genomic instability; • Activate pro‐cancer signaling pathways.	HERV‐K expression correlates with tumor prognosis.	[190]

Abbreviations: BLV, bovine leukemia virus; CC, cervical cancer; EBNA2, Epstein–Barr virus nuclear antigen‐2; EBV, Epstein–Barr virus; HBV, Hepatitis B virus; HCV, Hepatitis C virus; HIV, human immunodeficiency virus; HPV, human papilloma virus; EMT, epithelial‐mesenchymal transition; EV71, enterovirus 71; HCMV, human cytomegalovirus; HERV‐K, human endogenous retrovirus‐K; LMP1, latent membrane protein1; MMTV, mouse mammary tumor virus; NPC, nasopharyngeal carcinoma; OC, ovarian cancer; Th17, T helper 17; TIME, tumor immune microenvironment.

#### Carcinogenic viruses and tumorigenesis

Oncogenic viruses represent a specialized category of viruses that infect host cells and interfere with cellular growth regulatory mechanisms, thereby inducing aberrant cell proliferation and ultimately promoting tumor formation [191, 192, 193, 194, 195]. These viruses can promote tumorigenesis through multiple mechanisms, including induction of DNA damage, disruption of DNA damage response (DDR) system, expression of oncogenic proteins, activating cancer‐related signaling pathways, activation of cell cycle control, and dysregulation of apoptosis [196, 197]. Within the spectrum of digestive system oncogenic viruses, HBV and HCV represent the predominant pathogens responsible for HCC. These viruses could promote HCC development and progression through multiple mechanisms, including inducing epithelial–mesenchymal transition (EMT), regulating the cell cycle, and inducing chronic inflammatory responses and tissue fibrosis [175, 178, 198]. Epstein–Barr virus (EBV) demonstrates strong associations with B‐cell lymphoproliferative disorders and nasopharyngeal carcinoma (NPC) [179], while additionally influencing tumor development through modulation of intestinal microbiota and their metabolites [199], as well as contributing to GC progression [180]. Human papillomavirus (HPV) may contribute to cervical cancer (CC) pathogenesis by suppressing immune surveillance and inhibiting cytotoxic responses in lymphocytes, thereby significantly influencing the TIME [181]. Additionally, human immunodeficiency virus (HIV) may contribute to the development and progression of oral and anal cancerous lesions through alteration of regional microbiota composition via mechanisms linked to carcinogenesis [182].

#### Phages and tumorigenesis

In recent years, the role of phages in tumor development has garnered increasing attention, with their regulatory mechanisms exhibiting distinct characteristics across different tumor types [200, 201, 202, 203, 204, 205, 206]. Several studies in CRC have revealed specific alterations in phage communities. Phages of the families *Siphoviridae* and *Myoviridae* were significantly enriched in the feces of CRC patients, while streptococcal phages and Vibrio‐inhabiting phage populations were also abnormally increased [176, 183]. Although the biological significance of temperate phages remains controversial [176], experiments have shown that they may indirectly influence CRC progression through modulation of gut microbial composition and metabolic functions [184].

Dysbiosis in phage communities induces lysis of host bacteria and subsequent alterations in gut microbial abundance, resulting in the release of antigenic substances including proteins, lipids, and nucleic acids from bacteria. This process can induce host inflammatory responses and tissue damage, promoting CRC [185]. Notably, *Caudovirales* phages exhibit dual roles in the intestinal inflammatory environment, both enhancing survival in CRC‐prone animals through inhibition of oncogenic bacterial colonization, while paradoxically exacerbating IBD pathology through their over‐amplification [186]. Certain phages directly interact with cancer cells and modulate the expression levels of proteins involved in carcinogenesis and metastasis, particularly integrins [207]. These findings indicate that phages function as both potential drivers of tumorigenesis and novel bioregulatory targets, though their specific mechanisms warrant comprehensive investigation within the context of cancer‐specific microbial‐immune microenvironments.

#### Other viruses and tumorigenesis

Beyond established oncogenic viruses, diverse viral agents contribute to tumorigenesis and progression through both direct and indirect mechanisms. In patients with persistent enterovirus 71 (EV71) infection, chronic presence of EV71 viral antigen in intestinal tissue significantly correlates with advanced CRC, potentially promoting disease progression through recruitment and stimulation of Th17 cells in the TME [177]. In BC studies, both mouse mammary tumor virus (MMTV) and Bovine leukemia virus (BLV) have been associated with BC development, with studies suggesting a likely causal relationship [187]. Additionally, human cytomegalovirus (HCMV) has been associated with various malignant tumors, including BC, possibly related to potent oncogenes in its genome [188, 189]. Human endogenous retrovirus K (HERV‐K) is highly expressed in various reproductive tumors, including ovarian cancer, and has also been associated with the progression of PC and HCC [190].

### Association of fungi with tumorigenesis

Fungal communities represent a key component of the human microbiome, with their homeostatic imbalances strongly associated with tumor development. Specific fungal species induce inflammatory responses and disrupt immune homeostasis, consequently promoting tumorigenesis and tumor progression [208, 209, 210, 211]. This process manifests primarily through aberrant proliferation of commensal fungi or excessive colonization by pathogenic fungi [212, 213]. As shown in Table 3, distinct tumors exhibit characteristic fungal signatures that correlate significantly with tumorigenesis, progression, and prognosis, providing critical insights into fungal‐mediated tumor pathogenesis and potential therapeutic interventions.

**Table 3:** Characteristic fungal alteration patterns in tumors and their mechanisms.

Cancer type	Characteristic fungal alterations	Mechanism	Clinical relevance	References
GC	Symbiotic fungal imbalance: *Malassezia globosa* ↑ Saccharomyces cerevisiae ↓	• Fungal dysbiosis induces chronic inflammation; • Disrupts mucosal barrier function.	Salivary/tongue coating fungal communities as early screening markers for GC.	[214, 215]
CRC	Pathogenic fungal enrichment: *Candida albicans* ↑ Schizosaccharomyces pombe ↑ *Malassezia spp*. ↑	• Activate Wnt/β‐catenin pathway to promote proliferation; • Secrete pro‐cancer proteins; • Induce cell adhesion gene dysregulation.	• *Candida* abundance correlates with advanced CRC; • Fungal translocation to blood indicates metastasis risk.	[216, 217, 218]
PC	3000‐ fold fungal concentration ↑:	• Complement C3 pathway activation promotes pro‐inflammatory microenvironment.	• Fungal load correlates with poor prognosis; • Potential biomarker for therapeutic resistance.	[219, 220]
	*Malassezia* ↑			
HCC	Opportunistic fungal colonization: *Candida albicans* ↑ *Malassezia furfur* ↑	• Induce oxidative stress; • Synergize with HBV/HCV to promote liver fibrosis.	Fungal‐viral co‐infection accelerates HCC progression.	[217]
BC	Tumor microenvironment dysbiosis: *Candida albicans* infection ↑	• Increase Treg cells to suppress immunity; • Promote angiogenesis.	Antifungal therapy may enhance ICIs efficacy.	[213]
Melanoma	*Saccharomycetales* ↑	• T cells recognize melanoma antigens.	Fungal diversity may correlate with immunotherapy response.	[221]
PCa	Plasma fungal dysbiosis: *Sordariomycetes* ↑	• Promote inflammatory microenvironment.	• Specific fungi may assist in identifying PCa	[222]

Abbreviations: ↑, Increased; ↓, Decreased; ICIs, immune checkpoint inhibitors; IL‐6, Interleukin‐6; PCa, prostate cancer; Treg, regulatory T cells.

#### Symbiotic fungi and tumorigenesis

The human body harbors numerous fungi that establish symbiotic relationships with host tissues, predominantly colonizing mucosal surfaces (including the oral cavity, intestines, and vagina) and skin [223, 224]. These symbiotic fungi serve critical functions in maintaining microbial community equilibrium, regulating metabolic processes, and preserving immune system homeostasis [225]. However, ecological dysbiosis within the fungal microbiome contributes to both initiation and progression of digestive system malignancies through diverse mechanisms, including modulation of the host immune microenvironment and fungal–bacterial interactions [212]. Studies demonstrate that fungal community compositions in saliva and tongue samples from GC patients undergo significant alterations, characterized by marked enrichment of *Malassezia globosa* (*M. globosa*) and concurrent depletion of the symbiotic fungus *Saccharomyces cerevisiae* (*S. cerevisiae*) [214]. Patients with CRC showed not only significant increases in *S. cerevisiae* and *Malassezia spp*. abundance [226, 227] but also elevated levels of proteins secreted by *Schizosaccharomyces pombe*, including four key proteins strongly implicated in tumor progression [216]. In PC studies, fungal concentrations in tumor tissues were approximately 3000‐fold higher than in normal tissues, with *Malassezia spp*. demonstrating significant enrichment and capacity to promote pancreatic tumor growth through the complement pathway. This was particularly pronounced in PC, where genes related to immunity and inflammation exhibited significant upregulation, suggesting a substantial role for *Malassezia* [219, 220] in disease pathogenesis. Additionally, the modulatory role of intestinal fungal microbiota on the TME extends to other cancer types, with melanoma patients demonstrating significantly elevated fungal abundance compared to healthy controls and selective enrichment of *Saccharomycetales spp*. [221].

#### Pathogenic fungi and tumorigenesis

Extensive research has demonstrated that specific pathogenic fungi promote the development of diverse tumor types through multiple oncogenic mechanisms. Additionally, certain commensal fungi transition to pathogenic phenotypes through interactions with other microorganisms, influenced by host immune status and genetic factors [217]. The fungal microbiome of GC patients exhibits significant dysbiosis compared to healthy controls. *Candida albicans* (*C. albicans*) may promote GC by increasing intestinal inflammation, with significantly elevated proportions of *Malassezia*, *Cutaneotrichosporon*, and *Fusicolla acetilerea*, alongside significantly reduced abundance of *Penicillium arenicola*, *Aspergillus montevidensis*, and *C. glabrata* [215, 217, 228]. Patients with CRC exhibit enrichment of *Basidiomycota* and *Ascomycota*, with significant increases in *C. albicans*, *Malassezia*, and *Rhodotorula* abundance. *C. albicans* promotes intestinal epithelial cell proliferation through activation of Wnt signaling pathway, thereby accelerating tumor progression [218, 226, 227]. Elevated *Candida* abundance in advanced CRC patients may lead to dysregulated expression of cell adhesion genes and facilitate translocation of fungal components into the bloodstream [217]. The observation that *Aspergillus rambellii* significantly enhances cancer cell proliferation in vitro and accelerates tumor growth in vivo [229] further substantiates the potential causal relationship between fungal dysbiosis and CRC [217].

Studies in HCC have established that aberrant colonization by *C. albicans* and *M. furfur* promotes tumor development, while *Alternaria alternata* contributes to PC progression [217]. Beyond digestive system malignancies, *C. albicans* infection enhances BC growth by expanding regulatory T cell (Treg) populations within splenocytes and the TME [213]. Patients with head and neck squamous cell carcinoma (HNSCC), GC, CRC, and lung cancer have significant intestinal fungal dysbiosis, characterized by enrichment of opportunistic pathogenic fungi (including *Cutaneotrichosporon*, *Malassezia*, and *Trichosporon*) [221]. The diversity of circulating fungal microbiome in plasma of PCa patients differs significantly from healthy controls, with marked *Sordariomycetes* enrichment observed in PCa cases with high pathological grade of tumor [222].

### Microbial metabolites and tumorigenesis

Microbial metabolites produced by intestinal microbiota can contribute to tumorigenesis and progression through multiple mechanisms, including modulation of host immune responses, regulation of cell proliferation signaling pathways, and alteration of intestinal barrier integrity. Short‐chain fatty acids (SCFAs), secondary bile acids, and tryptophan metabolites represent key microbial products that exert important regulatory effects on the intestinal microenvironment and host health [230, 231, 232, 233, 234, 235, 236]. Table 4 summarizes the mechanisms through which these key microbial metabolites influence diverse tumor types and their potential clinical applications, highlighting recent advances in microbial metabolism within oncological research.

**Table 4:** Key microbial metabolites: mechanisms of action in different tumor types and their potential clinical significance.

Metabolite type	Metabolite	Associated cancer types	Mechanism	Clinical significance	References
SCFAs	Butyrate	CRC	• Promotes abnormal proliferation and transformation of colonic epithelial cells; • Regulates HDAC activity to induce apoptosis in cancer cells.	Probiotics (such as *C. butyricum*) are targeted to deliver butyric acid or as adjunctive therapy.	[237, 238]
	Propionate	BC	• Inhibits STAT3 pathway; • Promote the accumulation of ROS; • Activates p38; • Reverses microbiota dysbiosis inducedby psychological stress.	Sodium propionate combined with chemotherapy enhances efficacy.	[239, 240]
	Anti‐inflammatory SCFAs	HCC	SCFA‐producing bacteria (such as *Faecalibacterium*) decrease, leading to weakened anti‐inflammatory effects and promoting HCC progression.	Fecal microbiota transplantation to restore SCFA levels or inhibit HCC progression.	[91]
	Pentanoate Butyrate	Melanoma Pancreatic cancer	Enhance the anti‐tumor effects of CTLs and CAR‐T cells.	Synergistic cancer immunotherapy.	[241]
Secondary bile acids	Deoxycholic acid	CRC	• Induces DNA oxidative damage and chromosomal instability; • Promotes proliferation of mutagenic bacteria (such as Bilophila).	Monitor deoxycholic acid levels in high‐fat diet patients to prevent adenoma‐to‐carcinoma transition.	[242, 243]
	Lithocholic acid	BC	• Induces oxidative stress to inhibit EMT.	Lithocholic acid analogs as potential therapies for metastatic BC.	[244, 245]
Tryptophan metabolites	Indole derivatives	CRC	• *Akkermansia muciniphila* inhibits AhR/β‐catenin pathway to reduce risk.	Indole derivatives (such as indole‐3‐carbinol) as chemopreventive agents.	[246, 247, 248, 249]
	Kynurenine	Pan‐cancer	• Suppresses T‐cell function to promote immune evasion; • Activates IDO1 pathway to drive tumor progression.	IDO1 inhibitors combined with ICIs therapy.	[250, 251]

Abbreviations: AhR, aryl hydrocarbon receptor; CTLs, cytotoxic T lymphocytes; HDAC, histone deacetylase; IDO1, indoleamine 2,3‐dioxygenase‐1; ROS, reactive oxygen species; SCFAs, short‐chain fatty acids.

#### Short‐chain fatty acids

SCFAs constitute essential intestinal microbial metabolites, primarily consisting of acetic acid, propionic acid, and butyric acid. These compounds exhibit bidirectional regulatory effects on digestive system tumor development through modulation of host immune response, inflammatory processes, and epigenetic modifications [252, 253, 254, 255]. SCFAs may play a significant regulatory role in CRC, particularly butyric acid, which modulates tumor cell proliferation, apoptosis, and invasive capacity through receptor‐mediated signaling, thereby inhibiting intestinal inflammation and carcinogenesis [237]. Additionally, oral probiotic spores (spores‐dex) specifically colonize CRC lesions and generate anti‐tumor SCFAs through *C. butyricum*‐fermented dextran, achieving targeted intervention within the TME [238]. In HCC patients, the abundance of SCFA‐producing commensal bacteria (such as *Faecalibacterium*, *Ruminococcus*, and *Ruminoclostridium*) is significantly reduced, and the decrease in SCFA levels may promote HCC progression through impairment of their anti‐inflammatory effects [91]. Disturbances in bacterial metabolites, including SCFAs, lipopolysaccharides (LPS), and lipoproteins, resulting from microbiota dysbiosis constitute established pathogenic mechanisms in PC development [256]. In BC research, sodium propionate (SP) induces apoptosis and suppresses tumor cell proliferation through inhibiting the STAT3 signaling pathway, enhancement of reactive oxygen species (ROS) accumulation, and activating p38 [239]. Concurrently, psychological stressors including social isolation may diminish SCFA production through disruption of the brain–gut–microbiome axis homeostasis, thereby potentially increasing BC risk [240]. Furthermore, SCFAs demonstrate considerable therapeutic potential for neutrophil dysfunction‐related conditions, including IBD and various malignancies [257, 258]. As critical mediators at the microbiota–tumor interface, imbalances in SCFA levels and functions may contribute to the pathogenesis of digestive and systemic cancers through diverse molecular mechanisms [259, 260, 261, 262, 263].

Microbial‐derived SCFAs modulate the activity and function of immune cells, including lymphocytes, macrophages, DCs, and neutrophils, thereby influencing the TIME and exerting complex bidirectional effects on tumor progression [264]. Consequently, SCFAs represent potential therapeutic targets for cancer therapy. In vitro experiments demonstrate that sodium butyrate (NaB) inhibits lung cancer development and progression through suppression of cancer cell proliferation, induction of tumor cell apoptosis, and modulation of immune responses [265]. SCFAs generated through dietary fiber fermentation by gut microbiota enhance host antibody responses, with their capacity to potentiate B cell immune function documented in both mice and humans [266]. Dietary fiber‐derived SCFAs regulate immune system hematopoiesis, thereby exerting anti‐neoplastic effects [267]. In murine tumor models, SCFAs (pentanoate and butyrate) significantly potentiate the anti‐tumor efficacy of cytotoxic T lymphocytes (CTLs) and chimeric antigen receptor T (CAR‐T) cells, demonstrating potential for synergistic therapy in cancer immunotherapy [241]. SCFAs are significantly reduced in CRC and supplementation with probiotics that metabolize SCFAs can inhibit tumor cell growth. In both mouse CRC models and CRC patients, SCFAs have been observed to enhance host responses to chemotherapy and immunotherapy [268]. Therefore, SCFAs are regarded as substances with potential for adjuvant cancer treatment.

#### Secondary bile acids

Secondary bile acids (including deoxycholic acid [DCA] and lithocholic acid [LCA]) are metabolites generated through microbial transformation of primary bile acid in the gut and may play crucial roles in host immunomodulatory and metabolic networks with complex regulatory effects on digestive system cancers [269, 270, 271, 272, 273, 274]. Studies demonstrate significantly elevated secondary bile acid concentrations in the intestines of patients with CRC [242], potentially driving carcinogenesis through induction of cellular damage including membrane disruption, mitochondrial dysfunction, and genomic mutations [275]. High‐fat diet (HFD) promotes tumor‐promoting properties of mouse colonic mesenchymal stromal cells (MSCs) by increasing primary/secondary bile acid production [276]. Furthermore, secondary bile acid‐enriched microenvironments selectively promote the proliferation of bile salt‐resistant microorganisms (particularly *Bilophila* and *Desulfovibrio*), which accelerate adenoma‐to‐CRC transformation through release of pro‐inflammatory or mutagenic metabolites including hydrogen sulfide (H_2_S) and secondary bile acids [243]. HCC‐related studies show that intestinal bacteria suppress hepatic natural killer T (NKT) cell accumulation, thereby attenuating anti‐tumor immune responses and facilitating hepatic metastasis through mediation of primary‐to‐secondary bile acid conversion [277]. Animal experiments demonstrate that in BC, LCA induces oxidative stress, promotes mesenchymal‐to‐epithelial transformation, inhibits tumor cell proliferation and metastasis, and suppresses cancer cell proliferation [244, 245]. Secondary bile acids may exert context‐dependent effects, both oncogenic and tumor‐suppressive, in digestive and systemic malignancies through mechanisms including direct genotoxicity, immune microenvironment modulation, and microbial interactions [278, 279, 280, 281].

#### Tryptophan metabolites

Tryptophan metabolites (including indoleacetic acid, kynurenine, and tryptamine), as key molecules generated through microbial tryptophan metabolism, exert complex regulatory effects on digestive cancer development via multiple pathways encompassing immunomodulation, inflammatory response regulation, and epigenetic modifications [282, 283, 284, 285, 286, 287, 288]. Tryptophan catabolism, involving this essential amino acid critical for protein synthesis, modulates cancer‐related immune responses and tumor suppression mechanisms [250, 289]. Studies demonstrate that dysregulated microbiota‐mediated tryptophan metabolism strongly correlates with impaired intestinal barrier function, while its metabolites (including indole and indole‐3‐acetic acid) modulate colon cancer progression through regulation of cell proliferation, metastasis, and anti‐inflammatory activity via multiple signaling pathways [246, 247]. Notably, mucin‐producing *Akkermansia muciniphila* significantly reduces CRC development risk in mice through specific inhibition of tryptophan metabolism‐dependent aryl hydrocarbon receptor (AhR)/β‐catenin signaling pathway [248]. Additionally, indole derivatives (such as indole sodium analogs) can modulate IL‐6 expression in colon tumor cell lines through AhR activation [249]. A comprehensive analysis across five cancer types revealed that alterations in plasma tryptophan and its metabolites (such as GABA and melatonin) in cancer patients may interfere with immune responses and contribute to malignant progression [251]. Research further established that compositional differences in gut microbiota between cancer patients and healthy individuals significantly alter tryptophan metabolite profiles, suggesting these metabolites may serve as biomarkers for cancer screening and detection [251].

### Intestinal barrier function alteration and tumorigenesis

**Figure 1:**
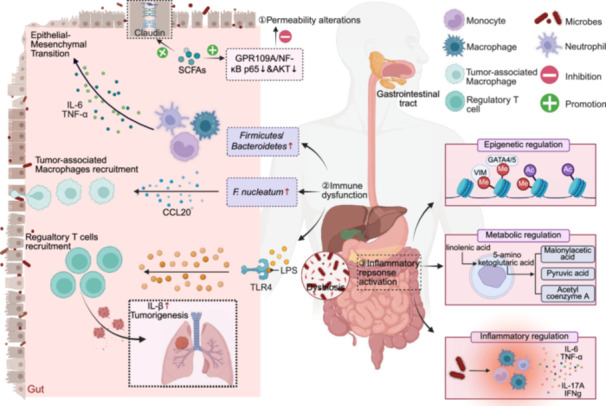
Mechanisms and cancer type‐specific characteristics of tumor‐associated microbiota. Multiple mechanisms induce intestinal barrier function disruption, involving intestinal permeability alteration, immune dysfunction, and inflammatory response activation. Increased intestinal permeability impairs intestinal barrier function and triggers microbial translocation, potentially exacerbating malignancy. SCFAs act as a protectors that maintain intestinal barrier integrity through upregulating transcription of Claudin‐1 protein and activating the GPR109A signaling to downregulate NF‐κB/AKT inflammatory response signaling. Regarding immune dysfunction, an imbalanced *Firmicutes* and *Bacteroidetes* ratio, along with the secretion of pro‐inflammatory factors IL‐6 and TNF‐α, promotes EMT and tumor invasion. Pathogenic bacteria like *F. nucleatum* recruit TAMs through CCL20 upregulation. Gut microbes of lung cancer patients could indirectly promote malignancy deterioration by mediating LPS transport and Tregs recruitment via the gut‐lung axis, while modulating IL‐1β expression in lung tissues. Furthermore, aberrant gene methylation of VIM, GATA4/5, dysregulated histone modifications, abnormal linolenic acid metabolism, and inflammatory microenvironment formation collectively contribute to inflammatory response activation by gut microbes. AKT, protein kinase B; EMT, epithelial‐mesenchymal transition; IL‐1β, interleukin‐1 beta; IL‐6, interleukin‐6; LPS, lipopolysaccharide; NF‐κB, nuclear factor‐kappa B; SCFAs, short chain fatty acids; TAMs, tumor‐associated macrophages; TNF‐α, tumor necrosis factor‐alpha; Tregs, regulatory T cells.

Disruption of intestinal barrier function constitutes a critical driver of tumorigenesis and development [290, 291, 292, 293]. Studies demonstrate that altered intestinal permeability represents a core feature of impaired intestinal barrier function. Additionally, immune dysfunction serves as a pivotal mediator in intestinal barrier disruption processes [294, 295, 296, 297, 298]. Notably, inflammatory response activation constitutes a fundamental molecular mechanism underlying altered intestinal barrier function [299, 300]. Importantly, intestinal barrier disruption resulting from microbiota dysbiosis contributes to cancer development and progression through multiple pathways. In summary, intestinal barrier function disruption establishes a critical link between microbiome and tumorigenesis through multiple interconnected processes, including enhanced intestinal permeability, immune system dysregulation, and inflammatory cascade activation, a complex pathophysiological sequence with molecular mechanisms illustrated in Figure 1.

#### Intestinal permeability alteration

Increased intestinal permeability represents a cardinal pathological feature of microbe–host interactions in digestive cancer pathogenesis, operating through multiple mechanisms including facilitation of microbial translocation, induction of inflammatory cascades, and enhancement of carcinogen exposure [301]. Studies show that tight junctions (TJ) between colonic epithelial cells function cooperatively with the epithelial cells to maintain intestinal barrier stability, and increased TJ permeability correlates with CRC development and progression [302]. Regular probiotic supplementation potentially prevents CRC through the reduction of intestinal permeability and diminished carcinogen absorption [303]. Hepatic research demonstrates that obesity‐induced intestinal hyperpermeability facilitates dysbiosis, promotes chronic inflammatory responses, and consequently accelerates CRC initiation and progression [304, 305]. Intestinal inflammation induces altered intestinal permeability, while compromised barrier function permits bacterial translocation, thereby exacerbating malignancy through the establishment of chronic inflammatory states [306]. In contrast, SCFAs serve a crucial function in maintaining intestinal barrier integrity. Butyrate protects intestinal epithelial cells, alleviates local inflammatory responses, and enhances barrier function through stabilization of hypoxia‐inducible factor‐1 (HIF‐1) and increasing transcription of Claudin‐1 protein (an integral membrane protein present in epithelial and endothelial cells) [307, 308]. Additionally, NaB activates the GPR109A signaling pathway and while concurrently downregulating NF‐κB p65 and AKT signaling cascades, thereby suppressing intestinal inflammation and restoring epithelial barrier function [309]. Modulation of intestinal permeability constitutes a critical interface between microbiota dysbiosis and tumorigenesis. Consequently, targeted interventions aimed at intestinal barrier restoration or microbiome manipulation represent promising therapeutic strategies for prevention and treatment of gastrointestinal malignancies.

#### Immune dysfunction

As a fundamental pathological consequence of dysregulated microbiota–host interactions, immune dysfunction exerts multifaceted influences on digestive cancer development through modulation of immune cell polarization, activation of inflammatory signaling pathways, and orchestration of cross‐organ immune responses [310, 311]. Perturbation of the gastric micro‐ecosystem, encompassing bacterial, fungal, and viral community dysbiosis, induces immune dysfunction within the gastric mucosa, characterized by diminished commensal microbes and enhanced abundance or virulence of pathogenic microorganisms, consequently promoting carcinogenesis through sustained inflammatory responses and immune dysregulation [312]. Concurrently, pathogenic bacteria such as *F. nucleatum* can induce intestinal immune dysfunction, thereby accelerating CRC progression [157]. Additionally, research has established that the gut microbiome of lung cancer patients indirectly promotes tumor development through mediation of LPS transport and Tregs recruitment via the gut–lung axis, modulating IL‐1β expression in pulmonary tissues and disrupting metabolic‐immune homeostasis [313]. The altered immune microenvironment and dysregulated immune responses resulting from microbial dysbiosis facilitate the TME establishment and maintenance in gastrointestinal malignancies and distant anatomical sites.

#### Inflammatory response activation

The bidirectional interplay between chronic inflammation and microbiota dysbiosis exerts sustained influence on gastrointestinal cancer initiation and progression [314, 315, 316]. Studies demonstrate that IBD, comprising Crohn's disease (CD) and ulcerative colitis (UC), functions as a significant precursor condition for CRC development [316, 317, 318, 319, 320, 321]. The pathophysiological mechanisms underlying progression from IBD to colitis‐associated CRC encompass multiple integrated factors. Factors including immune cell dysfunction, intestinal epithelial cells alterations, and microbial dysbiosis collectively potentiate inflammatory responses and drive carcinogenic processes [322]. Intestinal microbiota dysregulation in IBD patients manifests through epigenetic mechanisms (including aberrant gene methylation of *VIM*, *GATA4/5*, and dysregulated histone modifications) and metabolic pathway perturbations (particularly disrupted linolenic acid metabolism), thereby promoting carcinogenesis [323, 324]. Probiotics, particularly *Lactobacillus bulgaricus* (*L. bulgaricus*), significantly attenuate tumor progression in CAC models through the reduction of pro‐inflammatory mediators, including IL‐6 and TNF‐α [325]. Dysregulation of the *Firmicutes/Bacteroidetes* ratio, especially significantly increased *Bacteroidetes* abundance, promotes EMT and enhances tumor growth, invasion, and metastasis through the induction of pro‐inflammatory cytokine secretion, including IL‐6 and TNF‐α [157]. Meanwhile, *Lactobacillus plantarum* and *Lactococcus lactis* show dual application potential in IBD treatment and CRC prevention by modulating the intestinal immune microenvironment [326]. Concerningly, even in patients with primary sclerosing cholangitis (PSC) without typical IBD symptoms, elevated intestinal inflammatory factors such as *IL‐17A* and *IFNG*, along with microbiota dysbiosis, may still increase CRC risk [327]. Additionally, anti‐inflammatory diets (especially bioactive substances like flavonoids) exert anti‐tumor effects through multiple mechanisms including inflammatory response inhibition, intestinal barrier integrity maintenance, and immune cell function regulation [328, 329]. These findings indicate the central role of inflammatory microenvironment and microbiota interactions in digestive system tumorigenesis, providing an important theoretical basis for developing cross‐cancer prevention and treatment strategies.

## INTRATUMORAL MICROBIOME

### Characterization of bacteria in tumors and mechanisms of tumorigenesis

**Figure 2:**
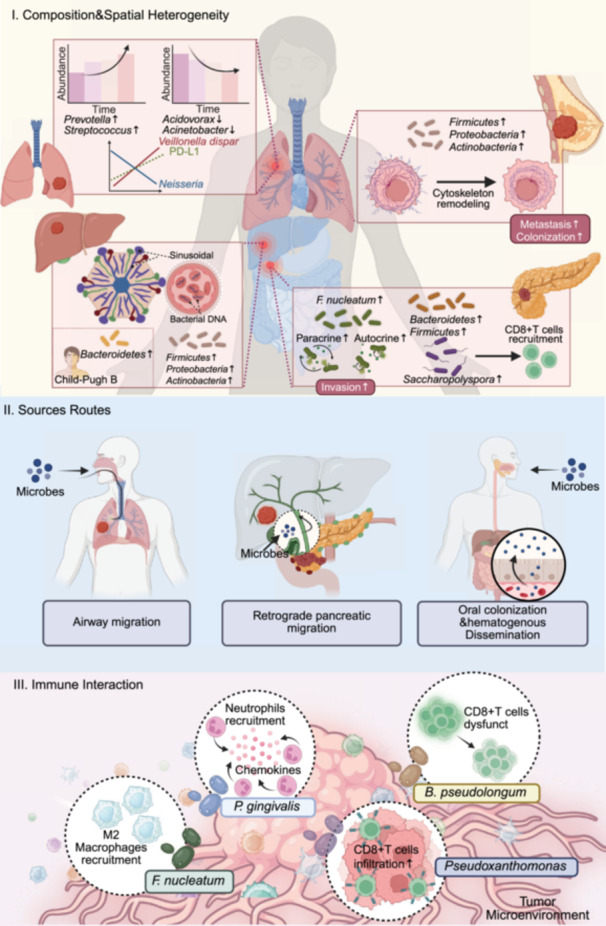
Composition and spatial heterogeneity, invasion routes, and immune interactions of bacterial communities in different tumor types. *Prevotella* and *Streptococcus* show significantly higher abundance in lung cancer, while *Acidovorax* and *Acinetobacter* show decreasing trends. *Veillonella dispar* shows positive correlation with high PD‐L1 expression in lung cancer, while *Neisseria* predominates in patients with low PD‐L1 expression. In HCC, *Actinobacteria*, *Proteobacteria*, and *Firmicutes* show abundance with *Bacteroidetes* higher in Child‐Pugh B classification liver tissue, and bacterial DNA specifically accumulates within peritumoral hepatic sinusoidal erythrocytes. Breast cancer exhibits significant enrichment of *Firmicutes*, *Proteobacteria*, and *Actinobacteria*, which regulate cytoskeletal reorganization and subsequent tumor metastasis. In PC, *F. nucleatum* promotes malignant cell invasion via autocrine/paracrine pathways. Additionally, elevated levels of *Elizabethkingia*, *Pseudomonas spp*., and specific *Saccharopolyspora* enhance anti‐tumor immunity through increased CD8^+^ T cell infiltration. Regarding bacterial colonization routes, colorectal cancer microbiota originates from both oral bacteria via circulation and gut bacteria through hematogenous transfer. Lung tumor microbiota derives from airway microbes, while PC‐associated bacteria utilize retrograde migration through the pancreatic duct to trigger metastasis. The intratumoral microbiota exhibits complex cross‐talk with the immune microenvironment. Regarding pro‐tumor responses, *F. nucleatum* associates with M2 macrophage activation, while *P. gingivalis* triggers tumor‐associated neutrophil recruitment through chemokines and elastase secretion. Regarding anti‐tumor immunity, *B. pseudolongum* impairs CD8^+^ T cell function, whereas like *Pseudoxanthomonas* positively correlate with CD8^+^ T cell infiltration. *B. pseudolongum*, *Bifidobacterium pseudolongum*; HCC, hepatocellular carcinoma; PD‐L1, programmed death‐ligand 1.

The characterization of bacteria within tumors and their oncogenic mechanisms reveals a complex multilevel biological regulatory network. Bacterial communities in different tumor types have unique compositional characteristics. The complex interactions between bacteria and the TME, and their involvement in tumor development through multiple signaling pathway regulation [330, 331, 332], are outlined in Figure 2. It illustrates the carcinogenesis process from bacterial community compositional heterogeneity to microenvironmental interactions. This framework provides a systematic approach for understanding the multidimensional role of the microbiome in tumor development.

#### Characterization of bacterial community composition

The intratumoral microbiome exhibits significant compositional heterogeneity across different cancer types [333]. For instance, the *Firmicutes/Bacteroidetes* ratio is highest in precancerous lesions of gastric cancer (PLGC) mouse models, while GC groups show increased abundance of *Proteobacteria* and *Actinobacteria* [334]. Research indicates that tumor‐associated microbiota in HCC patients is predominantly composed of *Actinobacteria*, *Proteobacteria*, and *Firmicutes*, while *Bacteroidetes* shows higher abundance specifically in Child‐Pugh grade B HCC [335]. Studies suggest that an elevated *Firmicutes/Bacteroidetes* ratio is associated with favorable responses to nutritional interventions [336]. This microbial diversity closely relates to tumor biological characteristics and patient prognosis. Multiple studies demonstrate that bacteria exhibit specific spatial distribution patterns within tumor tissues. Gram‐negative bacteria primarily localize in cancer cells and immune cells, whereas Gram‐positive bacteria are mainly detected in melanoma and preferentially appear in macrophages [26]. In HCC, bacterial DNA predominantly enriches in peritumoral hepatic sinusoidal erythrocytes, whereas within the tumor it mainly localizes in the hepatocyte cytoplasm [337]. This spatial distribution difference directly affects biological functions. For example, *F. nucleatum* in PC promotes cancer cell invasion via autocrine/paracrine pathways [159], whereas microbes in BC enhance metastatic colonization of tumor cells by regulating cytoskeletal reorganization [338]. Bacterial load and abundance in BC tissues are significantly higher than in adjacent normal tissues and most other cancer subtypes. This enrichment is particularly evident for viable bacteria of *Firmicutes*, *Proteobacteria*, and *Actinobacteria*, with *F. nucleatum* showing specific enrichment in BC and PC [158]. However, Jeongshin A et al. found that the *Firmicutes/Bacteroidetes* ratio is threefold lower in BC patients compared with healthy individuals and is considered a risk factor for BC [339]. *Corynebacteriaceae* and *Micrococcaceae* abundance is significantly higher in non‐gastrointestinal tumors (such as BC and osteosarcoma), whereas *Proteobacteria*, *Bacteroidetes*, and *Firmicutes* dominate the PC microenvironment [26, 110, 158]. In CRC, microbiota community analysis can help determine clinical subtypes and prognostic subgroups. Among stage II/III cases, tumors characterized by *Firmicutes/Bacteroidetes* communities demonstrate better OS for microsatellite stable tumors compared with other microbial subtypes (*Fusobacterium*/oral pathogens; *Escherichia/Pseudescherichia/Shigella*) [340]. In CRC, mice supplemented with kefir exhibited decreased *Firmicutes/Bacteroidetes* and *Ascomycota/Basidiomycota* ratios, with improved CRC outcomes [341]. For patients with locally advanced non‐small cell lung cancer (NSCLC) receiving concurrent chemoradiotherapy, early nutritional intervention can improve host nutritional status and reduce post‐treatment adverse reactions. In lung cancer, microbiota compositional features exhibit significant diagnostic and prognostic value. Compared with normal lung tissues, genera such as *Prevotella* and *Streptococcus* show significantly higher abundance in lung cancer, while genera such as *Acidovorax* and *Acinetobacter* show decreasing trends. Particularly, *Thermus* shows cumulative enrichment in patients with advanced (stage IIIB/IV) disease, and *Legionella* are significantly enriched in metastatic cases, suggesting that microbiota compositional dynamic changes are closely associated with disease progression [342, 343]. Regarding prognosis, *Veillonella dispar* in bronchoalveolar lavage fluid (BALF) of lung cancer patients shows positive correlation with high PD‐L1 expression, while *Neisseria* is enriched in the low PD‐L1 expression group [344].

Notably, intratumoral microbial diversity demonstrates a significant negative correlation with immune microenvironment parameters (such as tumor‐infiltrating lymphocytes [TILs] and PD‐L1 expression) and exhibits a clear association with shorter overall patient survival [345]. In normal pancreatic tissues, microbial abundance is low and predominantly in extracellular colonization form, whereas *Elizabethkingia* and *Pseudomonas spp*. are significantly elevated, and enrichment of specific genera (such as *Saccharopolyspora*) improves anti‐tumor immune responses by enhancing CD8^+^ T cell infiltration in PC [110, 152, 346]. Together, these findings reveal the critical role of intratumoral microbiota in spatiotemporal heterogeneity, immune regulation, and clinical translation, providing new research directions and ideas for precision oncology.

#### Bacterial and tumor microenvironment interactions

The origin of tumor microbiota remains controversial, and its distributional characteristics likely vary depending on tumor type. Studies have demonstrated that gut microbes can reach tumors via hematogenous transfer, and oral microbes that colonize tumors through the circulatory system (such as *F. nucleatum* from oral cavity to CRCs) constitute important tumor microbiota sources [347, 348]. Microbiota from adjacent healthy tissues also represents an important source. For example, one study showed that lung tumor microbes originated from the airways [349]. Additionally, bacteria in PC can migrate retrogradely through the pancreatic duct [350].

Intratumoral microbiota forms a complex interaction network with the TME through immune regulation [32, 351, 352, 353, 354]. The intratumoral microbiota exhibits a clear double‐edged sword effect. Regarding pro‐tumor responses, *F. nucleatum* is closely associated with M2 macrophage activation in a specific tumor type; *Porphyromonas gingivalis* (*P. gingivalis*) attracts tumor‐associated neutrophils by secreting neutrophil chemokines and stimulates their release of pro‐tumorigenic neutrophil elastase [158]. Additionally, *Bifidobacterium pseudolongum* (*B. pseudolongum*) suppresses T‐cell immunity, whereas microbes such as *Pseudoxanthomonas* show a significant positive correlation with CD8^+^ T cell infiltration [158].

### Mechanisms of viral infection and tumorigenesis in tumors

**Figure 3:**
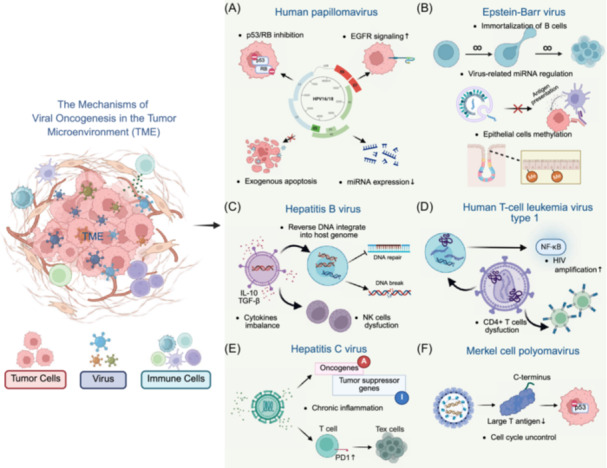
Mechanisms of viral oncogenesis in the TME. The schematic illustration demonstrates how different oncogenic viruses modulate the TME to promote carcinogenesis. (A) HPV induces oncogenesis through multiple mechanisms, including p53/RB inhibition, enhanced EGFR signaling, suppression of miRNA expression, and regulation of exogenous apoptosis. (B) EBV promotes tumor development via B cell immortalization, virus‐related miRNA regulation inhibiting antigen presentation, and ECs methylation altering the stability of ECs. (C) HBV integrates its reverse DNA into the host genome, triggers DNA breaks, induces cytokine imbalance (IL‐10, TGF‐β), and leads to NK cell dysfunction. (D) HTLV‐1 induces CD4^+^ T cell dysfunction and activates NF‐κB signaling, resulting in enhanced HIV amplification. (E) HCV contributes to oncogenesis through chronic inflammation, activation of oncogenes, inhibition of tumor suppressor gene expression, and upregulation of PD‐1 on T cells, leading to exhausted T cell transformation. (F) MCPyV promotes cell cycle dysregulation through C‐terminus‐mediated large T antigen reduction and p53 down‐regulation. EBV, Epstein‐Barr virus; ECs, epithelial cells; EGFR, epidermal growth factor receptor; HBV, Hepatitis B virus; HCV, Hepatitis C virus; HIV, Human immunodeficiency virus; HPV, Human papillomavirus; HTLV‐1, Human T‐cell leukemia virus type 1; IL‐10, interleukin‐10; MCPyV, Merkel cell polyomavirus; miRNA, microRNA; NK, natural killing; PD‐1, programmed cell death protein 1; Tex, exhausted T cell; TGF‐β, transforming growth factor‐beta; TME, tumor microenvironment.

Investigations of tumor‐associated viral infection processes and their oncogenic mechanisms provide critical insights into the complex interactions between microorganisms and their hosts [355, 356, 357]. Current studies have identified numerous viruses with established oncogenic potential, including HBV, HCV, EBV, HPV, Kaposi's sarcoma‐associated herpesvirus (KSHV), human T‐cell leukemia virus type 1 (HTLV‐1), and Merkel cell polyposis virus (MCPyV), among others. Notably, initial oncogenic virus infection in humans typically does not directly lead to tumorigenesis. However, under conditions of immunosuppression or in synergy with other oncogenic factors, these viruses can promote malignant cell transformation through multiple mechanisms. Figure 3 comprehensively illustrates the molecular mechanism network and interactions among major oncogenic viruses through three core mechanisms: infection pattern establishment, genome modification, and immune escape to induce tumorigenesis.

#### Virus infection patterns

Virus‐mediated tumorigenesis is intricately linked to the mode of viral infection. The mode involves a systematic biological process comprising host cell invasion, establishment of persistent infection, and induction of malignant transformation. The core mechanisms underlying this process reside in the molecular interactions between viruses and host cells, as well as the dynamic regulation of viral latency. For example, high‐risk HPV types (especially types 16 and 18) integrate their viral genome into host cell DNA to induce CC transformation [358]. The oncogenic proteins encoded by these viruses, E6/E7, drive the development of epithelial tumors such as CC through the specific degradation of tumor suppressors, notably p53 and RB. In the B‐lymphocyte transformation paradigm, viral infection promotes B‐cell immortalization [359], enables evasion of apoptotic regulation, and activates proliferative signaling pathways, ultimately leading to hematological malignancies like lymphomas. HBV infection specifically targets hepatocytes, wherein the virus completes DNA replication via reverse transcription and subsequently integrates into the host genome [360]. Hepatocytes harboring these integrated viral fragments acquire proliferative advantages which, in concert with a chronic inflammatory microenvironment, synergistically promote HCC development. In T‐cell leukemia, HTLV‐1 induces clonal expansion and genomic instability through infection of CD4^+^ T cells and subsequent integration of its viral genome into host cellular DNA. The viral Tax protein of HTLV‐1 constitutively activates the NF‐κB pathway, ultimately triggering adult T‐cell leukemia/lymphoma [361, 362]. MCPyV initiates infection through cutaneous transmission, followed by viral DNA integration into the host genome, resulting in expression of the large T (LT) antigen that interacts with tumor suppressor p53 through its C‐terminal domain. Truncation of this replicative domain disrupts anti‐tumor surveillance, resulting in dysregulated cell cycle control and subsequent Merkel cell carcinoma (MCC) pathogenesis [363]. Although these viruses display tropism for distinct target cell populations, they employ convergent mechanisms to achieve malignant transformation, including genomic integration, expression of viral oncoproteins, and subversion of host signaling pathways, underscoring the fundamental relationship between viral infection dynamics and carcinogenesis.

#### Virus‐mediated genome modifications

Virus‐mediated genome modification, as an important molecular mechanism of tumorigenesis, primarily interferes with host cell homeostasis through two pathways: viral genetic material integration and epigenetic regulation, thus promoting abnormal cell proliferation. Among DNA viruses, HPV achieves malignant transformation through integration into host chromosomal DNA, whereby its viral oncoproteins E5, E6, and E7 cooperatively promote carcinogenesis through enhancing aberrant EGFR signaling, causing p53 and RB dysfunction, deregulating microRNA expression, and inhibiting exogenous apoptosis [364]. EBV, with its unique epigenetic regulation in gastric carcinogenesis, including hypermethylation features of EBV‐associated GC (EBVaGC), induces aberrant methylation patterns in gastric epithelial cells. This effect silences tumor suppressor genes and cell cycle regulators while concurrently repressing differentiation‐associated transcriptional programs, ultimately promoting a poorly differentiated, highly proliferative precancerous cellular state [365]. The oncogenic mechanism of HBV closely relates to its genome integration. During early clonal amplification stages, HBV DNA integration into host genome triggers insertional mutations and drives genome instability which, synergistically with HBV‐encoded X protein (HBx)‐mediated effects (including impairment of DNA damage repair mechanisms and inactivation of tumor suppressors), collectively accelerates HCC progression [366, 367, 368, 369, 370]. RNA viruses lack direct genomic integrative capacity but nevertheless compromise host genome stability through alternative molecular mechanisms. HCV induces genetic and epigenetic disorders through its viral protein repertoire, facilitating both oncogene activation and tumor suppressor silencing. Meanwhile, HCV simultaneously establishes a chronic inflammatory microenvironment through its persistent infection that constitutes an important factor for HCC development [371, 372]. In summary, whether mediated through direct genomic integration by DNA viruses or epigenetic reprogramming by RNA viruses, virus‐mediated genome modification ultimately promotes tumor invasion and metastasis through common pathways such as metabolic abnormalities, cell cycle dysregulation, and genetic instability.

#### Virus‐induced immune escape

Virus‐mediated immune escape mechanisms represent critical determinants of oncogenesis, wherein diverse viral pathogens establish persistent infections and induce tumorigenesis by specifically targeting distinct components of immune surveillance networks [373]. HPVs evade host immune clearance mainly through strategies of spatial immune escape. Their viral gene expression and viral particle production are strictly confined to the most superficial epithelial tissue layer, which is inherently subject to limited immune cell surveillance [374, 375]. EBV evades immunological clearance through expression of specialized viral microRNAs that modulate host immune responses and antigen presentation, effectively circumventing immune surveillance and facilitating lymphomagenesis and other related malignancies [376]. Among HCC‐associated viruses, HBV achieves immune evasion through remodeling the TME. Its chronic infection can induce functional exhaustion of intrahepatic immune cells (particularly NK cells) and establish immunosuppressive cytokine networks (characterized by increased IL‐10 and TGF‐β), thereby promoting malignant hepatocyte clonal expansion while attenuating antiviral immune responses [377]. HCV‐induced persistent chronic inflammation and tissue fibrosis accelerate cirrhosis, while concurrent chronic inflammatory signaling drives T cell exhaustion and aberrant upregulation of PD‐1, ultimately establishing immune tolerance and promoting HCC progression [371]. These complex molecular mechanisms demonstrate how viruses circumvent host immune surveillance through multidimensional strategies, including immune microenvironment restructuring, subversion of immune signaling networks, and induction of immune tolerance.

### Intratumoral fungi and tumor progression

The mechanism by which intratumoral fungi influence tumor progression can be systematically analyzed across three interconnected dimensions: specific distribution patterns of fungal communities within the TME, interaction mechanisms between fungi and the TIME, and fungal‐mediated metabolic reprogramming of tumor cells. These mechanistic dimensions exhibit substantial interconnectivity and synergism, collectively orchestrating the complex processes underlying tumor development and progression.

#### Characterization of fungal communities

Fungal community composition and spatial distribution exhibit significant heterogeneity across diverse tumor types. Macrogenome sequencing‐based analyses have demonstrated that various fungi, including *C. albicans*, *Malassezia*, and *S. cerevisiae*, are prevalent across multiple human malignancies, including BC, melanoma, and PC [378]. Fungal abundance is significantly elevated in all examined tumor tissues compared with corresponding normal tissues, with fungal species predominantly localized within intracellular compartments of tumor cells and tumor‐associated macrophages [158]. Each cancer type shows distinct fungal community signatures: *Saccharomycetes* abundance is higher in colon cancer, melanoma shows predominant enrichment of *Malasseziomycetes* [158], and PC tissues contain approximately 3000‐fold higher fungal concentrations compared to normal pancreatic tissues, with *Malassezia* significantly enriched in both human specimens and murine models [219]. Additionally, *Candida* is detected in GC tissues, with its increased abundance correlating with early‐stage GC progression, while *Blastomyces* demonstrates a significant association with lung cancer pathogenesis [215].

#### Interaction between fungi and the tumor immune microenvironment

Intratumoral fungal communities actively modulate tumor–host interactions through direct regulation of the TIME [379, 380]. Studies have demonstrated that *Alternaria alternata* and *M. globosa* induce cancer cell IL‐33 secretion through activation of Src‐Syk‐CARD9‐NF‐κB signaling cascade, which subsequently promotes the migration of Th2 cells and type 2 innate lymphoid cells (ILC2) into the TME. The subsequent secretion of pro‐tumorigenic cytokines, including IL‐4, IL‐5, and IL‐13, by these activated immune cells further accelerates tumor growth and progression [381, 382]. These findings establish that intratumoral fungi function as critical pro‐tumorigenic mediators within tumor‐immune interactions through modulation of key signaling pathways and orchestration of cytokine networks [158].

### Regulation of the tumor microenvironment by microbial metabolites

**Figure 4:**
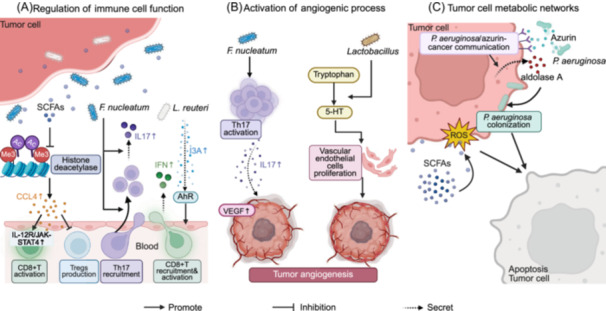
Regulation mechanisms of the TME by microbial metabolites. This schematic illustrates three core mechanisms (reagulation of immune cell function, activation of angigenic process, and metabolic networks) by which microbial metabolites modulate the tumor microenvironment. (A) SCFAs inhibit histone deacetylase, leading to increased CCL4 production and subsequent modulation of CD8^+^ T cell activation and Tregs decrease via IL‐12/JAK‐STAT4 signaling. *F. nucleatum* promotes Th17 cell recruitment and IL17 production, while *L. reuteri* also affecting CD8^+^ T cell recruitment and activation through indole 3 aldehyde and IFN‐γ pathways mediated by AhR signaling. (B) *F. nucleatum* activates Th17 cells, leading to increased IL17 and VEGF expression. In addition, *Lactobacillus* metabolizes tryptophan to 5‐HT, ultimately promoting vascular endothelial cell proliferation and tumor angiogenesis. (C) *P. aeruginosa* enrichment leads to azurin secretion, which triggers tumor cells to produce aldolase A. This creates a feedback loop that further promotes *P. aeruginosa* colonization on the tumor surface, collectively contributing to tumor cell apoptosis. Furthermore, SCFAs generate ROS, which also contribute to tumor cell apoptosis through oxidative stress mechanisms. AhR, aryl hydrocarbon receptor; IFN‐γ, interferon γ; IL12, interleukin‐12; IL17, interleukin‐17; *L.reuteri*, *Lactobacillus reuteri*; ROS, reactive oxygen species; Th17, T helper 17; VEGF, vascular endothelial growth factor.

Microbial metabolites function as multifaceted regulators within the TME, exerting diverse biological effects across multiple cellular compartments. These metabolites can modulate immune cell phenotype and function, mediating immune responses in the TME that significantly influence tumor progression trajectories [383, 384, 385, 386, 387]. Notably, microbial metabolites substantially influence tumor growth, invasion, and metastatic potential through the regulation of angiogenic processes and vascular remodeling. Furthermore, microbial metabolites directly interact with tumor cells and modulate cellular metabolic pathways, underscoring the complexity and diversity of these compounds within the TME regulatory network. As shown in Figure 4, microbial metabolites establish an intricate regulatory network through modulation of immune cell function, orchestration of angiogenic processes, and reprogramming of tumor cell metabolism, collectively shaping the functional characteristics of the TME and ultimately influencing tumor development.

#### Metabolites and immune cells

The TIME is a dynamic microenvironment system comprising immune cells that infiltrate tumor tissues and their secreted cytokines, chemokines, and other bioactive molecules [388, 389, 390, 391]. Evidence indicates that microbial metabolites can regulate immune cell function and phenotype through various pathways, thereby contributing to the TIME remodeling processes. SCFAs are produced when gut microbes ferment dietary fiber. Butyrate, a key member of the SCFA family, exhibits significant immunomodulatory effects and accumulates in tumor tissues. In the TIME, butyrate enhances anti‐tumor immune responses through inhibiting HDAC activity, upregulating CCL4 expression, promoting CD8^+^ T cell activation, and enhancing IL‐12R signaling pathway activity. Additionally, it also reduces Tregs and promotes anti‐PD‐L1 effects, thereby augmenting anti‐tumor efficacy [392, 393]. In a CRC mouse model, intratumoral *F. nucleatum* was found to affect the TIME through the metabolite‐sensing receptor FFAR2 [381]. A study in melanoma demonstrated that after *Lactobacillus reuteri* (*L.reuteri*) colonization of tumor cells, its metabolite indole 3 aldehyde (I3A) promoted CD8^+^ T cell activation and recruitment while elevating interferon γ (IFN‐γ) levels through an AhR signaling‐dependent pathway, thereby enhancing anti‐tumor immune responses and immune checkpoint inhibitor (ICI) efficacy [394]. These findings elucidate the multidimensional regulatory role of microbial metabolites in TIME modulation and establish a theoretical foundation for developing metabolic intervention‐based tumor treatment strategies.

#### Metabolites and angiogenesis

Microbes and their metabolites significantly shape the TME through modulation of angiogenic pathways [395]. In a CRC mouse model, *F. nucleatum* within tumor cells stimulates IL‐17 secretion via metabolite receptors, promoting the formation of an inflammatory TME. IL‐17 can act on tumor cells to promote angiogenic factor VEGF secretion, which induces tumor angiogenesis and accelerates CRC progression [160, 396]. *Lactobacillus spp*. in tumor cells have shown a strong association with immunosuppression in PC and the immunosuppressive TME in esophageal squamous cell carcinoma (ESCC), significantly correlating with poor patient prognosis [397, 398]. *Lactobacillus* converts tryptophan into an intermediate product, which promotes 5‐hydroxytryptamine (5‐HT) synthesis, a metabolite that stimulates vascular endothelial cell proliferation and thus promotes tumor angiogenesis [399, 400]. These findings reveal that microbial metabolites influence tumor evolution by regulating tumor vascular homeostasis.

#### Metabolites and tumor metabolism

Microbial metabolites regulate TME homeostasis through comprehensive remodeling of tumor metabolic networks [268, 401, 402]. Microbial metabolites accumulate within tumor tissues and modulate tumor cell metabolic processes through specific interactions with cancer cell surface receptors, thus establishing a distinct “tumor‐microbial microenvironment.” Butyrate directly affects tumor cell metabolic processes independently of its established effects on the immune microenvironment. Studies have demonstrated that butyrate induces lipid peroxidation and promotes ROS generation in HT‐29 colon cancer cells, consequently promoting tumor cell apoptosis [403]. Recent investigations have elucidated the bidirectional signaling mechanisms between intratumoral microbes and cancer cells. Azurin protein secreted by *P. aeruginosa* disrupts cancer cell signaling pathways through binding to specific receptor molecules on cellular surfaces, thereby inducing apoptotic cascades. Conversely, cancer cells facilitate *P. aeruginosa* colonization within tumor tissues through aldolase A‐mediated metabolic processes [404]. These findings establish that microbial metabolites significantly influence tumor progression and development through direct modulation of tumor cell metabolic pathways.

## ORGAN‐SPECIFIC MICROBIOME AND TUMORS

Organ‐specific microbiomes function as critical determinants in tumorigenesis and development. Studies show that distinct microbial communities at different anatomical sites exhibit specific association patterns with neoplastic transformation in corresponding tissues [405, 406, 407, 408, 409, 410, 411, 412]. Microbial dysbiosis at multiple anatomical sites, including the oral cavity, skin, genitourinary system, and respiratory tract, promotes tumor progression through multiple pathways, including induction of inflammatory responses, production of oncogenic metabolites, and activation of immune escape pathways. The oncogenic role of specific pathogenic microorganisms, particularly HPV in cervical and oral cancers, has been extensively documented, while the broader microbiome significantly influences patient responses to immunotherapeutic and chemotherapeutic interventions. Table 5 summarizes the correlated characteristics between major organ‐specific microbiomes and corresponding malignancies.

**Table 5:** Characteristics associated with organ‐specific microbiome and corresponding tumor.

Organ system	Key microbes	Associated cancer types	Core mechanisms	References
Oral	*Porphyromonas gingivalis* *Fusobacterium nucleatum* *Streptococcus* *C. albicans* HPV‐16/18	OSCC	• Chronic inflammation (NF‐κB activation); • Carcinogenic metabolites (such as acetaldehyde) and butyrate; • HPV‐mediated inhibition of DNA repair and induction of genomic instability.	[413, 414, 415, 416, 417, 418]
Skin	*Staphylococcus aureus* β‐HPV *Staphylococcus epidermidis*	SCC Melanoma	• Release of virulence factors and pro‐proliferative cytokines; • Secretion of 6‐N‐hydroxylaminopurine to inhibit tumor DNA synthesis.	[419, 420, 421, 422]
Urogenital	HPV *Veillonella* *Bifidobacterium* *Lactobacillus*	CC BCa PCa	• HPV integration causing genomic instability; • Microbiome dysbiosis activating; IGF‐1/TLR pathways; • SCFAs modulating macrophage polarization; • Upregulation of Toll‐like receptor expression,inducing chronic inflammation.	[423, 424, 425, 426]
Respiratory	*Streptococcus* *Veillonella* *C. Pneumoniae* *Cyanobacteria* SCFA‐producing bacteria	NSCLC	• ERK/PI3K pathway activation promoting proliferation; • Immune‐suppressive microenvironment; • Metabolic metabolites inducing apoptosis; • Decreased CD36; • Increased PARP1; • Activate CD4^+^ T cells, inhibit CD8^+^ T cells and NK cells, and induce IFN‐γ‐R1 in NK cells.	[265, 427, 428, 429, 430, 431, 432]
Breast	*Proteobacteria* *Methylobacterium radiotolerans* HPV	BC	• TLR pathway dysregulation; • Induced DNA damage.	[433, 434, 435, 436]
Brain	*Fusobacterium* *Limosilactobacillus* *Lactobacillus*	Glioma	• *Fusobacterium nucleatum* promotes tumor proliferation; • Microbial metabolites regulating Neuronal genes.	[80]
Bone Marrow	SCFA‐producing bacteria *Klebsiella*	Multiple myeloma	• SCFAs inhibit NF‐κB‐mediated inflammation; • Nitrogen‐cycling microbiota promoting immune‐suppressive microenvironment.	[437]

Abbreviations: CC, cervical cancer; IGF‐1, insulin‐like growth factor 1; NSCLC, non‐small cell lung cancer; OSCC, oral squamous cell carcinoma; PARP1, poly [ADP‐ribose] polymerase 1; PI3K, phosphatidylin‐ositol‐3‐kinase; SCC, squamous cell carcinoma.

### Oral microbiome and oral cancer

Current evidence demonstrates a complex and mechanistic relationship between the oral microbiome composition and oral cancer pathogenesis. Dysbiosis of the oral microbiota represents a significant risk factor for oral cancer development, wherein the aberrant proliferation of specific bacterial communities promotes malignant transformation and progression through inflammatory response activation and dysregulation of critical cellular signaling pathways. Additionally, HPV infection is significantly associated with oral squamous cell carcinoma (OSCC) development, while specific patterns of oral fungal colonization strongly predict clinical outcomes in oral cancer patients. Metabolites produced by oral microorganisms significantly reshape the TME, thereby modulating cancer cell proliferation and metastatic potential and highlighting the microbiome's multifaceted regulatory role in oral carcinogenesis [438, 439].

#### Association of oral microbial imbalance with oral tumorigenesis

Numerous studies confirm the close association between oral microbiota dysbiosis and oral cancer development. Dysregulation of microbiome is now recognized as an important hallmark of carcinogenesis. The oral microbiota (including bacteria, fungi, archaea, and viruses) maintains ecological homeostasis through dynamic interactions with the host microenvironment, and its dysregulation can promote carcinogenesis by disrupting these host–microbiota interactions [440]. Pathogenic bacteria such as *P. gingivalis*, *F. nucleatum*, *C. albicans*, *Streptococcus anginosus*, and *Selenomonas spp*. show significant enrichment in cancerous tissues compared to healthy oral mucosa [413, 433, 441]. Further studies have demonstrated that the abundance of *Prevotella*, *Chlamydia*, and *Firmicutes* in the salivary microbiomes of OSCC patients differs significantly from that of healthy controls [442]. Evidence indicates that *C. albicans* demonstrates significant oncogenic potential in OSCC, contributing to tumor progression mainly through multiple mechanisms, including epithelial damage, production of oncogenic factors, and regulation of the TME [413]. Notably, oral microecological dysbiosis induced by betel nut chewing appears to accelerate carcinogenesis, with the abnormal proliferation of *F. nucleatum*, *P. gingivalis*, *Prevotella melaninogenica*, and other microbes in the saliva of cancer patients being mechanistically linked to their carcinogenic potential [414]. Collectively, these findings elucidate how oral microbial dysbiosis contributes to oral cancer development through diverse molecular mechanisms.

Several systematic reviews and meta‐analyses have investigated the relationship between the oral microbiome composition and oral cancer development. Han et al. observed significantly increased α and β diversity in the salivary microbiota of OSCC patients compared to healthy individuals [443]. The oral microbiota in OSCC patients was predominantly characterized by *Streptococcus*, *Lactobacillus*, and *Prevotella* [443]. A recent meta‐analysis summarized the characteristics of oral microbiome across different developmental stages of OSCC, revealing variations in microbial diversity, species, and abundance among different samples [444]. Another meta‐analysis indicated that oral microbiome composition is associated with cancer patient prognosis [445]. For instance, patients with lower oral diversity, increased *Fusobacterium* abundance, or *P. gingivalis* colonization were associated with compromised OS [445]. Additionally, established risk factors, including dietary patterns and tobacco exposure, significantly modulate microbiome composition and cancer progression. Cohort studies have demonstrated that increased carbohydrate intake is associated with increased oral bacterial diversity, enrichment of *Fusobacterium* and *Leptotrichia*, and depletion of *Actinomyces* operational taxonomy units (OTUs) [446]. Conversely, dietary patterns characterized by high sugar, processed foods, and alcohol accelerate oral carcinogenesis, whereas diets rich in fruits, vegetables, and fish demonstrate chemopreventive properties against cancer development [447]. In tobacco users, irrespective of conventional combustible cigarettes or electronic nicotine, significant alterations and abundance profiles in oral microbiome composition have been indicated compared to non‐smoking controls [448]. Previous research has indicated that approximately 74% of OSCC cases are attributed to tobacco exposure and chronic heavy alcohol consumption [449, 450]. Furthermore, in OSCC pathogenesis, tobacco‐derived carcinogens potentially interact with EBV infection [450].

The composition and functional characteristics of the oral microbiome are substantially influenced by modifiable factors, including dietary patterns and smoking behaviors, which subsequently modulate susceptibility to multiple malignancies. Zhang et al. indicate that inadequate oral hygiene practices, including irregular tooth brushing and non‐use of dental floss, facilitate dysbiotic microbial overgrowth, and significantly elevate oral carcinoma risk [451]. Another study has established associations between compromised oral hygiene status and elevated risk of NPC, with the oral microbe *Leptotrichia wadei* as a potential mediator connecting quantitative oral hygiene assessments with NPC pathogenesis [452]. Additionally, a tooth brushing frequency of once daily or less (compared with twice or more daily) is independently associated with a 1.8‐fold elevated risk of ESCC. Individuals with ESCC exhibit significantly reduced oral bacterial diversity compared to healthy individuals [453].

#### Mechanisms by specific bacteria promote oral cancer progression

Oral microbiota contributes to oral cancer development through multiple molecular pathways. Substantial evidence demonstrates that oral microbes, including *C. albicans* and *S. aureus*, are significantly associated with increased oral cancer risk. Their oncogenic mechanisms include disruption of oral microenvironment homeostasis and upregulation of critical oncogenes, such as *PI3KCA*, *hRAS*, *mTOR*, and *BRAF*, which can accelerate malignant transformation [40]. Comprehensive microbiome analyses have established that multiple oral microorganisms, including *P. gingivalis*, *F. nucleatum*, and *Streptococcus spp*., are causally linked to oral carcinogenesis through the induction of chronic inflammatory responses, regulation of cell proliferation, NF‐κB signaling pathway activation, and production of carcinogenic compounds [415, 454]. Microbial components induce β‐defensin 2 expression and promote the progression of precancerous lesions through stimulation of mast cell degranulation and establishment of persistent inflammatory responses, a process directly implicated in the pathogenesis of tongue squamous cell carcinoma [455]. Notably, specific oral microbes may diminish chemotherapeutic agent efficacy and exacerbate treatment‐related complications [456], suggesting the importance of microbial modulation in oncology treatment strategies. Collectively, these findings delineate a complex molecular network wherein oral microbiota drives oral carcinogenesis through interconnected mechanisms encompassing transcriptional regulation, inflammatory pathway activation, and comprehensive remodeling of the immune microenvironment.

#### HPV virus infection and the development of oral squamous cell carcinoma

HPV functions as a critical driver in OSCC progression. High‐risk HPV genotypes (particularly types 16 and 18) drive oncogenesis by interfering with the DDR system, consequently suppressing tumor suppressor gene function and inducing genomic instability [457, 458, 459, 460]. Additionally, viral components of the oral microbiome, principally HPV, contribute to oral mucosal dysbiosis, precancerous lesions, and OSCC progression through activation of inflammatory pathways and dysregulation of cancer stem cell‐associated signaling networks [416]. HPV interacts synergistically with diverse microorganisms, including EBV, herpes simplex viruses, HIV, and multiple bacterial species. This interaction promotes persistent viral infection, accelerating cancer development [457]. These mechanisms reveal that HPV both disrupts cellular regulatory processes through genetic material interference and promotes OSCC transformation through remodeling of the local microenvironment, thereby revealing novel therapeutic targets for improved clinical diagnosis and treatment strategies.

#### Relationship between fungal colonization and prognosis of oral cancer

Recent studies have confirmed significant correlations between patterns of fungal colonization within the oral ecosystem and clinical outcomes in oral cancer patients [461, 462]. Clinical investigations have demonstrated the presence of complex polymicrobial communities, including *Chlamydia* and diverse fungal taxa in salivary samples from OSCC patients [442]. As a representative pathogenic fungus, *C. albicans* promotes oral carcinogenesis and progression through multiple molecular mechanisms. Its cell wall components (especially β‐glucan) induce Th17 cell‐mediated immune responses and activate the NF‐κB and Wnt signaling pathways, consequently fostering a pro‐carcinogenic microenvironment formation and enhancing tumor cell resistance to apoptosis [417, 463, 464]. Additionally, *C. albicans* and yeast upregulate expression of pro‐inflammatory cytokines, including IL‐6 and IL‐8, thereby accelerating oral cancer progression [418]. *C. albicans* also upregulates PD‐L1 expression to facilitate tumor immune escape, while simultaneously accelerating HNSCC progression through modulation of acetaldehyde metabolism processes [465]. These findings suggest that alterations in fungal community composition may serve as valuable diagnostic and prognostic biomarkers for oral malignancies.

#### Impact of oral microbial metabolites on the oral cancer microenvironment

Metabolites derived from oral microorganisms orchestrate the dynamic evolution of the oral cancer microenvironment through complex and interconnected signaling networks. Disruption of oral microbial communities induces aberrant metabolite release, compromises epithelial barrier integrity, and precipitates immune homeostasis dysregulation. These pathological alterations synergistically converge on oral mucosal cells, promoting malignant transformation and dysregulated cellular proliferation [466]. Interactions between microbial metabolites and the host immune system constitute a fundamental driver of tumor progression. Specifically, lactate metabolites attenuate anti‐tumor immune responses through recruitment of TAMs, Tregs, and myeloid‐derived suppressor cells [467]. Among microbial metabolites, SCFAs are closely associated with OSCC development. Particularly, NaB produced by *P. gingivalis* promotes OSCC progression through mechanisms that remain incompletely characterized despite intensive investigation [468, 469]. Microbial–host interactions exhibit mutually reinforcing relationships. Oral microorganisms and their metabolites promote tumor progression, while the TME creates favorable conditions for microbial colonization. This mutually reinforcing vicious cycle constitutes an important pathogenic mechanism in oral cancer progression [470].

### Skin microbiome and skin cancer

The skin microbiome serves as a critical regulator of cutaneous homeostasis, and disruption of this dynamic balance is strongly associated with skin carcinogenesis and progression [471]. Accumulating evidence has demonstrated that skin microbiome dysregulation promotes cutaneous carcinogenesis through multiple mechanisms, including disruption of epidermal barrier integrity, induction of immune escape, and activation of inflammatory cascade responses. UV radiation, a major causative factor in skin carcinogenesis [472, 473, 474], not only directly damages host cells but also increases the risk of photocarcinogenesis by altering the composition and function of skin microbial communities. These multidimensional interaction mechanisms highlight the central regulatory role of the skin microbiome in cancer prevention, occurrence, and treatment, establishing a theoretical foundation for the development of microbiome‐targeted preventive and therapeutic strategies.

#### Relationship between skin microbial imbalance and skin carcinogenesis

Dysbiosis of the skin microbial community contributes to cutaneous carcinogenesis through both direct oncogenic or oncostatic effects and indirect immunometabolic regulatory mechanisms. Among carcinogenesis‐associated microbes, *S. aureus* induces carcinogenesis through multiple virulence factors and cytokine release, promoting SCC cells proliferation [419]. This pathogen shows a significantly elevated relative abundance in SCC patients [475]. *Fusobacterium spp*., *Trueperella spp*., and *Corynebacterium* are significantly enriched in melanoma lesions [476, 477]. Notably, *Corynebacterium* demonstrates a significant positive correlation with IL‐17 levels, a critical factor in melanoma progression [477]. Mendelian randomization analysis has revealed a bidirectional positive correlation between class *Bacilli* and malignant melanoma, whereas decreased phylum *Bacteroidetes* abundance may elevate disease risk [478]. *Pseudomonas aeruginosa* also promotes skin cancer cell growth [479]. Among protective microorganisms, *Staphylococcus epidermidis* (*S. epidermidis*) significantly reduces UV‐induced skin cancer incidence by inhibiting tumor cell DNA synthesis through the secretion of 6‐N‐hydroxyaminopurine (6‐HAP) [420]. Similarly, *Cutibacterium acnes* contributes to anti‐tumor processes through anti‐angiogenic effects, and its reduced abundance correlates strongly with SCC disease progression [480]. These findings suggest that the skin microbiota bidirectionally regulates skin cancer progression by directly affecting cell proliferation and indirectly regulating immune metabolism [481], providing new therapeutic targets for microbial intervention‐based skin cancer prevention and treatment strategies.

#### Relationship between microbiome alterations due to ultraviolet exposure and skin cancer

Ultraviolet exposure promotes both the initiation and progression of skin cancer. Beyond directly damaging host cells, ultraviolet radiation can also kill skin microorganisms and induce dysbiosis of the cutaneous microbial community, thereby synergistically promoting skin cancer initiation and progression [482, 483, 484, 485]. UV radiation (especially the UVC band) exerts broad‐spectrum bactericidal effects by disrupting microbial nucleic acid structure and enzymatic activity [421, 486], resulting in significant alterations to skin microbiome composition. UV irradiation increases the abundance of *Proteobacteria*, impairing anti‐inflammatory immune response functions. Similarly, it decreases *Lactobacillaceae* and *Pseudomonadaceae*, compromising cutaneous barrier homeostasis. Abnormal *Cyanobacteria* proliferation may contribute to tumorigenesis, progression, and metastasis through the release of pro‐inflammatory metabolites and upregulation of immunosuppressive factors [487, 488]. This microbial dysbiosis synergizes with UV‐induced keratinocyte DNA damage, and the dysregulated microbiota exacerbates oxidative stress and chronic inflammatory states, further destabilizing genomic integrity and accelerating non‐melanoma skin cancer (NMSC) progression [489]. Additionally, UV radiation and HPV infection show significant synergistic effects in CSCC, with UV‐induced damage interacting with HPV oncoproteins through activation of the Wnt/β‐catenin signaling pathway, collectively driving malignant cellular transformation [490]. These mechanisms indicate that UV radiation not only directly damages host cells but also multidimensionally promotes cutaneous carcinogenesis through remodeling the microbe–host interaction network.

#### Viral infections and the mechanisms of skin cancer development

Viral infections represent critical etiological factors in the pathogenesis and progression of cutaneous malignancies. Studies show β‐HPV exhibits significant tropism for cutaneous tissues and is strongly associated with non‐melanoma skin cancer development, whereas HPVs of γ, μ, and v genera mainly induce the formation of benign cutaneous lesions [491]. In states of immunosuppression (including verrucous epidermal dysplasia, HIV infection, or long‐term immunosuppressive drug use), β‐HPV significantly accelerates cutaneous oncogenesis [422, 492]. Additionally, KSHV has been established as a direct etiological agent in Kaposi's sarcoma (KS) development, evidenced by the persistence of specific viral DNA sequences within KS lesions and the temporal precedence of KSHV infection before tumor formation, further corroborating its pathogenic mechanism [493]. In studies of MCC, Merkel cell polyomavirus (MCV) DNA is detectable in approximately 80% of tumor tissues, suggesting viral genome integration functions as a potential driver of clonal tumor expansion [494]. Together, this body of evidence underscores the pivotal role of oncogenic viruses in cutaneous carcinogenesis through multiple molecular mechanisms, including viral genome integration and host immune evasion.

#### Microbe‐mediated inflammatory response promotes skin cancer progression

The skin microbiota drives the cancer process by activating inflammatory signaling pathways. Pathogenic microbes can abnormally activate Toll‐like receptors (TLRs), triggering chronic inflammatory responses that promote skin cancer development [495, 496]. Animal experiments show that TLR‐5 signaling pathway activation, through chronic inflammatory responses, plays an important role in regulating tumorigenesis in murine skin cancer models [497]. Additionally, *S. aureus* releases various pro‐inflammatory factors that promote tumor development‐conducive immune microenvironment formation and induce SCC progression [419]. Conversely, *S. epidermidis* activates immune‐inflammatory responses while reducing pathogenic bacteria colonization (such as *S. aureus*) through competitive inhibition, thereby inhibiting cancer cell proliferation [498]. Aberrations in skin microbiota metabolites also influence tumor progression, for example, reduced levels of the tryptophan metabolite I3A accelerate the malignant transformation of precancerous lesions [499]. These mechanisms suggest that microbe‐mediated inflammatory networks participate in skin tumorigenesis and evolution through multiple signaling pathways.

#### Impact of the microbiome on melanoma immunotherapy

ICI therapy has markedly improved prognosis in melanoma patients; however, substantial heterogeneity exists in individual treatment responses [500, 501, 502]. Several studies have confirmed that the microbiome plays a pivotal role in modulating ICI therapeutic efficacy [503, 504]. A clinical investigation found that patients receiving antibiotics during cancer treatment, including those with melanoma, exhibited significantly reduced response rates to ICI therapy [505]. Cutaneous commensal bacteria also show immunomodulatory potential. In a murine model, genetically engineered *S. epidermidis* induced robust T‐cell immune responses through expression of melanoma‐specific antigens and synergistically enhanced ICI efficacy, resulting in potentiated anti‐tumor effects [506]. Notably, synergistic effects between the microbiome and immunotherapy can also be achieved through alternative strategies, such as the combination of oncolytic viruses (OVs) and ICIs. OVs induce both local and systemic immune responses by selectively lysing tumor cells and facilitating antigen release, while the combination of anti‐CTLA‐4 and anti‐PD‐1 therapy significantly inhibits primary tumor progression and simultaneously promotes regression of metastatic lesions [507]. Collectively, these findings suggest that the skin microbiome not only directly modulates host immune status but may potentially remodel the TME through synergistic effects with other therapeutic modalities, thereby providing a multifaceted scientific rationale for optimizing melanoma immunotherapeutic strategies [508, 509, 510, 511, 512].

### Urinary/genital microbiome and urinary tumors

The genitourinary microbiome exhibits complex interactions with urogenital tumorigenesis and progression [513, 514, 515]. Studies reveal that dysbiosis of the urogenital microbiota represents a significant risk factor for bladder carcinogenesis, while HPV infection constitutes a key causative factor in CC development and progression. Additionally, numerous studies show that specific bacterial infections are significantly associated with PCa risk, and the composition and functional profile of the urinary microbiome can markedly influence patients' clinical responses to anti‐tumor therapies. With advancing research, microbiome‐derived biomarkers are emerging as important molecular tools for the early diagnosis and prognostic assessment of urological malignancies.

#### Association of urethral microbial imbalance with bladder cancer

Recent studies have progressively elucidated the association between urethral microbial imbalance and BCa through microbial characterization. One study showed microbial richness (α‐diversity) was significantly lower in BCa patients compared with healthy controls, suggesting that dysbiosis may contribute to disease progression [516]. Additionally, urinary microbiome β‐diversity in BCa patients differed significantly from that of the recurrence‐free tumor group, with elevated abundance of *Veillonella* and *Bifidobacterium* in the tumor group, whereas the recurrence‐free group exhibited enrichment of *Escherichia‐Shigella* and *Helococcus* [423]. Further studies identified significant correlations between bladder carcinogenesis and various microbial taxa, including members of the family *Rikenellaceae* and the genera *Allisonella* and *Senegalimassilia* [517]. Microbial compositional analysis between muscle‐invasive bladder cancer (MIBC) and non‐muscle‐invasive bladder cancer (NMIBC) revealed that there were no significant changes in the relative abundance of major phyla such as *Firmicutes* and *Proteobacteria*. However, *Flavobacteriales*, *Eubacterium CAG‐581*, and *Bacteroides sp. 43‐47FAA* were enriched in NMIBC and were strongly associated with decreased patient survival [518]. In summary, structural dysbiosis of the urogenital microbiome may promote BCa development through regulation of the local microenvironment, and these characteristic microbial signatures may serve as potential biomarkers, providing new avenues for BCa diagnosis and prognostic assessment.

#### Association of bacterial infections with prostate cancer risk

A substantial body of evidence confirms a significant association between bacterial infections and PCa development. Several studies show that the prostatic microbiome of PCa patients displays characteristic alterations, with significant differences in the relative abundance of specific bacterial taxa. In a case‐control study of patients with benign and malignant prostate lesions, researchers found significant alterations in bacterial communities within high‐grade PCa tissues, with notable enrichment of species including *Streptococcus anginosus*, *Anaerococcus lactolyticus*, and *Actinobaculum schaalii* [519]. SCFAs produced by intestinal microbial communities can induce chronic inflammatory responses through multiple mechanisms: modulation of the IGF‐1 signaling pathway, promotion of M2‐phenotype macrophage polarization, and upregulation of TLR expression, collectively creating a microenvironment conducive to PCa progression [424]. In summary, urinary tract bacterial infections promote PCa development through modulation of key signaling pathways and induction of chronic inflammatory responses that alter the TME.

#### Mechanisms of HPV infection and the development of cervical cancer

The vast majority of CC cases are attributable to persistent HPV infection [520, 521, 522]. The mechanisms underlying HPV infection and subsequent CC development encompass multifaceted molecular and pathological processes, including effects on the cervicovaginal microbiota, which represents an emerging frontier in gynecologic oncology research. During the progression of cervical lesions from premalignant to malignant states, HPV infection significantly alters the composition and abundance of vaginal microbial communities. Specifically, commensal bacteria such as *Lactobacillus crispatus* and *Lactobacillus iners* exhibit significant decreases in abundance, whereas potentially pathogenic bacteria including *Prevotella* and *Gardnerella* demonstrate significant proliferation [425, 523, 524, 525, 526]. Other viruses (such as HIV, EBV, and herpes simplex virus) are also closely associated with HPV infection, collectively promoting malignant transformation and tumor progression [527]. Importantly, increased cervicovaginal microbiome (CVM) diversity, together with decreased *Lactobacillus spp*., is significantly associated with elevated risk of tumorigenic intraepithelial lesions resulting from persistent HPV infection, ultimately accelerating progression to invasive carcinoma [528]. Notably, HPV infection not only alters the compositional structure of the vaginal microbiota but also collaboratively establishes a tumor‐promoting microenvironment through complex interactions with microbial metabolic activities [426]. Additionally, HPV maintains its infection status through dual mechanisms: inducing dysbiosis of the CVM and simultaneously suppressing the expression of host antimicrobial peptides essential for innate immunity, further exacerbating vaginal microecological imbalance. HPV‐associated perturbations in the cervicovaginal microenvironment exhibit distinctive metabolomic signatures and inflammatory responses that correlate with the extent of HPV infection, collectively facilitating tumorigenesis and disease progression [426].

#### Impact of the urinary microbiome on response to treatment

Distinct compositions of the urinary tract microbiome exhibit significant heterogeneity in their responses to therapeutic interventions. A growing body of evidence confirms that the microbiota plays a pivotal role in renal cell carcinoma (RCC) pathogenesis, progression, and therapeutic responsiveness, with the underlying mechanism mainly realized through immune system function modulation, host metabolism, and drug response [529]. Microbiome diversity is significantly reduced in RCC tissue specimens compared to adjacent non‐neoplastic renal parenchyma. The end‐stage renal disease‐associated microbiome demonstrates decreased abundance of putatively beneficial bacteria (such as *Roseburia*) and concurrent enrichment of potentially oncogenic microbes (such as *Escherichia*, *Fusobacterium*, and *Bacteroides*) compared to control specimens [530]. Together, these findings underscore the potential contributory role of urinary microbiome alterations in renal disease pathogenesis, thereby providing a critical theoretical foundation for the development of novel microbiome‐targeted therapeutic strategies in RCC management.

#### Microbial markers in predicting prognosis of urologic tumors

Microbiome‐derived biomarkers demonstrate significant clinical applications in urologic tumor diagnosis. Numerous studies confirm dynamic alterations in specific microbial communities exhibit significant correlations with PCa development and prognostic assessment. The β‐diversity of circulating fungal microbiomes in peripheral blood significantly differs between PCa patients and healthy controls, with particularly pronounced distinctions in patients with high pathological tumor stages (pT3 or pT4). Notably, the abundance of *Sordariomycetes* is significantly elevated in advanced PCa and has emerged as a promising biomarker for identifying high‐grade disease [222]. Additionally, in post‐digital rectal examination urine samples and post‐prostatectomy prostatic secretions, alterations in the abundance of five strictly anaerobic bacterial genera (including *Fenollaria*) show significant correlation with PCa aggressiveness and biochemical recurrence risk, suggesting their potential utility as non‐invasive urinary diagnostic and prognostic biomarkers [531]. These findings suggest that specific microbiome markers may not only participate in urologic tumor pathogenesis but also serve as potential diagnostic tools, providing new approaches for early detection, risk stratification, and therapeutic monitoring of urologic malignancies.

### Respiratory microbiome and lung cancer

A complex bidirectional relationship exists between the respiratory microbiome and lung cancer development [532, 533, 534]. Emerging evidence demonstrates that respiratory microbiome dysbiosis is significantly associated with lung cancer development, involving molecular mechanisms that may contribute to lung cancer progression through interconnected signaling pathways, revealing the critical role of microbial community alterations in lung cancer pathogenesis. Additionally, the respiratory microbiome significantly modulates key lung cancer biological behaviors, including proliferation, invasion, and metastasis, through regulation of the TIME. Notably, respiratory microbiome composition and temporal dynamics correlate strongly with treatment responses and clinical outcomes in lung cancer patients, offering promising avenues for the development of microbiome‐based precision medicine strategies.

#### Association of respiratory microbial imbalance with lung carcinogenesis

Substantial evidence now establishes that respiratory microbiota dysbiosis is causally linked to lung cancer pathogenesis. Clinical investigations confirm that the respiratory microbiome composition and community structure in lung cancer patients differ significantly from those of healthy individuals. Lower respiratory microbial α‐diversity is slightly reduced in lung cancer patients [427, 535]. In patients with central lung tumors, *Streptococcus* constitutes the predominant bronchial microbes at both the lesion site and contralateral bronchus, whereas *Pseudomonas* predominates in the bronchial microbiome of healthy controls [428]. Significant taxonomic overlap exists between the oral and pulmonary microbiomes of lung cancer patients, whose salivary samples demonstrate significantly altered bacterial abundance (*Streptococcus*, *Lactobacillus*, etc.) and fungal composition (*Candida*, *Malassezia*, etc.) compared to control individuals [428]. Distinct lung cancer histological subtypes exhibit characteristic microbial signatures: lung adenocarcinoma (LUAD) demonstrates predominance of *Propionibacterium*, whereas lung squamous cell carcinoma (LUSC) shows enrichment of *Enterobacteriaceae*, with significantly higher microbial diversity observed in LUSC patients [313]. In early‐stage I/II NSCLC patients, *Firmicutes*, *Bacillus*, and *Actinobacteria* demonstrate significant enrichment, whereas *Actinobacteria* predominates in patients with advanced‐stage (III/IV) disease [313]. Respiratory microbiota dysbiosis drives lung cancer progression through multiple interconnected mechanisms, including metabolic reprogramming, inflammatory cascade modulation, and establishment of an immunosuppressive TME. For example, elevated *Veillonella parvula* (*V. parvula*) abundance correlates with reduced CD4^+^ T cell infiltration and upregulation of PD‐1 and IL‐17 expression, collectively fostering an immunosuppressive TME [429]. These findings suggest respiratory microbial dysbiosis functions not only as a critical driver of lung carcinogenesis but also provides novel research directions for developing innovative diagnostic biomarkers and therapeutic interventions.

#### Molecular mechanisms underlying the association between specific bacteria and lung cancer progression

Current evidence elucidates distinct molecular mechanisms connecting specific bacterial taxa to lung cancer initiation and progression. Specific bacterial genera, including *Veillonella* and *Streptococcus*, modulate lung cancer cell proliferation and survival through activation of key oncogenic pathways, particularly ERK and PI3K, thereby contributing to lung cancer development and progression [536]. Studies confirm specific bacterial groups significantly correlate with distinct lung cancer histopathological subtypes, with *Cyanobacteria* exhibiting significant enrichment in LUAD, which correlates with decreased CD36 expression and elevated poly[ADP‐ribose] polymerase 1 (PARP1) levels. These inflammatory mediators are mechanistically linked to carcinogenic processes and tumor‐promoting effects [430]. LUSC demonstrates significant associations with distinct bacterial families, particularly *Enterobacter* and *Serratia* [537, 538]. These specific bacterial taxa contribute to tumorigenesis and malignant progression through modulation of multiple cancer‐associated signaling networks. Interestingly, microbial diversity within lung cancer lesions exhibits modest reduction compared to adjacent normal lung parenchyma, while specific bacterial taxa associated with SCFA production demonstrate significant predictive value for lung cancer risk and progression [427, 538]. SCFAs (particularly NaB) modulate immune cell function and immunoregulatory molecules, including activation of CD4^+^ T cells, Tregs, and Th2 cells; inhibition of CD8^+^ T cells and NK cells, and induction of IFN‐γ‐R1 in NK cells, thereby suppressing lung cancer development and progression [265]. Comprehensive analyses have identified several bacterial taxa, including *Actinomycetota*, *Bacteroidota*, and *Cyanobacteria*, that are significantly associated with lung cancer progression, while respiratory microbiome dysbiosis contributes to pulmonary tumorigenesis through multiple interconnected mechanisms, including metabolic network perturbation, inflammatory pathway modulation, and immune response alteration [535].

#### Microbiome regulation of the immune microenvironment in lung cancer

The mechanisms by which the microbiome regulates the lung cancer immune microenvironment exhibit remarkable complexity and diversity, with profound implications for tumorigenesis, progression, and therapeutic response [539]. Existing studies show oral microorganisms and their metabolites (such as proteins and endotoxins) translocate to pulmonary tissues through either direct respiratory tract inhalation or hematogenous dissemination. Subsequently, they influence lung cancer initiation and progression through multiple mechanisms, including chronic inflammatory response induction, host immune system reprogramming, and activation of oncogenic signaling pathways [431]. Specifically, oral *H. pylori* can translocate across lung endothelium, establishing persistent inflammatory microenvironments that ultimately promote malignant transformation and tumor growth through modulation of DC function and suppression of CD8^+^ T cell cytotoxicity [431]. Additionally, dysregulation of oral microbiota can modulate lung cancer cell survival through interference with p53‐dependent apoptotic pathways [540]. Pulmonary microorganisms contribute to lung cancer development and progression through multiple integrated pathways, including directly affecting local immune microenvironment, modulating tumor‐associated signaling pathways promoting cell cycle dysregulation and genomic instability, and bacterial metabolite‐mediated effects involving bacteriocin production, TLR signaling activation, and TNF release [541]. Recent studies identified *Chlamydia pneumoniae* infection and HPV types 16 and 18 infection as potential lung cancer‐associated risk factors that contribute to disease progression through profound remodeling of the local immune microenvironment [432]. In summary, the microbiome plays pivotal roles in regulating the TIME regulation of lung cancer, offering novel insights and potential therapeutic targets for comprehensive lung cancer prevention, early detection, and personalized treatment approaches.

#### Relationship between respiratory microbiome and prognosis of lung cancer treatment

Studies show respiratory microbiome composition and diversity are strongly associated with therapeutic outcomes in patients with lung cancer. A comprehensive study of never‐smoking female LUAD patients revealed that the relative abundances of *Faecalibacterium*, *Fusicatenibacter*, and *Bacteroides* correlate with tumor size, and *Fusicatenibacter* relative abundances also correlate with tumor stage, suggesting that specific microbial signatures may play pivotal roles in lung cancer progression and treatment response [542]. Additionally, alterations in the abundance of butyrate‐producing bacteria in NSCLC patients have garnered significant scientific attention [543]. These commensal microorganisms may favorably influence lung cancer treatment outcomes through multiple mechanisms, including inhibition of pathogenic bacterial growth, enhancement of nutrient absorption, modulation of immune responses, and maintenance of intestinal barrier integrity [543]. Recent investigations confirm that physiological concentrations of SCFAs can significantly inhibit lung cancer cell proliferation by inducing cell cycle arrest and promoting apoptosis, further substantiating the potential modulatory role of the gut–lung axis in lung cancer treatment efficacy [544, 545].

### Other organ microbiomes and tumors

Microbial communities in other organs also demonstrate significant associations with tumorigenesis and progression in their respective tissues. Emerging evidence indicates that dysbiosis of the breast microbiome significantly correlates with BC initiation and progression; similarly, aberrant alterations in the brain microbiome increase brain tumor (BT) risk and promote disease progression, while interactions between the bone marrow microbiome and hematological malignancies are increasingly recognized as an important research focus.

#### Breast microbiome and breast cancer

Recent studies have demonstrated a significant association between breast microbiome and BC development [546, 547]. Breast tissue harbors a distinct microbial ecosystem with characteristic taxonomic distribution patterns, predominantly dominated by members of the *Proteobacteria* and *Firmicutes* [434]. Comparative metagenomic analyses revealed significant differences in breast tissue microbial composition and functional profiles between healthy individuals and BC patients [434]. Quantitative microbiome profiling has demonstrated an overall elevation in microbial biomass within BC tissues, concurrent with a significantly reduced taxonomic diversity index compared to adjacent normal tissues [548]. In BC patients, total bacterial load is significantly elevated, with characteristic enrichment of specific microbial species such as *Methylobacterium radiotolerans*, while the relative abundance of *Sphingomonas yanoikuyae* is significantly depleted compared to non‐malignant tissues [435]. Additionally, the TLR signaling pathway is significantly dysregulated in BC tissues, a phenomenon closely associated with microbial pattern recognition and immune response modulation [433]. At the molecular mechanism level, breast microbiome may contribute to mammary carcinogenesis. Key biological pathways implicated in this process include DNA damage mediated by toxins from *Escherichia coli* (*E. coli*) and other bacteria, and cancer susceptibility mediated by HPV [434]. Furthermore, the composition and functional status of the breast microbiome significantly influence BC treatment outcomes. For example, *Lactobacillus iners* is significantly associated with poor patient prognosis and may lead to decreased chemotherapy and radiotherapy sensitivity [436]. In summary, breast microbiome plays complex and important regulatory roles in BC occurrence, development, and therapeutic response. However, the precise molecular mechanisms underlying these interactions and their potential translational applications in clinical oncology warrant further comprehensive investigation [549, 550, 551, 552].

#### Brain microbiome and brain tumor

Research exploring the association between BTs and the microbiome has faced considerable challenges due to the blood–brain barrier's (BBB) intrinsic function in preventing direct microbial invasion into the central nervous system. However, recent studies have revealed the critical role of specific microbiomes in modulating the brain TME. For example, comprehensive metagenomic analyses have demonstrated significant differences in microbial composition between glioma tissues and adjacent non‐neoplastic brain parenchyma, with the phyla *Firmicutes* and *Fusobacteria* exhibiting significantly higher abundance in gliomas. In‐depth analysis showed that genera including *Fusobacterium*, *Limosilactobacillus*, and *Pasteurella* are significantly enriched within glioma tissues [80, 553]. Notably, integrated multi‐omics analyses have indicated that the intratumoral microbiome may regulate neuron‐related gene expression networks through bacterial‐derived metabolites. Both *in vivo* and *in vitro* experimental models have confirmed that key bacterial species enriched in gliomas influence tumor growth. *F. nucleatum* significantly promotes tumor cell proliferation [80]. These findings suggest that although microorganisms rarely directly penetrate the intact BBB, resident microbial communities within the brain TME may significantly influence tumor progression through metabolite production or immune regulation, thereby providing novel direction for exploring the complex “microbiome–tumor” interaction mechanisms.

The gut–brain axis represents a bidirectional communication network through which the gut microbiota and central nervous system reciprocally regulate each other, influencing the progression of neurological malignancies and gastrointestinal neoplasms [554]. Research using a neurofibromatosis type 1‐associated low‐grade glioma mouse model has elucidated the molecular mechanisms by which intestinal *Bacteroides* species regulate optic pathway glioma progression through the TGF‐β signaling pathway within the gut–brain–tumor axis, suggesting that targeted manipulation of gut microbiota could represent a novel therapeutic strategy for neurological malignancies [555]. Through integrated multi‐omics analysis and experimental validation in mouse models, Lin et al. have identified a gut–brain–tumor axis regulatory network involving enrichment of *Clostridium* species and consequent purine metabolism dysregulation, which collectively mediate target organ redox imbalance. These molecular alterations synergistically contribute to disease pathogenesis, potentially mediating the initiation and progression of craniopharyngioma‐associated hypothalamic complications [556]. The loss of intestinal epithelial interleukin‐17 receptor A (IL17RA) signaling induced by aberrant expansion of Th17 cells can influence brain tumor growth by regulating the gut microbial community ecology [557]. Additionally, the microbiome may participate in regulating immune responses, modulating metabolite secretion, and influencing therapeutic efficacy to affect tumor development [558].

#### Bone marrow microbiome and hematologic neoplasms

The bone marrow microbiome has emerged as a critical factor influencing the initiation, development, and progression of hematologic malignancies [559, 560, 561]. Numerous investigations have confirmed that the microbiota significantly influences the bone marrow microenvironment through the synthesis of various bioactive metabolites. SCFAs exert bidirectional immunomodulatory effects within the bone marrow niche by inhibiting the expression of pro‐inflammatory factors, including NF‐κB, IL‐6, and TNF‐α, while concurrently promoting anti‐inflammatory factor IL‐10 production and enhancing Th17 and Th1 cell activities [562, 563, 564]. Clinical studies have revealed a significant decrease in the abundance of SCFA‐producing bacterial taxa in multiple myeloma (MM) patients, concurrent with significant proliferation of bacterial groups involved in the nitrogen cycle, such as *Klebsiella* [437]. These findings reveal that microbiome affects hematologic malignancies by modulating immune microenvironments and altering metabolic profiles, thereby providing potential targets for related disease treatment.

## MICROBIOME IN TUMOR DIAGNOSIS

### Microbiome markers

Microbiome markers have demonstrated significant clinical utility in tumor diagnostics. Comprehensive studies have established that microbial signatures can serve as robust diagnostic bioindicators, encompassing (including specific microbes, diversity indicators, and metabolites), viral markers (including infection status, viral load quantification, and viral oncoproteins), and fungal markers (including mycobiome community structures and fungal‐derived metabolites) [565, 566, 567, 568, 569]. Among these, bacterial marker research has been more extensively characterized, with compelling examples including *F. nucleatum* serving as a specific bacterial marker in CRC, GC, and oral cancer; gut microbiota diversity indices and SCFA metabolic profiles showing value for early detection, risk stratification, and prognostic assessment in CRC. Specific viral biomarkers demonstrate exceptionally high diagnostic value for particular malignancies, exemplified by high‐risk HPV for CC, HBV for liver cancer, and EBV for NPC. Additionally, liquid biopsy techniques show advantages for non‐invasive diagnosis in liver and PCs, particularly suitable for early screening and therapeutic monitoring. These microbiome‐derived biomarkers offer multifaceted clinical applications, including population‐based early screening, minimal residual disease detection, enhancement of conventional diagnostic accuracy, and provision of novel strategies for precision oncology diagnostics. Table 6 summarizes recent progress and the clinical application value of microbiome biomarker research in different tumor types.

**Table 6:** Advances and clinical applications of microbiomebiomarkers in tumor diagnosis.

Tumor type	Biomarker category	Representative biomarkers	Related research	Clinical utility	References
CRC	Bacteria & Metabolites	*Fusobacterium nucleatum* *Bacteroides fragilis* γ‐aminobutyric acid L‐aspartic acid phenylacetic acid	Enrichment of *Fusobacterium nucleatum* in tumor tissue correlates positively with tumor stage.	• Non‐invasive fecal screening (sensitivity 85%); • Recurrence monitoring.	[161, 570, 571]
	Viruses	*Bacteroides phage* *Streptococcus phage* Biomarker group containing 14 viruses	Viral biomarker panel distinguishes CRC from healthy controls (AUC 0.89).	Differentiation of adenoma vs. carcinoma (specificity 79%).	[572, 573]
	Fungi	*Rhodotorula dairenensis* *Cutaneotrichosporon curvatus*	Reduced fungal diversity in CRC, enrichment of specific taxa.	TME profiling: predicting immunotherapy response.	[574]
GC	Bacteria	*Fusobacterium* *Streptococcus*	*Streptococcus* ↑ *Lactobacillus* ↑	Non‐invasive salivary screening (AUC 0.76).	[575, 576]
	Fungi	*Candida albicans Malassezia globosa*	C. albicans promotes nitrosamine synthesis, correlates with GC risk.	Tissue biopsy support: risk stratification.	[43, 214]
HCC	Viruses	HBV viral load miR‐122	The viral load of HBV is negatively correlated with mi‐R122.	Stratified monitoring in hepatitis patients: antiviral efficacy assessment.	[577]
CC	Viruses	HPV‐16/18 ctDNA	HPV‐ctDNA positivity predicts recurrence risk.	Minimal residual disease monitoring:survival prognosis.	[578]
PC	Bacterial metabolites	Polyamine metabolites (spermine, spermidine)	Elevated serum polyamine levels precede imaging abnormalities.	High‐risk population screening: improved diagnosis with CA19‐9.	[579]
BC	Bacteria	Total bacterial load in tumor tissue	Load inversely correlates with tumor stage.	Prognostic assessment (low load indicates advanced risk).	[580]
NPC	Viruses	EBV antibodies	Combined detection AUC 0.93 (vs. healthy controls).	Creening in high‐risk regions.	[581]
PCa	Fungi	*Sordariomycetes*	Increased in plasma abundance (*p* < 0.01).	Non‐invasive diagnosis of advanced PCa; Biochemical recurrence prediction.	[222]

Abbreviations: AUC, area under curve; CA19‐9, carbohydrate antigen 19‐9; ctDNA, circulating tumor DNA; LPS, lipopolysaccharide.

#### Bacterial markers

Specific microbes: The human microbiome has demonstrated significant value in cancer diagnostics, with the biomarker potential of specific microbial taxa attracting substantial research interest [582, 583, 584, 585, 586, 587]. Numerous studies have confirmed that specific microbiota, when utilized as tumor diagnostic biomarkers, has significant clinical utility. In colorectal neoplasia studies, comparative analyses between patients with colorectal polyps and healthy controls have revealed significant differences in both salivary and fecal microbiota composition and diversity, characterized predominantly by increased abundance of potentially pathogenic bacteria and concurrent decreased representation of beneficial microbial taxa [588]. Additionally, intestinal microorganisms like *Veillonella*, *Bifidobacterium dentium*, and *Lactobacillus salivarius* have demonstrated good diagnostic value for GC [143]. *F. nucleatum* has emerged as a promising biomarker for CRC [161]. In familial adenomatous polyposis studies, researchers found intratumoral *E. coli*, as a precancerous lesion, could serve as an early microbial biomarker for CRC risk detection [589]. Additionally, peripheral blood CRC‐associated microorganisms, such as *B. fragilis* and *S. gallolyticus*, have shown bacterial marker potential in CRC prediction [570]. In GC diagnostics, specific microbial signatures, including *Fusobacterium*, *Streptococcus*, and *Pseudomonas*, have been identified as important discriminatory indicators differentiating malignant from non‐malignant conditions [575]. In BC research, investigators have observed a significant inverse correlation between tumor tissue total bacterial load and tumor stage, providing novel insights into microbiome‐based approaches for BC diagnosis [580]. These collective findings confirm the significant potential of microbiome‐based biomarkers in cancer screening and diagnosis. In conclusion, the human microbiome demonstrates extensive clinical potential for oncological diagnostics, with microbial‐based biomarkers representing a promising frontier that warrants further mechanistic investigation.

Indicators of bacterial diversity: As microbiome research has advanced, bacterial diversity metrics have emerged as particularly promising diagnostic indicators with significant clinical potential across multiple cancer types. Extensive investigations have confirmed that quantitative assessments of microbial abundance and community compositional characteristics provide innovative approaches for cancer detection and classification [588]. Different cancer types present unique bacterial compositional features. Tissue‐associated microbiome across various cancer types has demonstrated prognostic value for predicting disease recurrence, with a specific combination of nine bacterial genera significantly enhancing the accuracy of patient survival prediction [590]. Gut microbiota characterization has evolved as an effective non‐invasive assessment methodology for evaluating HCC risk. Through microbial sequencing technology, investigators have successfully identified optimal combinations of microbial markers demonstrating significant diagnostic potential for both early‐stage and advanced HCC. Concurrently, blood microbiome profiling‐based diagnostic models have achieved high accuracy in distinguishing HCC from healthy controls [591, 592]. In a comparative study, bacterial diversity was determined through OTUs analysis and represented by Shannon index, Simpson index, and Invsimpson index [591]. This study demonstrated that all three indices of fecal microbial diversity were significantly reduced in cirrhosis patients compared to the control group. Conversely, compared to cirrhosis, all three indices were significantly elevated, suggesting a characteristic microbial diversity pattern associated with hepatocarcinogenesis. The study concluded that indices based on OTU markers possess good diagnostic value [591]. A cross‐sectional study indicated that α‐diversity of circulating microbiota was significantly reduced in HCC patients. At the genus level, seven bacterial taxa demonstrated significantly differential abundance between HCC and control subjects. Among these, a diagnostic index composed of five genus‐level microbial signatures could characteristically distinguish HCC [592]. Additionally, gut microbiota‐based prediction models incorporating 37 specific strains have accurately identified advanced fibrosis status in non‐alcoholic steatohepatitis (NASH) patients, further substantiating the important clinical application of microbial signatures in early disease detection [593]. The diagnostic potential of microbial diversity metrics has also been extensively demonstrated in oral microbiome studies. Studies confirm characteristic alterations in tongue microbiota are significantly associated with GC development, potentially becoming novel non‐invasive biomarkers [576]. This study represented microbial α‐ and β‐diversity using multiple indices, finding that GC patients exhibited significantly increased tongue coating bacterial richness metrics concurrent with decreased overall bacterial diversity indices compared to non‐cancer controls. Specifically, GC patients demonstrated significantly reduced relative abundance of *Bacteroidetes*, *Fusobacteria*, *Proteobacteria*, and *Actinobacteria* [576]. In summary, microbial taxonomic signatures and bacterial diversity metrics demonstrate substantial clinical potential and research value in tumor diagnosis.

Bacterial metabolites: Bacterial metabolite markers play an increasingly important role in tumor diagnosis. Studies confirm intestinal microbial metabolites can accurately reflect the homeostatic state of the gut microecosystem and serve as key biomarkers for cancer diagnosis [571]. In non‐invasive diagnostic approaches, the combination of specific metabolite markers (such as γ‐aminobutyric acid, L‐aspartic acid, and phenylacetic acid) and bacterial markers (such as *F. nucleatum* and *P. anaerobius*) has significantly improved discriminatory accuracy between CRC, pre‐existing lesions (CRA), and healthy controls [571]. Metabolomic analysis has revealed primary microbial metabolites associated with tumor progression, particularly polyamine metabolites. These metabolites demonstrate significant elevation in the serum of PC patients, with notably preceding detectable histologic changes [579]. This suggests intestinal microbes analysis and microbial metabolite detection (such as polyamines) could serve as potential non‐invasive PC detection biomarkers [579]. In HCC studies, bacterial population compositional alterations and their metabolites demonstrate strong mechanistic correlations with tumorigenesis. Specifically, decreases in beneficial butyrate‐producing bacterial populations and increases in LPS‐producing bacteria are strongly associated with early HCC development. Consequently, researchers have developed and validated relevant microbial profiles that demonstrate excellent diagnostic performance in independent case‐control validation cohorts, further confirming the significant promise of microbiome in tumor diagnosis [591].

#### Viral markers

Indicators of viral infection: Viral infection‐related biomarkers represent a critical category of microbial signatures with important application value in cancer diagnosis [594, 595, 596, 597, 598]. Various oncogenic viruses, including HBV, high‐risk HPV, and EBV, can infect host cells and integrate their genomic material into the cellular genome, thereby serving as biomarkers for assessing minimal residual diseases (MRDs) through detection of viral circulating tumor DNA (ctDNA) or viral oncoproteins in liquid biopsies. For example, persistent detection of HPV ctDNA in CC patients following completion of radiotherapy strongly correlates with inferior progression‐free survival (PFS), and MRDs can be quantified through HPV‐ctDNA detection [578]. In CRC diagnosis, viral markers have demonstrated significant clinical applications. Studies have shown specific gut virome signatures, including Phage FAKO27_000271F, *Faecalibacterium* virus *Toutatis*, and *Faecalibacterium* virus *Lugh* markers, can effectively differentiate CRC patients from healthy individuals. Furthermore, distinct patterns of *Faecalibacterium* virus *Brigit*, *Streptococcus* phage *Javan191*, and *Streptococcus* phage *YMC‐2011* abundance can discriminate between CRC and CRA with clinically relevant sensitivity and specificity [572]. Additionally, a multi‐component viral signature comprising 14 novel viruses has been shown to significantly differentiate CRC patients from healthy controls, with several viral species enriched in CRC patients [573]. Moreover, phages not only participate in CRC pathogenesis but also show potential in diagnosis. Studies have confirmed specific phage prevalence in early, intermediate, and advanced CRC patients, potentially serving as CRC biomarkers [599]. In summary, viral components of the human microbiome and relative infection indicators demonstrate potential as biomarkers for cancer diagnosis.

Viral loads: Viral load and related molecular markers hold valuable diagnostic and prognostic indicators in tumor diagnosis. HBV and HCV, as major hepatotropic viral pathogens, not only significantly disrupt normal liver physiological functions but also demonstrate clear etiological associations with HCC initiation, development, and progression. MicroRNA‐122 (miR‐122) promotes HCV replication, while HBV viral load demonstrates a significant inverse correlation with miR‐122 expression. Dysregulated miRNAs mediate complex interactions at the host–virus interface and can promote viral persistence in HCC, thereby potentially serving as novel detection biomarkers [577]. Other viruses like HPV demonstrate well‐established etiological associations with malignancies. In cervical pre‐cancer (CIN) and CC, HPV viral load serves as an important risk assessment parameter [600]. Therefore, viral components of the microbiome and viral load represent important molecular tools in cancer diagnosis. In‐depth investigation of virus‐related molecular signatures and their mechanistic connections to carcinogenesis will provide critical theoretical foundations and innovative research directions for early cancer diagnosis, prognosis assessment, and personalized therapeutic strategy development.

Viral antigens: Viral antigens and their corresponding host‐derived antibody responses represent critical viral biomarkers in cancer diagnosis. Current studies explore diverse predictive biomarkers incorporating viral antigens, with the objective of enhancing diagnostic sensitivity and specificity [601]. Viral markers demonstrate particular significance in NPC diagnosis and monitoring. Serological analyses have demonstrated that five EBV‐specific antibodies, including BLRF2‐IgA, BLRF2‐IgG, and BDLF1‐IgG5, exhibit significantly elevated levels in NPC patients compared to healthy controls, highlighting their potential as biomarkers for early disease detection [581]. Follow‐up studies have confirmed that integrating these five EBV‐associated antibodies with EBNA1‐IgA significantly enhances the diagnostic accuracy for NPC [581]. Viral antigens exhibit significant clinical utility beyond NPC, contributing to diagnostic approaches for various other malignancies. In China, chronic hepatitis B and hepatitis B carriers (HBsAg positive) are correlated with significantly elevated PC risk [602], suggesting that HBsAg screening may facilitate early risk assessment and inform preventive interventions. In summary, viral components of the microbiome, particularly viral antigens and their corresponding antibody responses, play an increasingly important role in tumor diagnosis.

#### Fungal markers

Characterization of fungal communities: Studies have shown that alterations in fungal abundance and community composition within tumor tissues and associated samples correlate significantly with cancer development [603]. For example, *C. albicans* exhibits significant enrichment in GC tissues and demonstrates potential utility as a fungal biomarker [43]. Additionally, oral *M. globosa* has emerged as a candidate fungal biomarker species for GC diagnosis [214]. Another study revealed that 14 fungal biomarkers effectively distinguished CRC patients from healthy individuals across different racial populations, highlighting the significant diagnostic potential of fungal signatures in CRC [227]. In a tongue microorganism study, 14 fungal taxa, including *Ampelomyces sp IRAN 1* and related species, demonstrated significant abundance increases in GC patients and exhibited potential as diagnostic biomarkers, further expanding the taxonomic scope of fungi in cancer diagnosis [604]. Linear discriminant analysis effect size (LEfSe) analysis further revealed significant differences in fungal taxonomic profiles among patients with CRC, colorectal polyps, and healthy controls. Several fungal taxa, including *Rhodotorula dairenensis* and *Cutaneotrichosporon curvatus*, exhibited significant enrichment in CRC patients and may contribute to the formation of a tumor‐permissive microenvironment [574]. Additionally, other studies have reported the significant correlation between specific fecal fungal signatures and GC, providing rationale for the application of fecal mycobiome profiling as a non‐invasive cancer screening approach [143]. Notably, disease progression stages demonstrated significant correlation with characteristic patterns of fungal microbiota [605].

Fungal metabolites: Fungi play important roles in tumorigenesis and progression through metabolite regulation, with both fungal taxa and their secreted metabolites demonstrating significant potential as clinical biomarkers. Fungal biomarkers and metabolites potentially exhibit diagnostic potential across multiple cancer types. For example, *Candida spp*., identified as potential fungal biomarkers for OSCC, synthesize carcinogenic metabolites including nitrosamines that participate in oncogenic transformation and progression. This mechanistic relationship provides a scientific foundation for the utilization of fungal‐derived metabolomic signatures as diagnostic indicators [606]. Recent studies have identified fecal fungi, including *Sordaria pseudoproxies*, *Gibellulopsis*, and *Cercophora*, significantly correlate with elevated GC risk. They also demonstrate significant correlations with altered serum amino acid profiles, particularly methionine, L‐alanine, and L‐threonine levels, suggesting these metabolic signatures may serve as complementary biomarkers for enhanced GC detection [607].

### Microbial metabolite markers

As the field of cancer metabolomics advances, microbial‐derived metabolite signatures demonstrate significant diagnostic potential across multiple cancer types. Evidence indicates that intestinal microbial communities and their associated metabolites (including bile acid) undergo significant alterations during PC development, affecting chemotherapeutic efficacy and clinical outcomes while potentially serving as discriminatory biomarkers for PC [608]. Additionally, metabolites derived from gastrointestinal microorganisms, particularly SCFAs, demonstrate significant associations with diverse malignancies and exhibit potential as reliable biomarkers for cancer type differentiation [609]. In CRC investigations, researchers have documented significant differences in gut microbial composition and metabolic functions between healthy individuals and patients across the colorectal neoplasia continuum, from adenomas to invasive adenocarcinomas, providing novel metabolic biomarker candidates for early‐stage CRC detection [610]. Quantitative analysis of eight gut microbiota‐derived serum metabolites has validated their high diagnostic accuracy for distinguishing CRC and precancerous adenomas [611]. In lung cancer studies, specific metabolites including cysteinyl valine, 3‐chlorobenzoic acid, and 3,4‐dihydroxyphenyl ethanol effectively discriminated lung cancer patients from controls and exhibited enhanced diagnostic performance when analyzed as a composite signature, suggesting potential utility as clinically relevant biomarkers for lung cancer diagnosis [612]. Various intestinal microorganism metabolites (such as secondary bile acids) demonstrate significant associations with gastrointestinal carcinogenesis, most notably in CRC, and represent promising non‐invasive biomarkers [613]. Of particular clinical significance, early detection of PC may be achieved through analysis of intestinal microbial community alterations and metabolites (such as polyamines). These aberrant patterns exist prior to histologically detectable disease [579]. In conclusion, microbial‐derived metabolites demonstrate considerable potential as cancer biomarkers, offering novel avenues for early detection and therapeutic strategy development.

### Inflammation‐related markers

In recent years, inflammation‐related markers have emerged as promising biomarkers for cancer detection. Multiple investigations have demonstrated significant enrichment of *Parvimonas micra* (*P. micra*) in both the intestinal mucosal tissues and fecal samples of CRC patients compared to healthy controls [614]. Notably, *P. micra* colonization significantly upregulates the expression of several pro‐inflammatory mediators, including IL‐5, IL‐8, CCL20, and CSF2, which collectively contribute to the establishment and maintenance of a pro‐tumorigenic inflammatory microenvironment in CRC [614]. Therefore, *P. micra* abundance and its associated inflammatory signature may serve as clinically relevant biomarkers for CRC, providing new strategies for early detection and treatment of CRC. Additionally, *C. albicans* promotes cytokine production, particularly through Th17 cell response induction, and enhances adhesion molecule expression, thereby mediating pro‐inflammatory responses that contribute to cancer progression [417]. This mechanistic relationship suggests that *C. albicans* colonization patterns and associated inflammatory signatures may represent valuable biomarkers for cancer.

### Organ‐specific microbial markers

Applications of microbiome analysis in cancer diagnostics demonstrate distinct organ‐specific signatures, with site‐specific microbial markers offering novel approaches for early cancer detection and characterization. Existing studies have explored microbial markers in the oral cavity, skin, urinary system, and respiratory tract, collectively establishing a comprehensive framework for microbiome‐based cancer diagnosis. Table 7 summarizes different organ system key microbial markers and their clinical applications and research progress in corresponding tumor types, providing an important reference for advancing precision oncology and individualized treatment strategies.

**Table 7:** Organ‐specific microbial biomarkers and their association with tumor characteristics.

Organ system	Key microbial biomarkers	Associated cancer types	Related research	Clinical utility	References
Oral cavity	*Fusobacterium nucleatum* *Porphyromonas gingivalis* *Alloprevotella* *Streptococcus* *Malassezia globosa*	Oral cancer GC Lung cancer	• *Fusobacterium nucleatum* correlates with oral cancer gene expression; • Reduced *Streptococcus* abundance indicates GC risk; • Decreased oral α‐diversity predicts LC risk.	• Non‐invasive oral swab screening; • Pre‐cancerous lesion monitoring; • Prognostic stratification.	[162, 214]
Skin	*Staphylococcus aureus* *Ralstonia* *Diaphorobacter* *Streptococcus*	Melanoma SCC	*Staphylococcus aureus* virulence factors promote carcinogenesis.	• Skin swab diagnostic biomarkers; • High‐risk lesion.	[480, 615]
Urogenital	*Bacteroides* *Porphyrobacter* *Herbaspirillum* *Acinetobacter* *Fusobacterium* *Fenollaria/Ezakiella*	BCa PCa	• High prevalence of *Acinetobacter* in BCa; • *Fusobacterium* positively correlates with PCa invasiveness.	• Urine microbiome non‐invasive diagnosis; • Recurrence risk prediction; • Personalized therapy response assessment.	[531, 616]
Respiratory	*Streptococcus* *Prevotella* *Veillonella* *Akkermansia muciniphila*	Lung cancer	• *Streptococcus* enrichment correlates with poor prognosis; • *Akkermansia muciniphila* predicts immunotherapy response.	• Sputum/bronchoalveolar lavage fluid testing; • Immunotherapy efficacy prediction; • Survival period assessment.	[429, 617]

#### Oral microbial markers

Oral microbiome signatures represent a critical subset of organ‐specific microbial markers with demonstrated utility in disease diagnosis, prognostic stratification, and therapeutic response prediction across multiple cancer types. Recent studies have revealed that enrichment of periodontal pathogens, including *F. nucleatum*, *P. gingivalis*, and *T. denticola*, correlates with oral potentially malignant diseases and oral cancer [40, 162]. The magnitude of pathogenic bacterial enrichment is strongly associated with oncogenic genes and coincides with a significant reduction in commensal bacteria, particularly *Streptococcus*, suggesting these distinctive microbial signatures may serve as novel biomarkers for oral cancer [162]. Meta‐analytical studies have established significant correlations between oral microbiome profiles and multiple cancer types. Specifically, taxonomically diverse microorganisms, including *Alloprevotella*, *Streptococcus*, and *M. globosa*, have been characterized as potential oral biomarkers for GC [214, 618]. Notably, reduced α‐diversity within the oral microbiome has emerged as a predictive marker for lung cancer risk. Although the direct causal relationship between periodontal pathogens and lung cancer requires further mechanistic elucidation, specific microorganisms, particularly *F. nucleatum*, demonstrate potential utility as non‐invasive biomarkers for lung cancer [163]. Alterations in oral microbial composition, particularly changes in the abundance of five genera including *Bacillus spp*. and *Enterococcus spp*., effectively discriminate between patients with epithelial precursor lesions and those with invasive carcinoma, potentially serving as clinically relevant biomarkers for monitoring oral carcinogenesis progression [619]. In summary, oral microbial signatures represent essential components of the organ‐specific microbiome landscape with substantial clinical applications in cancer diagnosis, prognostic assessment, and longitudinal disease monitoring across diverse malignancies.

#### Skin microbial markers

Emerging evidence indicates that cutaneous microbiome signatures demonstrate significant diagnostic, prognostic, and risk stratification potential across various dermatological malignancies. Multiple studies have confirmed significant differences in microbiome composition and abundance (especially *S. aureus*) between melanoma and non‐melanoma skin carcinomas compared to site‐matched healthy controls [615]. Researchers have identified novel microbial markers associated with actinic keratosis (AK) or SCC, including AK‐associated *Ralstonia* and *Diaphorobacter*, as well as SCC‐associated *Ralstonia* and *Streptococcus* [480]. Host factors including immunosuppression, chronic inflammatory conditions, and oncogenic viral infections significantly increase cutaneous susceptibility to malignant transformation, while their complex interactions with the resident microbiome further modulate disease initiation and progression. Despite the significant potential of cutaneous microbial signatures as biomarkers, in‐depth exploration in this field remains relatively limited. With increasing research investment, cutaneous microbiome markers are anticipated to provide innovative strategies for early diagnosis, personalized treatment, and prognostic stratification of skin cancer.

#### Microbial markers of the urinary system

Urinary microbiome signatures represent an emerging class of non‐invasive biomarkers with significant utility in the diagnosis, molecular classification, risk stratification, and prognostic assessment of genitourinary malignancies. Recent comprehensive studies targeting urinary microbiota have systematically identified several bacterial genera with statistically significant associations with urinary tumors. The relative abundance of *Bacteroides*, *Porphyrobacter*, and *Herbaspirillum* shows significant increases in BCa patients characterized by high recurrence and progression risk, suggesting their potential value as risk stratification biomarkers [616]. To thoroughly elucidate the core microbiome characteristics of BCa, researchers integrated multiple data sets and identified 31 characteristic bacterial genera, including *Acinetobacter* [620]. The detection frequency of *Acinetobacter* was significantly higher in tumor patients, highlighting its potential value as a microbial marker for BCa.

Additionally, urinary microbial signatures have demonstrated significant clinical potential in the non‐invasive diagnosis, molecular classification, staging, and prognostic assessment of PCa. Comprehensive metagenomic analysis of post‐prostate massage urine specimens and post‐prostatectomy prostate secretions revealed that alterations in five strictly anaerobic bacterial genera, including *Fusobacterium* and *Fenollaria/Ezakiella*, significantly correlate with PCa aggressiveness and recurrence risk. This suggests their potential utility as urinary cancer biomarkers [531]. This emerging evidence not only emphasizes the important role of the urinary microbiome in the clinical management of PCa but also provides new research directions for developing novel liquid biopsy approaches and precision medicine strategies.

#### Respiratory microbial markers

The respiratory microbiome represents a complex ecological niche with emerging diagnostic, prognostic, and therapeutic implications in pulmonary malignancies. Comprehensive metagenomic analyses have demonstrated that elevated abundance of specific microbial communities within the lower respiratory tract, particularly oral commensals including *Streptococcus, Prevotella*, and *Veillonella*, is significantly associated with poor prognosis in lung cancer patients [429]. Furthermore, a clinical study encompassing patients with colorectal, breast, and lung malignancies demonstrated that intratumoral microbial diversity exhibits a significant negative correlation with immunohistopathological markers, including TILs and PD‐L1 expression. These immunological relationships are strongly associated with unfavorable clinical outcomes [345]. Additionally, the abundance of the intestinal mucinophilic bacterium *Akkermansia muciniphila* has emerged as a significant predictive biomarker for immunotherapeutic response in lung cancer patients [617]. In summary, respiratory microbiome signatures demonstrate substantial clinical utility in lung cancer detection and prognostic assessment, advancing our mechanistic understanding of lung cancer biology while establishing novel biomarker platforms and potential therapeutic targets for precision oncology approaches in pulmonary malignancies.

### Combined application of liquid biopsy and microbiome markers

The integration of microbiome analysis with liquid biopsy technologies represents a rapidly evolving frontier in cancer diagnosis, with this multimodal approach demonstrating exceptional potential. The detection of circulating microbial DNA offers the possibility of non‐invasive monitoring of tumor microbiome changes, while microbial‐derived exosomes function as biological couriers, conveying specific markers of the tumor microbiome. This combinatorial approach establishes novel paradigms for early cancer diagnosis, treatment efficacy monitoring, and recurrence surveillance across tumor types.

#### Circulating microbial DNA testing

The integration of liquid biopsy methodologies with microbiome analysis, particularly through the detection and characterization of circulating microbial DNA (cmDNA), demonstrates remarkable diagnostic and prognostic potential across diverse malignancies [621, 622, 623]. Circulating microbial DNA encompasses free DNA fragments of microbial origin detectable in peripheral blood and has demonstrated significant diagnostic value across various malignancies, particularly CRC [624]. In HCC research, significant changes in the abundance of seven bacterial species were detected in the serum of HCC patients compared to patients with cirrhosis and healthy controls, with particularly notable enrichment of *Staphylococcus*. Investigators subsequently developed a multivariate scoring model based on the abundance profiles of five bacterial species that successfully discriminated HCC patients, strongly suggesting the potential application of cmDNA in HCC diagnosis [592].

Beyond HCC, cmDNA has demonstrated significant diagnostic and prognostic value in other cancer types. For instance, consistently detected HPV‐ctDNA following chemoradiotherapy (CRT) for CC correlates with inferior PFS. Assessment of MRD through HPV‐ctDNA has demonstrated substantial clinical utility [578]. In oropharyngeal cancer (OPC) studies, detection of HPV‐16 DNA in plasma and saliva serves as a highly sensitive predictor of disease recurrence [625]. These collective findings not only underscore the critical role of HPV in cancer diagnosis and prognostic assessment but also establish scientific foundations for the clinical application of cmDNA detection technology across a diverse spectrum of malignancies.

#### Exosomes of microbial origin

Extracellular Vesicles (EVs) of microbial origin have demonstrated significant value as novel biomarkers in disease diagnosis. A systematic analysis of bacterial EVs isolated from both peripheral blood and tissues of patients with BTs revealed significant differences in bacterial EV distribution between BT patients and healthy controls. Specifically, *Saccharibacteria*, *Prevotellaceae*, and *Dialister* demonstrated significant depletion in both circulation and tissue microenvironments of BT patients, and *Erysipelotrichia* was significantly increased in both circulation and tissue microenvironments of BT patients. *Lachnospiraceae NK4A136* exhibited divergent compartment‐specific alterations, showing significant enrichment in peripheral blood yet concurrent depletion in tumor tissues of BT patients [626]. This finding elucidates the potential value of bacterial EVs for BT diagnosis, particularly for detecting early‐stage lesions that conventional imaging modalities fail to identify [626]. Furthermore, bacterial EVs in serum have important applications in HCC diagnosis [592]. A study developed a diagnostic method based on detecting glypican‐1 (GPC1), a membrane‐anchored proteoglycan enriched on the surface of EV. This approach effectively differentiates patients with benign pancreatic disorders from those with pre‐malignant pancreatic lesions and demonstrates superior sensitivity for identifying patients with advanced PC, offering significant advantages over conventional PC marker carbohydrate antigen 19‐9 (CA19‐9) [627]. In cancer biology research, EV‐based microRNA characterization provides novel concepts for developing clinical diagnostic algorithms applicable to early detection of various gastrointestinal cancers, including CRC [628]. In summary, the application of microbial‐derived EVs, representing a combination of liquid biopsy and microbiome markers, has demonstrated significant diagnostic advantages across various malignancies, including biliary tract tumors, HCC, and gastrointestinal cancers. This multidisciplinary approach has established innovative research directions for early disease diagnosis and treatment plan development.

### Standardization of microbiome diagnostic methods

The analytical validity and reproducibility of microbiome‐based diagnostic applications depend critically on rigorous standardization of sample collection protocols. Consequently, implementation of standardized operating procedures for specimen acquisition represents a fundamental prerequisite for generating reproducible, clinically actionable microbiome data.

#### Specifications for sample collection and handling

Implementation of standardized microbial sample collection and processing protocols represents a critical determinant of analytical accuracy and reliability. Acknowledging the spatial heterogeneity of microbial community distribution across anatomical niches [629], standardization efforts must ensure consistent sampling site selection with precise anatomical localization and documentation. Specimens must be collected in statistically sufficient quantities to ensure adequate representation of microbial diversity and biomass, with optimal sampling preferably performed during the acute phase of disease and before initiating antimicrobial therapy. Sampling procedures must follow standardized aseptic protocols to minimize environmental and cross‐site contamination from commensal microbiota. Acquired specimens should avoid contact with disinfectants or preservatives and be promptly submitted for testing [630]. Samples should be collected in sterile, sealed, specialized containers to maintain microbial viability during transportation [630]. Additionally, sample‐specific processing workflows must be established based on the unique biological characteristics and stability profiles of distinct specimen types. Specimens from sterile sites, including cerebrospinal fluid and body fluids, require expedited processing protocols. Fecal and urinary specimens, however, demonstrate microbiome stability and can be maintained under validated preservation conditions for extended periods. For sensitive organisms such as *Shigella spp*. and *Neisseria gonorrhoeae*, immediate sample processing is necessary to ensure testing accuracy [631]. A standardized system for microbiome research requires establishing evidence‐based standard operating procedures, encompassing critical elements including sampling site consistency, aseptic protocol implementation, appropriate collection method selection, suitable transport media, and differentiated sample processing strategies.

#### Standardization of detection methods

The standardization of microbiome characterization methodologies represents a fundamental challenge in translating microbiome research. Currently, microbial taxonomic classification primarily employs two complementary analytical approaches, targeted amplicon sequencing of phylogenetic marker genes and metagenomic shotgun sequencing analysis, which collectively provide robust technical frameworks for systematic microbiome investigation [632]. Establishing standardized processes is equally essential for implementing rapid detection techniques. These techniques include immunofluorescence, agglutination tests, immunochromatography (ICT), enzyme immunoassays (EIA), and molecular microbiology techniques, all widely implemented in clinical microbiome analysis due to their rapid turnaround time, operational simplicity, and diagnostic performance characteristics [631]. However, advanced assays alone cannot guarantee diagnostic performance; systematic optimization of the entire analytical workflow remains indispensable. Laboratories typically adopt selective testing strategies, including taking a cautious approach to culture results of suspected contaminants and avoiding unnecessary antimicrobial susceptibility tests. These practices collectively represent critical strategies for enhancing diagnostic specificity and optimizing antimicrobial stewardship [633]. The standardization of microbiome diagnostic methods requires not only harmonization of testing techniques but also optimization of testing processes and standardized application of rapid testing detection methods. Collectively, these measures constitute vital components of a standardized microbiome diagnostic framework, establishing a solid foundation for improving diagnostic accuracy, optimizing antimicrobial use, and advancing microbiome research.

## MICROBIOME IN TUMOR PROGNOSIS AND EFFICACY PREDICTION

The microbiome has emerged as a crucial bioindicator for tumor prognosis assessment and treatment efficacy prediction. Evidence indicates that alterations in microbial diversity, taxonomic composition, and metabolite profiles correlate significantly with clinical outcomes in cancer patients. The microbiome influences therapeutic outcomes through multiple mechanisms, including modulation of tumor response to chemotherapy, radiotherapy, and immunotherapy, while also contributing to treatment‐related adverse events. Table 8 summarizes current evidence regarding microbiome‐based biomarkers for tumor prognosis prediction and treatment response assessment, along with their potential clinical applications.

**Table 8:** Key roles and clinical applications of the microbiome in tumor prognosis and treatment response prediction.

Application direction	Tumor type	Key microbes/metabolites	Key roles	Clinical application strategies	References
Prognostic prediction					
Microbial diversity	PC	*Streptococcus* *Prevotella* *Veillonella*	Reduced microbial diversity correlates with shorter survival (HR = 1.8).	Multi‐site microbial detection (oral& gut) to guide postoperative management.	[429, 634]
	HNSCC	Postoperative microbial dynamics	Increased α‐diversity in recurrent patients (*p* = 0.006).	Postoperative microbiome monitoring to predict recurrence risk.	[635]
	NSCLC	Gut microbial diversity	High diversity linked to better immunotherapy response.	Stratification of immunotherapy patients.	[636]
Specific microbial biomarkers	CRC	*Fusobacterium nucleatum*	Tumor‐enriched *F. nucleatum* shortens OS and promotes pro‐inflammatory microenvironment.	Fecal testing to identify high‐risk patients guiding adjuvant chemotherapy intensity.	[164]
	BC GC LUAD	*Tissierella*	Higher abundance correlates with prolonged OS (*p* < 0.01) and inhibits metastasis.	Development of probiotic formulations to enhance anti‐tumor efficacy.	[44]
	HCC	*Lachnoclostridium* ↑ *Prevotella 9* ↓	Combined signature predicts high response to ICI.	FMT to optimize immunotherapy.	[637]
	Lung cancer	*Prevotella* *Streptococcus* *Veillonella*	Enrichment correlates with poor survival prognosis (*p* < 0.05).	Sputum testing to guide prognosis management.	[429]
	CC	*Microbacterium* *Streptococcaceae*	Higher abundance correlates with reduced OS/RFS (*p* < 0.001).	Identification of high‐risk patients; Recurrence monitoring.	[638]
	NSCLC	*Bacteroides dorei* *Parabacteroides distasonis*	Enrichment linked to prolonged OS (>6 mo).	Gut microbiome testing to identify patients likely to benefit from immunotherapy.	[639]
Metabolite biomarkers	HCC	Galanthaminone (bacterial metabolite)	High levels correlate with prolonged OS in ICI therapy, Inhibits PD‐1/PD‐L1 pathway.	Dynamic serum metabolite monitoring to guide ICI treatment timing.	[640]
	HCC	Bile acids (fecal metabolites)	Specific bile acid profiles correlate positively with ICI efficacy.	Personalized immunotherapy strategy development.	[637]
	Pan‐cancer	Butyrate	Activates T cell anti‐tumor activity; Enhances PD‐1 efficacy.	Oral butyrate supplementation combined with immunotherapy.	[641]
Efficacy prediction					
Drug resistance	PC	*Escherichia coli*	Enzyme‐mediated gemcitabine inactivation.	Antibiotics (such as ciprofloxacin) combined with chemotherapy to reverse resistance.	[642]
	Pan‐Cancer	High butyrate/propionate levels	Suppresses CTLA‐4 efficacy (*p* = 0.01), promotes Treg expansion.	Low‐fiber diet to reduce SCFAs and enhance immunotherapy response.	[643]
Adverse effects	CRC	*Escherichia coli* β‐glucuronidase	Converts irinotecan to toxic metabolites.	β‐glucuronidase inhibitors (such as nicasyn) to reduce intestinal toxicity.	[644]
	Melanoma	*Bifidobacterium*	Supplementation reduces colitis incidence.	Probiotic formulations to support immunotherapy and reduce toxicity.	[47]
	Radiation Dermatitis	Reduced skin microbiome diversity	Post‐radiation dysbiosis exacerbates inflammation.	Topical probiotic ointments to restore skin barrier.	[645]

Abbreviations: CTLA‐4, cytotoxic T lymphocyte associate protein‐4; FMT, fecal microbiota transplantation; HNSCC, head and neck squamous cell carcinoma; IL‐17, Interleukin‐17; LUAD, lung adenocarcinoma; OS, overall survival; PD‐1, programmed death 1; PD‐L1, programmed cell death ligand 1; RFS, recurrence‐free survival.

### Microbiome and tumor prognosis

#### Microbial diversity and prognosis

Microbial diversity and its influence on tumor prognosis have emerged as a critical focus in contemporary oncological research [646]. Evidence suggests that microbiota from both oral cavity and intestinal tract are associated with PC prognosis, with *Streptococcus spp*., *Prevotella spp*., or *Veillonella spp*. showing significant negative correlations with survival outcomes [429, 634]. These findings indicate that microorganisms from multiple anatomical sites have potential as prognostic biomarkers for PC. Previous studies have revealed significant alterations in the microbiome composition of patients with HNSCC before and after surgery. Microbial α‐diversity significantly decreases following surgery but subsequently increases in patients experiencing tumor recurrence [635], indicating that microbiota diversity patterns may serve as predictive indicators for HNSCC prognosis. Additionally, higher gut microbiome diversity consistently correlates with favorable clinical responses in NSCLC patients undergoing immunotherapy [636].

#### Specific microbial species and prognosis

Accumulating evidence highlights the prognostic significance of specific microbial species in predicting tumor progression and treatment outcomes. Multiple investigations have confirmed that oral mucositis (OM), a common adverse effect of cancer treatment. It exhibits variations in microbial composition that correlate with disease severity, specifically decreased abundance of *Prevotella*, *Leptotrichia*, and *Actinomyces* alongside increased prevalence of *Treponema* [647]. In CRC, the abundance of *F. nucleatum* in tumor tissues may have the potential to serve as a prognostic biomarker [164]. In vivo experiments have established that *E. coli* significantly reduces gemcitabine's anti‐tumor effect, resulting in increased tumor burden and compromised patient survival [46]. Conversely, co‐administration of *Lactobacillus* with cisplatin significantly suppresses tumor growth and improves survival in mouse models of lung cancer [648].

Notably, a potentially anti‐cancer microbiome genus, *Tissierella*, has been significantly associated with improved prognosis across various tumors, including breast, lung, and gastric cancers, further confirming the critical role of specific microbial species in tumor prognosis [44]. Additionally, the salivary microbiota of PC patients exhibits unique prognosis‐related features. Poor oral hygiene and periodontal disease caused by microbial dysbiosis represent independent risk factors for PC, underscoring the oral microbiome's significant role in PC prognosis [429, 649]. Moreover, differential abundances of *Neisseria elongata* and *Streptococcus mitis* in saliva samples from PC patients compared to healthy controls demonstrate potential as non‐invasive biomarkers for PC prediction [650].

In lung cancer studies, enrichment of *Prevotella spp*., *Streptococcus spp*., and *Veillonella spp*. consistently correlates with inferior survival outcomes [429]. Specifically, bacterial abundance in normal tissues adjacent to lung cancer correlates with recurrence‐free survival (RFS). Significant positive correlations exist between *Koribacteraceae* family enrichment and RFS, while inverse correlations are observed between *Bacteroidaceae*, *Ruminococcaceae*, and *Lachnospiraceae* family enrichment and RFS [651].

Viral–microbial interactions play equally important roles in tumor prognosis. HPV has been demonstrated to be a potent prognostic biomarker, with HPV‐positive patients typically experiencing better prognosis, while the presence of HPV DNA in plasma and saliva serves as an effective predictor of OPC recurrence [601, 652]. In HPV‐independent cervical adenocarcinoma, the abundance of *Microbacterium* and *Streptococcaceae* family microorganisms significantly correlates with diminished OS and RFS, further validating the utility of specific microbial signatures as prognostic indicators [638].

In patients with HCC undergoing immunotherapy, substantial evidence demonstrates significant associations between fecal microbiome composition and patients' OS and PFS [637]. Specifically, *Lachnoclostridium* enrichment coupled with *Prevotella 9* depletion correlates with improved OS, while patients harboring favorable microbial signatures exhibit extended PFS [637]. Enrichment of *Bacteroides dorei* and *Parabacteroides distasonis* in NSCLC patients treated with ICIs significantly correlates with prolonged OS, whereas abundance of *Chaetosphaeriales*, *Cortinarius davemallochii*, and *Helotiales* associates with shorter OS. These findings illuminate the complex interplay between the microbiome and therapeutic efficacy in cancer patients [639]. Consequently, the broad application of microbiome analysis in tumor prognosis prediction, while elucidating the relationships between specific microbial taxa and clinical outcomes, provides novel avenues for developing precision oncology approaches for cancer patients.

#### Microbial metabolites and prognosis

Microbial metabolites play crucial roles in modulating tumor progression and treatment outcomes. Investigations have identified the bacterial metabolite galanthaminone as a biomarker for predicting outcomes in HCC patients treated with ICIs. It provides novel insights for clinical applications of microbial metabolites in tumor prognosis assessment [640]. Further studies have demonstrated that fecal microbiota and their metabolites, especially bile acids, are closely associated with clinical efficacy and prognosis in HCC patients undergoing immunotherapy [637]. These observations substantiate the prognostic and predictive value of gut microbial communities and their metabolic derivatives in predicting ICI therapeutic responses in HCC patients. A study has revealed that the gut microbial metabolite butyrate plays a pivotal role in modulating CD8^+^ T cell functionality and phenotype, significantly potentiating anti‐PD‐1 immunotherapy efficacy. This important finding establishes butyrate as a potential prognostic biomarker for enhancing anti‐tumor immune responses [641]. Collectively, microbial metabolites, as functional mediators of the microbiome, demonstrate potential for integration into tumor prognostication frameworks and therapeutic response prediction.

### Microbiome and tumor treatment efficacy

The relationship between the microbiome and tumor treatment efficacy has been comprehensively investigated. The composition and abundance of the microbiome closely correlate with treatment outcomes while simultaneously exerting significant impacts on tumor therapy resistance. Furthermore, perturbations in microbiome structure and function significantly contribute to treatment‐related toxicities observed during oncologic interventions.

#### Microbiome and drug resistance

The contribution of tumor‐associated microbiota to chemoresistance mechanisms has emerged as a critical focus in translational oncology research. Studies have confirmed that colonizing bacteria present in the PC microenvironment can induce gemcitabine resistance and attenuate its cytotoxic efficacy through expression of specialized cytidine deaminase enzymes [642]. Further research has found that antibiotic therapy can effectively inhibit this drug inactivation pathway, consequently restoring gemcitabine's therapeutic efficacy [642]. Beyond gemcitabine, the intestinal microbiota demonstrates the capacity to mediate resistance to diverse chemotherapeutic agents, including cyclophosphamide, 5‐fluorouracil (5‐FU), and oxaliplatin [653]. However, high concentrations of SCFAs (butyrate and propionate) in the microenvironment have demonstrated significant associations with resistance to CTLA‐4 blockers [643].

#### Microbiome and adverse effects

Investigating the interplay between the microbiome and tumor therapy outcomes (including efficacy and toxicity) has emerged as a central paradigm in cancer research. Extensive research has confirmed that gut microbiota directly modulates the efficacy of chemotherapeutic agents via metabolic regulation. Specifically, *E. coli* converts irinotecan into toxic metabolites through β‐glucuronidase expression, resulting in severe diarrhea. A clinical investigation has found that co‐administration of β‐glucuronidase inhibitors significantly attenuates intestinal epithelial damage [644]. Additionally, *E. coli* possesses nitroreductase activity that bioactivates the prodrug CB 1954, enhancing its cytotoxic effects while concurrently compromising gemcitabine's anti‐tumor activity, resulting in increased tumor burden and diminished survival outcomes [46]. In tumor immunotherapy, perturbations in intestinal microbial homeostasis significantly exacerbate immune‐mediated adverse events. For instance, antibiotic therapy worsens anti‐CTLA‐4 therapy‐induced colitis, whereas *Bifidobacterium* supplementation mitigates intestinal inflammatory pathology through suppression of pro‐inflammatory cytokine cascades [47, 654]. Cutaneous microbiome perturbations strongly correlate with treatment‐related toxicity. Significant reductions in cutaneous microbial diversity and alterations in taxonomic proportions during radiotherapy or EGFR inhibitor treatment combined with chemotherapy potentiate localized inflammatory response. This condition may precipitate adverse effects including radiation dermatitis, palmar–plantar erythrodysesthesia syndrome, and papulopustular eruptions [645, 655]. Notably, specific microbial characteristics demonstrate utility as predictive biomarkers for treatment‐associated toxicities. For example, in metastatic RCC patients treated with vascular endothelial growth factor tyrosine kinase inhibitors (VEGF‐TKI), the elevated abundance of *Bacteroides spp*. shows a significant positive correlation with diarrhea incidence, while *Prevotella spp*. demonstrates a protective effect [656]. These insights suggest that precision microbiome modulation strategies, including microbial transplantation or combination with specific enzyme inhibitors, may constitute innovative approaches to optimize the safety window of oncologic interventions.

## MICROBIOME AND TUMOR THERAPY

### Microbiome and chemotherapy

**Figure 5:**
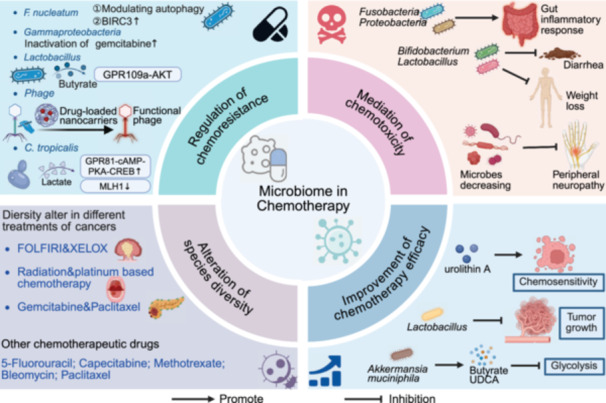
The role of microbiome in chemotherapy. This figure illustrates four key aspects of microbiome‐chemotherapy interactions. Regulation of chemoresistance (upper left): Various microorganisms, including *F. nucleatum*, *Gammaproteobacteria*, *Lactobacillus*, Phage, and *C. tropicalis*, influence drug resistance through different mechanisms. Mediation of chemotoxicity (upper right): Specific microorganisms (*Fusobacteria*, *Proteobacteria*, *Bifidobacterium*, and *Lactobacillus*) influence chemotherapy side effects, including gut inflammatory responses, diarrhea, and weight loss. Additionally, decreases in specific microbial populations may contribute to chemotherapy‐induced peripheral neuropathy. Alteration of species diversity (lower left): This section demonstrates different chemotherapy treatments' effects on microbial populations, including FOLFIRI/XELOX in CRC, radiation with platinum‐based chemotherapy in head and neck squamous cell carcinoma, and combinations of gemcitabine and paclitaxel in PC. Additional chemotherapeutic agents contributing to microbial community alterations include 5‐Fluorouracil, capecitabine, methotrexate, bleomycin, and paclitaxel. Improvement of chemotherapy efficacy (lower right): *Akkermansia muciniphila* influences tumor response through UDAC and butyrate production, subsequently inhibiting tumor glycolysis. Furthermore, urolithin A enhances chemosensitivity, while *Lactobacillus* species exhibit tumor growth inhibition. (Red text for anti‐chemoresistance; Black text for pro‐chemoresistance). CRC, colorectal cancer; *C. tropicalis*, *Candida tropicalis*; FOLFIRI, folinic acid (leucovorin), fluorouracil (5‐FU), and irinotecan; UDAC, ursodeoxycholic acid; XELOX, capecitabine (Xeloda) and oxaliplatin.

The relationship between microbiome and tumor therapy, with particular emphasis on chemotherapeutic modalities, has emerged as a rapidly expanding research domain. Bacteria, viruses, and fungi all significantly impact chemotherapy efficacy, while chemotherapeutic agents simultaneously affect microbiome composition and function. Furthermore, microbiome alterations may mediate toxic effects during chemotherapy, impacting therapeutic tolerability and health‐related quality of life. Consequently, microbiome modulation strategies to enhance chemotherapy efficacy while attenuating treatment‐limiting toxicities represent an emerging direction in cancer therapy. As illustrated in Figure 5, this framework encompasses the bidirectional regulatory networks between microbial ecosystems and chemotherapeutic agents, including influences exerted by bacterial, viral, and fungal constituents, chemotherapy‐induced alterations in the microbiome, microbe‐mediated toxicity mechanisms, and microbiome‐based intervention strategies.

#### Impact of the microbiome on chemotherapy

Role of bacteria: The regulatory influence of microbiota on antineoplastic agent pharmacodynamics, particularly intestinal microbial communities, has emerged as a central focus in translational oncology research. Certain bacteria, such as *Akkermansia muciniphila*, enhance chemotherapy efficacy [657]. In CRC, the intestinal microbiota closely relates to chemotherapeutic efficacy and resistance. *F. Nucleatum* can induce chemotherapy resistance and reduce the therapeutic effect [165, 166, 167], while butyric acid and its derivatives can alleviate the chemotherapy resistance induced by *F. Nucleatum* [165]. The microbial‐derived butyrate, beyond glucose metabolism inhibition, targets the G protein‐coupled receptor 109a‐AKT signaling pathway and enhances chemotherapeutic anti‐tumor effects [658]. In PC, *Gammaproteobacteria* enhance chemotherapeutic agent inactivation, including gemcitabine, substantially diminishing therapeutic efficacy [642, 659]. However, certain probiotics or microbiota metabolites may benefit CRC treatment. For example, *Lactobacillus plantarum*‐derived metabolites augment butyrate‐mediated tumor suppression and reverse multiple drug resistance [660]. Additionally, specific oral bacterial species diminish chemotherapeutic efficacy while simultaneously exacerbating treatment‐induced oral mucositis [456]. In summary, bacterial effects on chemotherapeutic drug metabolism exhibit remarkable complexity, playing crucial roles in tumor chemotherapy.

Role of viruses: Viruses, particularly bacteriophages, have emerged as remarkable mediators of chemotherapeutic efficacy. Experimental evidence demonstrates that engineered bacteriophages conjugated with chemotherapeutic‐loaded nanoparticles significantly potentiate cytotoxic activity against malignant cells [661]. Additionally, several studies have hypothesized that HPV‐positive HNSCC exhibits better responses to radiotherapy [662, 663]. Therefore, bacteriophages represent promising biological vectors for modulating microbiome‐mediated chemotherapeutic metabolism, offering remarkable application prospects for enhancing antineoplastic efficacy.

Role of fungi: Fungal communities have emerged as critical mediators of chemotherapeutic pharmacokinetics and treatment response through diverse metabolic and immunological mechanisms. Studies show that *Candida tropicalis* (*C.tropicalis*) is closely associated with CRC and promotes chemotherapy resistance through dual mechanisms, including lactate production and suppression of MLH1 expression. In this process, lactate, as a critical signaling metabolite, regulates MMR protein expression levels through activation of the GPR81‐cAMP‐PKA‐CREB signaling pathway, enhancing tumor cell chemoresistance [664]. To further elucidate fungal mechanisms in chemotherapeutic responses, investigators employed amphotericin B to selectively deplete the mycobiome, demonstrating that fungal ablation significantly enhanced gemcitabine cytotoxicity [219]. These findings reveal the critical contribution of fungi to chemotherapeutic agent metabolism and treatment efficacy, identifying potential interventional targets for novel adjunctive strategies to optimize chemotherapeutic outcomes.

#### Effect of chemotherapy on the microbiome

Chemotherapeutic interventions induce profound and heterogeneous alterations in microbial communities, exhibiting notable variability across malignancy types and chemotherapeutic agents. In HNSCC patients, chemotherapy and radiotherapy significantly alter the salivary microbiome, evidenced by substantially increased *Candida* and reduction in overall bacterial and fungal diversity [665]. In CRC patients, different chemotherapy regimens (including FOLFIRI[fluorouracil, leucovorin, and irinotecan] and XELOX[capecitabine and oxaliplatin]) induce significant taxonomic restructuring of intestinal microbial communities, with characteristic alterations in bacterial and fungal population dynamics [666]. PC patients treated with different chemotherapeutic agents (gemcitabine and paclitaxel) exhibit differentiable patterns of intestinal microbiome perturbation [667, 668]. Additional chemotherapeutic agents, including pyrimidine analogs (5‐fluorouracil) and platinum compounds (oxaliplatin), similarly induce characteristic microbial compositional shifts [669, 670]. 5‐FU treatment induces significant restructuring of intestinal microbial communities, decreasing abundance of *Streptococcus spp*. and *Bacteroides spp*. while increasing enrichment of *Clostridium hathewayi* and *Lachnospiraceae* [671]. Gemcitabine significantly decreases the proportion of *Firmicutes* and *Bacteroidetes* [667]. Additionally, capecitabine, methotrexate, bleomycin, and paclitaxel modulate the composition and abundance of gut microbiome [671]. Furthermore, chemotherapeutic interventions exert modulatory effects beyond intestinal microbial communities, significantly influencing intratumoral microbiome composition and ecological diversity. Comprehensive analyses reveal significant reductions in intratumoral bacterial diversity and characteristic shifts within the post‐treatment TME [548, 672]. This microbial community restructuring demonstrates substantial correlations with therapeutic response parameters and treatment‐associated adverse effects profiles.

#### Microbiome‐mediated toxicities of chemotherapy

Accumulating evidence establishes the microbiome as a critical mediator of chemotherapy‐induced toxicities. During irinotecan administration, gut microbial structure experiences profound alteration, characterized by decreased microbial diversity with elevated proportions of *Fusobacteria* and *Proteobacteria* phyla. This dysbiosis positively correlates with intestinal inflammatory responses, potentially exacerbating chemotherapy‐induced toxicity [673]. Studies demonstrate that specific bacterial strains (including *Bifidobacterium* and *Lactobacillus*) significantly alleviate irinotecan‐induced weight loss and diarrhea, further confirming the microbiome's critical role in modulating chemotherapy‐induced adverse effects [674]. Additionally, Shen et al. demonstrated that transient intestinal microbial depletion through targeted antibiotic intervention significantly attenuates oxaliplatin‐induced peripheral neuropathy in preclinical models, providing mechanistic evidence for understanding gut microbiome's role in oxaliplatin toxicity [675]. These findings elucidate the microbiome's role in modulating chemotherapeutic agent efficacy and reveal its important mechanisms in mediating chemotherapeutic toxicity, providing new research directions for developing precision microbiome‐targeted interventions to minimize therapeutic adverse effects [676].

#### Microbiome modulation strategies to improve chemotherapy outcomes

Microbiome‐based therapeutic interventions demonstrate considerable potential for enhancing chemotherapeutic efficacy. Accumulating evidence indicates that intestinal microbial communities significantly influence chemotherapeutic outcomes through both direct effects and metabolite modulation. Specifically, commensal bacteria with immunomodulatory properties like *Akkermansia muciniphila* significantly potentiate chemotherapeutic efficacy [657]. Additionally, gut microbiota‐produced metabolites such as butyrate and ursodeoxycholic acid (UDCA) enhance chemotherapeutic efficacy through multiple mechanisms, including glucose metabolism inhibition, specific signaling pathway modulation, and intestinal microbial community reconfiguration [677]. Metabolites like urolithin A can enhance cancer cell chemosensitivity and ameliorate chemotherapy‐induced systemic toxicities [678]. Furthermore, microbiome‐targeted modulation influences gemcitabine metabolism, generating synergistic therapeutic effects in specific cancer types [642]. Experimental studies demonstrate that cisplatin combined with *Lactobacillus* supplementation yields enhanced therapeutic responses in murine pulmonary carcinoma models, manifesting as significant tumor growth inhibition and prolonged survival intervals. The outcome closely relates to alterations in oncogene expression profiles and notable augmentation of anti‐tumor immune responses [648]. In summary, microbiome modulation strategies provide promising adjunctive therapeutic approaches to enhance chemotherapeutic efficacy. Through optimizing microbial communities and their metabolic outputs, these strategies may offer potential for significantly improved therapeutic outcomes while concurrently mitigating treatment‐associated adverse events, ultimately enhancing quality of life metrics for oncology patients.

### Microbiome and radiotherapy

Host‐associated microbial communities demonstrate significant bidirectional interactions with radiotherapeutic outcomes across diverse malignancies. Microbial ecosystems influence tumor radiosensitivity while concurrently, ionizing radiation significantly perturbs microbial community structures. Additionally, the microbiome may mediate radiotherapy's toxicities, potentially exacerbating treatment‐related burdens in patients.

Within the interaction between radiotherapy and the microbiome, distinct hierarchical patterns emerge regarding the clinical relevance of various bacteria, viruses, and fungi. Bacteria occupy a predominant position due to their extensive involvement in regulating radiotherapy sensitivity and mediating radiation‐induced toxicities. For example, *Lactobacillus iners* modulates CC and *Bacteroides vulgatus* influences rectal cancer response to radiotherapy [436, 679], with targeted probiotic interventions already demonstrating clinical efficacy for mitigating radiation‐induced enteropathy [680]. Among viruses, HPV demonstrates a well‐established role in HNSCC radiotherapy sensitivity. It induces immunomodulation significantly enhancing control rates, though its effects remain restricted to virus‐associated malignancies [662]. Fungal research primarily focuses on the bidirectional regulation between intestinal commensal fungi and the post‐radiotherapy immune microenvironment, though mechanistic understanding and clinical translation remain insufficient. In summary, bacterial research is the most systematic and has the greatest value in clinical translation in radiotherapy. Viral factors provide established clinical guidance in specific malignancy subtypes, while fungal contributions to radiotherapeutic outcomes warrant further mechanistic investigation.

**Figure 6:**
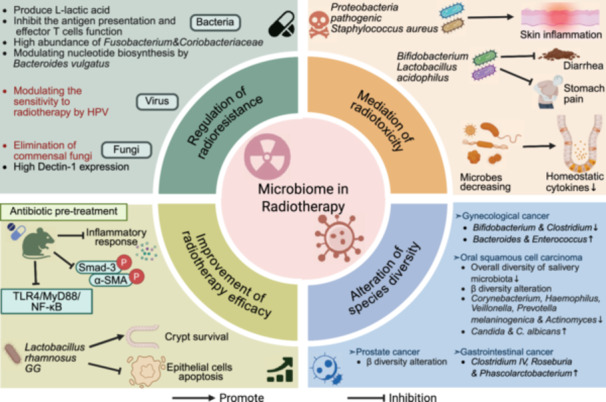
The role of microbiome in radiotherapy. This figure illustrates four key aspects of microbiome‐radiotherapy interactions. Regulation of radioresistance (upper left): Key mechanisms related to bacteria include L‐lactic acid production, affecting antigen presentation and effector T cell function, elevated abundance of *Fusobacterium* and *Coriobacteriaceae*, and nucleotide biosynthesis modulation by *Bacteroides vulgatus*. HPV enrichment or commensal fungi elimination improves radiotherapy sensitivity, whereas high Dectin‐1 expression of fungi contributes to resistance mechanisms. Mediation of radiotoxicity (upper right): Microbes including *Proteobacteria*, *pathogenic Staphylococcus aureus* trigger skin inflammation. *Lactobacillus acidophilus* plus *Bifidobacterium* supplementation correlates with reduced diarrhea and abdominal pain. Radiation‐induced decreases in microbial populations are associated with reduced homeostatic cytokine levels. Alteration of species diversity (lower right): Distinct microbial community changes occur across different anatomical sites. Gynecological cancer patients after radiotherapy show intestinal *Bifidobacterium* and *Clostridium* reduction concurrent with *Bacteroides* and *Enterococcus* increases. In OSCC, β‐diversity shows differences, particularly in *Corynebacterium*, *Haemophilus*, *Veillonella*, *Prevotella melaninogenica*, *Actinomyces*, and *Mycoplasma* populations. Additionally, *Candida* and *C. albicans* increased following radiotherapy. Only gut microbiome β‐diversity alterations were found in prostate cancer patients during radiotherapy. Likewise, gut microbiota diversity in CRC patients undergoing radiotherapy shows elevation with significant enrichment of *Clostridium IV*, *Roseburia*, and *Phascolarctobacterium*. Improvement of radiotherapy efficacy (lower left): Antibiotic pre‐treatment suppresses inflammatory responses, TLR4/MyD88/NF‐κB pathway signaling, and Smad‐3/pSMAD pathway activation to improve radiotherapy efficacy. Furthermore, *Lactobacillus rhamnosus GG* promotes intestinal crypt survival while inhibiting epithelial cell apoptosis. (Red text for anti‐radioresistance; Black text for pro‐radioresistance). OPC, oropharyngeal cancer; OSCC, oral squamous cell carcinoma; pSMAD, phosphorylated SMAD; TLR4, Toll‐like receptor 4.

Consequently, the development of targeted microbiome modulation strategies to potentiate radiotherapeutic efficacy while attenuating radiation‐induced normal tissue complications represents an emerging paradigm in precision radiation oncology. As illustrated in Figure 6, the microbiome‐radiotherapy interaction manifests in four key aspects: microbial community contributions to tumor radiosensitivity modulation, radiotherapeutic effects on microbial composition, microbiome‐mediated radiotherapy‐induced toxicities, and microbiome modulation intervention to improve radiotherapeutic outcomes.

#### Effect of microbiome on sensitivity to radiotherapy

The regulation of tumor radiosensitivity by the microbiome has emerged as a critical research area. Preclinical investigations utilizing murine models demonstrate that radiotherapeutic efficacy is determined by an intricate interplay between intrinsic tumor characteristics and intestinal microbial community composition, encompassing bacterial and fungal constituents [681]. Across diverse cancer types, gut microbes significantly modulate radiotherapy efficacy and influence radiotherapy‐induced adverse effects [681]. For instance, *Lactobacillus iners* produces L‐lactic acid, which is significantly associated with unfavorable clinical outcomes and induces radiotherapy resistance in CC cells [436]. Additionally, intestinal microbial dysbiosis may constitute an important mechanism of primary radiotherapeutic resistance through impairing anti‐tumor immune responses via inhibition of antigen presentation and effector T cell function [682].

The microbiome's clinical significance is particularly prominent in rectal cancer treatment. Specific microbes and metabolic pathway indicators serve as biomarkers for predicting efficacy and adverse effects in rectal cancer patients undergoing neoCRT [683]. A prospective longitudinal investigation confirmed that there are remarkable differences in the microbiome between responders and non‐responders to treatment. For example, butyrate‐producing bacteria dominated in radiotherapy‐sensitive individuals, whereas *Fusobacterium* and *Coriobacteriaceae* predominated in non‐responders [684]. Recent investigations have revealed that *Bacteroides vulgatus* inhibits rectal cancer radiosensitivity. Through modulating the nucleotide biosynthesis pathway, *Bacteroides vulgatus* substantiates the critical regulatory role of specific bacterial taxa in regulating radiotherapeutic responses [679]. Collectively, diverse bacterial taxa demonstrate remarkable modulatory effects on tumor radiosensitivity. Elucidation of these regulatory pathways will provide a basis for developing novel microbiome‐targeted strategies to enhance tumor radiotherapy efficacy.

Viral components of the microbiome have garnered increasing attention in tumor radiosensitivity regulation. Studies show that HPV‐positive patients with HNSCC exhibit higher radiotherapeutic sensitivity [662]. Clinical data confirms that HPV‐positive HNSCC patients demonstrate significantly higher local control rates following radiotherapy, representing a principal factor of improved OS [685]. Further mechanistic studies have revealed that HPV‐positive HNSCC patients exhibit significantly prolonged OS following radiotherapy compared to HPV‐negative cohorts [685]. These favorable prognostic distinctions appear related to HPV‐mediated alterations in HNSCC.

Recent comprehensive investigations have examined mycobiome‐mediated regulatory mechanisms influencing tumor response to radiotherapy. Intestinal fungal communities significantly modulate post‐radiotherapy anti‐tumor immune responses in murine models of BC and melanoma, demonstrating regulatory patterns contrast with those observed in bacterial communities [686]. Specifically, antifungal interventions enhance tumor radiotherapy efficacy by eliminating commensal fungi, while triggering a decrease in commensal bacterial abundance is associated with poor efficacy. Furthermore, elevated expression of Dectin‐1 in BC is associated with unfavorable survival outcomes. In murine radiotherapy models, Dectin‐1 demonstrated essential functionality for mediating commensal fungi‐induced biological effects. It provides that molecular mechanistic insights into the mycobiome contribute to radiotherapeutic response modulation [686], further establishing fungal communities as critical factors in tumor radiotherapy. In summary, fungal components significantly influence radiotherapeutic response.

#### Effects of radiotherapy on the microbiome

Radiotherapy exerts complex and multifaceted effects on host‐associated microbial ecosystems, with differential impacts observed across diverse anatomical niches and microbial kingdoms. In HNSCC patients, salivary microbial communities undergo substantial compositional restructuring following radiotherapy, characterized by substantial reductions in taxonomic diversity and concurrent enrichment of *Candida* [665]. Similarly, oral microbiome composition undergoes reconfiguration during radiotherapy in OSCC patients. While α‐diversity remains statistically unchanged between pre‐treatment and immediate post‐treatment timepoints, β‐diversity reveals significant divergence at 6 months post‐radiotherapy [441]. Specific species including *Corynebacterium*, *Haemophilus*, *Veillonella*, and *Actinomyces* decrease in OSCC patients, while concurrent enrichment of *Selenomonas* and *Mycoplasma* is observed [441]. Investigations confirm significant dynamics in oral microbial communities during and following OSCC radiotherapy, with notable depletion of *Prevotella melaninogenica* abundance in post‐radiotherapy samples [687]. Conversely, *C. albicans* demonstrates notable expansion following radiotherapy in HNSCC [688].

In gastrointestinal and pelvic malignancies, radiotherapy induces profound perturbations in intestinal microbial ecosystems. Pelvic radiotherapy for gynecological malignancies induces substantial intestinal dysbiosis, characterized by selective depletion of *Bifidobacterium* and *Clostridium*, and remarkable enrichment in *Bacteroides* and *Enterococcus* [660]. A prospective study of PCa patients has found substantial alterations in gut microbiome β‐diversity without corresponding changes in α‐diversity during radiotherapy [689]. Ferreira et al. similarly observed significantly reduced gut microbiota diversity in patients undergoing radiotherapy, characterized by depletion of *Clostridium perfringens* alongside enrichment of *Clostridium IV*, *Roseburia*, and *Phascolarctobacterium*, which may contribute to the pathogenesis of radiation‐induced enteropathy [690].

Notably, radiotherapeutic interventions modulate not only microbial community structure and ecological diversity but also alter the bacterial functional capacity and virulence expression. For example, neoadjuvant radiotherapy can convert inoculated *Pseudomonas aeruginosa* into a highly pathogenic phenotype that destroys anastomotic sites [691]. During HPV‐associated OPC radiotherapy, substantial reductions in microbial α‐diversity with concurrent alterations in specific taxonomic abundances have been demonstrated [692]. These radiation‐induced microbial perturbations reflect both direct effects on host physiology and clinical prognosis of patients.

#### Microbiome‐mediated radiotherapy toxicities

The microbiome represents a critical role in radiation‐induced normal tissue complication pathogenesis and progression. Clinical investigations demonstrate that radiotherapeutic intervention induces significant taxonomic shifts within microbial communities. These alterations are strongly, negatively correlated with homeostatic cytokine expression in the intestinal mucosa, potentially exacerbating radiotherapy‐induced mucosal injury [693]. In cutaneous tissues, radiation‐induced dermatitis demonstrates a marked association with diminished microbial diversity and characteristic taxonomic alterations, particularly elevated *Proteobacteria/Firmicutes* ratios and *pathogenic S. aureus* proliferation [645]. However, probiotic interventions offer novel therapeutic strategies to alleviate radiotherapy‐related tissue complications. Studies confirm that probiotics effectively attenuate multiple radiation‐induced toxicities through downregulating pro‐inflammatory cytokines [680]. Specifically, clinical studies have demonstrated that the prophylactic and therapeutic administration of *Lactobacillus acidophilus* plus *Bifidobacterium* supplements can alleviate radiation‐induced gastrointestinal toxicity symptoms, including diarrhea and abdominal pain. That concurrently improves health‐related quality of life during radiotherapy [680]. In summary, microbial communities represent critical mediators of radiotherapy‐induced toxicities, establishing a crucial theoretical basis for developing novel therapeutic strategies.

#### Microbiome modulation strategies to improve radiotherapy outcomes

Emerging evidence demonstrates that targeted microbiome manipulation strategies hold significant clinical potential in improving radiotherapy outcomes. *Lactobacillus rhamnosus GG* effectively attenuates radiotherapy‐induced epithelial damage by enhancing crypt survival and reducing epithelial cell apoptosis [694]. Further studies show that altering microbiota composition through antibiotic interventions significantly modulates radiotherapy outcomes. For example, preclinical studies showed that antibiotic pretreatment before radiotherapy partially reversed microbiota dysbiosis and accelerated recovery from radiation‐induced intestinal damage [690]. This process is associated with multiple factors, including the TLR4/MyD88/NF‐κB signaling pathway, macrophage polarization, and inflammatory mediator regulation, demonstrating novel mechanisms of microbiota modulation in improving radiotherapy efficacy [695, 696]. Additionally, prophylactic antifungal interventions appear more beneficial, as commensal fungal species may influence the TIME through pattern recognition receptor‐mediated interactions with macrophages and T cells, potentially enhancing radiotherapy efficacy [686].

### Microbiome and immunotherapy

**Figure 7:**
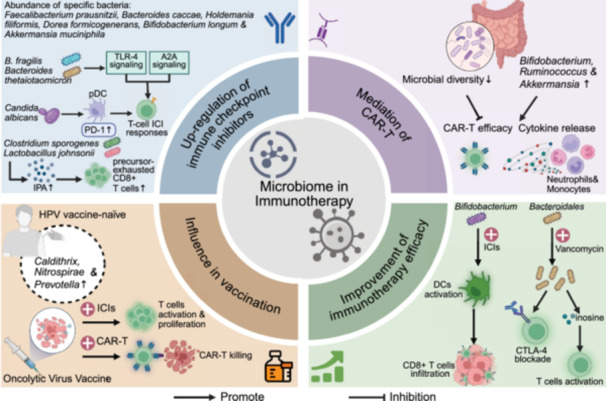
The role of microbiome in immunotherapy. This figure illustrates four key aspects of microbiome‐immunotherapy interactions. Upregulation of immune checkpoint inhibitors (upper left): Specific bacterial species (*Faecalibacterium prausnitzii*, *Bacteroides caccae*, *Holdemania filiformis*, *Dorea formicogenerans*, *Bifidobacterium longum*, and *Akkermansia muciniphila*) modulate immune responses. *B. fragilis* and *Bacteroides thetaiotaomicron* activate TLR4 and A2A receptor signaling pathways. *Candida albicans* influences PD‐1 expression during pDC activation, while *Clostridium sporogenes* and *Lactobacillus johnsonii* enhance precursor‐exhausted CD8^+^ T cell responses via increased IPA production. Mediation of CAR‐T therapy (upper right): Decreased microbial diversity correlates with reduced CAR‐T therapy efficacy. Specifically, *Bifidobacterium*, *Ruminococcus*, and *Akkermansia* populations significantly influence CAR‐T treatment outcomes. Improvement of immunotherapy efficacy (lower right): *Bifidobacterium* species combined with ICIs enhance immune responses through DC activation and increased CD8^+^ T cell infiltration. Additionally, vancomycin treatment enhances CTLA‐4 blockade therapy and T cell activation to show anti‐tumor effects by inducing *Bacteroidales* overexpression with subsequent inosine secretion. Influence in vaccination (lower left): In HPV vaccine‐naïve individuals, specific microbiota (*Caldithrix*, *Nitrospirae*, and *Prevotella*) exhibited enrichment. Additionally, immunotgerapy including ICIs and CAR‐T shows enhanced efficacy when combined with oncolytic virus vaccines, through promoting T cell activation, proliferation, and cytotoxic efficiency. A2A, adenosine 2A; CTLA‐4, cytotoxic T‐lymphocyte‐associated protein 4; DC, dendritic cell; ICIs, immune checkpoint inhibitors; IPA, indole‐3‐propionic acid; pDC, plasmacytoid dendritic cell.

Host‐associated microbial communities represent critical determinants of immunotherapeutic efficacy. They are strongly associated with ICI efficacy and significantly influence CAR‐T cell therapeutic outcomes and cancer vaccine efficacy. Modulation of the microbiome shows therapeutic potential for enhancing immunotherapy efficacy and strengthening anti‐tumor immune responses. Figure 7 summarizes the principal mechanistic pathways through which the microbiome modulates tumor immunotherapeutic outcomes, including bacterial and fungal effects on ICI efficacy, interactions between the microbiome and CAR‐T cell therapy, and microbiome‐mediated effects in vaccine therapy. Collectively, these mechanisms provide a theoretical foundation and practical direction for microbiome modulation strategies to optimize tumor immunotherapy.

#### Microbiome and immune checkpoint inhibitors

Immunomodulatory effects of bacteria: The gut microbiome exerts a significant influence on ICI responses through diverse immunomodulatory pathways, representing a critical focus in contemporary immuno‐oncology research. Studies demonstrate that specific bacterial species, including *Bacteroides thetaiotaomicron*, *B. fragilis*, and *Bifidobacterium*, promote CD8^+^ T cell immune responses through activation of TLR‐4 signaling pathway or adenosine 2A (A2A) receptor signaling pathway, consequently enhancing anti‐tumor immunosurveillance and regulating anti‐tumor immune responses [66]. Intestinal *Lactobacillus johnsonii* abundance demonstrates remarkable positive correlations with ICI efficacy. Specifically, indole‐3‐propionic acid (IPA), synergistically produced through intestinal probiotics *Clostridium sporogenes* and *Lactobacillus johnsonii*, promotes the generation of precursor‐exhausted CD8^+^ T cells, thereby enhancing ICI responses in diverse malignancies including CRC, BC, and melanoma [697].

Clinical investigations suggest intestinal microbial community is strongly associated with anti‐tumor effects across ICI classes [698, 699, 700]. Cohort studies indicate that in advanced melanoma, specific intestinal microbial signatures, characterized by enrichment of *Bifidobacterium pseudocatenulatum*, *Roseburia spp*., and *Akkermansia muciniphila*, demonstrate notable associations with favorable immunotherapeutic response profiles [701]. In patients with metastatic melanoma, intestinal microbiota enriched with *Faecalibacterium* and other *Firmicutes* tend to show enhanced response to ipilimumab treatment [702]. Meanwhile, elevated *Bacteroidetes* phylum demonstrates remarkable negative correlations with ICI‐induced colitis incidence and severity [703]. Additional investigations have confirmed that metastatic melanoma patients exhibiting favorable responses to combined ICIs (nivolumab plus ipilimumab therapy) show fecal enrichment of *Faecalibacterium prausnitzii*, *Bacteroides caccae*, and *Holdemania filiformis* strains, while patients responding to pembrolizumab exhibit selective enrichment of *Dorea formicogenerans* [704].

Preclinical investigations suggest that gut microbiome diversity and composition significantly impact PD‐1 blockade therapy efficacy. Specific strains including *Bifidobacterium longum*, *Akkermansia muciniphila*, and *Faecalibacterium spp*. are strongly associated with enhanced anti‐PD‐1 therapeutic efficacy [681]. In summary, the gut microbiome exerts a significant immunomodulatory influence on ICI therapeutic outcomes through modulation of immune cell functionality and regulation of drug metabolism and effects.

Immunomodulatory effects of fungi: Complex immunoregulatory networks exist between mycobiome components and ICIs, with fungus‐mediated immunomodulation demonstrating particular research value and clinical significance. A study exploring the relationship between the mycobiome and immunotherapy response demonstrated that predictive algorithms based on fungal taxonomic signatures exhibited superior predictive performance (mean AUC of 0.87), outperforming models relying solely on bacterial biomarkers. Further studies found that integration of fungal and bacterial taxonomic signatures into predictive models substantially enhanced discriminatory accuracy (AUC = 0.89), strongly establishing fungi's important regulatory role in ICI responses [705]. Additionally, *C. albicans* colonization induces upregulation of PD‐1 pathways in monocytes and plasmacytoid DCs (pDCs), and also CTLA‐4 pathways in both CD4^+^ and CD8^+^ T cells [706]. These findings provide a scientific foundation for understanding the contribution of the mycobiome to immune checkpoint regulation and ICI efficacy.

#### Microbiome and CAR‐T cell therapy

Emerging clinical evidence establishes the microbiome as a critical factor in CAR‐T cell therapy efficacy [707, 708, 709, 710, 711, 712]. Studies show diminished intestinal microbial diversity indices are strongly and negatively correlated with CAR‐T cell therapeutic response [713]. Specific bacterial taxa abundance, including *Bifidobacterium spp*., *Ruminococcus spp*., and *Akkermansia spp*., demonstrates a remarkable positive association with favorable CAR‐T cell therapeutic outcomes [681]. These findings provide new research trajectories for elucidating microbiome‐mediated regulation of CAR‐T cell therapeutic efficacy. Additionally, OVs as innovative therapeutic vectors demonstrate notable synergistic anti‐tumor activity when strategically combined with ICIs, including CAR‐T cells and anti‐PD‐1/PD‐L1 or anti‐CTLA‐4 [714]. In conclusion, microbial communities significantly influence CAR‐T cell efficacy through multiple immunomodulatory mechanisms, establishing rationale for developing precision microbiome modulation methods as adjunctive strategies to cellular immunotherapy.

#### Microbiome modulation strategies to improve immunotherapy outcomes

As research advances, microbiome modulation strategies exhibit considerable promise for enhancing the efficacy of cancer immunotherapy. Intestinal commensal bacteria exert particularly significant roles in immunotherapy. Studies show *Bifidobacterium* combined with PD‐L1 therapy exhibits significant therapeutic effects on solid tumors through enhancement of DC activity and augmentation of CD8^+^ T cell infiltration [715]. Vancomycin enhances the anti‐tumor effects of CTLA‐4 blockade therapy by inducing the expansion of *Bacteroidales* [716]. Additionally, the metabolite inosine promotes T cell activation in the gut, leading to enhanced efficacy of ICIs [717]. Notably, therapeutic strategies specifically targeting distinct microbial populations may provide new research directions for improving immunotherapy efficacy [171]. In summary, microbiome modulation strategies, including microbiome remodeling through antibiotic treatment, probiotic supplementation, or specific microbe‐targeted therapies, present promising research avenues for improving immunotherapy efficacy and mitigating treatment‐related adverse effects.

#### Microbiome and vaccine therapy

The complex interplay between the host microbiome and cancer vaccine efficacy represents an emerging focus in tumor immunology research. Recent studies investigating HPV therapeutic vaccines reveal associations between cervical microbiome and vaccine‐induced immune responses. Research systematically has analyzed cervical microbiomes of patients with high‐grade squamous intraepithelial lesions (HSIL) before and after administration of HPV therapeutic vaccination. Results showed that five bacterial groups, including *Caldithrix*, *Nitrospirae*, and *Prevotella*, exhibited enrichment in vaccine non‐responders, suggesting these bacterial communities may suppress vaccine‐induced immune responses [718]. These findings provide new insights into the role of the local microbiome in immune responses in patients with HSIL. Notably, enrichment of *Prevotella* may promote persistent HPV infection, thereby potentially conferring resistance to therapeutic vaccination [718]. Furthermore, OVs can be combined with anti‐tumor vaccines to synergistically enhance therapeutic efficacy [714]. In summary, the regulatory mechanisms by which the microbiome influences vaccine immunotherapy and their potential clinical application value require further in‐depth study.

### Microbiome and targeted therapies

The microbiome engages in complex bidirectional interactions with targeted anti‐tumor therapeutics. It influences the efficacy of targeted agents through various mechanisms. Concurrently, alterations in microbiome composition are associated with the development of resistance to targeted therapies, with specific microbial species potentially contributing to resistance. Integration of microbiome‐modulating interventions with targeted therapeutic strategies represents innovative approaches for cancer treatment.

#### Mechanisms in microbiome influence the efficacy of targeted drugs

The microbiome influences the efficacy of targeted drug therapeutic through multiple pathways, with bacterial‐mediated molecular mechanisms emerging as particularly significant mediators of treatment response. Studies indicate that in CRC cells, *Leuconostoc mesenteroides* regulates the NF‐κB/AKT/PTEN/MAPK signaling pathway to induce apoptosis [719], while *P. gingivalis* activates the MAPK signaling pathway to promote tumor cell proliferation [720]. BRAF mutations activate the MAPK pathway and trigger oncogenic effects, which can be effectively suppressed by BRAF and MEK inhibitors [721]. These findings demonstrate that microorganisms can affect tumor proliferation and survival via directly modulating cancer cell signaling networks, potentially altering the efficacy of therapeutic agents that target these signaling pathways.

#### Microbiome and targeted therapy resistance

The microbiome appears to play a significant role in the development of resistance to targeted anti‐tumor therapies. Accumulating evidence demonstrates *F. nucleatum* can activate the E‐cadherin/β‐catenin signaling pathway [156], and the β‐catenin signaling pathway is related to the development of resistance to lenvatinib in HCC [168, 169, 170]. Therefore, the modulation of β‐catenin expression by *F. nucleatum* may affect the sensitivity of tumor cells to lenvatinib. Additionally, NaB, a metabolite produced by specific gut microbes including *Roseburia cecical* and *Roseburia intestinalis*, inhibits expression of sorafenib‐targeted miR‐7641 and miR‐199. A combination approach using NaB with anti‐miR‐7641 or anti‐miR‐199 enhances apoptotic signaling and reduces the survival of drug‐resistant cells [722]. These findings suggest modulating the composition and metabolites of gut microbiota may provide novel intervention strategies to overcome resistance to targeted anti‐tumor agents.

#### Targeted therapeutic strategies for combined microbiome interventions

Research advances in integrating microbiome‐modulating interventions with targeted therapeutic strategies have established promising new avenues for cancer treatment. Studies show microbial metabolite butyrate significantly enhances the therapeutic efficacy of sorafenib in HCC, though its lack of target specificity limits clinical translation. To overcome this limitation, researchers developed nanoparticles (NPs) encapsulating both butyrate and sorafenib, significantly enhancing HCC therapeutic efficacy [723]. Researchers are intensively exploring strategies to optimize targeted therapy efficacy through gut microbiota modulation [724]. Hahn et al. found antibiotics targeting *Bacteroides spp*. modulation improved PFS in patients treated with VEGF‐TKI [725]. Conversely, FMT significantly alleviates diarrhea in metastatic RCC patients treated with TKIs, while simultaneously promoting favorable alterations in gut microbiome composition [726]. This suggests while antibiotics and microbiome modulation offer promising strategies for improving targeted therapeutic response, specific microbiota intervention strategies require careful selection to minimize potential adverse effects.

### Microbiome and surgery

The microbiome influences the outcome and prognosis of surgical treatment of tumors. Strategic preoperative microbiome modulation through targeted antimicrobial therapy or evidence‐based probiotic administration may enhance therapeutic efficacy and substantially reduce the incidence of postoperative complications. Additionally, surgical stress affects microbial homeostasis, while postoperative complications are associated with microbiome composition and metabolism. Therefore, microbiome modulation strategies may accelerate postoperative recovery, mitigate perioperative complications, and optimize outcomes following surgery.

#### Preoperative microbiome preparation

Targeted microbiome modulation approaches in colorectal surgery, particularly preoperative microbiome optimization protocols, significantly influence long‐term surgical prognosis. Numerous studies demonstrate colorectal anastomotic leak (AL) occurrence is associated with reduction in intestinal microbial diversity, whereas oral antibiotic preparations in elective colorectal surgery significantly reduce the incidence of postoperative complications, particularly AL risk [727, 728]. These findings underscore the critical importance of maintaining gut microbiota homeostasis in preventing postoperative complications. Probiotic supplementation demonstrates promise as a prophylactic measure in preoperative microbiome modulation. Studies demonstrate probiotic interventions effectively reduce inflammatory factor levels, attenuate chemotherapeutic side effects, prevent severe diarrhea, reduce postoperative infectious complication incidence, and shorten antibiotic treatment cycles [729]. Accumulating evidence suggests that preoperative probiotic or synbiotic interventions effectively improve surgical outcomes in patients with CRC. Additionally, preoperative mechanical bowel preparation (MBP) combined with antibiotics has garnered substantial clinical interest. Large‐scale retrospective analyses have demonstrated that MBP with oral antibiotics significantly reduces the incidence of postoperative complications, including surgical site infection (SSI), bowel obstruction, and AL [730]. These findings further substantiate preoperative microbiome optimization strategies, particularly evidence‐based probiotic interventions, in improving clinical prognosis following colorectal surgery.

#### Effect of surgical stress on the microbiome

Surgical stress effects on body microbiome represent key research issues in microbiome and surgical therapy. Studies show significantly reduced diversity in fecal microbiota of postoperative CRC patients, with significant compositional differences compared to healthy controls and preoperative CRC patients [731]. Preclinical animal models have confirmed significantly altered gut microbial composition following small bowel surgery, with marked reductions in the relative abundance of *Bacteroidetes* and *Proteobacteria* phyla [732]. Further studies systematically evaluating the effects of surgical resection and chemotherapy on intestinal microbiota in CRC patients have demonstrated significant decreases in obligate anaerobes, tumor‐associated microbial signatures, and butyric acid‐producing bacteria, with significant increases in opportunistic pathogens when comparing postoperative versus preoperative fecal samples [733]. Additionally, hepatectomy can induce gut microbial dysbiosis in HBV‐associated HCC patients, with elevated abundance of *Klebsiella* emerging as a potential predictive biomarker for postoperative liver failure (PHLF) in this patient population [734]. In conclusion, surgical stress modulating effects on microbiome deserve an in‐depth study and attention.

#### Postoperative complications and the microbiome

Current evidence reveals that the complex associations between postoperative complications following oncologic surgery and the patient microbiome are receiving increasing investigative attention. AL represents one of the most common life‐threatening postoperative complications in patients treated with gastrointestinal resection with primary anastomosis, with its pathogenesis strongly linked to gut microbiome regulation. Studies find gut microbiome influences AL development through two distinct molecular mechanisms. First, specific gut bacteria, notably *Enterococcus faecalis* and *Pseudomonas aeruginosa*, dissolve collagen and activate matrix metalloproteinases, potentially contributing to AL initiation and progression. Second, bacterial communities demonstrate protective effects on maintaining anastomotic integrity and promoting tissue healing [653]. Experimental studies have confirmed *Pseudomonas aeruginosa* inoculation significantly increased AL incidence following radiotherapy combined with low colonic anastomosis construction [691]. Additionally, patients developing postoperative pulmonary infections following GC surgical treatment exhibited significant alterations in the functional gene profiles of gut microbes, with enrichment of potentially pathogenic genera including *Klebsiella* and *Enterobacter* [735]. In summary, complex regulatory networks exist between the microbiome and postoperative complications, and comprehensive elucidation of these mechanistic interactions holds significance for improving patient prognosis.

#### Microbiome modulation for postoperative recovery

Microbiome modulation may play a fundamental role in optimizing postoperative recovery. Clinical studies confirm that high‐fat, high‐cholesterol (HFHC) dietary patterns significantly exacerbate gut microbiota dysbiosis and subsequently induce intestinal inflammatory responses in post‐cholecystectomy patients, providing a mechanistic rationale for targeted probiotic intervention strategies to restore microbiome homeostasis and accelerate postoperative recovery [736]. Specifically, gut microbiota significantly regulates surgical incision healing. Studies show gut microbiota stimulates vagus nerve‐mediated oxytocin release through lactic acid fermentation, subsequently promoting T cell recruitment and accelerating incision‐healing processes [737]. Microbiome modulation represents an important therapeutic avenue for enhancing postoperative recovery, and patients may benefit from more optimized post‐surgical care protocols through evidence‐based nutritional intervention, strain‐specific probiotic supplementation, and precision microbiome‐targeted therapies.

### Microbiome‐based strategies for tumor therapy

**Figure 8:**
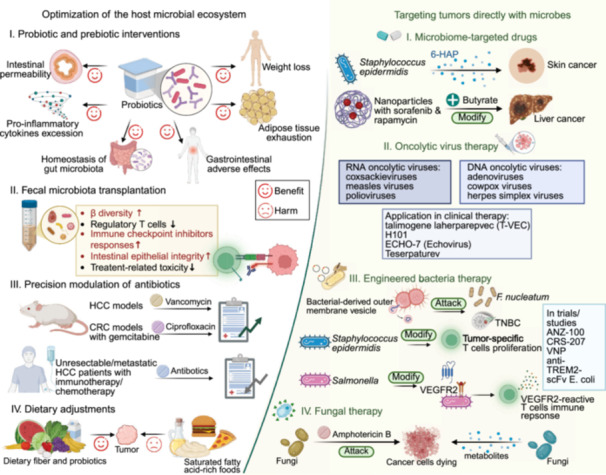
Therapeutic strategies for microbiome modulation in cancer treatment. On the one hand, optimize the host microbial ecosystem (Left). (I). Probiotic and prebiotic interventions show the beneficial effects on intestinal permeability, pro‐inflammatory cytokine expression, gut microbiota homeostasis, weight loss, adipose tissue exhaustion, and gastrointestinal adverse effects. (II). FMT shows outcomes including increased β‐diversity, decreased regulatory T cells (Tregs), enhanced ICI responses, improved intestinal epithelial integrity, and reduced treatment‐related toxicity. (III). Precision antibiotic interventions (vancomycin, ciprofloxacin) in HCC and CRC mouse models improve prognosis, while antibiotics for unresectable/metastatic HCC patients receiving immunotherapy/chemotherapy show the converse affects. (IV). Dietary options represent the key modifier in microbiome‐mediated tumor treatment. Dietary fiber and probiotics serve as protectors, whereas the role of saturated fatty acid‐rich foods is strongly associated with tumorigenesis and microbial dysbiosis. Alternatively, targeting tumors directly with microbes (Right). (I). Microbiome‐targeted drug development provides new therapeutic directions. *S. epidermidis* produces 6‐HAP with demonstrated efficacy against skin cancer, while engineered nanoparticles containing sorafenib/rapamycin facilitate delivery of microbial metabolites (butyrate) for liver cancer therapy. (II). Oncolytic virus therapy, including RNA oncolytic viruses (coxsackieviruses, measles viruses, polioviruses), DNA oncolytic viruses (adenoviruses, cowpox viruses, herpes simplex viruses), and particularly clinical applications (T‐VEC, H101, ECHO‐7, and Teserpaturev), shows potential for improvement in anti‐tumor therapeutic strategies. (III). Engineered bacteria therapy involves bacterial‐derived outer membrane vesicles, which can target *F. nucleatum* and attack triple‐negative breast cancer cells. *S. epidermidis* promotes tumor‐specific T cell proliferation. Engineered bacteria, including *Salmonella typhimurium* expressing vascular endothelial growth factor receptor 2 (VEGFR2)^+^, and *Listeria monocytogenes* attenuated live vaccine (ANZ‐100, CRS‐207) have progressed to Phase I trials. Additional bacterial candidates under investigation, including facultative anaerobic *Salmonella typhimurium (VNP)* conjuncted with CaCO3 and *E. coli* expressing anti‐TREM2 single‐chain antibody fragments (scFv), exert anti‐cancer activity and promote tumor radioimmunotherapy. (IV). Fungi‐based therapeutic approaches, when combined with amphotericin B or specific fungal‐derived metabolites, can induce cancer cell death. 1scFv, single‐chain antibody fragments; 6‐HAP, 6‐N‐hydroxylaminopurine; FMT, Fecal microbiota transplantation; VEGFR2, vascular endothelial growth factor receptor 2.

Microbiome‐targeted therapeutic approaches in oncology are being systematically investigated across multiple experimental and clinical platforms with accelerating momentum. Current microbial intervention approaches include probiotic, prebiotic and synbiotic interventions, FMT, precision antibiotic modulation, dietary modification, microbiome‐targeted drugs, OVs therapy, engineered bacterial therapy, and fungal therapeutic strategies. These options significantly expand the therapeutic landscape and offer promising avenues for improving therapeutic outcomes. Figure 8 provides a comprehensive mechanistic framework illustrating dual approaches for optimization of host microbiome and targeted methods with microbes.

#### Probiotic, prebiotic, and synbiotic interventions

Probiotics, prebiotics, and synbiotics, as established modulators of gut microbial ecology, play fundamental roles in maintaining intestinal microecological homeostasis. Specifically, probiotics are defined as live microorganisms, primarily *Bifidobacterium* and *Lactobacillus*. Prebiotics are non‐digestible substrates that selectively promote the growth and metabolic activity of beneficial microbial communities. Synbiotics represent strategic combinations of probiotics and prebiotics. With advancing mechanistic understanding of host–microbiome interactions, probiotic interventions demonstrate significant therapeutic potential in cancer treatment paradigms, particularly in enhancing anti‐tumor efficacy and mitigating treatment‐associated adverse effects. Preclinical investigations have confirmed that oral multi‐strain probiotic preparations significantly improve chemotherapy‐related symptoms, including weight loss, adipose tissue depletion, and increased intestinal permeability, while effectively reducing pro‐inflammatory cytokine levels. These implications further demonstrate the therapeutic potential of targeted probiotic interventions in alleviating chemotherapy‐related adverse effects [738]. Diverse probiotic strains, predominantly within the *Bifidobacterium* and *Lactobacillus* genera, exhibit anti‐tumor effects through multiple mechanisms, including enhancement of anti‐tumor immune responses and inhibition of tumor invasion [715, 739]. In the context of chemotherapeutic potentiation, probiotics enhance chemotherapeutic efficacy and reduce toxicity through SCFAs production, which is further enhanced through the synergistic application of prebiotics [677]. Specific *Lactobacillus* strains modulate intestinal microbiome homeostasis and metabolite profiles through dual mechanisms, favorably remodeling the TME, collectively demonstrating significant potential for suppressing CRC progression [740]. In PC models, *Lactobacillus* specifically regulates intestinal microbial homeostasis through inhibition of TLR4 signaling pathway, potentially effectively inhibiting PC progression [48]. In IBD patients, probiotics like *Lactobacillus plantarum* and *Lactococcus lactis* are proposed as potential therapeutic agents for both IBD management and associated CRC prevention through modulating intestinal microbial composition and rebalancing dysregulated mucosal immune responses [326]. In CRC prevention and treatment, probiotic/synbiotic interventions significantly reduce aberrant crypt foci formation while promoting the restoration of beneficial microbes, enhancing SCFA production, and modulating expression of key inflammatory mediators [741]. In patients with advanced lung cancer receiving platinum‐based combination chemotherapy regimens, clinical studies show probiotic complexes significantly attenuate chemotherapy‐related gastrointestinal adverse effects [742]. However, context‐dependent effects have been observed wherein *Lactobacillus* may exert potential pro‐tumorigenic activities by increasing lactic acid concentrations of the TME and subsequently suppressing anti‐tumor immune responses. This suggests that it is critical to consider the TME complexity when developing therapeutic probiotic interventions [743]. In RCC patients receiving ICIs, probiotic preparation of CBM588 containing *Clostridium butyricum* has demonstrated strong correlations with enhanced therapeutic responses through promoting proliferation of *Bifidobacterium spp*. and facilitating acetate and butyrate metabolism [744]. Additionally, probiotic spores (spores‐dex) specifically demonstrate selective accumulation within colon cancer tissues, exerting anti‐tumor effects through local production of SCFA and modulation of gut microbiota composition, collectively, significantly inhibiting tumor growth [238]. In conclusion, the strategically designed probiotic intervention represents a cornerstone of microbiome‐targeted therapeutic approaches, showing considerable promise across multiple clinical applications. However, the mechanistic complexity and context‐dependent efficacy of these interventions underscore the importance of rigorous investigation.

#### Fecal microbiota transplantation

FMT exhibits significant therapeutic potential across diverse malignancies [745, 746, 747, 748, 749]. In a primary HCC murine model, FMT significantly altered intestinal microbial β‐diversity while concurrently reducing Treg density, indicating FMT efficacy in modulating gut microbiota and immune microenvironment [750]. Subsequent investigations revealed FMT potentially enhances clinical outcomes following ICI administration in advanced or metastatic CRC treatment, particularly among CRC patients with poor immunotherapy response [751]. Mechanistically, FMT combined with lactic acid‐producing bacteria and their metabolic products (post‐probiotics) synergistically restructures intestinal microbial ecosystems and modulates the TIME, conferring both prophylactic and therapeutic benefits against CRC [7]. Interestingly, FMT from ICI‐responsive cancer patients significantly enhanced anti‐PD‐1 efficacy when administered to germ‐free or antibiotic‐treated murine models [24]. Additionally, FMT efficacy in the treatment of recurrent *Clostridium difficile* infections (CDI), coupled with its capacity to enhance survival, restore gastrointestinal function, and preserve intestinal epithelial barrier integrity in radiation‐exposed animal models, supports its therapeutic potential to improve clinical outcomes in patients with cancer [752]. In summary, FMT represents a central component of microbiome‐targeted therapeutic strategies with the potential to modulate the gut microbiota, enhance immunotherapeutic responses, and mitigate treatment‐associated toxicities.

Multiple clinical investigations are systematically evaluating FMT efficacy across diverse oncological contexts [753]. Randomized controlled trials show significantly higher rates of TKI‐induced diarrhea symptom relief in healthy donors treated with FMT compared to vehicle‐only controls [726]. A recent study suggests this process correlates with enhanced mucosa‐associated invariant T cell functionality [754]. A retrospective case series demonstrated that for cancer patients receiving chemotherapy who developed CDI, the majority of them experienced significant improvement without recurrence following FMT administration, supporting FMT as an effective and well‐tolerated intervention for chemotherapy‐associated complications [755]. Similarly, a recent study analyzing patients with ICI‐induced refractory immune‐mediated colitis found 83% achieved remission following FMT treatment [756]. A clinical trial exploring FMT to overcome ICI resistance in melanoma utilized fecal samples from melanoma patients exhibiting partial or complete ICI responses as donors. They have observed a 30% objective response rate in 10 melanoma patients receiving FMT combined with ICI therapy, a substantial improvement compared to ICI rechallenge response rates (<10%) [757]. Another study conducted a similar trial combining FMT with pembrolizumab in 15 immunotherapy‐refractory melanoma patients, documenting clinical benefit in three patients and disease control exceeding 1 year in three patients [758]. Administration of FMT derived from healthy donors to 20 melanoma patients concurrently receiving anti‐PD‐1 therapy yielded a 65% objective response rate [759]. For urothelial and prostate cancer patients developing colitis after ICI treatment, FMT from healthy donors effectively restructured intestinal microbial communities, resulting in rapid and significant improvement of refractory immune‐related colitis [760]. These clinical observations suggest that FMT holds promise for improving remission rates and reducing treatment‐associated adverse events.

#### Antibiotic regulation

Antibiotic‐mediated microbiome modulation represents a critical focus of microbiome‐directed tumor intervention strategies [761, 762, 763]. Vancomycin treatment significantly depletes intestinal *Clostridium cluster XIVa* populations in HCC murine models, consequently reducing circulating secondary bile acid concentrations and attenuating hepatocarcinogenesis. This finding elucidates a mechanistic pathway through which antibiotics influence oncogenesis via alterations of microbial metabolite profiles [764]. Notably, combining antimicrobial agents with chemotherapy demonstrates significant synergistic effects. Preclinical investigations utilizing colon cancer murine models demonstrate administration of gemcitabine and ciprofloxacin elicits significantly enhanced therapeutic responses compared to gemcitabine monotherapy. That suggests antibiotic‐mediated microbiome modulation may enhance chemotherapeutic efficacy [642]. However, antimicrobial interventions necessitate judicious implementation, as perturbations in intestinal microbial ecosystems may significantly influence immunotherapeutic outcomes. A retrospective analysis of clinical trial data of unresectable/metastatic HCC patients treated with ICIs demonstrated that early antimicrobial exposure significantly compromised ICIs' efficacy. The phenomenon is potentially due to disruption of microbiota communities [765]. Administration of anti‐anaerobic antimicrobial agents correlates with poor prognosis in HCC patients undergoing chemotherapy, whereas intestinal enrichment with anaerobic *Blautia* demonstrates a significant association with favorable outcomes. Consequently, rigorous antimicrobial stewardship protocols require implementation for agents targeting anaerobic microbial communities [766].

#### Dietary regulation

Dietary modification represents a key modifier in microbiome‐mediated oncological therapeutic approaches [767, 768, 769, 770]. In advanced melanoma patients receiving ICI therapy, adherence to the Mediterranean diet is positively correlated with immunotherapy efficacy [761]. Comprehensive analyses utilizing 3‐day, 24‐h dietary recall methodologies in lung cancer patients have established significant associations among dietary intake patterns, intestinal microbial diversity indices, and multiple clinical health parameters [772]. Dietary fiber, a critical nutritional component, exerts multiple biological functions in maintaining host homeostasis. It serves as a substrate for intestinal microbial fermentation, subsequently generating bioactive metabolites, including SCFAs and bile acids, and significantly influencing carcinogenesis [773]. Substantial evidence demonstrates dietary fiber has significant chemopreventive and therapeutic effects in preventing and intervening in CRC, including restoration of microbial community diversity, elevation of SCFA levels, and suppression of EMT [774]. Furthermore, strategic dietary interventions synergize with circadian regulatory mechanisms to maintain physiological homeostasis. Time‐restricted feeding (TRF), a chronobiologically aligned nutritional intervention, preserves circadian rhythm synchronization, ameliorates metabolic disorders, and significantly delays lung tumor growth. TRF exerts anti‐tumor effects through restructuring intestinal microbial communities, particularly increasing *Lactobacillus* and *Bacillus* abundance, and influencing immune and inflammatory processes [775]. Notably, different dietary components variably affect the gut microbiome. Dietary regimens incorporating sorghum, allium species, and cruciferous vegetables demonstrate inverse associations with risk of BT, which is mediated through circulating extracellular vesicle (EV) associated microbial signatures. However, consumption of specific plant‐derived foods, including tumbleweeds and pyrus fruits, potentially enhances oncogenic risk [626]. Furthermore, comparative analysis of dietary lipid models demonstrates that saturated fatty acids and cocoa butter exhibit significantly greater pro‐tumorigenic effects compared to monounsaturated (olive oil) or polyunsaturated (corn oil) fatty acid sources [776].

#### Microbiome‐targeted drugs

Within the expanding domain of microbiome‐directed therapeutic approaches, the development of agents specifically targeting microbial communities provides new therapeutic ideas and directions for cancer treatment. Studies show 6‐HAP secreted by *S. epidermidis* specifically inhibits cancer cells, establishing novel therapeutic strategies for cutaneous malignancies [420]. NPs functionalized with butyrate and co‐encapsulating sorafenib and rapamycin demonstrate significantly enhanced therapeutic efficacy in HCC [777]. Researchers increasingly emphasize the critical contributions of intestinal mycobiome components to hepatocarcinogenesis. Specific fungi demonstrate capacity for translocation from intestinal compartments to liver, closely related to HCC development, suggesting fungal genomes represent promising therapeutic targets [723]. Additionally, microbiome‐targeted therapies demonstrate significant synergistic interactions with chemotherapeutic agents, potentiating chemotherapeutic efficacy through modulation of drug metabolism [642]. Notably, precision therapeutic strategies against specific microorganisms like *F. nucleatum* show promising results for BC patients, especially when combined with immunotherapy and autophagy inhibitors [171]. In summary, microbiome‐targeted drug development may represent a significant potential to revolutionize contemporary oncological management.

#### Oncolytic virus therapy

Among microbiome‐mediated tumor therapy strategies, OV therapy (OVT) exhibits substantial anti‐tumor potential as an emerging therapeutic modality [778, 779, 780, 781, 782, 783]. OVs derive predominantly from attenuated human pathogens, vaccine vector platforms, and genetically modified replication‐selective viral constructs. RNA virus‐derived oncolytic agents include coxsackieviruses, measles viruses, and polioviruses represent typical OVs, while DNA virus‐derived therapeutic vectors encompass adenoviruses, vaccinia viruses, and herpes simplex viruses [784]. Each possesses a distinct clinical application. OVs mediate therapeutic effects through the induction of immunogenic cell death and modulation of host anti‐tumor immunity, achieving direct oncolysis while preserving non‐malignant tissues [785, 786]. Currently, four OV types have received regulatory approval for clinical oncological applications, including talimogene laherparepvec (T‐VEC), H101, ECHO‐7 (Echovirus), and Teserpaturev [785]. OVT demonstrates promising efficacy across diverse malignancies, yet faces challenges including virus leakage, safety hazards of accidental transmission, strict transportation and storage requirements, and necessity for specialized delivery systems [786, 787]. Therefore, developing safer and more effective OVT strategies represents a critical priority for advancing clinical implementation of its clinical translation.

#### Engineered bacteria therapy

Within the expanding arsenal of microbiome‐directed therapeutic modalities, engineered bacterial platforms represent a transformative approach with exceptional therapeutic potential. Engineered bacteria can be precisely modified to selectively colonize neoplastic tissues and functionalized as delivery vehicles for chemotherapeutic payloads, significantly enhancing chemotherapy efficacy [788]. These engineered microorganisms apply not only independently but also synergistically with other anti‐tumor therapies, serving as diagnostic signals and preventive vaccines, demonstrating remarkable multifunctionality [789]. Various bacteria have been engineered as therapeutic agents, with *Listeria monocytogenes* and *Salmonella enterica* representing the most extensively characterized. Currently, multiple engineered bacteria have progressed to Phase I and II trials, demonstrating favorable results [790]. In a Phase I clinical trial for advanced PC, oral administration of attenuated *Salmonella typhimurium* expressing VEGFR2 increased VEGFR2‐reactive CD4^+^ and CD8^+^ T cell responses and promoted production of IFNγ, TNF, and IL‐2, while significantly reducing tumor invasion [791]. Simultaneously, *Listeria monocytogenes* attenuated live vaccine (ANZ‐100) and *Listeria monocytogenes* attenuated live vaccine expressing tumor differentiation antigen mesothelin (CRS‐207) have completed Phase I clinical trials, demonstrating significant efficacy and good tolerability in patients with mesothelioma, lung cancer, PC, and ovarian cancer [792]. Comprehensive analyses of engineered bacterial projects show their capacity to regulate tumor cell and reprogram immune cell metabolism in the TME [793]. Additionally, engineered *S. epidermidis* induces tumor‐specific T cell production, resulting in significant inhibition of both primary and metastatic melanoma progression [506]. These studies indicate that engineered bacteria are associated with the TIME [794]. Furthermore, engineered bacteria can assist with immunotherapy for tumors [795, 796, 797]. For the specific TME, engineered bacterial therapy functions as a potent immunotherapeutic adjuvant. A recent study shows that engineered *E.coli* precisely expressing anti‐TREM2 single‐chain antibody fragments (scFv) can regulate macrophages to promote tumor radioimmunotherapy [798]. Bioconjugates of engineered bacteria (facultative anaerobic *Salmonella typhimurium* VNP20009, VNP) with CaCO_3_ can precisely target and colonize tumor cells, exerting anti‐cancer activity and helping improve immunotherapeutic efficacy [799]. Targeting the unique physiological and immunological characteristics of the TME, engineered microbial therapeutics facilitate precise and multifaceted immunomodulation. In triple‐negative BC research, a bacterial‐derived outer membrane vesicle (OMV)‐coated nanoplatform demonstrates dual‐targeting capacity against pathogenic *F. nucleatum* and cancer cells, effectively repurposing intratumoral microbial communities as an immunostimulatory adjuvant, providing an innovative therapeutic approach for triple‐negative BC [800]. Engineered bacterial therapies further demonstrate the capacity to maintain oxygen supply, activate chemotherapeutic agents, and enhance tumor radiosensitivity [794].

#### Fungal treatment strategies

Within the expanding landscape of microbiome‐directed oncological interventions, mycobiome‐targeted therapeutic approach emerge as an innovative anti‐tumor paradigm. Emerging evidence demonstrates that modulation of mycobiome composition in PC significantly influences treatment response, positioning antifungal interventions as promising therapeutic strategies for specific PC patients [221]. Additionally, targeted modulation of fungal‐associated immunological mediators, including MBL and IL‐33, provides novel immunotherapeutic strategies in cancer treatment [801]. Preclinical studies demonstrate that combination regimens incorporating antifungal agents with radiotherapy enhance tumor cell apoptosis and prolong survival compared to radiotherapy alone. This emphasizes the potential of mycobiome‐targeted interventions as radiotherapy‐sensitizing strategies [686]. However, the impact of fungal species on oncological outcomes demonstrates significant strain‐specific heterogeneity. For example, in BC murine models, *C. albicans* supplementation combined with radiotherapy accelerated tumor progression and decreased survival intervals, illustrating the critical importance of species‐specific mycobiome effects on therapeutic response [686]. Furthermore, fungal‐derived bioactive metabolites show cancer therapy potential, including impairment of DNA damage repair pathways of malignant cells and direct cytotoxic and genotoxic activities, collectively contributing to their anti‐tumor effects [802]. In conclusion, mycobiome‐directed therapeutic strategies represent an innovative component of the microbiome‐targeted oncological armamentarium, demonstrating notable promise through diverse mechanistic pathways.

## FUTURE PERSPECTIVES AND CONCLUSION

**Figure 9:**
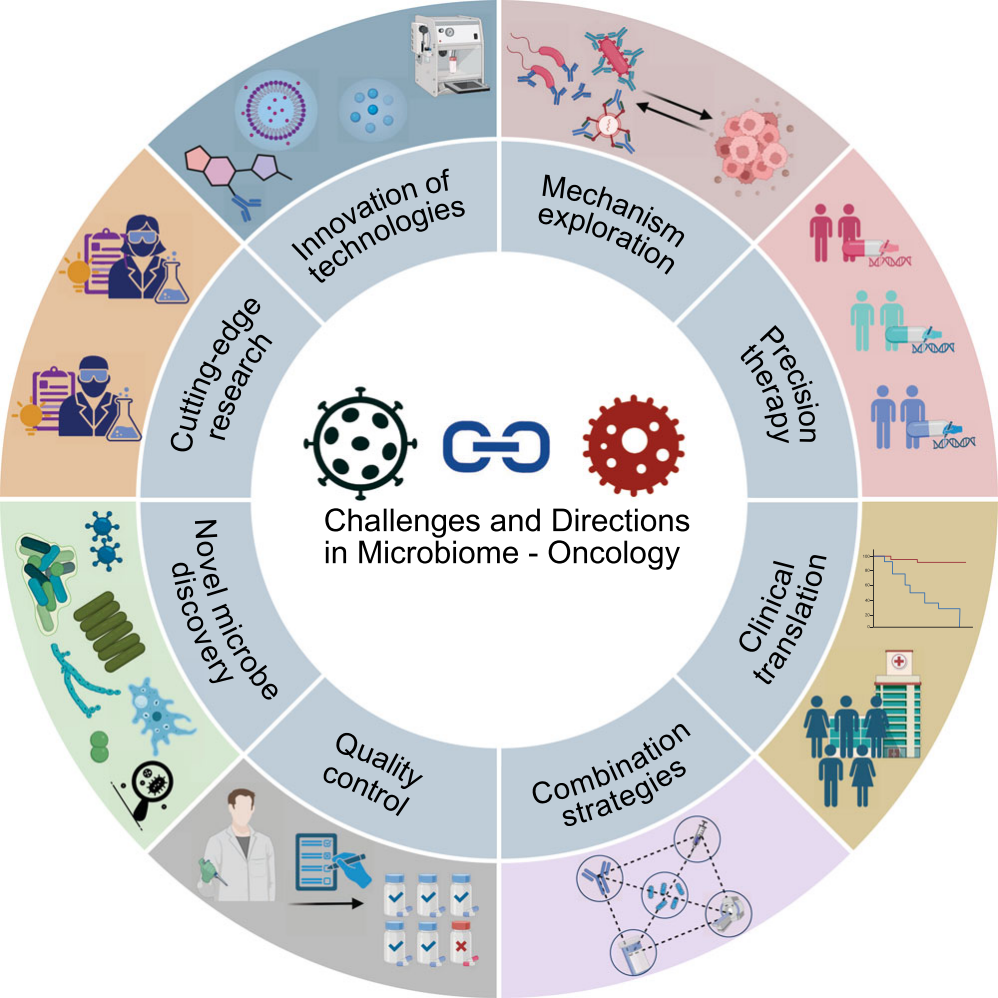
Challenges and future directions of microbiome in the field of precision oncology. This circular diagram illustrates eight key aspects defining the frontier of microbiome‐oncology research: (1) quality control, (2) clinical translation pathways, (3) combination strategies with microbes‐based cancer treatments, (4) personalized therapies based on individual mirobiome profiles, (5) cutting‐edge research developments, (6) new microbial species and their potential application, (7) emerging technological platforms and methodological advances of technologies, and (8) mechanistic insights into biological processes governing microbe‐tumor microenvironment interactions.

The evolving landscape of microbiome science presents unprecedented opportunities coupled with formidable methodological and translational challenges that demand innovative solutions. Microbiome research technologies require continuous refinement and transformative technological innovations. Elucidation of mechanistic pathways mediating microbial influences in oncogenesis, development of precision microbiome‐targeted therapeutic interventions, and acceleration of robust clinical translation collectively advance our comprehensive understanding of host–microbe interactions in malignancy while establishing novel paradigms for cancer management. Optimizing microbiome combination therapy strategies will enhance therapeutic efficacy and expand therapeutic paradigms. As scientific understanding advances, microbiome‐directed therapeutic agents are progressively entering clinical development pipelines, through necessitating rigorous regulatory frameworks that address concerns regarding the safety and efficacy. Integration of emerging research directions, exploration of understudied microbial communities, and characterization of their functional interactions promise notable clinical significance. Figure 9 comprehensively illustrates the major challenges and future directions of microbiome and oncology research, including technological innovations, mechanistic explorations, therapeutic strategy development, clinical translation approaches, and multivariate microbiome research frontiers, providing readers with an integrated framework and strategic perspective on the future development of microbiome and tumor research.

### Developments and innovations in microbiome research technologies

Recent technological innovations in microbiome research are driving unprecedented analytical depth and expanding investigative breadth across oncology. Single‐cell sequencing technology, as a representative emerging methodology, shows unique advantages and broad application prospects in microbiome research. Microbial split‐pool ligation transcriptomics (microSPLiT) enables high‐throughput transcriptional analysis of Gram‐negative and Gram‐positive bacteria, revealing gene expression heterogeneity in *Bacillus subtilis* [801]. Additionally, the innovative Barcoding Bacteria for Identification and Quantification (BarBIQ) method significantly improves 16S rRNA identification accuracy through single‐bacterial barcode labeling [803]. In the spatial dimension, emerging genomic technologies reveal complex microbe–host relationships within tissue microenvironments, with researchers successfully achieving in situ resolution of host–microbe interactions in OSCC and CRC using GeoMx digital spatial profiling (DSP) [804]. At the systemic research level, multi‐omics integration strategies establish new paradigms for studying microbial functions within lung cancer and other tumors by simultaneously capturing the microbiome and host transcriptional responses [349]. With the advent of the big‐data era, artificial intelligence (AI) and machine‐learning applications in microbiome data analysis have become increasingly critical for interpreting complex microbiome data sets. AI demonstrates remarkable potential in predicting host–microbe interactions, resolving uncharacterized metagenomic sequences, and optimizing experimental design [805]. Machine‐learning algorithms excel in pattern recognition for cancer risk prediction and therapeutic response forecasting [806]. In summary, multidimensional innovative developments in microbiome research technologies synergistically drive microbiome research in oncology toward greater precision and comprehensive mechanistic understanding.

Despite significant advances in microbiome research technologies, critical challenges remain to be addressed. The low biomass of intratumoral microbiota presents fundamental challenges in detection sensitivity and specificity, with heightened susceptibility to host DNA contamination and background noise that may generate false‐positive or false‐negative results. Furthermore, existing functional validation methods (such as in vitro co‐culture or murine models) inadequately recapitulate the complex microbe–host interaction networks in humans, while most studies rely on correlation analyses that provide insufficient evidence for causal relationships. For example, the carcinogenic effects of intratumoral microbes are often indirectly verified through gene knockout or antibiotic intervention approaches, but it remains difficult to exclude the influence of off‐target effects or ecological niche disturbances. Future efforts require the development of high‐resolution in situ detection technologies, particularly single‐cell spatial multi‐omics, combined with organoid‐microbe co‐culture systems to enhance both analytical precision and clinical translational value.

### In‐depth exploration of microbiome mechanisms of tumors

Intensified mechanistic investigations have progressively elucidated the complex bidirectional interactions between the microbiome and cancer initiation, progression, and therapeutic response. Microbiome‐mediated metabolic reprogramming has emerged as a focal area of investigation, with recent research deeply elucidating molecular mechanisms underlying the modulation of tumor proliferation and invasion. The immune–tumor–microbiota (IOM) axis framework, recently conceptualized, illustrates the complex regulatory network among the immune system, cancer cells, and microbiota. Systematic resolution of the gut IOM axis has emerged as a research priority, offering novel perspectives on mechanisms by which microbiota influences the TME through immune regulation. In the realm of epigenetic regulation, significant advances have characterized microbiome‐mediated mechanisms, with evidence supporting a bidirectional “epigenome–microbiome axis” [807]. This evidence reveals the critical role of microbiota‐mediated epigenetic modifications in tumorigenesis and development. Additionally, SAHMI technology has successfully identified microbes associated with single cells, revealing critical relationships between the intratumoral microbiome and lung cancer progression that may yield novel diagnostic biomarkers and therapeutic targets [808]. In summary, microbiome mechanism studies involve multiple dimensions, including metabolic reprogramming, immune regulation, epigenetic modifications, and cellular interaction networks within the TME. These mechanistic insights substantially advance our understanding of how diverse microbial communities influence tumorigenesis and tumor progression.

### Development of precision microbiome therapeutic strategies

The development of precision microbiome‐targeted therapeutic strategies has emerged as a significant frontier in addressing diverse malignancies. As an integral component of precision oncology, microbiome analyses in human diseases continue to evolve, providing a reliable scientific foundation for individualized therapeutic strategies [809]. The development of engineered bacterial platforms represents a pivotal technological approach for modulating microbiome‐associated diseases, with therapeutic potential systematically demonstrated in several studies [810, 811]. Evidence indicates that genetically engineered bacteria can function as sophisticated delivery vectors, precisely transporting therapeutic payloads to tumor sites, and significantly enhancing local drug concentrations [812]. Additionally, synthetic biology approaches applied to cancer‐associated microbiomes provide innovative strategies for overcoming treatment resistance, with CRISPR‐based and genome‐engineering technologies demonstrating significant potential for developing novel therapeutic modalities [813]. Therefore, the advancement of precision microbiome‐based cancer therapeutics hinges on complementary approaches, including individualized microbiome intervention protocol development, engineered bacterial development and optimization, microbiome‐targeted delivery system design, and innovative applications of synthetic biology in microbiome therapy. These integrated approaches collectively advance microbiome‐based cancer therapeutics toward improved target specificity and truly personalized interventions.

Among these, bacteriophage‐based therapeutic strategies are garnering significant attention in oncology due to their exceptional target selectivity and molecular affinity. Phages interact with immune cell cytokine networks, participating in the regulation of inflammation and immune tolerance [814], while simultaneously modulating the TME and effectively inhibiting tumor cell growth and metastasis. Based on the tumor‐suppressive functions of phages, numerous studies have screened highly selective and high‐affinity phage vectors combined with bioengineering techniques for cancer treatment. Lei et al. developed a CD40‐targeting engineered M13 phage (H‐GM‐M13CD40) that functions as an in situ vaccine by selectively targeting and activating DCs, inducing DC infiltration within tumor tissues to reverse the immunosuppressive TME [815]. Hou et al. developed a phage vector T4‐Lox‐DNA‐Fe (TLDF) that integrates T4 phage and biointelligent plasmids to disrupt redox homeostasis within the TME, achieving remarkable tumor growth inhibition of up to 78% and establishing a novel approach for precise microenvironmental modulation [816]. Additionally, researchers developed a tumor‐targeting transforming phage/AAV (RGD4C.TPA) vector engineered to deliver TNF‐α genes, which induced selective tumor cell apoptosis and vascular disruption. This approach enhances efficacy when combined with cisplatin, establishing a promising approach against medulloblastoma [817]. M2 macrophage‐specific targeting peptides identified through phage display technology have been engineered to bind with high affinity to both M1 and M2 phenotypes of tumor‐associated macrophages. When combined with complementary genetic modifications, these integrative products enable synergistic elimination of malignant cells while providing a dual‐targeting, light‐controlled therapeutic strategy for precise TME modulation [817]. Phage display technology was recognized with the Nobel Prize in Chemistry in 2018, with increasing research being devoted to phage‐based therapeutic exploration. Mechanistic models centered on phage‐based interventions will be crucial for future research, as systematic exploration of this direction will significantly advance the translational impact of microbial approaches in precision oncology.

### Advancement of clinical translational research

Translational research bridging the microbiome and tumor therapy is rapidly evolving as a critical research direction. Large‐scale multicenter clinical trials not only establish solid data foundations for clinical translational research but also provide strong support for the reliability of research outcomes. For example, a multicenter clinical trial revealed microbiota's potential as a novel therapeutic target in urological malignancies, with implications for developing personalized treatment algorithms [818]. Meanwhile, the development of standardized treatment protocols represents a critical prerequisite for advancing rigorous clinical investigation. Currently, tumor treatment modalities are diverse, including chemotherapy, radiotherapy, immunotherapy, targeted therapy, surgery, and microorganism‐based combination therapy [712, 819, 820, 821, 822, 823, 824, 825, 826]. While this therapeutic diversity expands clinical options, it simultaneously presents substantial methodological challenges for comparative research. In particular, the lack of uniform research standards impedes rigorous cross‐study analysis and meta‐analytical interpretation. The development of standardized treatment protocols will harmonize translational research methodologies and enhance clinical decision‐making efficiency, representing a critical priority for advancing evidence‐based oncological practice. Additionally, existing clinical biomarkers demonstrate inherent limitations despite their established utility. Novel biomarker development and validation have significant value in clinical translation of microbiome research. Particularly, microbiome‐based assessment protocols may provide precise cancer patient stratification by severity, potentially facilitating optimized intervention strategies and improved survival outcomes. For example, identification of conserved microbial biomarkers across ethnically heterogeneous patients enables applicability of optimal diagnostic and prognostic models across diverse populations [50]. Establishment of comprehensive systems for monitoring and preventing adverse effects constitutes an essential component of translational research. Despite continuous refinement of therapeutic modalities, adverse events remain key factors affecting the efficacy and quality of life of patients. Consequently, implementing robust pharmacovigilance systems for adverse effect detection and prevention is paramount for optimizing therapeutic outcomes and safeguarding patient well‐being. In summary, advancement in microbiome‐oncology clinical translational research requires integration of multiple components, including large‐scale multicenter clinical trials, standardized treatment protocol development, biomarker development and validation, and adverse events monitoring and prevention systems. These integrated approaches collectively advance oncological therapeutics toward enhanced precision, efficiency, and safety.

Rigorous clinical validation through well‐designed prospective multicenter trials represents a critical priority, exemplified by protocols that integrate longitudinal microbiome profiling with treatment response assessment in ICI therapy. Such protocols may randomize eligible patients to receive either monotherapy with ICIs or combination therapy with microbiome‐modulating agents, employing standardized techniques for sampling and monitoring changes in microbial marker dynamics. These protocols would incorporate systematic assessment of the TIME parameters, with subsequent analysis of correlations with PFS. Simultaneously, quantitative analysis of microbial metabolites could facilitate the development of integrated diagnostic models incorporating microbial taxa with metabolite signatures, potentially enhancing diagnostic precision and predictive accuracy. In the future, Mendelian randomization methods could further validate causal relationships between the microbiome and clinical outcomes, while elucidating the immunomodulatory mechanisms underlying microbiota‐targeted therapeutic interventions.

### Optimization of microbiome combination therapy strategies

Optimization of microbiome‐targeted combinatorial therapeutic strategies represents a frontier area in contemporary oncological research. In this field, elucidation of synergistic mechanisms between the microbiome and immunotherapy provides a key theoretical foundation for therapeutic development. Numerous studies demonstrate critical associations between the microbiome and immunotherapy, with in‐depth investigation of microbiome‐mediated enhancement of anti‐tumor immunity providing valuable insights for optimizing clinical protocols and developing precisely targeted combination therapies [827, 828]. Design and evaluation of multimodal therapeutic regimens aim to integrate multiple therapeutic modalities, leveraging mechanism‐specific advantages to generate synergistic therapeutic effects. This multimodal treatment strategy integrates various therapeutic approaches, including immunotherapy, chemotherapy, and radiotherapy, while comprehensively accounting for microbiome‐mediated influences on treatment efficacy. Through systematic characterization of patient‐specific microbiome profiles, precisely tailored multimodal therapeutic regimens can be developed, potentially enhancing treatment efficacy while mitigating adverse events. Additionally, elucidation of microbiome‐mediated drug resistance mechanisms represents a critical research priority for overcoming therapeutic resistance and improving treatment efficacy. Studies demonstrate that gut microbes can mediate immunotherapy resistance and accelerate CRC progression [829]. Research has identified intestinal microbe‐mediated drug catabolic pathways and key enzymes, providing novel perspectives into microbiome‐mediated treatment resistance mechanisms [830]. Notably, the development of patient‐specific combinatorial therapeutic approaches constitutes a primary objective in optimizing microbiome‐informed treatment strategies. Integrating microbiome profiles with other biomarkers significantly improves diagnostic precision and facilitates prediction of therapeutic responsiveness, enabling the development of precisely tailored interventional approaches. The gut microbiota shows expansive potential applications in precision medicine and individualized therapy, establishing a solid theoretical foundation for developing personalized combinatorial intervention strategies [809, 831, 832, 833, 834]. In summary, optimization of microbiome‐targeted combinatorial therapeutic strategies requires comprehensive consideration across multiple dimensions, including mechanistic synergies with immunotherapies, multimodal treatment regimen design and evaluation, microbiome‐mediated resistance mechanisms, and individualized combination therapy strategy development, collectively advancing oncology toward maximized treatment efficacy.

### Quality control in microbiome products

As a cutting‐edge biomedical innovation, microbiome‐based therapeutic product development and regulation aim to advance microbiome drug research, development, and clinical application through establishing scientifically standardized processes, stringent quality control standards, comprehensive regulatory frameworks, and systematic ethical guidelines, ultimately facilitating novel precision medicine approaches. Standardization of microbiome‐based therapeutic development processes constitutes the foundational infrastructure for successful product advancement. Integrating gut microbiomics into new drug development systems provides new ideas for resolving challenges of drug development while potentially enhancing therapeutic candidate success rates. Throughout therapeutic development pipelines, enhanced interdisciplinary collaboration between the microbiome and complementary research is essential, necessitating a comprehensive evaluation of candidate compounds' effects on microbiome composition and function while maintaining scientifically rigorous development protocols. Establishment of a quality control system critically determines the safety profile and therapeutic efficacy of microbiome‐based interventions. Pharmaceutical quality management systems should address all critical parameters influencing product quality to ensure consistent compliance with established quality standards and regulatory requirements. For microbiome‐based therapeutic products, quality control strategies must emphasize maintenance of microbial viability, stability, and thorough characterization of interactions with other formulation elements. Robust regulatory frameworks are fundamental to microbiome therapeutic development, encompassing scientifically sound product classification criteria, rigorous approval pathways, and comprehensive post‐marketing surveillance requirements to ensure thorough evaluation of safety and efficacy throughout product lifecycles. Meanwhile, ethical guidelines play a critical role in governing microbiome therapeutic advancement, necessitating thorough consideration of potential risks including microbial ecological dysbiosis and drug resistance. In summary, advancement of microbiome‐based therapeutic products requires coordinated multidimensional approaches, including R&D process standardization, quality control system construction, regulatory framework improvement, and ethical guideline formulation, to ensure product safety, efficacy, and regulatory compliance while facilitating innovation in precision medicine applications.

### Prospects for research direction

Microbiome research represents a frontier area in contemporary biomedical science with profound scientific implications and extensive applications in oncology. Existing studies have confirmed that the microbiome exerts critical regulatory influences in tumorigenesis and tumor development. Investigators propose that identifying microbial markers with cancer‐suppressive properties and developing microbiome‐targeted preventive agents will provide a scientific basis for preventive interventions in high‐risk populations. Intensive studies show that the microbiome has important indicative roles in tumor progression prediction. Specific microbial signatures and compositional alterations correlate significantly with tumor stage, histological grade, and patient outcomes, providing a theoretical basis for developing more refined microbiome‐derived biomarkers. In studies examining microbiome‐mediated remodeling of the TIME, researchers focus on optimizing anti‐tumor immune responses through targeted modulation of gut microbial communities, thereby enhancing immunotherapeutic efficacy. Strategic manipulation of microbial community composition and function can reshape the TIME to enhance immune cell activation and tumor clearance, offering novel approaches to augment cancer immunotherapy. Additionally, rigorous assessment of the long‐term efficacy and durability of microbiome‐targeted interventions represents a critical scientific priority. Considering the complexity and dynamic characteristics of microbiome therapy, establishing comprehensive evaluation frameworks and longitudinal monitoring of microbial community dynamics and host responses is essential for ensuring therapeutic safety and efficacy. Microbiome research shows significant research value and application potential across many oncological contexts. By delineating interactions between the microbiome and tumorigenesis, progression, and treatment response, researchers may achieve more precise approaches to cancer prevention, prediction, therapeutic selection, and outcome assessment, ultimately providing novel strategies to improve patient prognosis.

### Novel research directions for microbiome

Microbiome research, at the forefront of biomedical investigation particularly within oncology, has illuminated complex host–microbe interaction networks and opened expansive research horizons. In studies examining interactions between viral communities and the TME, researchers have thoroughly investigated viral contributions to tumorigenesis, development, and microenvironmental regulation. Future efforts resolving how viral communities interact with tumor cells, immune components, and other microorganisms to shape the TME may provide novel possibilities for exploring virus‐targeted therapeutic opportunities. Studies investigating fungal communities and tumor immune responses focus on how fungal communities regulate immune responses, affecting tumor therapeutic efficacy and clinical prognosis. Research in this area has elucidated associations between fungal diversity and immune evasion, immune cell functionality, and ICI efficacy, providing a mechanistic foundation for mycobiome‐targeted immunotherapeutic approaches. Multidimensional microbiome analyses are emerging as a critical research paradigm that aims to decipher complex ecological networks among diverse microbial communities and host systems by integrating multi‐omic data across bacterial, viral, and fungal domains. This comprehensive cross‐dimensional analysis enables more sophisticated understanding of microbiome contributions to tumorigenesis, tumor progression, and treatment mechanisms, offering novel insights for precision medicine and personalized therapy approaches. Development of new microbiome‐based therapies represents the translational culmination of microbial oncology research. Informed by enhanced mechanistic understanding of microbiome–tumor interactions, interventions targeting specific microbial communities or functions, including probiotics, prebiotics, and bacteriophage therapy, are being developed to regulate anti‐tumor immunity, enhance therapeutic efficacy, mitigate adverse effects, and potentially achieve durable tumor control.

In summary, the human microbiome has emerged as a pivotal regulator in cancer pathogenesis, progression, and therapeutic outcomes. This comprehensive review has synthesized substantial evidence demonstrating that diverse microbial communities influence tumorigenesis through multiple mechanisms, including inflammatory pathways, metabolite production, immune regulatory networks, and epigenetic alterations. Beyond their significant diagnostic potential as biomarkers, microorganisms fundamentally influence therapeutic efficacy and modulate treatment‐associated toxicities. Innovative therapeutic strategies targeting the microbiome, including probiotics, FMT, engineered bacteria, and precision antimicrobial approaches, have shown considerable promise in enhancing the efficacy of conventional cancer treatments and overcoming resistance. Future advances in this rapidly evolving field will require continued technological innovation, deeper mechanistic insights, standardized methodologies, and rigorous clinical validation to fully harness the microbiome's transformative potential in precision oncology and personalized medicine.

## AUTHOR CONTRIBUTIONS

**Anqi Lin:** Writing—original draft; writing—review and editing. **Minying Xiong:** Writing—original draft; writing—review and editing. **Aimin Jiang:** Writing—review and editing. **Lihaoyun Huang:** Writing—review and editing; visualization. **Hank Z. H. Wong:** Writing—review and editing. **Suyin Feng:** Writing—review and editing. **Chunyan Zhang:** Visualization. **Yu Li:** Investigation. **Li Chen:** Investigation. **Hao Chi:** Investigation. **Pengpeng Zhang:** Investigation. **Bicheng Ye:** Investigation. **Hengguo Zhang:** Investigation. **Nan Zhang:** Investigation. **Lingxuan Zhu:** Investigation. **Weiming Mou:** Investigation. **Junyi Shen:** Investigation. **Kailai Li:** Investigation. **Wentao Xu:** Investigation. **Haoxuan Ying:** Investigation. **Cangang Zhang:** Investigation. **Dongqiang Zeng:** Investigation. **Jindong Xie:** Investigation. **Xinpei Deng:** Investigation. **Qi Wang:** Investigation. **Jianying Xu:** Investigation. **Wenjie Shi:** Investigation. **Chang Qi:** Investigation. **Chunrun Qu:** Investigation. **Xufeng Huang:** Investigation. **András Hajdu:** Investigation. **Chaoqun Li:** Investigation. **Changmin Peng:** Investigation. **Xuanye Cao:** Investigation. **Guangsheng Pei:** Investigation. **Lin Zhang:** Investigation. **Yujia Huo:** Investigation. **Jiabao Xu:** Investigation. **Antonino Glaviano:** Investigation. **Attila Gábor Szöllősi:** Investigation. **Sicheng Bian:** Investigation. **Zhengrui Li:** Validation. **Hailin Tang:** Validation. **Bufu Tang:** Validation. **Zaoqu Liu:** Validation. **Jian Zhang:** Validation. **Kai Miao:** Validation. **Quan Cheng:** Conceptualization. **Ting Wei:** Conceptualization. **Shuofeng Yuan:** Conceptualization. **Peng Luo:** Conceptualization.

## CONFLICT OF INTEREST STATEMENT

The authors declare no conflict of interest.

## DATA AVAILABILITY STATEMENT

No new data and scripts were used for this review. Supplementary information (graphical abstract, slides, videos, Chinese translated version, and update materials) is available online DOI or http://www.imeta.science/.

## ETHICS STATEMENT

No animals or humans were involved in this study.

## References

[1] Human Microbiome Project Consortium . 2012. “Structure, Function and Diversity of the Healthy Human Microbiome.” Nature 486: 207–214. 10.1038/nature11234 22699609 PMC3564958

[2] Kuziel, Gavin A. , Seth Rakoff‐Nahoum . 2022. “The Gut Microbiome.” Current Biology 32: R257–R264. 10.1016/j.cub.2022.02.023 35349808

[3] Virtanen, Seppo , Schahzad Saqib , Tinja Kanerva , Rebecka Ventin‐Holmberg , Pekka Nieminen , Tiina Holster , Ilkka Kalliala , Anne Salonen . 2024. “Metagenome‐Validated Combined Amplicon Sequencing and Text Mining‐Based Annotations for Simultaneous Profiling of Bacteria and Fungi: Vaginal Microbiota and Mycobiota in Healthy Women.” Microbiome 12: 273. 10.1186/s40168-024-01993-9 39731160 PMC11681650

[4] Avershina, Ekaterina , Arfa Irej Qureshi , Hanne C. Winther‐Larsen , Trine B. Rounge . 2025. “Challenges in Capturing the Mycobiome From Shotgun Metagenome Data: Lack of Software and Databases.” Microbiome 13: 66. 10.1186/s40168-025-02048-3 40055808 PMC11887097

[5] Rooks, Michelle G. , Wendy S. Garrett . 2016. “Gut Microbiota, Metabolites and Host Immunity.” Nature Reviews Immunology 16: 341–352. 10.1038/nri.2016.42 PMC5541232 27231050

[6] Shea, Allyson E. , Valerie S. Forsyth , Jolie A. Stocki , Taylor J. Mitchell , Arwen E. Frick‐Cheng , Sara N. Smith , Sicily L. Hardy , Harry L. T. Mobley . 2024. “Emerging Roles for ABC Transporters as Virulence Factors in Uropathogenic *Escherichia coli* .” Proceedings of the National Academy of Sciences of the United States of America 121: e2310693121. 10.1073/pnas.2310693121 38607934 PMC11032443

[7] Xu, Yi , Xiahui Wu , Yan Li , Xuejie Liu , Lijian Fang , Ziyu Jiang . 2024. “Probiotics and the Role of Dietary Substrates in Maintaining the Gut Health: Use of Live Microbes and Their Products for Anticancer Effects Against Colorectal Cancer.” Journal of Microbiology and Biotechnology 34: 1933–1946. 10.4014/jmb.2403.03056 39210613 PMC11540615

[8] Montassier, Emmanuel , Rafael Valdés‐Mas , Eric Batard , Niv Zmora , Mally Dori‐Bachash , Jotham Suez , Eran Elinav . 2021. “Probiotics Impact the Antibiotic Resistance Gene Reservoir Along the Human GI Tract in a Person‐Specific and Antibiotic‐Dependent Manner.” Nature Microbiology 6: 1043–1054. 10.1038/s41564-021-00920-0 PMC8318886 34226711

[9] Bajaj, Jasmohan S. , Siew C. Ng , Bernd Schnabl . 2022. “Promises of Microbiome‐Based Therapies.” Journal of Hepatology 76: 1379–1391. 10.1016/j.jhep.2021.12.003 35589257 PMC9588437

[10] Serrano‐Del Valle, Alfonso , Javier Naval , Alberto Anel , Isabel Marzo . 2020. “Novel Forms of Immunomodulation for Cancer Therapy.” Trends in Cancer 6: 518–532. 10.1016/j.trecan.2020.02.015 32460005

[11] Negrón‐Figueroa, Dalissa , Lauren E. Colbert . 2024. “Mechanisms by Which the Intratumoral Microbiome May Potentiate Immunotherapy Response.” Journal of Clinical Oncology 42: 3350–3352. 10.1200/JCO.24.00908 39058969 PMC11427158

[12] Dzutsev, Amiran K. , Romina S. Goldszmid . 2024. “Towards Enhancing the Predictive Value of the Microbiota for Cancer Immunotherapy.” Trends in Cancer 10: 771–773. 10.1016/j.trecan.2024.07.007 39089931

[13] Peppercorn, MARK A. , PETER Goldman . 1972. “The Role of Intestinal Bacteria in the Metabolism of Salicylazosulfapyridine.” The Journal of Pharmacology and Experimental Therapeutics 181: 555–562, 10.1016/S0022-3565(25)29238-2 4402374

[14] Ley, Ruth E. , Peter J. Turnbaugh , Samuel Klein , Jeffrey I. Gordon . 2006. “Human Gut Microbes Associated With Obesity.” Nature 444: 1022–1023. 10.1038/4441022a 17183309

[15] Qin, Youwen , Aki S. Havulinna , Yang Liu , Pekka Jousilahti , Scott C. Ritchie , Alex Tokolyi , Jon G. Sanders , et al.2022. “Combined Effects of Host Genetics and Diet on Human Gut Microbiota and Incident Disease in a Single Population Cohort.” Nature Genetics 54: 134–142. 10.1038/s41588-021-00991-z 35115689 PMC9883041

[16] Nakatsu, Geicho , Natalia Andreeva , Meghan H. MacDonald , Wendy S. Garrett . 2024. “Interactions Between Diet and Gut Microbiota in Cancer.” Nature Microbiology 9: 1644–1654. 10.1038/s41564-024-01736-4 38907007

[17] Lei, Chao , Cong Liu , Yuling Peng , Yu Zhan , Xiaoming Zhang , Ting Liu , Zhihua Liu . 2023. “A High‐Salt Diet Induces Synaptic Loss and Memory Impairment via Gut Microbiota and Butyrate in Mice.” iMeta 2: e97. 10.1002/imt2.97 38868427 PMC10989808

[18] Huang, Yina , Jinxin Liu , Hein Min Tun , Catherine Stanton , Tingtao Chen , Hani El‐Nezami , Hua Wei , Mingfu Wang , Qinglong Wu . 2022. “Gut Microbiota Insights Into Human Adaption to High‐Plateau Diet.” iMeta 1: e6. 10.1002/imt2.6 35989883 PMC9387673

[19] Dapa, Tanja , Ricardo Serotte Ramiro , Miguel Filipe Pedro , Isabel Gordo , Karina Bivar Xavier . 2022. “Diet Leaves a Genetic Signature in a Keystone Member of the Gut Microbiota.” Cell Host & Microbe 30: 183–199.e10. 10.1016/j.chom.2022.01.002 35085504

[20] NIH HMP Working Group , Jane Peterson , Garges, Susan , Maria Giovanni , Pamela McInnes , Lu Wang , Schloss, Jeffery A. , Vivien Bonazzi , McEwen Jean E. , et al. 2009. “The NIH Human Microbiome Project.” Genome Research 19: 2317–2323. 10.1101/gr.096651.109 19819907 PMC2792171

[21] Qin, Junjie , Ruiqiang Li , Jeroen Raes , Manimozhiyan Arumugam , Kristoffer Solvsten Burgdorf , Chaysavanh Manichanh , Trine Nielsen , et al. 2010. “A Human Gut Microbial Gene Catalogue Established by Metagenomic Sequencing.” Nature 464: 59–65. 10.1038/nature08821 20203603 PMC3779803

[22] Yatsunenko, Tanya , Federico E. Rey , Mark J. Manary , Indi Trehan , Maria Gloria Dominguez‐Bello , Monica Contreras , Magda Magris , et al. 2012. “Human Gut Microbiome Viewed Across Age and Geography.” Nature 486: 222–227. 10.1038/nature11053 22699611 PMC3376388

[23] McDonald, Daniel , Embriette Hyde , Justine W. Debelius , James T. Morton , Antonio Gonzalez , Gail Ackermann , Alexander A. Aksenov , et al. 2018. “American Gut: An Open Platform for Citizen Science Microbiome Research.” mSystems 3: e00031‐18. 10.1128/mSystems.00031-18 29795809 PMC5954204

[24] Routy, Bertrand , Emmanuelle Le Chatelier , Lisa Derosa , Connie P. M. Duong , Maryam Tidjani Alou , Romain Daillère , Aurélie Fluckiger , et al. 2018. “Gut Microbiome Influences Efficacy of PD‐1‐Based Immunotherapy Against Epithelial Tumors.” Science 359: 91–97. 10.1126/science.aan3706 29097494

[25] Gagliani, Nicola , Bo Hu , Samuel Huber , Eran Elinav , Richard A. Flavell . 2014. “The Fire Within: Microbes Inflame Tumors.” Cell 157: 776–783. 10.1016/j.cell.2014.03.006 24813605

[26] Nejman, Deborah , Ilana Livyatan , Garold Fuks , Nancy Gavert , Yaara Zwang , Leore T. Geller , Aviva Rotter‐Maskowitz , et al. 2020. “The Human Tumor Microbiome Is Composed of Tumor Type‐Specific Intracellular Bacteria.” Science 368: 973–980. 10.1126/science.aay9189 32467386 PMC7757858

[27] Cogdill, Alexandria P. , Pierre Olivier Gaudreau , Reetakshi Arora , Vancheswaran Gopalakrishnan , Jennifer A. Wargo . 2018. “The Impact of Intratumoral and Gastrointestinal Microbiota on Systemic Cancer Therapy.” Trends in Immunology 39: 900–920. 10.1016/j.it.2018.09.007 30392721

[28] Wong‐Rolle, Abigail , Haohan Karen Wei , Chen Zhao , Chengcheng Jin . 2021. “Unexpected Guests in the Tumor Microenvironment: Microbiome in Cancer.” Protein & Cell 12: 426–435. 10.1007/s13238-020-00813-8 33296049 PMC8106554

[29] Xie, Yifan , Feng Xie , Xiaoxue Zhou , Lei Zhang , Bing Yang , Jun Huang , Fangwei Wang , et al. 2022. “Microbiota in Tumors: From Understanding to Application.” Advanced Science (Weinheim, Baden‐Wurttemberg, Germany) 9: e2200470. 10.1002/advs.202200470 35603968 PMC9313476

[30] Schwabe, Robert F. , Christian Jobin . 2013. “The Microbiome and Cancer.” Nature Reviews Cancer 13: 800–812. 10.1038/nrc3610 24132111 PMC3986062

[31] Castanheira, Cristina Paula , Mayara Luciana Sallas , Rafaella Almeida Lima Nunes , Noely Paula Cristina Lorenzi , Lara Termini . 2021. “Microbiome and Cervical Cancer.” Pathobiology 88: 187–197. 10.1159/000511477 33227782

[32] Cao, Yaqi , Hui Xia , Xueyun Tan , Chunwei Shi , Yanling Ma , Daquan Meng , Mengmeng Zhou , et al. 2024. “Intratumoural Microbiota: A New Frontier in Cancer Development and Therapy.” Signal Transduction and Targeted Therapy 9: 15. 10.1038/s41392-023-01693-0 38195689 PMC10776793

[33] Scott, Alasdair J. , James L. Alexander , Claire A. Merrifield , David Cunningham , Christian Jobin , Robert Brown , John Alverdy , et al. 2019. “International Cancer Microbiome Consortium Consensus Statement on the Role of the Human Microbiome in Carcinogenesis.” Gut 68: 1624–1632. 10.1136/gutjnl-2019-318556 31092590 PMC6709773

[34] Zhao, Lin‐Yong , Jia‐Xin Mei , Gang Yu , Lei Lei , Wei‐Han Zhang , Kai Liu , Xiao‐Long Chen , et al. 2023. “Role of the Gut Microbiota in Anticancer Therapy: From Molecular Mechanisms to Clinical Applications.” Signal Transduction and Targeted Therapy 8: 201. 10.1038/s41392-023-01406-7 37179402 PMC10183032

[35] Yang, Li , Aitian Li , Ying Wang , Yi Zhang . 2023. “Intratumoral Microbiota: Roles in Cancer Initiation, Development and Therapeutic Efficacy.” Signal Transduction and Targeted Therapy 8: 35. 10.1038/s41392-022-01304-4 36646684 PMC9842669

[36] Mou, Weiming , Zhixing Deng , Lingxuan Zhu , Aimin Jiang , Anqi Lin , Liling Xu , Gengwen Deng , et al. 2025 “Intratumoral Mycobiome Heterogeneity Influences the Tumor Microenvironment and Immunotherapy Outcomes in Renal Cell Carcinoma.” Science Advances 11:eadu1727. 10.1126/sciadv.adu1727 40203108 PMC11980860

[37] Li, Lei , Shouhua He , Boyi Liao , Manchun Wang , Huimin Lin , Ben Hu , Xinyue Lan , et al. 2024. “Orally Administrated Hydrogel Harnessing Intratumoral Microbiome and Microbiota‐Related Immune Responses for Potentiated Colorectal Cancer Treatment.” Research (Washington, D.C.) 7: 0364. 10.34133/research.0364 38721274 PMC11077293

[38] Liu, Weici , Zheshun Pi , Ning‐Ning Liu , Wenjun Mao . 2024. “Into the Era of Mycobiome‐Driven Cancer Research.” Trends in Cancer 10: 389–392. 10.1016/j.trecan.2024.02.009 38494372

[39] Soto, David , Christine Song , Margaret E. McLaughlin‐Drubin . 2017. “Epigenetic Alterations in Human Papillomavirus‐Associated Cancers.” Viruses 9: 248. 10.3390/v9090248 28862667 PMC5618014

[40] Amaya Arbeláez , María Isabel , Ana Carolina Alves De Paula E. Silva , Geovana Navegante , Valeria Valente , Paula Aboud Barbugli , Carlos Eduardo Vergani . 2021. “Proto‐Oncogenes and Cell Cycle Gene Expression in Normal and Neoplastic Oral Epithelial Cells Stimulated With Soluble Factors From Single and Dual Biofilms of *Candida albicans* and *Staphylococcus aureus* .” Frontiers in Cellular and Infection Microbiology 11: 627043. 10.3389/fcimb.2021.627043 33718274 PMC7947338

[41] Górska, Agata , Dawid Przystupski , Magdalena J. Niemczura , Julita Kulbacka . 2019. “Probiotic Bacteria: A Promising Tool in Cancer Prevention and Therapy.” Current Microbiology 76: 939–949. 10.1007/s00284-019-01679-8 30949803 PMC6586914

[42] Lili, Zhao , Wei Junyan , Zhao Hongfei , Zhu Baoqing , Zhang Bolin . 2018. “Detoxification of Cancerogenic Compounds by Lactic Acid Bacteria Strains.” Critical Reviews in Food Science and Nutrition 58: 2727–2742. 10.1080/10408398.2017.1339665 29053003

[43] Zhong, Mengya , Yubo Xiong , Jiabao Zhao , Zhi Gao , Jingsong Ma , Zhengxin Wu , Yongxi Song , Xuehui Hong . 2021. “ *Candida albicans* Disorder Is Associated With Gastric Carcinogenesis.” Theranostics 11: 4945–4956. 10.7150/thno.55209 33754037 PMC7978306

[44] Guan, Shi‐Wei , Quan Lin , Xi‐Dong Wu , Hai‐Bo Yu . 2023. “Weighted Gene Coexpression Network Analysis and Machine Learning Reveal Oncogenome Associated Microbiome Plays an Important Role in Tumor Immunity and Prognosis in Pan‐Cancer.” Journal of Translational Medicine 21: 537. 10.1186/s12967-023-04411-0 37573394 PMC10422781

[45] Liu, Le , Liping Liang , YingJie Luo , Jimin Han , Di Lu , RuiJun Cai , Gautam Sethi , Shijie Mai . 2024. “Unveiling the Power of Gut Microbiome in Predicting Neoadjuvant Immunochemotherapy Responses in Esophageal Squamous Cell Carcinoma.” Research (Washington, D.C.) 7: 0529. 10.34133/research.0529 39545038 PMC11562848

[46] Lehouritis, Panos , Joanne Cummins , Michael Stanton , Carola T. Murphy , Florence O. McCarthy , Gregor Reid , Camilla Urbaniak , William L. Byrne , Mark Tangney . 2015. “Local Bacteria Affect the Efficacy of Chemotherapeutic Drugs.” Scientific Reports 5: 14554. 10.1038/srep14554 26416623 PMC4586607

[47] Badgeley, Aja , Hina Anwar , Karan Modi , Paige Murphy , Ashakumary Lakshmikuttyamma . 2021. “Effect of Probiotics and Gut Microbiota on Anti‐Cancer Drugs: Mechanistic Perspectives.” Biochimica et Biophysica Acta (BBA)—Reviews on Cancer 1875: 188494. 10.1016/j.bbcan.2020.188494 33346129

[48] Zhu, Zemin , Bo Yi , Zikai Tang , Xun Chen , Ming Li , Tao Xu , Zhijian Zhao , Caixi Tang . 2023. “ *Lactobacillus casei* Combined With Lactobacillus Reuteri Alleviate Pancreatic Cancer by Inhibiting TLR4 to Promote Macrophage M1 Polarization and Regulate Gut Microbial Homeostasis.” BMC Cancer 23: 1044. 10.1186/s12885-023-11557-z 37904102 PMC10614400

[49] Khoruts, Alexander , Christopher Staley , Michael J. Sadowsky . 2021. “Faecal Microbiota Transplantation for *Clostridioides difficile*: Mechanisms and Pharmacology.” Nature Reviews Gastroenterology & Hepatology 18: 67–80. 10.1038/s41575-020-0350-4 32843743

[50] Dai, Jia‐Hao , Xi‐Rong Tan , Han Qiao , Na Liu . 2024. “Emerging Clinical Relevance of Microbiome in Cancer: Promising Biomarkers and Therapeutic Targets.” Protein & Cell 15: 239–260. 10.1093/procel/pwad052 37946397 PMC10984626

[51] Ting, Nick Lung‐Ngai , Harry Cheuk‐Hay Lau , Jun Yu . 2022. “Cancer Pharmacomicrobiomics: Targeting Microbiota to Optimise Cancer Therapy Outcomes.” Gut 71: 1412–1425. 10.1136/gutjnl-2021-326264 35277453 PMC9185832

[52] Matson, Vyara , Carolina Soto Chervin , Thomas F. Gajewski . 2021. “Cancer and the Microbiome‐Influence of the Commensal Microbiota on Cancer, Immune Responses, and Immunotherapy.” Gastroenterology 160: 600–613. 10.1053/j.gastro.2020.11.041 33253684 PMC8409239

[53] Lee, Mong‐Hong . 2021. “Harness the Functions of Gut Microbiome in Tumorigenesis for Cancer Treatment.” Cancer Communications 41: 937–967. 10.1002/cac2.12200 34355542 PMC8504147

[54] Queen, Jessica , Fyza Shaikh , Cynthia L. Sears . 2023. “Understanding the Mechanisms and Translational Implications of the Microbiome for Cancer Therapy Innovation.” Nature Cancer 4: 1083–1094. 10.1038/s43018-023-00602-2 37525016

[55] Park, Elizabeth M. , Manoj Chelvanambi , Neal Bhutiani , Guido Kroemer , Laurence Zitvogel , Jennifer A. Wargo . 2022. “Targeting the Gut and Tumor Microbiota in Cancer.” Nature Medicine 28: 690–703. 10.1038/s41591-022-01779-2 35440726

[56] Lou, Xinyu , Zhichao Chen , Zhonggui He , Mengchi Sun , Jin Sun . 2021. “Bacteria‐Mediated Synergistic Cancer Therapy: Small Microbiome Has a Big Hope.” Nano‐Micro Letters 13: 37. 10.1007/s40820-020-00560-9 34138211 PMC8187705

[57] Glitza, Isabella C. , Yongwoo David Seo , Christine N. Spencer , Jennifer R. Wortman , Elizabeth M. Burton , Farah A. Alayli , Christopher P. Loo , et al. 2024. “Randomized Placebo‐Controlled, Biomarker‐Stratified Phase Ib Microbiome Modulation in Melanoma: Impact of Antibiotic Preconditioning on Microbiome and Immunity.” Cancer Discovery 14: 1161–1175. 10.1158/2159-8290.CD-24-0066 38588588 PMC11215408

[58] Fernandes, Miriam R. , Poonam Aggarwal , Raquel G. F. Costa , Alicia M. Cole , Giorgio Trinchieri . 2022. “Targeting the Gut Microbiota for Cancer Therapy.” Nature Reviews Cancer 22: 703–722. 10.1038/s41568-022-00513-x 36253536

[59] Bhatt, Aadra P. , Matthew R. Redinbo , Scott J. Bultman . 2017. “The Role of the Microbiome in Cancer Development and Therapy.” CA: A Cancer Journal for Clinicians 67: 326–344. 10.3322/caac.21398 28481406 PMC5530583

[60] Zhang, Xiang , Olabisi Oluwabukola Coker , Eagle Sh Chu , Kaili Fu , Harry C. H. Lau , Yi‐Xiang Wang , Anthony W. H. Chan , et al. 2021. “Dietary Cholesterol Drives Fatty Liver‐Associated Liver Cancer by Modulating Gut Microbiota and Metabolites.” Gut 70: 761–774. 10.1136/gutjnl-2019-319664 32694178 PMC7948195

[61] Wong, Sunny H. , Jun Yu . 2019. “Gut Microbiota in Colorectal Cancer: Mechanisms of Action and Clinical Applications.” Nature Reviews Gastroenterology & Hepatology 16: 690–704. 10.1038/s41575-019-0209-8 31554963

[62] Ohtani, Naoko , Eiji Hara . 2021. “Gut‐Liver Axis‐Mediated Mechanism of Liver Cancer: A Special Focus on the Role of Gut Microbiota.” Cancer Science 112: 4433–4443. 10.1111/cas.15142 34533882 PMC8586687

[63] Cheng, Wing Yin Wu, Chun‐Ying Yu, Jun . 2020. “The Role of Gut Microbiota in Cancer Treatment: Friend or Foe?” Gut 69: 1867–1876. 10.1136/gutjnl-2020-321153 32759302 PMC7497589

[64] Cullin, Nyssa , Camila Azevedo Antunes , Ravid Straussman , Christoph?K. Stein‐Thoeringer , Eran Elinav . 2021. “Microbiome and Cancer.” Cancer Cell 39: 1317–1341. 10.1016/j.ccell.2021.08.006 34506740

[65] Zaidi, Ali H. , Lori A. Kelly , Rachael E. Kreft , Mark Barlek , Ashten N. Omstead , Daisuke Matsui , Natalie H. Boyd , et al. 2016. “Associations of Microbiota and Toll‐Like Receptor Signaling Pathway in Esophageal Adenocarcinoma.” BMC Cancer 16: 52. 10.1186/s12885-016-2093-8 26841926 PMC4739094

[66] Xue, Xiaoyu , Rui Li , Zhenni Chen , Guiyu Li , Bisheng Liu , Shanshan Guo , Qianhua Yue , et al. 2023. “The Role of the Symbiotic Microecosystem in Cancer: Gut Microbiota, Metabolome, and Host Immunome.” Frontiers in Immunology 14: 1235827. 10.3389/fimmu.2023.1235827 37691931 PMC10484231

[67] Jia, Xiaodong , Shanshan Lu , Zhen Zeng , Qingyan Liu , Zheng Dong , Chen, Yan Zhu, Zhenyu , et al. 2020. “Characterization of Gut Microbiota, Bile Acid Metabolism, and Cytokines in Intrahepatic Cholangiocarcinoma.” Hepatology 71: 893–906. 10.1002/hep.30852 31298745

[68] Rubenstein, Joel H. , John M. Inadomi , James Scheiman , Philip Schoenfeld , Henry Appelman , Min Zhang , Val Metko , John Y. Kao . 2014. “Association Between Helicobacter Pylori and Barrett's Esophagus, Erosive Esophagitis, and Gastroesophageal Reflux Symptoms.” Clinical Gastroenterology and Hepatology 12: 239–245. 10.1016/j.cgh.2013.08.029 23988686 PMC3947027

[69] Pei, Jiaxin , Chaoxu Zhang , Qian Zhang , Hao Yu , Huiya Yuan , Yufu Guo , Hui Shen , et al. 2024. “Probiotics Alleviate Chronic Ethanol Exposure‐Induced Anxiety‐Like Behavior and Hippocampal Neuroinflammation in Male Mice Through Gut Microbiota‐Derived Extracellular Vesicles.” Journal of Nanobiotechnology 22: 730. 10.1186/s12951-024-03017-y 39578835 PMC11585232

[70] Lai, Hao , Yunfeng Li , Yafang He , Fangyao Chen , Baibing Mi , Junqi Li , Jiawen Xie , et al. 2023. “Effects of Dietary Fibers or Probiotics on Functional Constipation Symptoms and Roles of Gut Microbiota: A Double‐Blinded Randomized Placebo Trial.” Gut Microbes 15: 2197837. 10.1080/19490976.2023.2197837 37078654 PMC10120550

[71] Khoruts, Alexander , Diane E. Hoffmann , Robert A. Britton . 2020. “Probiotics: Promise, Evidence, and Hope.” Gastroenterology 159: 409–413. 10.1053/j.gastro.2020.05.058 32531290

[72] Van Rossum, Thea , Annette Haiß , Rebecca L. Knoll , Janina Marißen , Daniel Podlesny , Julia Pagel , Marina Bleskina , et al. 2024. “Bifidobacterium and Lactobacillus Probiotics and Gut Dysbiosis in Preterm Infants: The PRIMAL Randomized Clinical Trial.” JAMA Pediatrics 178: 985–995. 10.1001/jamapediatrics.2024.2626 39102225 PMC12549143

[73] Castro‐Mejía, Josué L. , Sinéad O'Ferrall , Łukasz Krych , Elaine O'Mahony , Hanifa Namusoke , Betty Lanyero , Witold Kot , et al. 2020. “Restitution of Gut Microbiota in Ugandan Children Administered With Probiotics (*Lactobacillus rhamnosus GG* and *Bifidobacterium animalis* subsp. lactis BB‐12) During Treatment for Severe Acute Malnutrition.” Gut Microbes 11: 855–867. 10.1080/19490976.2020.1712982 31959047 PMC7524335

[74] Ralser, Anna , Alisa Dietl , Sebastian Jarosch , Veronika Engelsberger , Andreas Wanisch , Klaus Peter Janssen , Moritz Middelhoff , et al. 2023. “ *Helicobacter pylori* Promotes Colorectal Carcinogenesis by Deregulating Intestinal Immunity and Inducing a Mucus‐Degrading Microbiota Signature.” Gut 72: 1258–1270. 10.1136/gutjnl-2022-328075 37015754

[75] Luo, Shiqi , Jinlong Ru , Mohammadali Khan Mirzaei , Jinling Xue , Xue Peng , Anna Ralser , Joshua Lemuel Hadi , et al. 2024. “ *Helicobacter pylori* Infection Alters Gut Virome by Expanding Temperate Phages Linked to Increased Risk of Colorectal Cancer.” Gut 73: 1592–1595. 10.1136/gutjnl-2023-330362 37918887

[76] Butt, Julia , Matthew G. Varga , William J. Blot , Lauren Teras , Kala Visvanathan , Loïc Le Marchand , Christopher Haiman , et al. 2019. “Serologic Response to *Helicobacter pylori* Proteins Associated With Risk of Colorectal Cancer Among Diverse Populations in the United States.” Gastroenterology 156: 175–86.e2. 10.1053/j.gastro.2018.09.054 30296434 PMC6309494

[77] Hansen S. , K. K. Melby , S. Aase , E. Jellum , S.E. Vollset 1999. “ *Helicobacter pylori* Infection and Risk of Cardia Cancer and Non‐Cardia Gastric Cancer. A Nested Case‐Control Study.” Scandinavian Journal of Gastroenterology 34: 353–360. 10.1080/003655299750026353 10365894

[78] Wu, Xiao‐Wei , Hong‐Zan Ji , Miao‐Fang Yang , Lin Wu , Fang‐Yu Wang . 2015. “ *Helicobacter pylori* Infection and Inflammatory Bowel Disease in Asians: A Meta‐Analysis.” World Journal of Gastroenterology 21: 4750–4756. 10.3748/wjg.v21.i15.4750 25914487 PMC4402325

[79] Gutierrez‐Angulo, Melva , Maria de la Luz Ayala‐Madrigal , Jose Miguel Moreno‐Ortiz , Jorge Peregrina‐Sandoval , Fernando Daniel Garcia‐Ayala . 2023. “Microbiota Composition and Its Impact on DNA Methylation in Colorectal Cancer.” Frontiers in Genetics 14: 1037406. 10.3389/fgene.2023.1037406 37614819 PMC10442805

[80] Li, Ting , Zhanyi Zhao , Meichang Peng , Lu Zhang , Cheng Wang , Feiyang Luo , Meiqin Zeng , et al. 2025. “Multi‐Omics Analysis Reveals the Interplay Between Intratumoral Bacteria and Glioma.” mSystems 10: e0045724. 10.1128/msystems.00457-24 39660865 PMC11748541

[81] Gur, Chamutal , Yara Ibrahim , Batya Isaacson , Rachel Yamin , Jawad Abed , Moriya Gamliel , Jonatan Enk , et al. 2015. “Binding of the Fap2 Protein of *Fusobacterium nucleatum* to Human Inhibitory Receptor TIGIT Protects Tumors From Immune Cell Attack.” Immunity 42: 344–355. 10.1016/j.immuni.2015.01.010 25680274 PMC4361732

[82] Goodwin, Andrew C. , Christina E. Destefano Shields , Shaoguang Wu , David L. Huso , XinQun Wu , Tracy R. Murray‐Stewart , Amy Hacker‐Prietz , et al. 2011. “Polyamine Catabolism Contributes to Enterotoxigenic *Bacteroides fragilis*‐Induced Colon Tumorigenesis.” Proceedings of the National Academy of Sciences 108: 15354–15359. 10.1073/pnas.1010203108 PMC3174648 21876161

[83] Cao, Fangfang , Lulu Jin , Yong Gao , Yuan Ding , Hongyang Wen , Zhefeng Qian , Chenyin Zhang , et al. 2023. “Artificial‐Enzymes‐Armed *Bifidobacterium longum* Probiotics for Alleviating Intestinal Inflammation and Microbiota Dysbiosis.” Nature Nanotechnology 18: 617–627. 10.1038/s41565-023-01346-x 36973397

[84] Spencer, Christine N. , Jennifer L. McQuade , Vancheswaran Gopalakrishnan , John A. McCulloch , Marie Vetizou , Alexandria P. Cogdill , Md A. Wadud Khan , et al. 2021. “Dietary Fiber and Probiotics Influence the Gut Microbiome and Melanoma Immunotherapy Response.” Science 374: 1632–1640. 10.1126/science.aaz7015 34941392 PMC8970537

[85] Zitvogel, Laurence , Lisa Derosa , Guido Kroemer . 2022. “Modulation of Cancer Immunotherapy by Dietary Fibers and Over‐the‐Counter Probiotics.” Cell Metabolism 34: 350–352. 10.1016/j.cmet.2022.02.004 35235771

[86] Sugimura, Naoki , Qing Li , Eagle Siu Hong Chu , Harry Cheuk Hay Lau , Winnie Fong , Weixin Liu , Cong Liang , et al. 2021. “ *Lactobacillus gallinarum* Modulates the Gut Microbiota and Produces Anti‐Cancer Metabolites to Protect Against Colorectal Tumourigenesis.” Gut 71: 2011–2021. 10.1136/gutjnl-2020-323951 34937766 PMC9484392

[87] Nan, Ke , Ziwen Zhong , Ying Yue , Yang Shen , Hao Zhang , Zhiqiang Wang , Kameina Zhuma , et al. 2025. “Fasting‐Mimicking Diet‐Enriched *Bifidobacterium pseudolongum* Suppresses Colorectal Cancer by Inducing Memory CD8+ T Cells.” Gut 74:775–786. 10.1136/gutjnl-2024-333020 39870395

[88] Mitra, Anita , Murat Gultekin , Laura Burney Ellis , Nicolò Bizzarri , Sarah Bowden , Nadja Taumberger , Taja Bracic , et al. 2024. “Genital Tract Microbiota Composition Profiles and Use of Prebiotics and Probiotics in Gynaecological Cancer Prevention: Review of the Current Evidence, the European Society of Gynaecological Oncology Prevention Committee Statement.” The Lancet. Microbe 5: e291–e300. 10.1016/S2666-5247(23)00257-4 38141634

[89] Yu, Guoqin , Nan Hu , Lemin Wang , Chaoyu Wang , Xiao‐You Han , Mike Humphry , Jacques Ravel , et al. 2017. “Gastric Microbiota Features Associated With Cancer Risk Factors and Clinical Outcomes: A Pilot Study in Gastric Cardia Cancer Patients From Shanxi, China.” International Journal of Cancer 141: 45–51. 10.1002/ijc.30700 28319273 PMC5839466

[90] Huang, Hechen , Zhigang Ren , Xingxing Gao , Xiaoyi Hu , Yuan Zhou , Jianwen Jiang , Haifeng Lu , et al. 2020. “Integrated Analysis of Microbiome and Host Transcriptome Reveals Correlations Between Gut Microbiota and Clinical Outcomes in HBV‐Related Hepatocellular Carcinoma.” Genome Medicine 12: 102. 10.1186/s13073-020-00796-5 33225985 PMC7682083

[91] Liu, Qisha , Fan Li , Yaoyao Zhuang , Jian Xu , Jianwei Wang , Xuhua Mao , Yewei Zhang , Xingyin Liu . 2019. “Alteration in Gut Microbiota Associated With Hepatitis B and Non‐Hepatitis Virus Related Hepatocellular Carcinoma.” Gut Pathogens 11, 1: 1. 10.1186/s13099-018-0281-6 30675188 PMC6337822

[92] Tanoue, Takeshi , Satoru Morita , Damian R. Plichta , Ashwin N. Skelly , Wataru Suda , Yuki Sugiura , Seiko Narushima , et al. 2019. “A Defined Commensal Consortium Elicits CD8 T Cells and Anti‐Cancer Immunity.” Nature 565: 600–605. 10.1038/s41586-019-0878-z 30675064

[93] Bäumler, Andreas J. , Vanessa Sperandio . 2016. “Interactions Between the Microbiota and Pathogenic Bacteria in the Gut.” Nature 535: 85–93. 10.1038/nature18849 27383983 PMC5114849

[94] Chen, Yongyan , Wenwen Cui , Xiao Li , Huan Yang . 2021. “Interaction Between Commensal Bacteria, Immune Response and the Intestinal Barrier in Inflammatory Bowel Disease.” Frontiers in Immunology 12: 761981. 10.3389/fimmu.2021.761981 34858414 PMC8632219

[95] Zheng, Yajuan , Zhaoyuan Fang , Yun Xue , Jian Zhang , Junjie Zhu , Renyuan Gao , Shun Yao , et al. 2020. “Specific Gut Microbiome Signature Predicts the Early‐Stage Lung Cancer.” Gut Microbes 11: 1030–1042. 10.1080/19490976.2020.1737487 32240032 PMC7524275

[96] Hu, Shixian , Arno R. Bourgonje , Ranko Gacesa , Bernadien H. Jansen , Johannes R. Björk , Amber Bangma , Iwan J. Hidding , et al. 2024. “Mucosal Host‐Microbe Interactions Associate With Clinical Phenotypes in Inflammatory Bowel Disease.” Nature Communications 15: 1470. 10.1038/s41467-024-45855-2 PMC10874382 38368394

[97] Grasberger, Helmut , Andrew T. Magis , Elisa Sheng , Matthew P. Conomos , Min Zhang , Lea S. Garzotto , Guoqing Hou , et al. 2021. “DUOX2 Variants Associate With Preclinical Disturbances in Microbiota‐Immune Homeostasis and Increased Inflammatory Bowel Disease Risk.” The Journal of Clinical Investigation 131: e141676. 10.1172/JCI141676 33651715 PMC8087203

[98] Bishehsari, Faraz , Robin M. Voigt , Ali Keshavarzian . 2020. “Circadian Rhythms and the Gut Microbiota: From the Metabolic Syndrome to Cancer.” Nature Reviews Endocrinology 16: 731–739. 10.1038/s41574-020-00427-4 PMC8085809 33106657

[99] Smith, Alexander B. , Matthew L., Jenior L. Jenior , Orlaith Keenan , Jessica L. Hart , Jonathan Specker , Arwa Abbas , Paula C. Rangel , et al. 2022. “Enterococci Enhance *Clostridioides difficile* Pathogenesis.” Nature 611: 780–786. 10.1038/s41586-022-05438-x 36385534 PMC9691601

[100] Leimbach, Andreas , Jörg Hacker , Ulrich Dobrindt . 2013. “ *E.?coli* as an All‐Rounder: The Thin Line Between Commensalism and Pathogenicity.” Current Topics in Microbiology and Immunology 358: 3–32. 10.1007/82_2012_303 23340801

[101] Vogt, Stefanie L. , Antonio Serapio‐Palacios , Sarah E. Woodward , Andrew S. Santos , Stefan P. W. De Vries , Michelle C. Daigneault , Lisa V. Brandmeier , et al. 2023. “ *Enterohemorrhagic Escherichia coli* Responds to Gut Microbiota Metabolites by Altering Metabolism and Activating Stress Responses.” Gut Microbes 15: 2190303. 10.1080/19490976.2023.2190303 36951510 PMC10038027

[102] Scott, Timothy A. , Kate S. Baker , Caroline Trotter , Claire Jenkins , Serge Mostowy , Jane Hawkey , Hayden Schmidt , et al. 2024. “ *Shigella sonnei*: Epidemiology, Evolution, Pathogenesis, Resistance and Host Interactions.” Nature Reviews Microbiology 23: 303–317 10.1038/s41579-024-01126-x 39604656

[103] Rao, Krishna , Preeti N. Malani . 2020. “Diagnosis and Treatment of *Clostridioides (Clostridium) difficile* Infection in Adults in 2020.” JAMA 323: 1403–1404. 10.1001/jama.2019.3849 32150234

[104] Lin, Liyuan , Yahui Du , Jia Song , Wei Wang , Chaoyong Yang . 2021. “Imaging Commensal Microbiota and Pathogenic Bacteria in the Gut.” Accounts of Chemical Research 54: 2076–2087. 10.1021/acs.accounts.1c00068 33856204

[105] Kong, Cheng , Lei Liang , Guang Liu , Lutao Du , Yongzhi Yang , Jianqiang Liu , Debing Shi , Xinxiang Li , Yanlei Ma . 2023. “Integrated Metagenomic and Metabolomic Analysis Reveals Distinct Gut‐Microbiome‐Derived Phenotypes in Early‐Onset Colorectal Cancer.” Gut 72: 1129–1142. 10.1136/gutjnl-2022-327156 35953094

[106] Warren, René L. , Douglas J. Freeman , Stephen Pleasance , Peter Watson , Richard A. Moore , Kyla Cochrane , Emma Allen‐Vercoe , Robert A. Holt . 2013. “Co‐Occurrence of Anaerobic Bacteria in Colorectal Carcinomas.” Microbiome 1: 16. 10.1186/2049-2618-1-16 24450771 PMC3971631

[107] Wu, Na , Xi Yang , Ruifen Zhang , Jun Li , Xue Xiao , Yongfei Hu , Yanfei Chen , et al. 2013. “Dysbiosis Signature of Fecal Microbiota in Colorectal Cancer Patients.” Microbial Ecology 66: 462–470. 10.1007/s00248-013-0245-9 23733170

[108] Avilés‐Jiménez, F. , A. Guitron , F. Segura‐López , A. Méndez‐Tenorio , S. Iwai , A. Hernández‐Guerrero , J. Torres . 2016. “Microbiota Studies in the Bile Duct Strongly Suggest a Role for *Helicobacter pylori* in Extrahepatic Cholangiocarcinoma.” Clinical Microbiology and Infection 22: 178.e11–178.e22. 10.1016/j.cmi.2015.10.008 26493848

[109] Nagaraja, V. , G. D. Eslick . 2014. “Systematic Review With Meta‐Analysis: The Relationship Between Chronic *Salmonella Typhi* Carrier Status and Gall‐Bladder Cancer.” Alimentary Pharmacology & Therapeutics 39: 745–750. 10.1111/apt.12655 24612190

[110] Pushalkar, Smruti , Mautin Hundeyin , Donnele Daley , Constantinos P. Zambirinis , Emma Kurz , Ankita Mishra , Navyatha Mohan , et al. 2018. “The Pancreatic Cancer Microbiome Promotes Oncogenesis by Induction of Innate and Adaptive Immune Suppression.” Cancer Discovery 8: 403–416. 10.1158/2159-8290.CD-17-1134 29567829 PMC6225783

[111] Ma, Ji , Lingqi Sun , Ying Liu , Hui Ren , Yali Shen , Feng Bi , Tao Zhang , Xin Wang . 2020. “Alter Between Gut Bacteria and Blood Metabolites and the Anti‐Tumor Effects of *Faecalibacterium prausnitzii* in Breast Cancer.” BMC Microbiology 20: 82. 10.1186/s12866-020-01739-1 32272885 PMC7144064

[112] He, Chuan , Yue Liu , Shandong Ye , Shiwu Yin , Junfei Gu . 2021. “Changes of Intestinal Microflora of Breast Cancer in Premenopausal Women.” European Journal of Clinical Microbiology & Infectious Diseases: Official Publication of the European Society of Clinical Microbiology 40: 503–513. 10.1007/s10096-020-04036-x 32936397

[113] Plottel, Claudia S. , Martin J. Blaser . 2011. “Microbiome and Malignancy.” Cell Host & Microbe 10: 324–335. 10.1016/j.chom.2011.10.003 22018233 PMC3264051

[114] Rea, Domenica , Giovanni Coppola , Giuseppe Palma , Antonio Barbieri , Antonio Luciano , Paola Del Prete , Sabrina Rossetti , et al. 2018. “Microbiota Effects on Cancer: From Risks to Therapies.” Oncotarget 9: 17915–17927. 10.18632/oncotarget.24681 29707157 PMC5915165

[115] Lu, Xingbing , Li Xiong , Xi Zheng , Qiuju Yu , Yuling Xiao , Yi Xie . 2023. “Structure of Gut Microbiota and Characteristics of Fecal Metabolites in Patients With Lung Cancer.” Frontiers in Cellular and Infection Microbiology 13: 1170326. 10.3389/fcimb.2023.1170326 37577375 PMC10415071

[116] Wei, Zixin , Biying Yang , Tiantian Tang , Zijing Xiao , Fengzhan Ye , Xiaoyu Li , Shangbin Wu , Jin‐gang Huang , Shanping Jiang . 2023. “Gut Microbiota and Risk of Five Common Cancers: A Univariable and Multivariable Mendelian Randomization Study.” Cancer Medicine 12: 10393–10405. 10.1002/cam4.5772 36880394 PMC10225193

[117] Ajani, Jaffer A. , Jeeyun Lee , Takeshi Sano , Yelena Y. Janjigian , Daiming Fan , Shumei Song . 2017. “Gastric Adenocarcinoma.” Nature Reviews Disease Primers 3: 17036. 10.1038/nrdp.2017.36 28569272

[118] Zhao, Ya‐Shuang , Fan Wang , Dong Chang , Bing Han , Ding‐Yun You . 2008. “Meta‐analysis of Different Test Indicators: *Helicobacter pylori* Infection and the Risk of Colorectal Cancer.” International Journal of Colorectal Disease 23: 875–882. 10.1007/s00384-008-0479-z 18506454

[119] Ertz‐Archambault, Natalie , Paul Keim , Daniel Von Hoff . 2017. “Microbiome and Pancreatic Cancer: A Comprehensive Topic Review of Literature.” World Journal of Gastroenterology 23: 1899–1908. 10.3748/wjg.v23.i10.1899 28348497 PMC5352932

[120] Kim, Tae Jun Kim, Eun Ran Chang, Dong Kyung Kim, Young‐Ho Baek, Sun‐Young Kim, Kyunga Hong, Sung Noh . 2017. “ *Helicobacter pylori* Infection Is an Independent Risk Factor of Early and Advanced Colorectal Neoplasm.” Helicobacter 22: elocator. 10.1111/hel.12377 28124492

[121] Shah, Shailja C. , M. Constanza Camargo , Mark Lamm , Ranier Bustamante , Christianne L. Roumie , Otis Wilson , Alese E. Halvorson , et al. 2024. “Impact of *Helicobacter pylori* Infection and Treatment on Colorectal Cancer in a Large, Nationwide Cohort.” Journal of Clinical Oncology 42: 1881–1889. 10.1200/JCO.23.00703 38427927 PMC11588569

[122] Krüttgen, Alexander , Hans‐Peter Horz , Josefine Weber‐Heynemann , Mihael Vucur , Christian Trautwein , Gerhard Haase , Tom Luedde , Christoph Roderburg . 2012. “Study on the Association of Helicobacter Species With Viral Hepatitis‐Induced Hepatocellular Carcinoma.” Gut Microbes 3: 228–233. 10.4161/gmic.19922 22572832 PMC3427215

[123] Rocha, M . 2005. “Association of Helicobacter Species With Hepatitis C Cirrhosis With or Without Hepatocellular Carcinoma.” Gut 54: 396–401. 10.1136/gut.2004.042168 15710989 PMC1774397

[124] Fox, J. G. , Y. Feng , E. J. Theve , A. R. Raczynski , J. L. A. Fiala , A. L. Doernte , M. Williams , et al. 2010. “Gut Microbes Define Liver Cancer Risk in Mice Exposed to Chemical and Viral Transgenic Hepatocarcinogens.” Gut 59: 88–97. 10.1136/gut.2009.183749 19850960 PMC3891362

[125] Murphy, Gwen , Angelika Michel , Philip R. Taylor , Demetrius Albanes , Stephanie J. Weinstein , Jarmo Virtamo , Dominick Parisi , et al. 2014. “Association of Seropositivity to Helicobacter Species and Biliary Tract Cancer in the ATBC Study.” Hepatology 60: 1963–1971. 10.1002/hep.27193 24797247 PMC4216769

[126] Segura‐López, Fany K. , Francisco Avilés‐Jiménez , Alfredo Güitrón‐Cantú , Hilda A. Valdéz‐Salazar , Samuel León‐Carballo , Leoncio Guerrero‐Pérez , James G. Fox , Javier Torres . 2015. “Infection With *Helicobacter bilis* but not *Helicobacter hepaticus* Was Associated With Extrahepatic Cholangiocarcinoma.” Helicobacter 20: 223–230. 10.1111/hel.12195 25582431

[127] Shimoyama, Tadashi , Ryoki Takahashi , Daijiro Abe , Ichiro Mizuki , Tetsu Endo , Shinsaku Fukuda . 2010. “Serological Analysis of *Helicobacter hepaticus* Infection in Patients With Biliary and Pancreatic Diseases.” Journal of Gastroenterology and Hepatology 25 Suppl 1: S86–S89. 10.1111/j.1440-1746.2010.06224.x 20586873

[128] Sharma, Prateek , Shravani M. Phatak , Prisha Warikoo , Akshita Mathur , Shweta Mahant , Kunal Das , Rajashree Das . 2023. “Crosstalk Between *Helicobacter pylori* and Gastrointestinal Microbiota in Various Gastroduodenal Diseases—A Systematic Review.” 3 Biotech 13: 303. 10.1007/s13205-023-03734-5 PMC10425313 37588796

[129] Rokkas, Theodoros , Dimitrios Pistiolas , Panos Sechopoulos , Ioannis Robotis , Georgios Margantinis . 2007. “Relationship Between *Helicobacter pylori* Infection and Esophageal Neoplasia: A Meta‐Analysis.” Clinical Gastroenterology and Hepatology 5: 1413–7.e2.e14172. 10.1016/j.cgh.2007.08.010 17997357

[130] Corley, D. A. , A. Kubo , T. R. Levin , G. Block , L. Habel , W. Zhao , P. Leighton , et al. 2008. “ *Helicobacter pylori* Infection and the Risk of Barrett's Oesophagus: A Community‐Based Study.” Gut 57: 727–733. 10.1136/gut.2007.132068 17895354 PMC2670583

[131] Castaño‐Rodríguez, Natalia , Nadeem O. Kaakoush , Way Seah Lee , Hazel M. Mitchell . 2017. “Dual Role of Helicobacter and Campylobacter Species in IBD: A Systematic Review and Meta‐Analysis.” Gut 66: 235–249. 10.1136/gutjnl-2015-310545 26508508

[132] Whiteman, David C. , Priya Parmar , Paul Fahey , Suzanne P. Moore , Mitchell Stark , Zhen Zhen Zhao , Grant W. Montgomery , et al. 2010. “Association of *Helicobacter pylori* Infection With Reduced Risk for Esophageal Cancer Is Independent of Environmental and Genetic Modifiers.” Gastroenterology 139: 73–83; quiz e11‐12. 10.1053/j.gastro.2010.04.009 20399210

[133] Owyang, Stephanie Y. , Jay Luther , Christopher C. Owyang , Min Zhang , John Y. Kao . 2012. “ *Helicobacter pylori* DNA's Anti‐Inflammatory Effect on Experimental Colitis.” Gut Microbes 3: 168–171. 10.4161/gmic.19181 22356863 PMC3370948

[134] Luther, J. , S. Y. Owyang , T. Takeuchi , T. S. Cole , M. Zhang , M. Liu , J. Erb‐Downward , et al. 2011. “ *Helicobacter pylori* DNA Decreases Pro‐Inflammatory Cytokine Production by Dendritic Cells and Attenuates Dextran Sodium Sulphate‐Induced Colitis.” Gut 60: 1479–1486. 10.1136/gut.2010.220087 21471567 PMC3466055

[135] McColl, K. E. L. , H. Watabe , M. H. Derakhshan . 2008. “Role of Gastric Atrophy in Mediating Negative Association Between *Helicobacter pylori* Infection and Reflux Oesophagitis, Barrett's Oesophagus and Oesophageal Adenocarcinoma.” Gut 57: 721–723. 10.1136/gut.2007.144774 18477672

[136] Anderson, L. A. , S. J. Murphy , B. T. Johnston , R. G. P. Watson , H. R. Ferguson , K. B. Bamford , A. Ghazy , et al. 2008. “Relationship Between *Helicobacter pylori* Infection and Gastric Atrophy and the Stages of the Oesophageal Inflammation, Metaplasia, Adenocarcinoma Sequence: Results From the FINBAR Case‐Control Study.” Gut 57: 734–739. 10.1136/gut.2007.132662 18025067

[137] Chen, Hui , Chuanli Ren . 2024. “ *Helicobacter pylori* Eradication Treatment Might Help Reduce the Risk of Esophageal Adenocarcinoma.” Gastroenterology 167: 1507–1508. 10.1053/j.gastro.2024.08.043 39306253

[138] Wiklund, Anna‐Klara , Giola Santoni , Jane Yan , Cecilia Radkiewicz , Shaohua Xie , Helgi Birgisson , Eivind Ness‐Jensen , et al. 2024. “Risk of Esophageal Adenocarcinoma After *Helicobacter pylori* Eradication Treatment in a Population‐Based Multinational Cohort Study.” Gastroenterology 167: 485–92.e3. 10.1053/j.gastro.2024.03.016 38513743

[139] Zhu, Lei , Xingxing Jian , Bingjing Zhou , Runqiu Liu , Melba Muñoz , Wan Sun , Lu Xie , et al. 2024. “Gut Microbiota Facilitate Chronic Spontaneous Urticaria.” Nature Communications 15: 112. 10.1038/s41467-023-44373-x PMC10762022 38168034

[140] Agaronyan, Karen , Lokesh Sharma , Bharat Vaidyanathan , Keith Glenn , Shuang Yu , Charles Annicelli , Talia D. Wiggen , et al. 2022. “Tissue Remodeling by an Opportunistic Pathogen Triggers Allergic Inflammation.” Immunity 55: 895–911.e10. 10.1016/j.immuni.2022.04.001 35483356 PMC9123649

[141] Yu, Linda Chia‐Hui . 2018. “Microbiota Dysbiosis and Barrier Dysfunction in Inflammatory Bowel Disease and Colorectal Cancers: Exploring a Common Ground Hypothesis.” Journal of Biomedical Science 25: 79. 10.1186/s12929-018-0483-8 30413188 PMC6234774

[142] Schwabe, Robert F. , Tim F. Greten . 2020. “Gut Microbiome in HCC—Mechanisms, Diagnosis and Therapy.” Journal of Hepatology 72: 230–238. 10.1016/j.jhep.2019.08.016 31954488

[143] Wu, Juan , Cong Zhang , Shuo Xu , Chunjie Xiang , Ruiping Wang , Dongqing Yang , Lu, Bin , et al. 2020. “Fecal Microbiome Alteration May Be a Potential Marker for Gastric Cancer.” Disease Markers 2020: 3461315. 10.1155/2020/3461315 33014185 PMC7519184

[144] Boleij, Annemarie , Elizabeth M. Hechenbleikner , Andrew C. Goodwin , Ruchi Badani , Ellen M. Stein , Mark G. Lazarev , Brandon Ellis , et al. 2015. “The Bacteroides Fragilis Toxin Gene Is Prevalent in the Colon Mucosa of Colorectal Cancer Patients.” Clinical Infectious Diseases: An Official Publication of the Infectious Diseases Society of America 60: 208–215. 10.1093/cid/ciu787 25305284 PMC4351371

[145] Ulger Toprak, N. Yagci, A. Gulluoglu, B. M. Akin, M. L. Demirkalem, P. Celenk, T. Soyletir, G . 2006. “A Possible Role of Bacteroides Fragilis Enterotoxin in the Aetiology of Colorectal Cancer.” Clinical Microbiology and Infection 12: 782–786. 10.1111/j.1469-0691.2006.01494.x 16842574

[146] Wei, Zhiliang , Shougen Cao , Shanglong Liu , Zengwu Yao , Teng Sun , Yi Li , Jiante Li , Dongfeng Zhang , Yanbing Zhou . 2016. “Could Gut Microbiota Serve as Prognostic Biomarker Associated With Colorectal Cancer Patients' Survival? A Pilot Study on Relevant Mechanism.” Oncotarget 7: 46158–46172. 10.18632/oncotarget.10064 27323816 PMC5216788

[147] Abdulamir, Ahmed S. , Rand R. Hafidh , Fatimah Bakar . 2010. “Molecular Detection, Quantification, and Isolation of *Streptococcus gallolyticus* Bacteria Colonizing Colorectal Tumors: Inflammation‐Driven Potential of Carcinogenesis via IL‐1, COX‐2, and IL‐8.” Molecular Cancer 9: 249. 10.1186/1476-4598-9-249 20846456 PMC2946291

[148] Boleij, Annemarie , Harold Tjalsma . 2013. “The Itinerary of *Streptococcus gallolyticus* Infection in Patients With Colonic Malignant Disease.” The Lancet. Infectious Diseases 13: 719–724. 10.1016/S1473-3099(13)70107-5 23831427

[149] Balamurugan, Ramadass , Ethendhar Rajendiran , Sarah George , G. Vijay Samuel , Balakrishnan S. Ramakrishna . 2008. “Real‐Time Polymerase Chain Reaction Quantification of Specific Butyrate‐Producing Bacteria, Desulfovibrio and *Enterococcus faecalis* in the Feces of Patients With Colorectal Cancer.” Journal of Gastroenterology and Hepatology 23: 1298–1303. 10.1111/j.1440-1746.2008.05490.x 18624900

[150] Huycke, Mark M. , Victoria Abrams , Danny R. Moore . 2002. “ *Enterococcus faecalis* Produces Extracellular Superoxide and Hydrogen Peroxide That Damages Colonic Epithelial Cell DNA.” Carcinogenesis 23: 529–536. 10.1093/carcin/23.3.529 11895869

[151] Kohi, Shiro , Anne Macgregor‐Das , Mohamad Dbouk , Takeichi Yoshida , Miguel Chuidian , Toshiya Abe , Michael Borges , et al. 2022. “Alterations in the Duodenal?Fluid Microbiome of Patients With Pancreatic Cancer.” Clinical Gastroenterology and Hepatology 20: e196–e227. 10.1016/j.cgh.2020.11.006 33161160 PMC8120597

[152] Riquelme, Erick , Yu Zhang , Liangliang Zhang , Maria Montiel , Michelle Zoltan , Wenli Dong , Pompeyo Quesada , et al. 2019. “Tumor Microbiome Diversity and Composition Influence Pancreatic Cancer Outcomes.” Cell 178: 795–806.e12. 10.1016/j.cell.2019.07.008 31398337 PMC7288240

[153] Mitsuhashi, Kei , Katsuhiko Nosho , Yasutaka Sukawa , Yasutaka Matsunaga , Miki Ito , Hiroyoshi Kurihara , Shinichi Kanno , et al. 2015. “Association of Fusobacterium Species in Pancreatic Cancer Tissues With Molecular Features and Prognosis.” Oncotarget 6: 7209–7220. 10.18632/oncotarget.3109 25797243 PMC4466679

[154] Bullman, Susan , Chandra S. Pedamallu , Ewa Sicinska , Thomas E. Clancy , Xiaoyang Zhang , Diana Cai , Donna Neuberg , et al. 2017. “Analysis of Fusobacterium Persistence and Antibiotic Response in Colorectal Cancer.” Science 358: 1443–1448. 10.1126/science.aal5240 29170280 PMC5823247

[155] Yu, Jiahui , Yongyu Chen , Xiangsheng Fu , Xian Zhou , Yan Peng , Lei Shi , Ting Chen , Yaxin Wu . 2016. “Invasive *Fusobacterium nucleatum* May Play a Role in the Carcinogenesis of Proximal Colon Cancer Through the Serrated Neoplasia Pathway.” International Journal of Cancer 139: 1318–1326. 10.1002/ijc.30168 27130618

[156] Rubinstein, Mara Roxana, Xiaowei Wang, Wendy Liu, Yujun Hao, Guifang Cai, Yiping W. Han . 2013. “ *Fusobacterium nucleatum* Promotes Colorectal Carcinogenesis by Modulating E‐Cadherin/β‐Catenin Signaling via Its FadA Adhesin.” Cell Host & Microbe 14: 195–206. 10.1016/j.chom.2013.07.012 23954158 PMC3770529

[157] Zhou, Dongqin , Yongsheng Li . 2023. “Gut Microbiota and Tumor‐Associated Macrophages: Potential in Tumor Diagnosis and Treatment.” Gut Microbes 15: 2276314. 10.1080/19490976.2023.2276314 37943609 PMC10653702

[158] Wang, Man , Fei Yu , Peifeng Li . 2023. “Intratumor Microbiota in Cancer Pathogenesis and Immunity: From Mechanisms of Action to Therapeutic Opportunities.” Frontiers in Immunology 14: 1269054. 10.3389/fimmu.2023.1269054 37868956 PMC10587687

[159] Udayasuryan, Barath , Raffae N. Ahmad , Tam T. D. Nguyen , Ariana Umaña , LaDeidra Monét Roberts , Polina Sobol , Stephen D. Jones , et al. 2022. “ *Fusobacterium nucleatum* Induces Proliferation and Migration in Pancreatic Cancer Cells Through Host Autocrine and Paracrine Signaling.” Science Signaling 15: eabn4948. 10.1126/scisignal.abn4948 36256708 PMC9732933

[160] Brennan, Caitlin A. , Slater L. Clay , Sydney L. Lavoie , Sena Bae , Jessica K. Lang , Diogo Fonseca‐Pereira , Kathryn G. Rosinski , et al. 2021. “ *Fusobacterium nucleatum* Drives a Pro‐Inflammatory Intestinal Microenvironment Through Metabolite Receptor‐Dependent Modulation of IL‐17 Expression.” Gut Microbes 13: 1987780. 10.1080/19490976.2021.1987780 34781821 PMC8604392

[161] Wong, Sunny H. , Thomas N. Y. Kwong , Tai‐Cheong Chow , Arthur K. C. Luk , Rudin Z. W. Dai , Geicho Nakatsu , Thomas Y. T. Lam , et al. 2017. “Quantitation of Faecal Fusobacterium Improves Faecal Immunochemical Test in Detecting Advanced Colorectal Neoplasia.” Gut 66: 1441–1448. 10.1136/gutjnl-2016-312766 27797940 PMC5530471

[162] Radaic, Allan , Eliah R. Shamir , Kyle Jones , Alessandro Villa , Nandita R. Garud , Aaron D. Tward , Pachiyappan Kamarajan , Yvonne L. Kapila . 2023. “Specific Oral Microbial Differences in Proteobacteria and Bacteroidetes Are Associated With Distinct Sites When Moving From Healthy Mucosa to Oral Dysplasia—A Microbiome and Gene Profiling Study and Focused Review.” Microorganisms 11: 2250. 10.3390/microorganisms11092250 37764094 PMC10534919

[163] Zhang, Kai , Cheng He , Yuan Qiu , Xiuyang Li , Jian Hu , Baiping Fu . 2023. “Association of Oral Microbiota and Periodontal Disease With Lung Cancer: A Systematic Review and Meta‐Analysis.” Journal of Evidence‐Based Dental Practice 23: 101897. 10.1016/j.jebdp.2023.101897 37689446

[164] Pignatelli, Pamela , Federica Nuccio , Adriano Piattelli , Maria Cristina Curia . 2023. “The Role of *Fusobacterium nucleatum* in Oral and Colorectal Carcinogenesis.” Microorganisms 11: 2358. 10.3390/microorganisms11092358 37764202 PMC10537357

[165] Chen, Linfu , Rui Zhao , Zheyu Kang , Zhiqin Cao , Nanhui Liu , Jingjing Shen , Cheng Wang , et al. 2023. “Delivery of Short Chain Fatty Acid Butyrate to Overcome *Fusobacterium nucleatum*‐Induced Chemoresistance.” Journal of Controlled Release 363: 43–56. 10.1016/j.jconrel.2023.09.028 37734673

[166] Zhang, Sheng , Yongzhi Yang , Wenhao Weng , Bomin Guo , Guoxiang Cai , Yanlei Ma , Sanjun Cai . 2019. “ *Fusobacterium nucleatum* Promotes Chemoresistance to 5‐Fluorouracil by Upregulation of BIRC3 Expression in Colorectal Cancer.” Journal of Experimental & Clinical Cancer Research: CR 38: 14. 10.1186/s13046-018-0985-y 30630498 PMC6327560

[167] Yu, TaChung , Fangfang Guo , Yanan Yu , Tiantian Sun , Ma, Dan , Jixuan Han , Yun Qian , et al. 2017. “ *Fusobacterium nucleatum* Promotes Chemoresistance to Colorectal Cancer by Modulating Autophagy.” Cell 170: 548–63.e16. 10.1016/j.cell.2017.07.008 28753429 PMC5767127

[168] Yang, Hui , Jinghui Cheng , Hao Zhuang , Hongchuang Xu , Yinuo Wang , Tingting Zhang , Yinmo Yang , et al. 2024. “Pharmacogenomic Profiling of Intra‐Tumor Heterogeneity Using a Large Organoid Biobank of Liver Cancer.” Cancer Cell 42: 535–51.e8. 10.1016/j.ccell.2024.03.004 38593780

[169] Ma, Xiaolu , Kaixia Zhou , Tianqing Yan , Ling Hu , Suhong Xie , Hui Zheng , Ying Tong , et al. 2024. “Calpain 2 Promotes Lenvatinib Resistance and Cancer Stem Cell Traits via Both Proteolysis‐Dependent and Independent Approach in Hepatocellular Carcinoma.” Molecular Biomedicine 5: 74. 10.1186/s43556-024-00242-7 39739077 PMC11688263

[170] Li, Xinxiu , Hongmeng Su , Wenqing Tang , Shihui Shu , Luyu Zhao , Jinghan Sun , Hong Fan . 2024. “Targeting LEF1‐Mediated Epithelial‐Mesenchymal Transition Reverses Lenvatinib Resistance in Hepatocellular Carcinoma.” Investigational New Drugs 42: 185–195. 10.1007/s10637-024-01426-2 38372948

[171] Van der Merwe, Michelle , Gustav Van Niekerk , Alf Botha , Anna‐Mart Engelbrecht . 2021. “The Onco‐Immunological Implications of *Fusobacterium nucleatum* in Breast Cancer.” Immunology Letters 232: 60–66. 10.1016/j.imlet.2021.02.007 33647328

[172] Allen, D. W. , P. Cole . 1973. “Oncogenic RNA Viruses.” CA: A Cancer Journal for Clinicians 23: 193–200. 10.3322/canjclin.23.3.193 4122296

[173] MacLennan, Signe A. , Marco A. Marra . 2023. “Oncogenic Viruses and the Epigenome: How Viruses Hijack Epigenetic Mechanisms to Drive Cancer.” International Journal of Molecular Sciences 24: 9543. 10.3390/ijms24119543 37298494 PMC10253656

[174] Zur Hausen, Harald 2001. “Oncogenic DNA Viruses.” Oncogene 20: 7820–7823. 10.1038/sj.onc.1204958 11753664

[175] Akkari, Leila , Damien Grégoire , Nicolas Floc'h , Marie Moreau , Céline Hernandez , Yannick Simonin , Arielle R. Rosenberg , Patrice Lassus , Urszula Hibner . 2012. “Hepatitis C Viral Protein NS5A Induces EMT and Participates in Oncogenic Transformation of Primary Hepatocyte Precursors.” Journal of Hepatology 57: 1021–1028. 10.1016/j.jhep.2012.06.027 22750466

[176] Hannigan, Geoffrey D. , Melissa B. Duhaime , Mack T. Ruffin , Charlie C. Koumpouras , Patrick D. Schloss . 2018. “Diagnostic Potential and Interactive Dynamics of the Colorectal Cancer Virome.” mBio 9: e02248‐18. 10.1128/mBio.02248-18 30459201 PMC6247079

[177] Li, Yazhen , Qiancheng Qiu , Zhiqiang Fan , Ping He , Huanzhu Chen , Xiaoyang Jiao . 2018. “Th17 Cytokine Profiling of Colorectal Cancer Patients With or Without Enterovirus 71 Antigen Expression.” Cytokine 107: 35–42. 10.1016/j.cyto.2017.11.012 29175261

[178] Arzumanyan, A. , T. Friedman , E. Kotei , I. O. L. Ng , Z. Lian , M. A. Feitelson . 2012. “Epigenetic Repression of E‐Cadherin Expression by Hepatitis B Virus x Antigen in Liver Cancer.” Oncogene 31: 563–572. 10.1038/onc.2011.255 21706058 PMC3183380

[179] Pagano, Joseph?S. , Martin Blaser , Marie‐Annick Buendia , Blossom Damania , Kamel Khalili , Nancy Raab‐Traub , Bernard Roizman . 2004. “Infectious Agents and Cancer: Criteria for a Causal Relation.” Seminars in Cancer Biology 14: 453–471. 10.1016/j.semcancer.2004.06.009 15489139

[180] Shin, Wing Sum , Fuda Xie , Bonan Chen , Jun Yu , Kwok Wai Lo , Gary M. K. Tse , Ka Fai To , Wei Kang . 2023. “Exploring the Microbiome in Gastric Cancer: Assessing Potential Implications and Contextualizing Microorganisms beyond *H. pylori* and Epstein–Barr Virus.” Cancers 15: 4993. 10.3390/cancers15204993 37894360 PMC10605912

[181] Liu, Liying , Yanan Xie , Hong Yang , Anqi Lin , Minjun Dong , Haitao Wang , Cangang Zhang , et al. 2023. “HPVTIMER: A Shiny Web Application for Tumor Immune Estimation in Human Papillomavirus‐Associated Cancers.” iMeta 2: e130. 10.1002/imt2.130 38867938 PMC10989930

[182] Lacunza, Ezequiel , Valeria Fink , María E. Salas , Romina Canzoneri , Julián Naipauer , Sion Williams , Omar Coso , et al. 2023. “Oral and Anal Microbiome From HIV‐Exposed Individuals: Role of Host‐Associated Factors in Taxa Composition and Metabolic Pathways.” NPJ Biofilms and Microbiomes 9: 48. 10.1038/s41522-023-00413-4 37438354 PMC10338440

[183] Nakatsu, Geicho , Haokui Zhou , William Ka Kei Wu , Sunny Hei Wong , Olabisi Oluwabukola Coker , Zhenwei Dai , Xiangchun Li , et al. 2018. “Alterations in Enteric Virome Are Associated With Colorectal Cancer and Survival Outcomes.” Gastroenterology 155: 529–41.e5. 10.1053/j.gastro.2018.04.018 29689266

[184] Luo, Shiqi , Jinlong Ru , Mohammadali Khan Mirzaei , Jinling Xue , Xue Peng , Anna Ralser , Raquel Mejías‐Luque , Markus Gerhard , Li Deng . 2023. “Gut Virome Profiling Identifies an Association Between Temperate Phages and Colorectal Cancer Promoted by *Helicobacter pylori* Infection.” Gut Microbes 15: 2257291. 10.1080/19490976.2023.2257291 37747149 PMC10578192

[185] Wang, Zhihan , Kai Guo , Yingying Liu , Canhua Huang , Min Wu . 2022. “Dynamic Impact of Virome on Colitis and Colorectal Cancer: Immunity, Inflammation, Prevention and Treatment.” Seminars in Cancer Biology 86: 943–954. 10.1016/j.semcancer.2021.10.004 34656791 PMC9008076

[186] Gogokhia, Lasha , Kate Buhrke , Rickesha Bell , Brenden Hoffman , D. Garrett Brown , Christin Hanke‐Gogokhia , Nadim J. Ajami , et al. 2019. “Expansion of Bacteriophages Is Linked to Aggravated Intestinal Inflammation and Colitis.” Cell Host & Microbe 25: 285–99.e8. 10.1016/j.chom.2019.01.008 30763538 PMC6885004

[187] Lawson, James S. , Wendy K. Glenn . 2021. “Catching Viral Breast Cancer.” Infectious Agents and Cancer 16: 37. 10.1186/s13027-021-00366-3 34108009 PMC8191131

[188] Herbein, Georges , Amit Kumar . 2014. “The Oncogenic Potential of Human Cytomegalovirus and Breast Cancer.” Frontiers in Oncology 4: 230. 10.3389/fonc.2014.00230 25202681 PMC4142708

[189] Richardson, A. K. , L. C. Walker , B. Cox , H. Rollag , B. A. Robinson , H. Morrin , J. F. Pearson , et al. 2020. “Breast Cancer and Cytomegalovirus.” Clinical & Translational Oncology: Official Publication of the Federation of Spanish Oncology Societies and of the National Cancer Institute of Mexico 22: 585–602. 10.1007/s12094-019-02164-1 31256361

[190] Curty, Gislaine , Jez L. Marston , Miguel De Mulder Rougvie , Fabio E. Leal , Douglas F. Nixon , Marcelo A. Soares . 2020. “Human Endogenous Retrovirus K in Cancer: A Potential Biomarker and Immunotherapeutic Target.” Viruses 12: 726. 10.3390/v12070726 32640516 PMC7412025

[191] Gao, Peng , Jie Zheng . 2011. “Oncogenic Virus‐Mediated Cell Fusion: New Insights Into Initiation and Progression of Oncogenic Viruses—Related Cancers.” Cancer Letters 303: 1–8. 10.1016/j.canlet.2010.12.021 21306823

[192] Purdy, John G. , Micah A. Luftig . 2019. “Reprogramming of Cellular Metabolic Pathways by Human Oncogenic Viruses.” Current Opinion in Virology 39: 60–69. 10.1016/j.coviro.2019.11.002 31766001 PMC6986357

[193] Huang, Binhao , Qin Li , Qirong Geng , Jiawen Lao , Jing Guo , Shenglin Huang , Dazhi Xu . 2022. “ASTE1 Frameshift Mutation Triggers the Immune Response in Epstein–Barr Virus‐Associated Gastric Cancer.” Signal Transduction and Targeted Therapy 7: 4. 10.1038/s41392-021-00771-5 34983924 PMC8727626

[194] Deng, Chu‐Xia . 2024. “Revealing the Secret Behind Epstein–Barr Virus‐Specific Tumor Immune Contexture.” Cancer Communications 44: 491–494. 10.1002/cac2.12529 38446537 PMC11024679

[195] Armstrong, Heather , Mandana Rahbari , Heekuk Park , David Sharon , Aducio Thiesen , Naomi Hotte , Ning Sun , et al. 2023. “Mouse Mammary Tumor Virus Is Implicated in Severity of Colitis and Dysbiosis in the IL‐10−/− Mouse Model of Inflammatory Bowel Disease.” Microbiome 11: 39. 10.1186/s40168-023-01483-4 36869359 PMC9983191

[196] McBride, Alison A. , Elizabeth A. White . 2023. “HPV Integration Can Drive the Formation of Virus–Host Extrachromosomal DNA in Tumors.” Cancer Discovery 13: 814–816. 10.1158/2159-8290.CD-23-0097 37009703 PMC11350425

[197] He, Jiang , Liyu Liu , Feiyu Tang , You Zhou , Huan Liu , Can Lu , Deyun Feng , et al. 2021. “Paradoxical Effects of DNA Tumor Virus Oncogenes on Epithelium‐Derived Tumor Cell Fate During Tumor Progression and Chemotherapy Response.” Signal Transduction and Targeted Therapy 6: 408. 10.1038/s41392-021-00787-x 34836940 PMC8626493

[198] Sekiba, Kazuma , Motoyuki Otsuka , Kazuyoshi Funato , Yu Miyakawa , Eri Tanaka , Takahiro Seimiya , Mari Yamagami , et al. 2022. “HBx‐Induced Degradation of Smc5/6 Complex Impairs Homologous Recombination‐Mediated Repair of Damaged DNA.” Journal of Hepatology 76: 53–62. 10.1016/j.jhep.2021.08.010 34478763

[199] Xia, Wei , Lei Liu , Nan Shi , Chaoyin Zhang , Anzhou Tang , Guangyao He . 2023. “Epstein Barr Virus Infection in Tree Shrews Alters the Composition of Gut Microbiota and Metabolome Profile.” Virology Journal 20: 177. 10.1186/s12985-023-02147-3 37553712 PMC10410904

[200] Easwaran, Maheswaran , Fatma Abdelrahman , Sivagnanavelmurugan Madasamy , Baskar Venkidasamy . 2024. “Phage‐Delivered Melittin for Oral Squamous Cell Carcinoma: A Potential Therapeutic Agent.” International Immunopharmacology 134: 112163. 10.1016/j.intimp.2024.112163 38705763

[201] York, Ashley . 2025. “Predicting Phage‐Host Specificity.” Nature Reviews Microbiology 23: 5. 10.1038/s41579-024-01132-z 39562748

[202] York, Ashley . 2020. “Prophages Are Gut Virome Pioneers.” Nature Reviews Microbiology 18: 317. 10.1038/s41579-020-0374-3 32317729

[203] Wahida, Adam , Fang Tang , Jeremy J. Barr . 2021. “Rethinking Phage–Bacteria–Eukaryotic Relationships and Their Influence on Human Health.” Cell Host & Microbe 29: 681–688. 10.1016/j.chom.2021.02.007 33735620

[204] Tisza, Michael J. , Richard E. Lloyd , Kristi Hoffman , Daniel P. Smith , Marian Rewers , Sara J. Javornik Cregeen , Joseph?F. Petrosino . 2025. “Longitudinal Phage–Bacteria Dynamics in the Early Life Gut Microbiome.” Nature Microbiology 10: 420–430. 10.1038/s41564-024-01906-4 PMC11790489 39856391

[205] Shkoporov, Andrey N. , Colin Hill . 2019. “Bacteriophages of the Human Gut: The ‘Known Unknown’ of the Microbiome.” Cell Host & Microbe 25: 195–209. 10.1016/j.chom.2019.01.017 30763534

[206] Kotsiliti, Eleni . 2024. “Bacteriophages and Host Inflammation in IBD.” Nature Reviews Gastroenterology & Hepatology 21: 300. 10.1038/s41575-024-00925-0 38519791

[207] Putra, Ramendra Dirgantara , Diana Lyrawati . 2020. “Interactions Between Bacteriophages and Eukaryotic Cells.” Scientifica 2020: 3589316. 10.1155/2020/3589316 32582449 PMC7301238

[208] Magnaye, Kevin M. , Susan V. Lynch . 2021. “Fungus Fuels Mucosal Wounds in Crohn's Disease.” Immunity 54: 856–858. 10.1016/j.immuni.2021.04.013 33979584

[209] Huo, Xiaokui , Dawei Li , Fan Wu , Shenghui Li , Yanling Qiao , Chao Wang , Wang, Yan , et al. 2022. “Cultivated Human Intestinal Fungus *Candida metapsilosis* M2006B Attenuates Colitis by Secreting Acyclic Sesquiterpenoids as FXR Agonists.” Gut 71: 2205–2217. 10.1136/gutjnl-2021-325413 35173042

[210] Cho, Daniel H. , Gloria B. Choi . 2022. “Fungus Packs a Punch in the Gut.” Immunity 55: 586–588. 10.1016/j.immuni.2022.03.012 35417672

[211] Bordon, Yvonne . 2022. “Fungus Hijacks TLR4 to Build a Type 2 Immune Niche.” Nature Reviews Immunology 22: 532–533. 10.1038/s41577-022-00773-6 35945352

[212] Ost, Kyla S. , June L. Round . 2023. “Commensal Fungi in Intestinal Health and Disease.” Nature Reviews Gastroenterology & Hepatology 20: 723–734. 10.1038/s41575-023-00816-w 37479823

[213] Ahmadi, N. , A. Ahmadi , E. Kheirali , M. Hossein Yadegari , M. Bayat , A. Shajiei , A. Ali Amini , et al. 2019. “Systemic Infection With *Candida albicans* in Breast Tumor Bearing Mice: Cytokines Dysregulation and Induction of Regulatory T Cells.” Journal de Mycologie Médicale 29: 49–55. 10.1016/j.mycmed.2018.10.006 30470620

[214] He, Shengfu , Yating Sun , Weijie Sun , Mingyang Tang , Bao Meng , Yanyan Liu , Qinxiang Kong , et al. 2023. “Oral Microbiota Disorder in GC Patients Revealed by 2b‐RAD‐M.” Journal of Translational Medicine 21: 831. 10.1186/s12967-023-04599-1 37980457 PMC10656981

[215] Dohlman, Anders B. , Jared Klug , Marissa Mesko , Iris H. Gao , Steven M. Lipkin , Xiling Shen , Iliyan D. Iliev . 2022. “A Pan‐Cancer Mycobiome Analysis Reveals Fungal Involvement in Gastrointestinal and Lung Tumors.” Cell 185: 3807–22.e12. 10.1016/j.cell.2022.09.015 36179671 PMC9564002

[216] Chin, Siok‐Fong , Putri Intan Hafizah Megat Mohd Azlan , Luqman Mazlan , Hui‐Min Neoh . 2018. “Identification of *Schizosaccharomyces pombe* in the Guts of Healthy Individuals and Patients With Colorectal Cancer: Preliminary Evidence From a Gut Microbiome Secretome Study.” Gut Pathogens 10: 29. 10.1186/s13099-018-0258-5 30008808 PMC6040075

[217] Li, Fan , Yunhuan Gao , Wenyue Cheng , Xiaomin Su , Rongcun Yang . 2023. “Gut Fungal Mycobiome: A Significant Factor of Tumor Occurrence and Development.” Cancer Letters 569: 216302. 10.1016/j.canlet.2023.216302 37451425

[218] Wang, Yu , Yahui Ren , Yongming Huang , Xiangnan Yu , Yiming Yang , Di Wang , Liang Shi , et al. 2021. “Fungal Dysbiosis of the Gut Microbiota Is Associated With Colorectal Cancer in Chinese Patients.” American Journal of Translational Research 13: 11287–11301. 34786058 PMC8581944

[219] Aykut, Berk , Smruti Pushalkar , Ruonan Chen , Qianhao Li , Raquel Abengozar , Jacqueline I. Kim , Sorin A. Shadaloey , et al. 2019. “The Fungal Mycobiome Promotes Pancreatic Oncogenesis via Activation of MBL.” Nature 574: 264–267. 10.1038/s41586-019-1608-2 31578522 PMC6858566

[220] Brayer, Kathryn J. , Joshua A. Hanson , Shashank Cingam , Cathleen Martinez , Scott A. Ness , Ian Rabinowitz . 2023. “The Inflammatory Response of Human Pancreatic Cancer Samples Compared to Normal Controls.” PloS One 18: e0284232. 10.1371/journal.pone.0284232 37910468 PMC10619777

[221] Zhang, Lilong , Dongqi Chai , Chen Chen , Chunlei Li , Zhendong Qiu , Tianrui Kuang , Mungur Parveena , et al. 2022. “Mycobiota and C‐Type Lectin Receptors in Cancers: Know Thy Neighbors.” Frontiers in Microbiology 13: 946995. 10.3389/fmicb.2022.946995 35910636 PMC9326027

[222] Wang, Xu , Zejun Zhou , David Turner , Michael Lilly , Tongwen Ou , Wei Jiang . 2022. “Differential Circulating Fungal Microbiome in Prostate Cancer Patients Compared to Healthy Control Individuals.” Journal of Immunology Research 2022: 2574964. 10.1155/2022/2574964 35155686 PMC8831061

[223] Cheng, Wenyue , Fan Li , Yunhuan Gao , Rongcun Yang . 2024. “Fungi and Tumors: The Role of Fungi in Tumorigenesis (Review).” International Journal of Oncology 64: 52. 10.3892/ijo.2024.5640 38551162 PMC10997370

[224] Galloway‐Peña, Jessica , Iliyan D. Iliev , Florencia McAllister . 2024. “Fungi in Cancer.” Nature Reviews Cancer 24: 295–298. 10.1038/s41568-024-00665-y PMC11648847 38347100

[225] Xu, Bin , Zan Luo , Xing Niu , Zhi Li , Yeping Lu , Junyu Li . 2025. “Fungi, Immunosenescence and Cancer.” Seminars in Cancer Biology 109: 67–82. 10.1016/j.semcancer.2025.01.002 39788169

[226] Li, Xiaopeng , Jiahui Feng , Zhanggui Wang , Gang Liu , Fan Wang . 2023. “Features of Combined Gut Bacteria and Fungi From a Chinese Cohort of Colorectal Cancer, Colorectal Adenoma, and Post‐Operative Patients.” Frontiers in Microbiology 14: 1236583. 10.3389/fmicb.2023.1236583 37614602 PMC10443710

[227] Coker, Olabisi Oluwabukola , Geicho Nakatsu , Rudin Zhenwei Dai , William Ka Kei Wu , Sunny Hei Wong , Siew Chien Ng , Francis Ka Leung Chan , Joseph?Jao Yiu Sung , Jun Yu . 2019. “Enteric Fungal Microbiota Dysbiosis and Ecological Alterations in Colorectal Cancer.” Gut 68: 654–662. 10.1136/gutjnl-2018-317178 30472682 PMC6580778

[228] Yang, Ping , Xiaoshan Zhang , Rui Xu , Khan Adeel , Xiaofeng Lu , Min Chen , Han Shen , Zhiyang Li , Zhipeng Xu . 2022. “Fungal Microbiota Dysbiosis and Ecological Alterations in Gastric Cancer.” Frontiers in Microbiology 13: 889694. 10.3389/fmicb.2022.889694 35572666 PMC9100745

[229] Huang, Hui , Qiurong Wang , Ying Yang , Wei Zhong , Feng He , Jun Li . 2024. “The Mycobiome as Integral Part of the Gut Microbiome: Crucial Role of Symbiotic Fungi in Health and Disease.” Gut Microbes 16: 2440111. 10.1080/19490976.2024.2440111 39676474 PMC11651280

[230] Wang, Rui , Bo Li , Bingyuan Huang , Yikang Li , Qiaoyan Liu , Zhuwan Lyu , Ruiling Chen , et al. 2024. “Gut Microbiota‐Derived Butyrate Induces Epigenetic and Metabolic Reprogramming in Myeloid‐Derived Suppressor Cells to Alleviate Primary Biliary Cholangitis.” Gastroenterology 167: 733–49.e3. 10.1053/j.gastro.2024.05.014 38810839

[231] Sinha, Anurag K. , Martin F. Laursen , Julius E. Brinck , Morten L. Rybtke , Anna Pii Hjørne , Nicola Procházková , Mikael Pedersen , Henrik M. Roager , Tine R. Licht . 2024. “Dietary Fibre Directs Microbial Tryptophan Metabolism via Metabolic Interactions in the Gut Microbiota.” Nature Microbiology 9: 1964–1978. 10.1038/s41564-024-01737-3 PMC11306097 38918470

[232] Lu, Yijing , Wenlong Yang , Zhiyong Qi , Rifeng Gao , Jing Tong , Tingwen Gao , Yin Zhang , et al. 2023. “Gut Microbe‐Derived Metabolite Indole‐3‐Carboxaldehyde Alleviates Atherosclerosis.” Signal Transduction and Targeted Therapy 8: 378. 10.1038/s41392-023-01613-2 37789009 PMC10547776

[233] Kwon, Byungsuk . 2023. “A Metabolite of the Gut Microbiota: A Facilitator of Chemotherapy Efficacy in Cancer.” Signal Transduction and Targeted Therapy 8: 238. 10.1038/s41392-023-01506-4 37291097 PMC10250301

[234] Huang, Kan , Zilun Li , Xi He , Jun Dai , Bingding Huang , Yongxia Shi , Dongxiao Fan , et al. 2024. “Gut Microbial Co‐Metabolite 2‐Methylbutyrylcarnitine Exacerbates Thrombosis via Binding to and Activating Integrin α2β1.” Cell Metabolism 36: 598–616.e9. 10.1016/j.cmet.2024.01.014 38401546

[235] Collins, Stephanie L. , Jonathan G. Stine , Jordan E. Bisanz , C. Denise Okafor , Andrew D. Patterson . 2023. “Bile Acids and the Gut Microbiota: Metabolic Interactions and Impacts on Disease.” Nature Reviews Microbiology 21: 236–247. 10.1038/s41579-022-00805-x 36253479 PMC12536349

[236] Agus, Allison , Karine Clément , Harry Sokol . 2021. “Gut Microbiota‐Derived Metabolites as Central Regulators in Metabolic Disorders.” Gut 70: 1174–1182. 10.1136/gutjnl-2020-323071 33272977 PMC8108286

[237] Belcheva, Antoaneta , Thergiory Irrazabal , Susan J. Robertson , Catherine Streutker , Heather Maughan , Stephen Rubino , Eduardo H. Moriyama , et al. 2014. “Gut Microbial Metabolism Drives Transformation of MSH2‐Deficient Colon Epithelial Cells.” Cell 158: 288–299. 10.1016/j.cell.2014.04.051 25036629

[238] Zheng, Di‐Wei , Run‐Qing Li , Jia‐Xin An , Tian‐Qiu Xie , Zi‐Yi Han , Rui Xu , Yu Fang , Xian‐Zheng Zhang . 2020. “Prebiotics‐Encapsulated Probiotic?Spores Regulate Gut Microbiota and Suppress Colon Cancer.” Advanced Materials 32: e2004529. 10.1002/adma.202004529 33006175

[239] Park, Hyun‐Soo , Joo‐Hui Han , Jeong Won Park , Do‐Hyung Lee , Keun‐Woo Jang , Miji Lee , Kyung‐Sun Heo , Chang‐Seon Myung . 2021. “Sodium Propionate Exerts Anticancer Effect in Mice Bearing Breast Cancer Cell Xenograft by Regulating JAK2/STAT3/ROS/p38 MAPK Signaling.” Acta Pharmacologica Sinica 42: 1311–1323. 10.1038/s41401-020-00522-2 32973326 PMC8285538

[240] Hilakivi‐Clarke, Leena , Fabia De Oliveira Andrade . 2023. “Social Isolation and Breast Cancer.” Endocrinology 164: bqad126. 10.1210/endocr/bqad126 37586098

[241] Luu, Maik , Zeno Riester , Adrian Baldrich , Nicole Reichardt , Samantha Yuille , Alessandro Busetti , Matthias Klein , et al. 2021. “Microbial Short‐Chain Fatty Acids Modulate CD8+ T Cell Responses and Improve Adoptive Immunotherapy for Cancer.” Nature Communications 12: 4077. 10.1038/s41467-021-24331-1 PMC8249424 34210970

[242] Kim, Minsuk , Emily Vogtmann , David A. Ahlquist , Mary E. Devens , John B. Kisiel , William R. Taylor , Bryan A. White , et al. 2020. “Fecal Metabolomic Signatures in Colorectal Adenoma Patients Are Associated With Gut Microbiota and Early Events of Colorectal Cancer Pathogenesis.” mBio 11: e03186‐19. 10.1128/mBio.03186-19 32071266 PMC7029137

[243] Hale, Vanessa L. , Jun Chen , Stephen Johnson , Sean C. Harrington , Tracy C. Yab , Thomas C. Smyrk , Heidi Nelson , et al. 2017. “Shifts in the Fecal Microbiota Associated With Adenomatous Polyps.” Cancer Epidemiology, Biomarkers & Prevention: A Publication of the American Association for Cancer Research, Cosponsored by the American Society of Preventive Oncology 26: 85–94. 10.1158/1055-9965.EPI-16-0337 PMC5225053 27672054

[244] Mikó, Edit , András Vida , Tünde Kovács , Gyula Ujlaki , György Trencsényi , Judit Márton , Zsanett Sári , et al. 2018. “Lithocholic Acid, a Bacterial Metabolite Reduces Breast Cancer Cell Proliferation and Aggressiveness.” Biochimica et Biophysica Acta (BBA)—Bioenergetics 1859: 958–974. 10.1016/j.bbabio.2018.04.002 29655782

[245] Kovács, Patrik , Tamás Csonka , Tünde Kovács , Zsanett Sári , Gyula Ujlaki , Adrien Sipos , Zsolt Karányi , et al. 2019. “Lithocholic Acid, a Metabolite of the Microbiome, Increases Oxidative Stress in Breast Cancer.” Cancers 11: 1255. 10.3390/cancers11091255 31461945 PMC6769524

[246] Sun, Xi‐Zhen , Dong‐Yan Zhao , Yuan‐Chen Zhou , Qian‐Qian Wang , Geng Qin , Shu‐Kun Yao . 2020. “Alteration of Fecal Tryptophan Metabolism Correlates With Shifted Microbiota and May Be Involved in Pathogenesis of Colorectal Cancer.” World Journal of Gastroenterology 26: 7173–7190. 10.3748/wjg.v26.i45.7173 33362375 PMC7723673

[247] Díaz‐Díaz, Carol J. , Sean M. Ronnekleiv‐Kelly , Manabu Nukaya , Peter G. Geiger , Silvia Balbo , Romel Dator , Bryant W. Megna , et al. 2016. “The Aryl Hydrocarbon Receptor Is a Repressor of Inflammation‐Associated Colorectal Tumorigenesis in Mouse.” Annals of Surgery 264: 429–436. 10.1097/SLA.0000000000001874 27433903 PMC5125557

[248] Zhang, Lu , Qing Ji , Qian Chen , Zhenzhen Wei , Shuochuan Liu , Long Zhang , Yuli Zhang , et al. 2023. “ *Akkermansia muciniphila* Inhibits Tryptophan Metabolism via the AhR/β‐Catenin Signaling Pathway to Counter the Progression of Colorectal Cancer.” International Journal of Biological Sciences 19: 4393–4410. 10.7150/ijbs.85712 37781044 PMC10535706

[249] Patel, Dhwani , Iain A. Murray , Fangcong Dong , Andrew J. Annalora , Krishne Gowda , Denise M. Coslo , Jacek Krzeminski , et al. 2023. “Induction of AHR Signaling in Response to the Indolimine Class of Microbial Stress Metabolites.” Metabolites 13: 985. 10.3390/metabo13090985 37755265 PMC10535990

[250] Seo, Su‐Kil , Byungsuk Kwon . 2023. “Immune Regulation Through Tryptophan Metabolism.” Experimental & Molecular Medicine 55: 1371–1379. 10.1038/s12276-023-01028-7 37394584 PMC10394086

[251] Hussain, Ahad , Li Xie , Guozhe Deng , Xuejun Kang . 2023. “Common Alterations in Plasma Free Amino Acid Profiles and Gut Microbiota‐Derived Tryptophan Metabolites of Five Types of Cancer Patients.” Amino Acids 55: 1189–1200. 10.1007/s00726-023-03308-y 37490156

[252] He, Jin , Peiwen Zhang , Linyuan Shen , Lili Niu , Ya Tan , Lei Chen , Ye Zhao , et al. 2020. “Short‐Chain Fatty Acids and Their Association With Signalling Pathways in Inflammation, Glucose and Lipid Metabolism.” International Journal of Molecular Sciences 21: 6356. 10.3390/ijms21176356 32887215 PMC7503625

[253] Mann, Elizabeth R. , Ying Ka Lam , Holm H. Uhlig . 2024. “Short‐Chain Fatty Acids: Linking Diet, the Microbiome and Immunity.” Nature Reviews Immunology 24: 577–595. 10.1038/s41577-024-01014-8 38565643

[254] Vinolo, Marco A. R. , Hosana G. Rodrigues , Renato T. Nachbar , Rui Curi . 2011. “Regulation of Inflammation by Short Chain Fatty Acids.” Nutrients 3: 858–876. 10.3390/nu3100858 22254083 PMC3257741

[255] Van der Hee, Bart , Jerry M. Wells . 2021. “Microbial Regulation of Host Physiology by Short‐chain Fatty Acids.” Trends in Microbiology 29: 700–712. 10.1016/j.tim.2021.02.001 33674141

[256] Tsunedomi, Ryouichi , Yoshitaro Shindo , Masao Nakajima , Kiyoshi Yoshimura , Hiroaki Nagano . 2023. “The Tumor Immune Microenvironment in Pancreatic Cancer and Its Potential in the Identification of Immunotherapy Biomarkers.” Expert Review of Molecular Diagnostics 23: 1121–1134. 10.1080/14737159.2023.2281482 37947389

[257] Yan, Qingzhu , Shengnan Jia , Dongfu Li , Junling Yang . 2023. “The Role and Mechanism of Action of Microbiota‐Derived Short‐Chain Fatty Acids in Neutrophils: From the Activation to Becoming Potential Biomarkers.” Biomedicine & Pharmacotherapy = Biomedecine & Pharmacotherapie 169: 115821. 10.1016/j.biopha.2023.115821 37952355

[258] Sun, Mingming , Wei Wu , Zhanju Liu , Yingzi Cong . 2017. “Microbiota Metabolite Short Chain Fatty Acids, GPCR, and Inflammatory Bowel Diseases.” Journal of Gastroenterology 52: 1–8. 10.1007/s00535-016-1242-9 27448578 PMC5215992

[259] Tsukuda, Naoki , Kana Yahagi , Taeko Hara , Yohei Watanabe , Hoshitaka Matsumoto , Hiroshi Mori , Koichi Higashi , et al. 2021. “Key Bacterial Taxa and Metabolic Pathways Affecting Gut Short‐Chain Fatty Acid Profiles in Early Life.” The ISME Journal 15: 2574–2590. 10.1038/s41396-021-00937-7 33723382 PMC8397723

[260] Quinn‐Bohmann, Nick , Tomasz Wilmanski , Katherine Ramos Sarmiento , Lisa Levy , Johanna W. Lampe , Thomas Gurry , Noa Rappaport , et al. 2024. “Microbial Community‐Scale Metabolic Modelling Predicts Personalized Short‐Chain Fatty Acid Production Profiles in the Human Gut.” Nature Microbiology 9: 1700–1712. 10.1038/s41564-024-01728-4 PMC11841136 38914826

[261] Nogal, Ana , Francesco Asnicar , Amrita Vijay , Afroditi Kouraki , Alessia Visconti , Panayiotis Louca , Kari Wong , et al. 2023. “Genetic and Gut Microbiome Determinants of SCFA Circulating and Fecal Levels, Postprandial Responses and Links to Chronic and Acute Inflammation.” Gut Microbes 15: 2240050. 10.1080/19490976.2023.2240050 37526398 PMC10395212

[262] Ikeda, Takako , Akari Nishida , Mayu Yamano , Ikuo Kimura . 2022. “Short‐Chain Fatty Acid Receptors and Gut Microbiota as Therapeutic Targets in Metabolic, Immune, and Neurological Diseases.” Pharmacology & Therapeutics 239: 108273. 10.1016/j.pharmthera.2022.108273 36057320

[263] Hu, Tongtong , Qingqing Wu , Qi Yao , Kebing Jiang , Jiabin Yu , Qizhu Tang . 2022. “Short‐Chain Fatty Acid Metabolism and Multiple Effects on Cardiovascular Diseases.” Ageing Research Reviews 81: 101706. 10.1016/j.arr.2022.101706 35932976

[264] Gomes, Sara , Ana Catarina Rodrigues , Valerio Pazienza , Ana Preto . 2023. “Modulation of the Tumor Microenvironment by Microbiota‐Derived Short‐Chain Fatty Acids: Impact in Colorectal Cancer Therapy.” International Journal of Molecular Sciences 24: 5069. 10.3390/ijms24065069 36982144 PMC10048801

[265] Thome, Carolin D. , Patrick Tausche , Katja Hohenberger , Zuqin Yang , Susanne Krammer , Denis I. Trufa , Horia Sirbu , Joachim Schmidt , Susetta Finotto . 2024. “Short‐Chain Fatty Acids Induced Lung Tumor Cell Death and Increased Peripheral Blood CD4+ T Cells in NSCLC and Control Patients Ex Vivo.” Frontiers in Immunology 15: 1328263. 10.3389/fimmu.2024.1328263 38650948 PMC11033355

[266] Kim, Myunghoo , Yaqing Qie , Jeongho Park , Chang H. Kim . 2016. “Gut Microbial Metabolites Fuel Host Antibody Responses.” Cell Host & Microbe 20: 202–214. 10.1016/j.chom.2016.07.001 27476413 PMC4982788

[267] Thiruvengadam, Muthu , Umadevi Subramanian , Baskar Venkidasamy , Prabhu Thirupathi , Ramkumar Samynathan , Mohammad Ali Shariati , Maksim Rebezov , Ill‐Min Chung , Kannan R. R. Rengasamy . 2023. “Emerging Role of Nutritional Short‐Chain Fatty Acids (SCFAs) Against Cancer via Modulation of Hematopoiesis.” Critical Reviews in Food Science and Nutrition 63: 827–844. 10.1080/10408398.2021.1954874 34319824

[268] Hou, Huiqin , Danfeng Chen , Kexin Zhang , Wanru Zhang , Tianyu Liu , Sinan Wang , Xin Dai , et al. 2022. “Gut Microbiota‐Derived Short‐Chain Fatty Acids and Colorectal Cancer: Ready for Clinical Translation?” Cancer Letters 526: 225–235. 10.1016/j.canlet.2021.11.027 34843863

[269] Winston, Jenessa A. , Casey M. Theriot . 2020. “Diversification of Host Bile Acids by Members of the Gut Microbiota.” Gut Microbes 11: 158–171. 10.1080/19490976.2019.1674124 31595814 PMC7053883

[270] Su, Xiaomin , Yunhuan Gao , Rongcun Yang . 2023. “Gut Microbiota Derived Bile Acid Metabolites Maintain the Homeostasis of Gut and Systemic Immunity.” Frontiers in Immunology 14: 1127743. 10.3389/fimmu.2023.1127743 37256134 PMC10225537

[271] Sinha, Sidhartha R. , Yeneneh Haileselassie , Linh P. Nguyen , Carolina Tropini , Min Wang , Laren S. Becker , Davis Sim , et al. 2020. “Dysbiosis‐Induced Secondary Bile Acid Deficiency Promotes Intestinal Inflammation.” Cell Host & Microbe 27: 659–70.e5. 10.1016/j.chom.2020.01.021 32101703 PMC8172352

[272] Režen, Tadeja , Damjana Rozman , Tünde Kovács , Patrik Kovács , Adrienn Sipos , Péter Bai , Edit Mikó . 2022. “The Role of Bile Acids in Carcinogenesis.” Cellular and Molecular Life Sciences 79: 243. 10.1007/s00018-022-04278-2 35429253 PMC9013344

[273] Lenci, Ilaria , Martina Milana , Alessandro Signorello , Giuseppe Grassi , Leonardo Baiocchi . 2023. “Secondary Bile Acids and the Biliary Epithelia: The Good and the Bad.” World Journal of Gastroenterology 29: 357–36. 10.3748/wjg.v29.i2.357 36687129 PMC9846939

[274] Situ, Yingheng , Pengpeng Zhang , Cangang Zhang , Aimin Jiang , Nan Zhang , Lingxuan Zhu , Weiming Mou , et al. 2025. “The Metabolic Dialogue Between Intratumoural Microbes and Cancer: Implications for Immunotherapy.” EBioMedicine 115: 105708. 10.1016/j.ebiom.2025.105708 40267755 PMC12052696

[275] Krumz, L. M. , R. B. Gudkova , L. Kh. Indejkina , E. A. Sabelnikova , A. I. Parfenov . 2020. “[Bile Acids Are a Risk Factor for Colorectal Cancer].” Terapevticheskii Arkhiv 92: 93–96. 10.26442/00403660.2020.02.000457 32598725

[276] Kim, Tae‐Young , Seungil Kim , Yeji Kim , Yong‐Soo Lee , Sohyeon Lee , Su‐Hyun Lee , Mi‐Na Kweon . 2022. “A High‐Fat Diet Activates the BAs‐FXR Axis and Triggers Cancer‐Associated Fibroblast Properties in the Colon.” Cellular and Molecular Gastroenterology and Hepatology 13: 1141–1159. 10.1016/j.jcmgh.2021.12.015 34971821 PMC8873938

[277] Ma, Chi , Miaojun Han , Bernd Heinrich , Qiong Fu , Qianfei Zhang , Milan Sandhu , David Agdashian , et al. 2018. “Gut Microbiome‐Mediated Bile Acid Metabolism Regulates Liver Cancer via NKT Cells.” Science 360: eaan5931. 10.1126/science.aan5931 29798856 PMC6407885

[278] Zha, Andong , Ming Qi , Yuankun Deng , Hao Li , Nan Wang , Chengming Wang , Simeng Liao , et al. 2024. “Gut *Bifidobacterium pseudocatenulatum* Protects Against Fat Deposition by Enhancing Secondary Bile Acid Biosynthesis.” iMeta 3: e261. 10.1002/imt2.261 39742294 PMC11683477

[279] Tobón‐Cornejo, Sandra , Monica Sanchez‐Tapia , Rocio Guizar‐Heredia , Laura Velázquez Villegas , Lilia G. Noriega , Janette Furuzawa‐Carballeda , Rogelio Hernández‐Pando , et al. 2025. “Increased Dietary Protein Stimulates Amino Acid Catabolism via the Gut Microbiota and Secondary Bile Acid Production.” Gut Microbes 17: 2465896. 10.1080/19490976.2025.2465896 39980327 PMC11849929

[280] Louca, Panayiotis , Abraham S. Meijnikman , Ana Nogal , Francesco Asnicar , Ilias Attaye , Amrita Vijay , Afroditi Kouraki , et al. 2023. “The Secondary Bile Acid Isoursodeoxycholate Correlates With Post‐Prandial Lipemia, Inflammation, and Appetite and Changes Post‐Bariatric Surgery.” Cell Reports. Medicine 4: 100993. 10.1016/j.xcrm.2023.100993 37023745 PMC10140478

[281] Lamichhane, Santosh , Partho Sen , Alex M. Dickens , Marina Amaral Alves , Taina Härkönen , Jarno Honkanen , Tommi Vatanen , et al. 2022. “Dysregulation of Secondary Bile Acid Metabolism Precedes Islet Autoimmunity and Type 1 Diabetes.” Cell Reports. Medicine 3: 100762. 10.1016/j.xcrm.2022.100762 36195095 PMC9589006

[282] Wei, Yanxin , Chen Lu , Shengsheng Jiang , Yanyan Zhang , Qiuchun Li , Wen‐Ju Bai , Xiqing Wang . 2020. “Directed Evolution of a Tryptophan 2,3‐Dioxygenase for the Diastereoselective Monooxygenation of Tryptophans.” Angewandte Chemie International Edition 59: 3043–3047. 10.1002/anie.201911825 31828916

[283] Cervenka, Igor , Leandro Z. Agudelo , Jorge L. Ruas . 2017. “Kynurenines: Tryptophan's Metabolites in Exercise, Inflammation, and Mental Health.” Science 357: eaaf9794. 10.1126/science.aaf9794 28751584

[284] Zhu, Yueting , Aolei Hu , Meilin Chen , Yunting Zhang , Tao Gong , Zhirong Zhang , Ruilian Yu , Yao Fu . 2025. “3‐Indoleacetic Acid‐Modified Chondroitin Sulfate‐Mediated Paclitaxel Nanocrystal Assembly for the Treatment of Pancreatic Cancer.” ACS Applied Materials & Interfaces 17: 9035–9046. 10.1021/acsami.4c19450 39901810

[285] Li, Zhuangzhuang , Baoyan Ding , Mustafa R. K. Ali , Lizhen Zhao , Xiaoling Zang , Zhihua Lv . 2022. “Dual Effect of Tryptamine on Prostate Cancer Cell Growth Regulation: A Pilot Study.” International Journal of Molecular Sciences 23: 11087. 10.3390/ijms231911087 36232383 PMC9569450

[286] Jing, Wanghui , Sijing Dong , Yinyue Xu , Jingjing Liu , Jiawei Ren , Xue Liu , Min Zhu , et al. 2025. “Gut Microbiota‐Derived Tryptophan Metabolites Regulated by Wuji Wan to Attenuate Colitis Through AhR Signaling Activation.” Acta Pharmaceutica Sinica. B 15: 205–223. 10.1016/j.apsb.2024.11.009 40041900 PMC11873645

[287] Chen, Chuan , Zheng Cao , Hehua Lei , Cui Zhang , Mengjing Wu , Shaohua Huang , Xinzhi Li , et al. 2024. “Microbial Tryptophan Metabolites Ameliorate Ovariectomy‐Induced Bone Loss by Repairing Intestinal AhR‐Mediated Gut‐Bone Signaling Pathway.” Advanced Science (Weinheim, Baden‐Wurttemberg, Germany) 11: e2404545. 10.1002/advs.202404545 39041942 PMC11423200

[288] Arinze, Nkiruka V. , Wenqing Yin , Saran Lotfollahzadeh , Marc Arthur Napoleon , Sean Richards , Joshua A. Walker , Mostafa Belghasem , et al. 2022. “Tryptophan Metabolites Suppress the Wnt Pathway and Promote Adverse Limb Events in Chronic Kidney Disease.” The Journal of Clinical Investigation 132: e142260. 10.1172/JCI142260 34752422 PMC8718145

[289] Platten, Michael , Nikolaus Von Knebel Doeberitz , Iris Oezen , Wolfgang Wick , Katharina Ochs . 2014. “Cancer Immunotherapy by Targeting IDO1/TDO and Their Downstream Effectors.” Frontiers in Immunology 5: 673. 10.3389/fimmu.2014.00673 25628622 PMC4290671

[290] Ghosh, Sweta , Caleb Samuel Whitley , Bodduluri Haribabu , Venkatakrishna Rao Jala . 2021. “Regulation of Intestinal Barrier Function by Microbial Metabolites.” Cellular and Molecular Gastroenterology and Hepatology 11: 1463–1482. 10.1016/j.jcmgh.2021.02.007 33610769 PMC8025057

[291] Turpin, Williams , Sun‐Ho Lee , Juan Antonio Raygoza Garay , Karen L. Madsen , Jonathan B. Meddings , Larbi Bedrani , Namita Power , et al. 2020. “Increased Intestinal Permeability Is Associated With Later Development of Crohn's Disease.” Gastroenterology 159: 2092–100.e5. 10.1053/j.gastro.2020.08.005 32791132

[292] Tozzi, Michela , Alessia Fiore , Sara Travaglione , Francesca Marcon , Gabriella Rainaldi , Elena Angela Pia Germinario , Ilenia Laterza , et al. 2025. “ *E. Coli* Cytotoxic Necrotizing Factor‐1 Promotes Colorectal Carcinogenesis by Causing Oxidative Stress, DNA Damage and Intestinal Permeability Alteration.” Journal of Experimental & Clinical Cancer Research: CR 44: 29. 10.1186/s13046-024-03271-w 39876002 PMC11776187

[293] Leibovitzh, Haim , Sun‐Ho Lee , Mingyue Xue , Juan Antonio Raygoza Garay , Cristian Hernandez‐Rocha , Karen L. Madsen , Jonathan B. Meddings , et al. 2022. “Altered Gut Microbiome Composition and Function Are Associated With Gut Barrier Dysfunction in Healthy Relatives of Patients With Crohn's Disease.” Gastroenterology 163: 1364–76.e10. 10.1053/j.gastro.2022.07.004 35850197

[294] Santos, Javier , Maria Rescigno . 2024. “Gut Barrier Leakiness: Time to Take It Seriously?” Gastroenterology 167: 1080–1082. 10.1053/j.gastro.2024.08.011 39154775

[295] Magnus, Yorick , Joelle BouSaba , Wassel Sannaa , Sanna McKinzie , Irene Busciglio , Michael Camilleri . 2022. “Bile Acid Diarrhea Is Associated With Increased Intestinal Permeability Compared With Irritable Bowel Syndrome‐Diarrhea.” Gastroenterology 162: 1343–5.e1. 10.1053/j.gastro.2021.12.243 34922946 PMC8934275

[296] Lee, Sun‐Ho , Maham Bushra , Lanhui Qiu , Anne M. Griffiths , Williams Turpin , Kenneth Croitoru , Crohn's and Colitis Canada‐Genetic, Environmental, Microbial (CCC‐GEM) Project Research Consortium , Kenneth Croitoru , Sun‐Ho Lee , et al. 2025. “Early Life Exposure to Parental Crohn's Disease Is Associated With Offspring's Gut Microbiome, Gut Permeability, and Increased Risk of Future Crohn's Disease.” Gastroenterology 168: 385–8.e3. 10.1053/j.gastro.2024.09.033 39384162

[297] Horowitz, Arie , Sandra D. Chanez‐Paredes , Xenia Haest , Jerrold R. Turner . 2023. “Paracellular Permeability and Tight Junction Regulation in Gut Health and Disease.” Nature Reviews Gastroenterology & Hepatology 20: 417–432. 10.1038/s41575-023-00766-3 37186118 PMC10127193

[298] Chopyk, Daniel M. , Arash Grakoui . 2020. “Contribution of the Intestinal Microbiome and Gut Barrier to Hepatic Disorders.” Gastroenterology 159: 849–863. 10.1053/j.gastro.2020.04.077 32569766 PMC7502510

[299] Rashidah, Nur Hannah , Siong Meng Lim , Chin Fen Neoh , Majeed, Abu Bakar Abdul Tan, Maw Pin , Hui Min Khor , Ai Huey Tan , Siti Hajar Rehiman , Kalavathy Ramasamy ,. 2022. “Differential Gut Microbiota and Intestinal Permeability Between Frail and Healthy Older Adults: A Systematic Review.” Ageing Research Reviews 82: 101744. 10.1016/j.arr.2022.101744 36202312

[300] Martel, Jan , Shih‐Hsin Chang , Yun‐Fei Ko , Tsong‐Long Hwang , John D. Young , David M. Ojcius . 2022. “Gut Barrier Disruption and Chronic Disease.” Trends in Endocrinology & Metabolism 33: 247–265. 10.1016/j.tem.2022.01.002 35151560

[301] Giambra, Vincenzo , Danilo Pagliari , Pierluigi Rio , Beatrice Totti , Chiara Di Nunzio , Annalisa Bosi , Cristina Giaroni , et al. 2023. “Gut Microbiota, Inflammatory Bowel Disease, and Cancer: The Role of Guardians of Innate Immunity.” Cells 12: 2654. 10.3390/cells12222654 37998389 PMC10669933

[302] Soler, A. P . 1999. “Increased Tight Junctional Permeability Is Associated With the Development of Colon Cancer.” Carcinogenesis 20: 1425–1432. 10.1093/carcin/20.8.1425 10426787

[303] Karczewski, Jurgen , Freddy J. Troost , Irene Konings , Jan Dekker , Kleerebezem, Michiel Brummer, Robert‐Jan M. Wells, Jerry M . 2010. “Regulation of Human Epithelial Tight Junction Proteins by *Lactobacillus plantarum* In Vivo and Protective Effects on the Epithelial Barrier.” American Journal of Physiology‐Gastrointestinal and Liver Physiology 298: G851–G859. 10.1152/ajpgi.00327.2009 20224007

[304] Méndez‐Sánchez, Nahum , Alejandro Valencia‐Rodriguez , Alfonso Vera‐Barajas , Ludovico Abenavoli , Emidio Scarpellini , Guadalupe Ponciano‐Rodriguez . 2020. “The Mechanism of Dysbiosis in Alcoholic Liver Disease Leading to Liver Cancer.” Hepatoma Research 6: 5. 10.20517/2394-5079.2019.29 32582865 PMC7313221

[305] Rychter, Anna Maria , Liliana Łykowska‐Szuber , Agnieszka Zawada , Aleksandra Szymczak‐Tomczak , Alicja Ewa Ratajczak , Kinga Skoracka , Michalina Kolan , Agnieszka Dobrowolska , Iwona Krela‐Kaźmierczak . 2023. “Why Does Obesity as an Inflammatory Condition Predispose to Colorectal Cancer?” Journal of Clinical Medicine 12: 2451. 10.3390/jcm12072451 37048534 PMC10094909

[306] Klein, Gerald L. , Bryon W. Petschow , Audrey L. Shaw , Eric Weaver . 2013. “Gut Barrier Dysfunction and Microbial Translocation in Cancer Cachexia: A New Therapeutic Target.” Current Opinion in Supportive and Palliative Care 7: 361–367. 10.1097/SPC.0000000000000017 24157715 PMC3819310

[307] Wang, Hong‐Bo , Peng‐Yuan Wang , Xin Wang , Yuan‐Lian Wan , Yu‐Cun Liu . 2012. “Butyrate Enhances Intestinal Epithelial Barrier Function via Up‐Regulation of Tight Junction Protein Claudin‐1 Transcription.” Digestive Diseases and Sciences 57: 3126–3135. 10.1007/s10620-012-2259-4 22684624

[308] Fachi, José Luís , Jaqueline de Souza Felipe , Laís Passariello Pral , Bruna Karadi Da Silva , Renan Oliveira Corrêa , Mirella Cristiny Pereira De Andrade , Denise Morais Da Fonseca , et al. 2019. “Butyrate Protects Mice From Clostridium Difficile‐Induced Colitis Through an HIF‐1‐Dependent Mechanism.” Cell Reports 27: 750–61.e7. 10.1016/j.celrep.2019.03.054 30995474

[309] Chen, Guangxin , Xin Ran , Bai Li , Yuhang Li , Dewei He , Bingxu Huang , Shoupeng Fu , Juxiong Liu , Wei Wang . 2018. “Sodium Butyrate Inhibits Inflammation and Maintains Epithelium Barrier Integrity in a TNBS‐Induced Inflammatory Bowel Disease Mice Model.” EBioMedicine 30: 317–325. 10.1016/j.ebiom.2018.03.030 29627390 PMC5952406

[310] Tong, Yao , Huiru Gao , Qiuchen Qi , Xiaoyan Liu , Juan Li , Jie Gao , Peilong Li , et al. 2021. “High Fat Diet, Gut Microbiome and Gastrointestinal Cancer.” Theranostics 11: 5889–5910. 10.7150/thno.56157 33897888 PMC8058730

[311] Muszyński, Damian , Anna Kudra , Bartosz Kamil Sobocki , Marcin Folwarski , Ermanno Vitale , Veronica Filetti , Wojciech Dudzic , Karolina Kaźmierczak‐Siedlecka , Karol Połom . 2022. “Esophageal Cancer and Bacterial Part of Gut Microbiota—A Multidisciplinary Point of View.” Frontiers in Cellular and Infection Microbiology 12: 1057668. 10.3389/fcimb.2022.1057668 36467733 PMC9709273

[312] Huang, Hui , Wei Zhong , Xiaojiao Wang , Ying Yang , Tianmu Wu , Runyang Chen , Yanling Liu , Feng He , Jun Li . 2023. “The Role of Gastric Microecological Dysbiosis in Gastric Carcinogenesis.” Frontiers in Microbiology 14: 1218395. 10.3389/fmicb.2023.1218395 37583514 PMC10423824

[313] Miao, Sainan , Huan Qiu . 2024. “The Microbiome in the Pathogenesis of Lung Cancer: The Role of Microbiome in Lung Cancer Pathogenesis.” APMIS 132: 68–80. 10.1111/apm.13359 37974493

[314] Lo, Bobby , Mirabella Zhao , Ida Vind , Johan Burisch . 2021. “The Risk of Extraintestinal Cancer in Inflammatory Bowel Disease: A Systematic Review and Meta‐analysis of Population‐Based Cohort Studies.” Clinical Gastroenterology and Hepatology 19: 1117–38.e19. 10.1016/j.cgh.2020.08.015 32801010

[315] Lavelle, Aonghus , Stéphane Nancey , Jean‐Marie Reimund , David Laharie , Philippe Marteau , Xavier Treton , Matthieu Allez , et al. 2022. “Fecal Microbiota and Bile Acids in IBD Patients Undergoing Screening for Colorectal Cancer.” Gut Microbes 14: 2078620. 10.1080/19490976.2022.2078620 35638103 PMC9176255

[316] Jin, Xuanhong , Liangkun You , Jincheng Qiao , Weidong Han , Hongming Pan . 2024. “Autophagy in Colitis‐Associated Colon Cancer: Exploring Its Potential Role in Reducing Initiation and Preventing IBD‐Related CAC Development.” Autophagy 20: 242–258. 10.1080/15548627.2023.2259214 37723664 PMC10813649

[317] Zhao, Rui , Qian‐Yi Wan , Yutao Wu , Yong Wang , Ya‐Ping Cui , Xiaoding Shen , Xiao Ting Wu . 2021. “Crohn's Disease Instead of UC Might Increase the Risk of Small Bowel Cancer.” Gut 70: 809–810. 10.1136/gutjnl-2020-322201 32665339

[318] Wetwittayakhlang, Panu , Petra A. Golovics , Lorant Gonczi , Laszlo Lakatos , Peter L. Lakatos , Gyula David , Zsuzsanna Erdelyi , et al. VESZPREM EPIDEMIOLOGY STUDY GROUP . 2024. “Stable Incidence and Risk Factors of Colorectal Cancer in Ulcerative Colitis: A Population‐Based Cohort Between 1977–2020.” Clinical Gastroenterology and Hepatology 22: 191–3.e3. 10.1016/j.cgh.2023.03.022 37004972

[319] Shah, Shailja C. , Steven H. Itzkowitz . 2022. “Colorectal Cancer in Inflammatory Bowel Disease: Mechanisms and Management.” Gastroenterology 162: 715–30.e3. 10.1053/j.gastro.2021.10.035 34757143 PMC9003896

[320] Axelrad, Jordan E. , Ola Olén , Michael C. Sachs , Rune Erichsen , Lars Pedersen , Jonas Halfvarson , Johan Askling , et al. 2021. “Inflammatory Bowel Disease and Risk of Small Bowel Cancer: A Binational Population‐Based Cohort Study From Denmark and Sweden.” Gut 70: 297–308. 10.1136/gutjnl-2020-320945 32474410

[321] Elmahdi, Rahma , Camilla E. Lemser , Sandra B. Thomsen , Kristine H. Allin , Manasi Agrawal , Tine Jess . 2022. “Development of Cancer Among Patients With Pediatric‐Onset Inflammatory Bowel Disease: A Meta‐Analysis of Population‐Based Studies.” JAMA Network Open 5: e220595. 10.1001/jamanetworkopen.2022.0595 35230438 PMC8889462

[322] Li, Junshu , Yanhong Ji , Na Chen , Lei Dai , Hongxin Deng . 2023. “Colitis‐Associated Carcinogenesis: Crosstalk Between Tumors, Immune Cells and Gut Microbiota.” Cell & Bioscience 13: 194. 10.1186/s13578-023-01139-8 37875976 PMC10594787

[323] Deris Zayeri, Zeinab , Abazar Parsi , Saeid Shahrabi , Masoud Kargar , Nader Davari , Najmaldin Saki . 2023. “Epigenetic and Metabolic Reprogramming in Inflammatory Bowel Diseases: Diagnostic and Prognostic Biomarkers in Colorectal Cancer.” Cancer Cell International 23: 264. 10.1186/s12935-023-03117-z 37936149 PMC10631091

[324] Liang, Bing , Yanhong Wang , Jiazhen Xu , Yingchun Shao , Dongming Xing . 2023. “Unlocking the Potential of Targeting Histone‐Modifying Enzymes for Treating IBD and CRC.” Clinical Epigenetics 15: 146. 10.1186/s13148-023-01562-1 37697409 PMC10496233

[325] Silveira, Denise Sayuri Calheiros , Luciana Chain Veronez , Luís Carlos Lopes‐Júnior , Elen Anatriello , Mariângela Ottoboni Brunaldi , Gabriela Pereira‐da‐Silva . 2020. “ *Lactobacillus bulgaricus* Inhibits Colitis‐Associated Cancer via a Negative Regulation of Intestinal Inflammation in Azoxymethane/Dextran Sodium Sulfate Model.” World Journal of Gastroenterology 26: 6782–6794. 10.3748/wjg.v26.i43.6782 33268961 PMC7684459

[326] Javid, Hossein , Mahsa Akbari Oryani , Sanaz Akbari , Taghi Amiriani , Samaneh Ravanbakhsh , Nastaran Rezagholinejad , Amir‐R. Afshari , Mehdi Karimi‐Shahri . 2023. “ *L. plantarum* and *L. lactis* as a Promising Agent in Treatment of Inflammatory Bowel Disease and Colorectal Cancer.” Future Microbiology 18: 1197–1209. 10.2217/fmb-2023-0076 37882738

[327] Wittek, Agnes , Babett Steglich , Christian Casar , Oliver Seiz , Philipp Huber , Hanno Ehlken , Dominik Reher , et al. 2024. “A Gradient of Intestinal Inflammation in Primary Sclerosing Cholangitis.” Inflammatory Bowel Diseases 30: 900–910. 10.1093/ibd/izad137 37540889

[328] Zhang, Henan , Junrui Wu , Na Li , Rina Wu , Wei Chen . 2023. “Microbial Influence on Triggering and Treatment of Host Cancer: An Intestinal Barrier Perspective.” Biochimica et Biophysica Acta (BBA)—Reviews on Cancer 1878: 188989. 10.1016/j.bbcan.2023.188989 37742727

[329] Wang, Lei , Mengfan Li , Yu Gu , Junli Shi , Jing Yan , Xin Wang , Bingqing Li , et al. 2024. “Dietary Flavonoids‐Microbiota Crosstalk in Intestinal Inflammation and Carcinogenesis.” The Journal of Nutritional Biochemistry 125: 109494. 10.1016/j.jnutbio.2023.109494 37866426

[330] Intratumor Microbes Promote Murine Breast Cancer Cell Invasion. 2022. Cancer Discovery 12: 1407. 10.1158/2159-8290.CD-RW2022-067 35452082

[331] Liu, Ning‐Ning , Cheng‐Xiang Yi , Lu‐Qi Wei , Jin‐An Zhou , Tong Jiang , Cong‐Cong Hu , Lu Wang , et al. 2024. “The Intratumor Mycobiome Promotes Lung Cancer Progression via Myeloid‐Derived Suppressor Cells.” Cancer Cell 42: 318–322. 10.1016/j.ccell.2024.01.005 38350423

[332] Liu, Ning‐Ning , Cheng‐Xiang Yi , Lu‐Qi Wei , Jin‐An Zhou , Tong Jiang , Cong‐Cong Hu , Lu Wang , et al. 2023. “The Intratumor Mycobiome Promotes Lung Cancer Progression via Myeloid‐Derived Suppressor Cells.” Cancer Cell 41: 1927–44.e9. 10.1016/j.ccell.2023.08.012 37738973

[333] Gao, Zhifei , Aimin Jiang , Zizhuo Li , Lingxuan Zhu , Weiming Mou , Weitao Shen , Peng Luo , et al. 2025. “Heterogeneity of Intratumoral Microbiota Within the Tumor Microenvironment and Relationship to Tumor Development.” Med Research 1(1): 32–61. 10.1002/mdr2.70006

[334] Yu, Chunyue , Zeqi Su , Yicong Li , Yadong Li , Kaige Liu , Fuhao Chu , Ting Liu , Runhua Chen , Xia Ding . 2020. “Dysbiosis of Gut Microbiota Is Associated With Gastric Carcinogenesis in Rats.” Biomedicine & Pharmacotherapy = Biomedecine & Pharmacotherapie 126: 110036. 10.1016/j.biopha.2020.110036 32172061

[335] Sun, Lejia , Xindi Ke , Ai Guan , Bao Jin , Jiangming Qu , Yinhan Wang , Xiang Xu , et al. 2023. “Intratumoural Microbiome Can Predict the Prognosis of Hepatocellular Carcinoma After Surgery.” Clinical and Translational Medicine 13: e1331. 10.1002/ctm2.1331 37462602 PMC10353526

[336] Liu, Fangjie , Qiaoting Luo , Yu Xi , Pengxin Zhang , Yingjia Wu , Suping Guo , Yaoling Dong , et al. 2025. “Early Nutritional Intervention in Patients With Non‐Small Cell Lung Cancer Receiving Concurrent Chemoradiotherapy: A Phase II Prospective Study.” Nutrients 17: 1389. 10.3390/nu17081389 40284252 PMC12030435

[337] Huang, Jian‐Hang , Jie Wang , Xiao‐Qiang Chai , Zhong‐Chen Li , Ying‐Hua Jiang , Jun Li , Xing Liu , et al. 2022. “The Intratumoral Bacterial Metataxonomic Signature of Hepatocellular Carcinoma.” Microbiology Spectrum 10: e0098322. 10.1128/spectrum.00983-22 36173308 PMC9602924

[338] Fu, Aikun , Bingqing Yao , Tingting Dong , Yongyi Chen , Jia Yao , Yu Liu , Hang Li , et al. 2022. “Tumor‐Resident Intracellular Microbiota Promotes Metastatic Colonization in Breast Cancer.” Cell 185: 1356–72.e26. 10.1016/j.cell.2022.02.027 35395179

[339] An, Jeongshin , Hyungju Kwon , Young Ju Kim . 2023. “The Firmicutes/Bacteroidetes Ratio as a Risk Factor of Breast Cancer.” Journal of Clinical Medicine 12: 2216. 10.3390/jcm12062216 36983217 PMC10052522

[340] Mouradov, Dmitri , Paul Greenfield , Shan Li , Eun‐Jung In , Claire Storey , Anuratha Sakthianandeswaren , Peter Georgeson , et al. 2023. “Oncomicrobial Community Profiling Identifies Clinicomolecular and Prognostic Subtypes of Colorectal Cancer.” Gastroenterology 165: 104–120. 10.1053/j.gastro.2023.03.205 36933623

[341] Zeng, Xuejun , Hang Jia , Xiao Zhang , Xin Wang , Zhouli Wang , Zhenpeng Gao , Yahong Yuan , Tianli Yue . 2021. “Supplementation of Kefir Ameliorates Azoxymethane/Dextran Sulfate Sodium Induced Colorectal Cancer by Modulating the Gut Microbiota.” Food & Function 12: 11641–11655. 10.1039/d1fo01729b 34724014

[342] Liu, Yanhong , Jacqueline L. O'Brien , Nadim J. Ajami , Michael E. Scheurer , E. Susan Amirian , Georgina Armstrong , Spiridon Tsavachidis , et al. 2018. “Lung Tissue Microbial Profile in Lung Cancer Is Distinct From Emphysema.” American Journal of Cancer Research 8: 1775–1787. 30323970 PMC6176189

[343] Yu, Guoqin , Mitchell H. Gail , Dario Consonni , Michele Carugno , Michael Humphrys , Angela C. Pesatori , Neil E. Caporaso , et al. 2016. “Characterizing Human Lung Tissue Microbiota and Its Relationship to Epidemiological and Clinical Features.” Genome Biology 17: 163. 10.1186/s13059-016-1021-1 27468850 PMC4964003

[344] Jang, Hye Jin , Ji Yeon Choi , Kangjoon Kim , Seung Hyun Yong , Yeon Wook Kim , Song Yee Kim , Eun Young Kim , et al. 2021. “Relationship of the Lung Microbiome With PD‐L1 Expression and Immunotherapy Response in Lung Cancer.” Respiratory Research 22: 322. 10.1186/s12931-021-01919-1 34963470 PMC8715618

[345] Hilmi, Marc , Maud Kamal , Sophie Vacher , Célia Dupain , Sabrina Ibadioune , Maral Halladjian , Marie Paule Sablin , et al. 2023. “Intratumoral Microbiome Is Driven by Metastatic Site and Associated With Immune Histopathological Parameters: An Ancillary Study of the SHIVA Clinical Trial.” European Journal of Cancer 183: 152–161. 10.1016/j.ejca.2023.01.024 36868056

[346] Peng, Shengkun , Anqi Lin , Aimin Jiang , Cangang Zhang , Jian Zhang , Quan Cheng , Peng Luo , Yifeng Bai . 2024. “CTLs Heterogeneity and Plasticity: Implications for Cancer Immunotherapy.” Molecular Cancer 23: 58. 10.1186/s12943-024-01972-6 38515134 PMC10956324

[347] Zhu, Zhuxian , Jixu Cai , Weiwei Hou , Ke Xu , Xuxiao Wu , Yuanlin Song , Chunxue Bai , Yin‐Yuan Mo , Ziqiang Zhang . 2023. “Microbiome and Spatially Resolved Metabolomics Analysis Reveal the Anticancer Role of Gut *Akkermansia muciniphila* by Crosstalk With Intratumoral Microbiota and Reprogramming Tumoral Metabolism in Mice.” Gut Microbes 15: 2166700. 10.1080/19490976.2023.2166700 36740846 PMC9904296

[348] Abed, Jawad , Johanna E. M. Emgård , Gideon Zamir , Mouhammad Faroja , Gideon Almogy , Amalie Grenov , Asaf Sol , et al. 2016. “Fap2 Mediates *Fusobacterium nucleatum* Colorectal Adenocarcinoma Enrichment by Binding to Tumor‐Expressed Gal‐GalNAc.” Cell Host & Microbe 20: 215–225. 10.1016/j.chom.2016.07.006 27512904 PMC5465824

[349] Wong‐Rolle, Abigail , Qiang Dong , Yunhua Zhu , Prajan Divakar , Jyh Liang Hor , Noemi Kedei , Madeline Wong , et al. 2022. “Spatial Meta‐Transcriptomics Reveal Associations of Intratumor Bacteria Burden With Lung Cancer Cells Showing a Distinct Oncogenic Signature.” Journal for Immunotherapy of Cancer 10: e004698. 10.1136/jitc-2022-004698 35793869 PMC9260850

[350] Shirai, Hiroaki , Cocoro Ito , Kosuke Tsukada . 2022. “pH‐Taxis Drives Aerobic Bacteria in Duodenum to Migrate Into the Pancreas With Tumors.” Scientific Reports 12: 1783. 10.1038/s41598-022-05554-8 35110595 PMC8810860

[351] Zhang, Zhilin , Yiqun Liao , Dong Tang . 2022. “Intratumoral Microbiota: New Frontiers in Tumor Immunity.” Carcinogenesis 43: 719–727. 10.1093/carcin/bgac063 35868230

[352] Peng, Fei , Mengyuan Hu , Zhiyue Su , Lin Hu , Lingchuan Guo , Kai Yang . 2024. “Intratumoral Microbiota as a Target for Advanced Cancer Therapeutics.” Advanced Materials 36: e2405331. 10.1002/adma.202405331 39054925

[353] Lu, Ying‐Qi , Han Qiao , Xi‐Rong Tan , Na Liu . 2024. “Broadening Oncological Boundaries: The Intratumoral Microbiota.” Trends in Microbiology 32: 807–822. 10.1016/j.tim.2024.01.007 38310023

[354] Ferrari, Valentina , Maria Rescigno . 2023. “The Intratumoral Microbiota: Friend or Foe?” Trends in Cancer 9: 472–479. 10.1016/j.trecan.2023.03.005 37061408

[355] Zhang, Baochun , Il‐Kyu Choi . 2022. “Facts and Hopes in the Relationship of EBV With Cancer Immunity and Immunotherapy.” Clinical Cancer Research: An Official Journal of the American Association for Cancer Research 28: 4363–4369. 10.1158/1078-0432.CCR-21-3408 35686929 PMC9714122

[356] Yang, Jing , Zhifeng Liu , Bin Zeng , Hu, Guangsheng Gan, Runliang . 2020. “Epstein–Barr Virus‐Associated Gastric Cancer: A Distinct Subtype.” Cancer Letters 495: 191–199. 10.1016/j.canlet.2020.09.019 32979463

[357] Berti, Fernanda Costa Brandão , Ana Paula Lombardi Pereira , Guilherme Cesar Martelossi Cebinelli , Kleber Paiva Trugilo , Karen Brajão de Oliveira . 2017. “The Role of Interleukin 10 in Human Papilloma Virus Infection and Progression to Cervical Carcinoma.” Cytokine & Growth Factor Reviews 34: 1–13. 10.1016/j.cytogfr.2017.03.002 28365229

[358] Zur Hausen, Harald . 2002. “Papillomaviruses and Cancer: From Basic Studies to Clinical Application.” Nature Reviews Cancer 2: 342–350. 10.1038/nrc798 12044010

[359] Zheng, Zhi‐Ming . 2010. “Viral Oncogenes, Noncoding RNAs, and RNA Splicing in Human Tumor Viruses.” International Journal of Biological Sciences 6: 730–755. 10.7150/ijbs.6.730 PMC2999850 21152115

[360] Tu, Thomas , Magdalena Budzinska , Nicholas Shackel , Stephan Urban . 2017. “HBV DNA Integration: Molecular Mechanisms and Clinical Implications.” Viruses 9: 75. 10.3390/v9040075 28394272 PMC5408681

[361] Tan, Benjy J. Y. , Kenji Sugata , Omnia Reda , Misaki Matsuo , Kyosuke Uchiyama , Paola Miyazato , Vincent Hahaut , et al. 2021. “HTLV‐1 Infection Promotes Excessive T Cell Activation and Transformation Into Adult T Cell Leukemia/Lymphoma.” The Journal of Clinical Investigation 131: e150472. 10.1172/JCI150472 34907908 PMC8670839

[362] He, Yunlong , Nagesh Pasupala , Huijun Zhi , Batsuhk Dorjbal , Imran Hussain , Hsiu‐Ming Shih , Sharmistha Bhattacharyya , et al. 2021. “NF‐κB‐Induced R‐Loop Accumulation and DNA Damage Select for Nucleotide Excision Repair Deficiencies in Adult T Cell Leukemia.” Proceedings of the National Academy of Sciences of the United States of America 118: e2005568118. 10.1073/pnas.2005568118 33649200 PMC7958262

[363] Ameya, Gemechu , Dagim Jirata Birri . 2023. “The Molecular Mechanisms of Virus‐Induced Human Cancers.” Microbial Pathogenesis 183: 106292. 10.1016/j.micpath.2023.106292 37557930

[364] Chen, Jiezhong . 2015. “Signaling Pathways in HPV‐Associated Cancers and Therapeutic Implications.” Reviews in Medical Virology 25 Suppl 1: 24–53. 10.1002/rmv.1823 25752815

[365] Stanland, Lyla J. , Micah A. Luftig . 2020. “The Role of EBV‐Induced Hypermethylation in Gastric Cancer Tumorigenesis.” Viruses 12: 1222. 10.3390/v12111222 33126718 PMC7693998

[366] Lamontagne, R. Jason , Sumedha Bagga , Michael J. Bouchard . 2016. “Hepatitis B Virus Molecular Biology and Pathogenesis.” Hepatoma Research 2: 163–186. 10.20517/2394-5079.2016.05 28042609 PMC5198785

[367] Yeh, Chau‐Ting 2000. “Hepatitis B Virus X Protein: Searching for a Role in Hepatocarcinogenesis.” Journal of Gastroenterology and Hepatology 15: 339–341. 10.1046/j.1440-1746.2000.02166.x 10824873

[368] Levrero, Massimo , Jessica Zucman‐Rossi . 2016. “Mechanisms of HBV‐Induced Hepatocellular Carcinoma.” Journal of Hepatology 64: S84–S101. 10.1016/j.jhep.2016.02.021 27084040

[369] Wu, Qiong , Lu Zhang , Xiazhen Xu , Yi Zhang , Jiajian Shi , Xu Lin , Wannan Chen . 2022. “Hepatitis B Virus X Protein Is Stabilized by the Deubiquitinating Enzyme VCPIP1 in a Ubiquitin‐Independent Manner by Recruiting the 26S Proteasome Subunit PSMC3.” Journal of Virology 96: e0061122. 10.1128/jvi.00611-22 35695579 PMC9278118

[370] Kim, Chang‐Min , Kazuhiko Koike , Izumu Saito , Tatsuo Miyamura , Gilbert Jay . 1991. “HBx Gene of Hepatitis B Virus Induces Liver Cancer in Transgenic Mice.” Nature 351: 317–320. 10.1038/351317a0 2034275

[371] Pol, Stanislas , Anaïs Vallet‐Pichard , Olivier Hermine . 2018. “Extrahepatic Cancers and Chronic HCV Infection.” Nature Reviews Gastroenterology & Hepatology 15: 283–290. 10.1038/nrgastro.2017.172 29339810

[372] Liang, T. Jake Heller, Theo . 2004. “Pathogenesis of Hepatitis C‐Associated Hepatocellular Carcinoma.” Gastroenterology 127: S62–S71. 10.1053/j.gastro.2004.09.017 15508105

[373] Cinatl, Jindrich , Martin Scholz , Hans Wilhelm Doerr . 2005. “Role of Tumor Cell Immune Escape Mechanisms in Cytomegalovirus‐Mediated Oncomodulation.” Medicinal Research Reviews 25: 167–185. 10.1002/med.20018 15389728

[374] Doorbar, John , Nagayasu Egawa , Heather Griffin , Christian Kranjec , Isao Murakami . 2015. “Human Papillomavirus Molecular Biology and Disease Association.” Reviews in Medical Virology 25 Suppl 1: 2–23. 10.1002/rmv.1822 25752814 PMC5024016

[375] Tian, Tian , Cai Aijie , Huang Bingxue , Dai Jianghong . 2016. “Research Progress of Human Papilloma Virus Immune Evasion Mechanisms.” Chinese Journal of Disease Control and Prevention 20: 1172–1175. 10.16462/j.cnki.zhjbkz.2016.11.024

[376] Židovec Lepej, Snježana , Maja Matulić , Paula Gršković , Mirjana Pavlica , Leona Radmanić , Petra Korać . 2020. “miRNAs: EBV Mechanism for Escaping Host's Immune Response and Supporting Tumorigenesis.” Pathogens (Basel, Switzerland) 9: 353. 10.3390/pathogens9050353 32397085 PMC7281681

[377] Yang, Pengyuan , Geoffrey J. Markowitz , Xiao‐Fan Wang . 2014. “The Hepatitis B Virus‐Associated Tumor Microenvironment in Hepatocellular Carcinoma.” National Science Review 1: 396–412. 10.1093/nsr/nwu038 25741453 PMC4346158

[378] Narunsky‐Haziza, Lian , Gregory D. Sepich‐Poore , Ilana Livyatan , Omer Asraf , Cameron Martino , Deborah Nejman , Nancy Gavert , et al. 2022. “Pan‐Cancer Analyses Reveal Cancer‐Type‐Specific Fungal Ecologies and Bacteriome Interactions.” Cell 185: 3789–806.e17. 10.1016/j.cell.2022.09.005 36179670 PMC9567272

[379] Rahal, Zahraa , Humam Kadara . 2023. “Beyond Bacteria: How the Intratumor Mycobiome Modulates Lung Adenocarcinoma Progression.” Cancer Cell 41: 1846–1848. 10.1016/j.ccell.2023.09.002 37774700 PMC10901296

[380] Li, Xin , Deepak Saxena . 2022. “The Mycobiome‐Immune Axis: The Next Frontier in Pancreatic Cancer.” Cancer Cell 40: 120–122. 10.1016/j.ccell.2022.01.009 35167821 PMC10191147

[381] Alam, Aftab , Eric Levanduski , Parker Denz , Helena Solleiro Villavicencio , Maulasri Bhatta , Lamees Alhorebi , Yali Zhang , et al. 2022. “Fungal Mycobiome Drives IL‐33 Secretion and Type 2 Immunity in Pancreatic Cancer.” Cancer Cell 40: 153–67.e11. 10.1016/j.ccell.2022.01.003 35120601 PMC8847236

[382] Chevalier, Mathieu F. , Sara Trabanelli , Julien Racle , Bérengère Salomé , Valérie Cesson , Dalila Gharbi , Perrine Bohner , et al. 2017. “ILC2‐Modulated T Cell‐to‐MDSC Balance Is Associated With Bladder Cancer Recurrence.” The Journal of Clinical Investigation 127: 2916–2929. 10.1172/JCI89717 28650339 PMC5531411

[383] Liu, Lanxiang , Haiyang Wang , Xueyi Chen , Yangdong Zhang , Hanping Zhang , Peng Xie . 2023. “Gut Microbiota and Its Metabolites in Depression: From Pathogenesis to Treatment.” EBioMedicine 90: 104527. 10.1016/j.ebiom.2023.104527 36963238 PMC10051028

[384] Li, Xinpei , Shijie Shang , Meng Wu , Qian Song , Dawei Chen . 2024. “Gut Microbial Metabolites in Lung Cancer Development and Immunotherapy: Novel Insights Into Gut‐Lung Axis.” Cancer Letters 598: 217096. 10.1016/j.canlet.2024.217096 38969161

[385] Koh, Ara , Filipe De Vadder , Petia Kovatcheva‐Datchary , Fredrik Bäckhed . 2016. “From Dietary Fiber to Host Physiology: Short‐Chain Fatty Acids as Key Bacterial Metabolites.” Cell 165: 1332–1345. 10.1016/j.cell.2016.05.041 27259147

[386] Jia, Dingjiacheng , Zheng Kuang , Liangjing Wang . 2024. “The Role of Microbial Indole Metabolites in Tumor.” Gut Microbes 16: 2409209. 10.1080/19490976.2024.2409209 39353090 PMC11445886

[387] Descamps, Hélène C. , Beatrice Herrmann , Daphne Wiredu , Christoph?A. Thaiss . 2019. “The Path Toward Using Microbial Metabolites as Therapies.” EBioMedicine 44: 747–754. 10.1016/j.ebiom.2019.05.063 31201140 PMC6606739

[388] Qiu, Zilin , Zhengrui Li , Cangang Zhang , Qun Zhao , Zaoqu Liu , Quan Cheng , Jian Zhang , Anqi Lin , Peng Luo . 2025. “NK Cell Senescence in Cancer: From Molecular Mechanisms to Therapeutic Opportunities.” Aging and Disease. 10.14336/AD.2025.0053 PMC12834417 40249925

[389] Wang, Zeyu , Ziyu Dai , Hao Zhang , Nan Zhang , Xisong Liang , Luo Peng , Jian Zhang , et al. 2023. “Comprehensive Analysis of Pyroptosis‐Related Gene Signatures for Glioblastoma Immune Microenvironment and Target Therapy.” Cell Proliferation 56: e13376. 10.1111/cpr.13376 36681858 PMC9977674

[390] Huang, Lihaoyun , Cangang Zhang , Aimin Jiang , Anqi Lin , Lingxuan Zhu , Weiming Mou , Dongqiang Zeng , et al. 2025. “T‐Cell Senescence in the Tumor Microenvironment.” Cancer Immunology Research 13: 618–632. 10.1158/2326-6066.CIR-24-0894 40232041

[391] Lin, Anqi , Ting Wei , Hui Meng , Peng Luo , Jian Zhang . 2019. “Role of the Dynamic Tumor Microenvironment in Controversies Regarding Immune Checkpoint Inhibitors for the Treatment of Non‐Small Cell Lung Cancer (NSCLC) With EGFR Mutations.” Molecular Cancer 18: 139. 10.1186/s12943-019-1062-7 31526368 PMC6745797

[392] Ma, Jiayao , Lingjuan Huang , Die Hu , Shan Zeng , Ying Han , Hong Shen . 2021. “The Role of the Tumor Microbe Microenvironment in the Tumor Immune Microenvironment: Bystander, Activator, or Inhibitor?” Journal of Experimental & Clinical Cancer Research: CR 40: 327. 10.1186/s13046-021-02128-w 34656142 PMC8520212

[393] Li, Yu , Cangang Zhang , Aimin Jiang , Anqi Lin , Zaoqu Liu , Xiangshu Cheng , Wanting Wang , et al. 2024. “Potential Anti‐Tumor Effects of Regulatory T Cells in the Tumor Microenvironment: A Review.” Journal of Translational Medicine 22: 293. 10.1186/s12967-024-05104-y 38509593 PMC10953261

[394] Bender, Mackenzie J. , Alex C. McPherson , Catherine M. Phelps , Surya P. Pandey , Colin R. Laughlin , Jake H. Shapira , Luzmariel Medina Sanchez , et al. 2023. “Dietary Tryptophan Metabolite Released by Intratumoral *Lactobacillus reuteri* Facilitates Immune Checkpoint Inhibitor Treatment.” Cell 186: 1846–62.e26. 10.1016/j.cell.2023.03.011 37028428 PMC10148916

[395] Wang, Yan , Mingshuai Bai , Qifan Peng , Leping Li , Feng Tian , Ying Guo , Changqing Jing . 2024. “Angiogenesis, a Key Point in the Association of Gut Microbiota and Its Metabolites With Disease.” European Journal of Medical Research 29: 614. 10.1186/s40001-024-02224-5 39710789 PMC11664877

[396] Liu, Jiankun , Yuzhong Duan , Xiaoming Cheng , Xi Chen , Wei Xie , Haixia Long , Zhihua Lin , Bo Zhu . 2011. “IL‐17 Is Associated With Poor Prognosis and Promotes Angiogenesis via Stimulating VEGF Production of Cancer Cells in Colorectal Carcinoma.” Biochemical and Biophysical Research Communications 407: 348–354. 10.1016/j.bbrc.2011.03.021 21396350

[397] Abe, Shohei , Atsuhiro Masuda , Tomonori Matsumoto , Jun Inoue , Hirochika Toyama , Arata Sakai , Takashi Kobayashi , et al. 2024. “Impact of Intratumoral Microbiome on Tumor Immunity and Prognosis in Human Pancreatic Ductal Adenocarcinoma.” Journal of Gastroenterology 59: 250–262. 10.1007/s00535-023-02069-5 38242997 PMC10904450

[398] Zhang, Shuyue , Shuishen Zhang , Xiaofan Ma , Jing Zhan , Chuqing Pan , Huizhong Zhang , Xiuying Xie , Jing Wen , Xuan Xie . 2023. “Intratumoral Microbiome Impacts Immune Infiltrates in Tumor Microenvironment and Predicts Prognosis in Esophageal Squamous Cell Carcinoma Patients.” Frontiers in Cellular and Infection Microbiology 13: 1165790. 10.3389/fcimb.2023.1165790 37180444 PMC10174428

[399] Gao, Kan , Chun‐Long Mu , Aitak Farzi , Wei‐Yun Zhu . 2020. “Tryptophan Metabolism: A Link Between the Gut Microbiota and Brain.” Advances in Nutrition 11: 709–723. 10.1093/advances/nmz127 31825083 PMC7231603

[400] Peters, Marloes A. M. , Annemiek M. E. Walenkamp , Ido P. Kema , Coby Meijer , Elisabeth G. E. De Vries , Sjoukje F. Oosting . 2014. “Dopamine and Serotonin Regulate Tumor Behavior by Affecting Angiogenesis.” Drug Resistance Updates 17: 96–104. 10.1016/j.drup.2014.09.001 25269824

[401] Yang, Qiqing , Bin Wang , Qinghui Zheng , Heyu Li , Xuli Meng , Fangfang Zhou , Long Zhang . 2023. “A Review of Gut Microbiota‐Derived Metabolites in Tumor Progression and Cancer Therapy.” Advanced Science (Weinheim, Baden‐Wurttemberg, Germany) 10: e2207366. 10.1002/advs.202207366 36951547 PMC10214247

[402] Liu, Yali , Harry Cheuk‐Hay Lau , Jun Yu . 2023. “Microbial Metabolites in Colorectal Tumorigenesis and Cancer Therapy.” Gut Microbes 15: 2203968. 10.1080/19490976.2023.2203968 37095682 PMC10132243

[403] Hofmanová, J. , A. Vaculová , A. Lojek , A. Kozubík . 2005. “Interaction of Polyunsaturated Fatty Acids and Sodium Butyrate During Apoptosis in HT‐29 Human Colon Adenocarcinoma Cells.” European Journal of Nutrition 44: 40–51. 10.1007/s00394-004-0490-2 15309463

[404] Choi, Juliana K. , Samer A. Naffouje , Masahide Goto , Jing Wang , Konstantin Christov , David J. Rademacher , Albert Green , et al. 2023. “Cross‐Talk Between Cancer and *Pseudomonas aeruginosa* Mediates Tumor Suppression.” Communications Biology 6: 16. 10.1038/s42003-022-04395-5 36609683 PMC9823004

[405] Li, Ruomeng , Jing Li , Xikun Zhou . 2024. “Lung Microbiome: New Insights Into the Pathogenesis of Respiratory Diseases.” Signal Transduction and Targeted Therapy 9: 19. 10.1038/s41392-023-01722-y 38228603 PMC10791971

[406] Dickson, Robert P. , John R. Erb‐Downward , Fernando J. Martinez , Gary B. Huffnagle . 2016. “The Microbiome and the Respiratory Tract.” Annual Review of Physiology 78: 481–504. 10.1146/annurev-physiol-021115-105238 PMC4751994 26527186

[407] Łaniewski, Paweł , Zehra Esra Ilhan , Melissa M. Herbst‐Kralovetz . 2020. “The Microbiome and Gynaecological Cancer Development, Prevention and Therapy.” Nature Reviews Urology 17: 232–250. 10.1038/s41585-020-0286-z 32071434 PMC9977514

[408] Markowski, Mark C. , Stephen A. Boorjian , Jeremy P. Burton , Noah M. Hahn , Molly A. Ingersoll , Saman Maleki Vareki , Sumanta K. Pal , Karen S. Sfanos . 2019. “The Microbiome and Genitourinary Cancer: A Collaborative Review.” European Urology 75: 637–646. 10.1016/j.eururo.2018.12.043 30655087 PMC9774685

[409] Scharschmidt, Tiffany C. , Julia A. Segre . 2025. “Skin Microbiome and Dermatologic Disorders.” The Journal of Clinical Investigation 135: e184315. 10.1172/JCI184315 39895627 PMC11785926

[410] Boxberger, Manon , Valérie Cenizo , Nadim Cassir , Bernard La Scola . 2021. “Challenges in Exploring and Manipulating the Human Skin Microbiome.” Microbiome 9: 125. 10.1186/s40168-021-01062-5 34053468 PMC8166136

[411] Sedghi, Lea , Vincent DiMassa , Anthony Harrington , Susan V. Lynch , Yvonne L. Kapila . 2021. “The Oral Microbiome: Role of Key Organisms and Complex Networks in Oral Health and Disease.” Periodontology 2000 87: 107–131. 10.1111/prd.12393 34463991 PMC8457218

[412] Lim, Yenkai , Makrina Totsika , Mark Morrison , Chamindie Punyadeera . 2017. “Oral Microbiome: A New Biomarker Reservoir for Oral and Oropharyngeal Cancers.” Theranostics 7: 4313–4321. 10.7150/thno.21804 29158828 PMC5695015

[413] Yang, Zhixin , Shiyu Zhang , Ning Ji , Jing Li , Qianming Chen . 2024. “The Evil Companion of OSCC: *Candida albicans* .” Oral Diseases 30: 1873–1886. 10.1111/odi.14700 37530513

[414] Diwan, Prerna , Mohit Nirwan , Mayank Bahuguna , Shashi Prabha Kumari , James Wahlang , Rakesh Kumar Gupta . 2023. “Evaluating Alterations of the Oral Microbiome and Its Link to Oral Cancer among Betel Quid Chewers: Prospecting Reversal Through Probiotic Intervention.” Pathogens (Basel, Switzerland) 12: 996. 10.3390/pathogens12080996 37623956 PMC10459687

[415] Karpiński, Tomasz M. 2019. “Role of Oral Microbiota in Cancer Development.” Microorganisms 7: 20. 10.3390/microorganisms7010020 30642137 PMC6352272

[416] Saikia, Partha Jyoti , Lekhika Pathak , Shirsajit Mitra , Bikul Das 2023. “The Emerging Role of Oral Microbiota in Oral Cancer Initiation, Progression and Stemness.” Frontiers in Immunology 14: 1198269. 10.3389/fimmu.2023.1198269 37954619 PMC10639169

[417] Ramirez‐Garcia, Andoni , Aitor Rementeria , Jose Manuel Aguirre‐Urizar , Maria Dolores Moragues , Aitziber Antoran , Aize Pellon , Ana Abad‐Diaz‐de‐Cerio , Fernando Luis Hernando . 2016. “ *Candida albicans* and Cancer: Can This Yeast Induce Cancer Development or Progression?” Critical Reviews in Microbiology 42: 181–193. 10.3109/1040841X.2014.913004 24963692

[418] Arzmi, Mohd Hafiz , Stuart Dashper , Michael McCullough . 2019. “Polymicrobial Interactions of *Candida albicans* and Its Role in Oral Carcinogenesis.” Journal of Oral Pathology & Medicine: Official Publication of the International Association of Oral Pathologists and the American Academy of Oral Pathology 48: 546–551. 10.1111/jop.12905 31183906

[419] Madhusudhan, Nandhitha , Manuela R. Pausan , Bettina Halwachs , Marija Durdević , Markus Windisch , Jan Kehrmann , VijayKumar Patra , et al. 2020. “Molecular Profiling of Keratinocyte Skin Tumors Links *Staphylococcus aureus* Overabundance and Increased Human β‐Defensin‐2 Expression to Growth Promotion of Squamous Cell Carcinoma.” Cancers 12: 541. 10.3390/cancers12030541 32111012 PMC7139500

[420] Nakatsuji, Teruaki , Tiffany H. Chen , Anna M. Butcher , Lynnie L. Trzoss , Sang‐Jip Nam , Karina T. Shirakawa , Wei Zhou , et al. 2018. “A Commensal Strain of *Staphylococcus epidermidis* Protects Against Skin Neoplasia.” Science Advances 4: eaao4502. 10.1126/sciadv.aao4502 29507878 PMC5834004

[421] Bhardwaj, Sanjeev K. , Harpreet Singh , Akash Deep , Madhu Khatri , Jayeeta Bhaumik , Ki‐Hyun Kim , Neha Bhardwaj . 2021. “UVC‐Based Photoinactivation as an Efficient Tool to Control the Transmission of Coronaviruses.” Science of The Total Environment 792: 148548. 10.1016/j.scitotenv.2021.148548 34465056 PMC8238411

[422] Patel, Tejas , L. Katie Morrison , Peter Rady , Stephen Tyring . 2010. “Epidermodysplasia Verruciformis and Susceptibility to HPV.” Disease Markers 29: 199–206. 10.1155/2010/345436 21178278 PMC3835378

[423] Hussein, Ahmed A. , Tariq A. Bhat , Zhe Jing , Eduardo Cortes Gomez , Mahmood Abdul Wasay , Prashant K. Singh , Song Liu , Gary Smith , Khurshid A. Guru . 2023. “Does the Urinary Microbiome Profile Change After Treatment of Bladder Cancer?” World Journal of Urology 41: 3593–3598. 10.1007/s00345-023-04627-1 37796319

[424] Prakash, Pranav , Shiv Verma , Sanjay Gupta . 2024. “Influence of Microbiome in Intraprostatic Inflammation and Prostate Cancer.” The Prostate 84: 1179–1188. 10.1002/pros.24756 38899408

[425] Li, Xiaoge , Jin Wu , Yutong Wu , Zhaoning Duan , Ming Luo , Ling Li , Sijing Li , Ying Jia . 2023. “Imbalance of Vaginal Microbiota and Immunity: Two Main Accomplices of Cervical Cancer in Chinese Women.” International Journal of Women's Health 15: 987–1002. 10.2147/IJWH.S406596 PMC10329453 37424699

[426] Kombe, Arnaud John Kombe Zoa‐Assoumou, Samira Bounda, Guy‐Armel Nsole‐Biteghe, Fleury‐Augustin Jin, Tengchuan Zouré, Abdou Azaque . 2023. “Advances in Etiopathological Role and Control of HPV in Cervical Cancer Oncogenesis.” Frontiers in Bioscience‐Landmark 28: 245. 10.31083/j.fbl2810245 37919078

[427] Zhang, Yong , Xiangxiang Chen , Yuan Wang , Ling Li , Qing Ju , Yan Zhang , Hangtian Xi , et al. 2023. “Alterations of Lower Respiratory Tract Microbiome and Short‐Chain Fatty Acids in Different Segments in Lung Cancer: A Multiomics Analysis.” Frontiers in Cellular and Infection Microbiology 13: 1261284. 10.3389/fcimb.2023.1261284 37915846 PMC10617678

[428] Bello, Salvador , José J. Vengoechea , Manuel Ponce‐Alonso , Ana L. Figueredo , Elisa Mincholé , Antonio Rezusta , Paula Gambó , et al. 2021. “Core Microbiota in Central Lung Cancer With Streptococcal Enrichment as a Possible Diagnostic Marker.” Archivos De Bronconeumologia 57: 681–689. 10.1016/j.arbres.2020.05.034 35699005

[429] Tsay, Jun‐Chieh J. , Benjamin G. Wu , Imran Sulaiman , Katherine Gershner , Rosemary Schluger , Yonghua Li , Ting‐An Yie , et al. 2021. “Lower Airway Dysbiosis Affects Lung Cancer Progression.” Cancer Discovery 11: 293–307. 10.1158/2159-8290.CD-20-0263 33177060 PMC7858243

[430] Apopa, Patrick L. , Lisa Alley , Rosalind B. Penney , Konstantinos Arnaoutakis , Mathew A. Steliga , Susan Jeffus , Emine Bircan , et al. 2018. “PARP1 Is Up‐Regulated in Non‐Small Cell Lung Cancer Tissues in the Presence of the Cyanobacterial Toxin Microcystin.” Frontiers in Microbiology 9: 1757. 10.3389/fmicb.2018.01757 30127774 PMC6087756

[431] Lan, Zhou , Wei‐Jia Liu , Hao Cui , Ke‐Long Zou , Hao Chen , Yu‐Yue Zhao , Guang‐Tao Yu . 2023. “The Role of Oral Microbiota in Cancer.” Frontiers in Microbiology 14: 1253025. 10.3389/fmicb.2023.1253025 37954233 PMC10634615

[432] Drokow, Emmanuel Kwateng , Clement Yaw Effah , Clement Agboyibor , Jemima Twumwaah Budu , Francisca Arboh , Priscilla Akyaa Kyei‐Baffour , Yao Xiao , Fan Zhang , Irene Xy Wu . 2023. “Microbial Infections as Potential Risk Factors for Lung Cancer: Investigating the Role of Human Papillomavirus and *Chlamydia pneumoniae* .” AIMS Public Health 10: 627–646. 10.3934/publichealth.2023044 37842273 PMC10567973

[433] Aitmanaitė, Lina , Karolis Širmonaitis , Giancarlo Russo . 2023. “Microbiomes, Their Function, and Cancer: How Metatranscriptomics Can Close the Knowledge Gap.” International Journal of Molecular Sciences 24: 13786. 10.3390/ijms241813786 37762088 PMC10531294

[434] Actis, Silvia , Massimiliano Cazzaniga , Valentina Elisabetta Bounous , Marta D'Alonzo , Roberta Rosso , Francesca Accomasso , Carola Minella , Nicoletta Biglia . 2023. “Emerging Evidence on the Role of Breast Microbiota on the Development of Breast Cancer in High‐Risk Patients.” Carcinogenesis 44: 718–725. 10.1093/carcin/bgad071 37793149

[435] Xuan, Caiyun , Jaime M. Shamonki , Alice Chung , Maggie L. Dinome , Maureen Chung , Peter A. Sieling , Delphine J. Lee . 2014. “Microbial Dysbiosis Is Associated With Human Breast Cancer.” PloS One 9: e83744. 10.1371/journal.pone.0083744 24421902 PMC3885448

[436] Colbert, Lauren E. , Molly B. El Alam , Rui Wang , Tatiana Karpinets , David Lo , Erica J. Lynn , Timothy A. Harris , et al. 2023. “Tumor‐Resident *Lactobacillus iners* Confer Chemoradiation Resistance Through Lactate‐Induced Metabolic Rewiring.” Cancer Cell 41: 1945–62.e11. 10.1016/j.ccell.2023.09.012 37863066 PMC10841640

[437] Jian, Xingxing , Yinghong Zhu , Jian Ouyang , Yihui Wang , Qian Lei , Jiliang Xia , Yongjun Guan , et al. 2020. “Alterations of Gut Microbiome Accelerate Multiple Myeloma Progression by Increasing the Relative Abundances of Nitrogen‐Recycling Bacteria.” Microbiome 8: 74. 10.1186/s40168-020-00854-5 32466801 PMC7257554

[438] Zheng, Xiao‐Hui , Xi‐Zhao Li , Cao‐Li Tang , Yu‐Meng Zhang , Ting Zhou , Xiao‐Jing Yang , Ying Liao , et al. 2024. “Detection of Epstein‒Barr Virus DNA Methylation as Tumor Markers of Nasopharyngeal Carcinoma Patients in Saliva, Oropharyngeal Swab, Oral Swab, and Mouthwash.” MedComm 5: e673. 10.1002/mco2.673 39161799 PMC11331033

[439] Muñoz‐Grez, Camila Paz , Mabel Angélica Vidal , Tamara Beatriz Rojas , Luciano Esteban Ferrada , Felipe Andrés Zuñiga , Agustin Andrés Vera , Sergio Andrés Sanhueza , et al. 2025. “Host‐Microbe Computational Proteomic Landscape in Oral Cancer Revealed Key Functional and Metabolic Pathways Between *Fusobacterium nucleatum* and Cancer Progression.” International Journal of Oral Science 17, 1: 1. 10.1038/s41368-024-00326-8 39743544 PMC11693762

[440] Wang, Xiu‐Li , Hua‐Wen Xu , Ning‐Ning Liu . 2023. “Oral Microbiota: A New Insight into Cancer Progression, Diagnosis and Treatment.” Phenomics 3: 535–547. 10.1007/s43657-023-00124-y 37881320 PMC10593652

[441] Anjali, Kanadan , Muhammed Manzoor , Mangesh Vasant Suryavanshi , Parthiban Rudrapathy , Punchappady Devasya Rekha , Ranajit Das , Asif Hameed , Ananthapadmanabha Bhagwath Arun . 2023. “Dysbiosis of the Oral Microbiota Composition Is Associated With Oral Squamous Cell Carcinoma and the Impact of Radiotherapy: A Pilot Study.” FEMS Microbiology Letters 370: fnad111. 10.1093/femsle/fnad111 37881017

[442] Mauceri, Rodolfo , Martina Coppini , Davide Vacca , Giorgio Bertolazzi , Valeria Cancila , Claudio Tripodo , Giuseppina Campisi . 2023. “No Clear Clustering Dysbiosis From Salivary Microbiota Analysis by Long Sequencing Reads in Patients Affected by Oral Squamous Cell Carcinoma: A Single Center Study.” Cancers 15: 4211. 10.3390/cancers15174211 37686487 PMC10486367

[443] Han, Zewen , Yichen Hu , Xin Lin , Hongyu Cheng , Biao Dong , Xuan Liu , Buling Wu , Zhenjiang Zech Xu . 2025. “Systematic Analyses Uncover Robust Salivary Microbial Signatures and Host–Microbiome Perturbations in Oral Squamous Cell Carcinoma.” mSystems 10: e0124724. 10.1128/msystems.01247-24 39873508 PMC11834404

[444] Wang, Zizheng , Yilong Chen , Haoning Li , Yuan Yue , Haopeng Yu . 2025. “Exploring Oral Microbiome in Oral Squamous Cell Carcinoma Across Environment‐Associated Sample Types.” Microbiology Spectrum 13: e0085224. 10.1128/spectrum.00852-24 40013780 PMC11960067

[445] Li, Shuluan , Tianyu Wang , Ya Ren , Zhou Liu , Jidong Gao , Zhi Guo . 2024. “Prognostic Impact of Oral Microbiome on Survival of Malignancies: A Systematic Review and Meta‐Analysis.” Systematic Reviews 13: 41. 10.1186/s13643-023-02419-7 38273347 PMC10809532

[446] Monson, Kelsey R. , Brandilyn A. Peters , Mykhaylo Usyk , Caroline Y. Um , Paul E. Oberstein , Marjorie L. McCullough , Mark P. Purdue , et al. 2022. “Elevated Dietary Carbohydrate and Glycemic Intake Associate With an Altered Oral Microbial Ecosystem in Two Large U.S. Cohorts.” Cancer Research Communications 2: 1558–1568. 10.1158/2767-9764.crc-22-0323 36567732 PMC9770587

[447] Menon, Anil , Vimi S. Mutalik , Yongqiang Chen , Spd Ponamgi , Sujatha Peela , Robert J. Schroth , Saeid Ghavami , Prashen Chelikani . 2025. “Beyond Genetics: Exploring Lifestyle, Microbiome, and Social Determinants in Oral Cancer Development.” Cancers 17: 1094. 10.3390/cancers17071094 40227635 PMC11988157

[448] Wang, Xue , Qili Mi , Ji Yang , Ying Guan , Wanli Zeng , Haiying Xiang , Xin Liu , et al. 2022. “Effect of Electronic Cigarette and Tobacco Smoking on the Human Saliva Microbial Community.” Brazilian Journal of Microbiology 53: 991–1000. 10.1007/s42770-022-00721-5 35229279 PMC9151971

[449] Petersen, Poul Erik . 2009. “Oral Cancer Prevention and Control—The Approach of the World Health Organization.” Oral Oncology 45: 454–460. 10.1016/j.oraloncology.2008.05.023 18804412

[450] Al‐Hebshi, Nezar N. , Akram T. Nasher , David J. Speicher , Mushfiq H. Shaikh , Newell W. Johnson . 2016. “Possible Interaction Between Tobacco Use and EBV in Oral Squamous Cell Carcinoma.” Oral Oncology 59: e4–e5. 10.1016/j.oraloncology.2016.06.005 27338286

[451] Zhang, Yaobang , Pimporn Thongmuang , Sarisak Soontornchai . 2025. “Analysis on Risk Factors of Oral Cancer in Chengdu Community.” Proceeding National & International Conference 18: 204. http://www.journalgrad.ssru.ac.th/index.php/8thconference/article/view/5078

[452] Liu, Qiao‐Yun , Ying Liao , Yan‐Xia Wu , Hua Diao , Yan Du , Yi‐Wei Chen , Jin‐Ru Xie , et al. 2023. “The Oral Microbiome as Mediator Between Oral Hygiene and Its Impact on Nasopharyngeal Carcinoma.” Microorganisms 11: 719. 10.3390/microorganisms11030719 36985292 PMC10058307

[453] Conway, Erica , Haisheng Wu , Linwei Tian . 2023. “Overview of Risk Factors for Esophageal Squamous Cell Carcinoma in China.” Cancers 15: 5604. 10.3390/cancers15235604 38067307 PMC10705141

[454] La Rosa, Giusy Rita Maria , Giuseppe Gattuso, Eugenio Pedullà , Ernesto Rapisarda, Daria Nicolosi , Mario Salmeri . 2020. “Association of Oral Dysbiosis With Oral Cancer Development.” Oncology Letters 19: 3045–3058. 10.3892/ol.2020.11441 32211076 PMC7079586

[455] Salem, Abdelhakim , Rabeia Almahmoudi , Jaana Hagström , Holger Stark , Dan Nordström , Tuula Salo , Kari K. Eklund . 2019. “Human β‐Defensin 2 Expression in Oral Epithelium: Potential Therapeutic Targets in Oral Lichen Planus.” International Journal of Molecular Sciences 20: 1780. 10.3390/ijms20071780 30974892 PMC6479702

[456] Triarico, Silvia , Pierpaolo Agresti , Emanuele Rinninella , Maria Cristina Mele , Alberto Romano , Giorgio Attinà , Palma Maurizi , Stefano Mastrangelo , Antonio Ruggiero . 2022. “Oral Microbiota During Childhood and Its Role in Chemotherapy‐Induced Oral Mucositis in Children With Cancer.” Pathogens (Basel, Switzerland) 11: 448. 10.3390/pathogens11040448 35456122 PMC9025665

[457] Akbari, Elahe , Alireza Milani , Masoud Seyedinkhorasani , Azam Bolhassani . 2023. “HPV Co‐Infections With Other Pathogens in Cancer Development: A Comprehensive Review.” Journal of Medical Virology 95: e29236. 10.1002/jmv.29236 37997472

[458] Wallace, Nicholas A. , Sujita Khanal , Kristin L. Robinson , Sebastian O. Wendel , Joshua J. Messer , Denise A. Galloway . 2017. “High‐Risk Alphapapillomavirus Oncogenes Impair the Homologous Recombination Pathway.” Journal of Virology 91: e01084‐17. 10.1128/JVI.01084-17 28768872 PMC5625488

[459] Zhou, Chaozheng , Anqi Lin , Manming Cao , Weimin Ding , Weiming Mou , Ningyi Guo , Zhenyu Chen , Jian Zhang , Peng Luo . 2021. “Activation of the DDR Pathway Leads to the Down‐Regulation of the TGFβ Pathway and a Better Response to ICIs in Patients With Metastatic Urothelial Carcinoma.” Frontiers in Immunology 12: 634741. 10.3389/fimmu.2021.634741 34220801 PMC8253049

[460] Shi, Congqi , Kaiyu Qin , Anqi Lin , Aimin Jiang , Quan Cheng , Zaoqu Liu , Jian Zhang , Peng Luo . 2022. “The Role of DNA Damage Repair (DDR) System in Response to Immune Checkpoint Inhibitor (ICI) Therapy.” Journal of Experimental & Clinical Cancer Research: CR 41: 268. 10.1186/s13046-022-02469-0 36071479 PMC9450390

[461] Raudenská, Martina , Maria Bugajová , David Kalfeřt , Jan Plzák , Šubrt, Adam Tesařová, Petra Masařík, Michal . 2024. “The Interplay Between Microbiome and Host Factors in Pathogenesis and Therapy of Head and Neck Cancer.” Biochimica et Biophysica Acta (BBA)—Reviews on Cancer 1879: 189216. 10.1016/j.bbcan.2024.189216 39542383

[462] Stasiewicz, Mark , Tomasz M. Karpiński . 2022. “The Oral Microbiota and Its Role in Carcinogenesis.” Seminars in Cancer Biology 86: 633–642. 10.1016/j.semcancer.2021.11.002 34743032

[463] Dai, Zhujiang , Jingqiu Zhang , Qi Wu , Juan Chen , Jun Liu , Lu Wang , Chaowu Chen , et al. 2019. “The ROLE of Microbiota in the development of colorectal cancer.” International Journal of Cancer 145: 2032–2041. 10.1002/ijc.32017 30474116 PMC6899977

[464] Karati, Dipanjan , Dileep Kumar . 2024. “Molecular Insight Into the Apoptotic Mechanism of Cancer Cells: An Explicative Review.” Current Molecular Pharmacology 17: e18761429273223. 10.2174/0118761429273223231124072223 38389419

[465] Kumai, Takumi , Hirotaka Shinomiya , Hirofumi Shibata , Hideaki Takahashi , Toshihiro Kishikawa , Ryuhei Okada , Shigeharu Fujieda , Masafumi Sakashita . 2024. “Translational Research in Head and Neck Cancer: Molecular and Immunological Updates.” Auris, Nasus, Larynx 51: 391–400. 10.1016/j.anl.2023.08.006 37640594

[466] Zhou, Jiaying , Zixuan Hu , Lei Wang , Qinchao Hu , Zixu Chen , Tao Lin , Rui Zhou , et al. 2024. “Tumor‐Colonized *Streptococcus mutans* Metabolically Reprograms Tumor Microenvironment and Promotes Oral Squamous Cell Carcinoma.” Microbiome 12: 193. 10.1186/s40168-024-01907-9 39369210 PMC11452938

[467] Hayes, Conall , Claire L. Donohoe , Maria Davern , Noel E. Donlon . 2021. “The Oncogenic and Clinical Implications of Lactate Induced Immunosuppression in the Tumour Microenvironment.” Cancer Letters 500: 75–86. 10.1016/j.canlet.2020.12.021 33347908

[468] Zang, Wenli , Junchao Liu , Fengxue Geng , Dongjuan Liu , Shuwei Zhang , Yuchao Li , Yaping Pan . 2022. “Butyrate Promotes Oral Squamous Cell Carcinoma Cells Migration, Invasion and Epithelial‐Mesenchymal Transition.” PeerJ 10: e12991. 10.7717/peerj.12991 35223210 PMC8877342

[469] Barbour, Abdelahhad , Omnia Elebyary , Noah Fine , Morvarid Oveisi , Michael Glogauer . 2022. “Metabolites of the Oral Microbiome: Important Mediators of Multikingdom Interactions.” FEMS Microbiology Reviews 46: fuab039. 10.1093/femsre/fuab039 34227664

[470] Chattopadhyay, Indranil , Mukesh Verma , Madhusmita Panda . 2019. “Role of Oral Microbiome Signatures in Diagnosis and Prognosis of Oral Cancer.” Technology in Cancer Research & Treatment 18: 1533033819867354. 10.1177/1533033819867354 31370775 PMC6676258

[471] Chen, Yiyin Erin Tsao, Hensin . 2013. “The Skin Microbiome: Current Perspectives and Future Challenges.” Journal of the American Academy of Dermatology 69: 143–55.e3. 10.1016/j.jaad.2013.01.016 23489584 PMC3686918

[472] Skin Cancer and Ultraviolet‐Radiation Exposure in Different Jobs. 2023. The British Journal of Dermatology 188: e22. 10.1093/bjd/ljad020 36810594

[473] Wittlich, Marc , Stephan Westerhausen , Benjamin Strehl , Helmut Versteeg , Wiho Stöppelmann . 2023. “The GENESIS‐UV Study on Ultraviolet Radiation Exposure Levels in 250 Occupations to Foster Epidemiological and Legislative Efforts to Combat Nonmelanoma Skin Cancer.” The British Journal of Dermatology 188: 350–360. 10.1093/bjd/ljac093 36635210

[474] Pega, Frank , Natalie C. Momen , Kai N. Streicher , Maria Leon‐Roux , Subas Neupane , Mary K. Schubauer‐Berigan , Joachim Schüz , et al. 2023. “Global, Regional and National Burdens of Non‐Melanoma Skin Cancer Attributable to Occupational Exposure to Solar Ultraviolet Radiation for 183 Countries, 2000–2019: A Systematic Analysis From the WHO/ILO Joint Estimates of the Work‐Related Burden of Disease and Injury.” Environment International 181: 108226. 10.1016/j.envint.2023.108226 37945424

[475] Kullander, Johanna , Ola Forslund , Joakim Dillner . 2009. “ *Staphylococcus aureus* and Squamous Cell Carcinoma of the Skin.” Cancer Epidemiology, Biomarkers & Prevention: A Publication of the American Association for Cancer Research, Cosponsored by the American Society of Preventive Oncology 18: 472–478. 10.1158/1055-9965.EPI-08-0905 19155437

[476] Mrázek, Jakub , Chahrazed Mekadim , Petra Kučerová , Roman Švejstil , Hana Salmonová , Jitka Vlasáková , Renata Tarasová , Jana Čížková , Monika Červinková . 2019. “Melanoma‐Related Changes in Skin Microbiome.” Folia Microbiologica 64: 435–442. 10.1007/s12223-018-00670-3 30554379

[477] Mizuhashi, Satoru , Ikko Kajihara , Soichiro Sawamura , Hisashi Kanemaru , Katsunari Makino , Jun Aoi , Takamitsu Makino , et al. 2021. “Skin Microbiome in Acral Melanoma: Corynebacterium Is Associated With Advanced Melanoma.” The Journal of Dermatology 48: e15–e16. 10.1111/1346-8138.15633 33017068

[478] Zhu, Yuhang , Wanguo Liu , Mei Wang , Xu Wang , Sibo Wang . 2024. “Causal Roles of Skin Microbiota in Skin Cancers Suggested by Genetic Study.” Frontiers in Microbiology 15: 1426807. 10.3389/fmicb.2024.1426807 39161599 PMC11330880

[479] Takei, Itsuki , Soichiro Sawamura , Tselmeg Mijiddorj Myangat , Ikko Kajihara , Hisashi Kanemaru , Kayo Kashiwada‐Nakamura , Katsunari Makino , et al. 2021. “Clinical Significance of Skin Colonization of *Pseudomonas aeruginosa* in Cutaneous Squamous Cell Carcinoma.” The Journal of Dermatology 48: e581–e582. 10.1111/1346-8138.16148 34462950

[480] Voigt, Anita Y. , Akintunde Emiola , Jethro S. Johnson , Elizabeth S. Fleming , Hoan Nguyen , Wei Zhou , Kenneth Y. Tsai , Christine Fink , Julia Oh . 2022. “Skin Microbiome Variation With Cancer Progression in Human Cutaneous Squamous Cell Carcinoma.” Journal of Investigative Dermatology 142: 2773–82.e16. 10.1016/j.jid.2022.03.017 35390349 PMC9509417

[481] Xia, Chenglai , Jiyan Su , Can Liu , Zhikai Mai , Shuanghong Yin , Chuansheng Yang , Liwu Fu . 2023. “Human Microbiomes in Cancer Development and Therapy.” MedComm 4: e221. 10.1002/mco2.221 36860568 PMC9969057

[482] Rangwala, S. , K. Y. Tsai . 2011. “Roles of the Immune System in Skin Cancer.” The British Journal of Dermatology 165: 953–965. 10.1111/j.1365-2133.2011.10507.x 21729024 PMC3197980

[483] Yu, Zhen‐Wei , Min Zheng , Hua‐Yang Fan , Xin‐Hua Liang , Ya‐Ling Tang . 2024. “Ultraviolet (UV) Radiation: A Double‐Edged Sword in Cancer Development and Therapy.” Molecular Biomedicine 5: 49. 10.1186/s43556-024-00209-8 39417901 PMC11486887

[484] Yamada, Miko , Tarl W. Prow . 2020. “Physical Drug Delivery Enhancement for Aged Skin, UV Damaged Skin and Skin Cancer: Translation and Commercialization.” Advanced Drug Delivery Reviews 153: 2–17. 10.1016/j.addr.2020.04.008 32339593

[485] Lopes, Fabiana C. P. S. , Marc G. Sleiman , Kate Sebastian , Roxanne Bogucka , Elizabeth A. Jacobs , Adewole S. Adamson . 2021. “UV Exposure and the Risk of Cutaneous Melanoma in Skin of Color: A Systematic Review.” JAMA Dermatology 157: 213–219. 10.1001/jamadermatol.2020.4616 33325988

[486] Coohill, Thomas P. , Jose‐Luis Sagripanti . 2008. “Overview of the Inactivation by 254?nm Ultraviolet Radiation of Bacteria With Particular Relevance to Biodefense.” Photochemistry and Photobiology 84: 1084–1090. 10.1111/j.1751-1097.2008.00387.x 18627518

[487] Burns, Erin M. , Hana Ahmed , Prescilia N. Isedeh , Indermeet Kohli , William Van Der Pol , Abdullah Shaheen , Anum F. Muzaffar , et al. 2019. “Ultraviolet Radiation, Both UVA and UVB, Influences the Composition of the Skin Microbiome.” Experimental Dermatology 28: 136–141. 10.1111/exd.13854 30506967 PMC7394481

[488] Willmott, Thomas , Paul M. Campbell , Christopher E. M. Griffiths , Clare O'Connor , Michael Bell , Rachel E. B. Watson , Andrew J. McBain , Abigail K. Langton . 2023. “Behaviour and Sun Exposure in Holidaymakers Alters Skin Microbiota Composition and Diversity.” Frontiers in Aging 4: 1217635. 10.3389/fragi.2023.1217635 37614517 PMC10442491

[489] Azzimonti, Barbara , Chiara Ballacchino , Paola Zanetta , Marie Angele Cucci , Chiara Monge , Margherita Grattarola , Chiara Dianzani , Giuseppina Barrera , Stefania Pizzimenti . 2023. “Microbiota, Oxidative Stress, and Skin Cancer: An Unexpected Triangle.” Antioxidants (Basel, Switzerland) 12: 546. 10.3390/antiox12030546 36978794 PMC10045429

[490] Tang Hongbo , Ma Qingyu , Sang Yingbing , Mao Lidan , Liang Junqin , Kang Xiaojing . 2022. “Mechanisms Underlying Synergistic Induction and Promotion of Cutaneous Squamous Cell Carcinoma in Nude Mice by Ultraviolet Light and Human Papillomavirus 16 E6.” Chinese Journal of Dermatology 55: 982‐989. 10.35541/cjd.20220084

[491] Harden, Mallory E. , Karl Munger . 2017. “Human Papillomavirus Molecular Biology.” Mutation Research/Reviews in Mutation Research 772: 3–12. 10.1016/j.mrrev.2016.07.002 28528688 PMC5500221

[492] Rogers, Heather D. , Jennifer L. Macgregor , Kristin M. Nord , Stephen Tyring , Peter Rady , Danielle E. Engler , Marc E. Grossman . 2009. “Acquired Epidermodysplasia Verruciformis.” Journal of the American Academy of Dermatology 60: 315–320. 10.1016/j.jaad.2008.08.035 19150275

[493] Dupin, Nicolas . 2020. “Update on Oncogenesis and Therapy for Kaposi Sarcoma.” Current Opinion in Oncology 32: 122–128. 10.1097/CCO.0000000000000601 31815777

[494] Feng, Huichen , Masahiro Shuda , Yuan Chang , Patrick S. Moore . 2008. “Clonal Integration of a Polyomavirus in Human Merkel Cell Carcinoma.” Science 319: 1096–1100. 10.1126/science.1152586 18202256 PMC2740911

[495] Zhao, Qihang , Qiang Wang , Tengjiao Wang , Junfang Xu , Tingting Li , Qiuyan Liu , Qinghua Yao , Pin Wang . 2021. “Pattern Recognition Receptors (PRRs) in Macrophages Possess Prognosis and Immunotherapy Potential for Melanoma.” Frontiers in Immunology 12: 765615. 10.3389/fimmu.2021.765615 34858419 PMC8630683

[496] Vijay, Kumar . 2018. “Toll‐Like Receptors in Immunity and Inflammatory Diseases: Past, Present, and Future.” International Immunopharmacology 59: 391–412. 10.1016/j.intimp.2018.03.002 29730580 PMC7106078

[497] Hoste, Esther , Esther N. Arwert , Rohit Lal , Andrew P. South , Julio C. Salas‐Alanis , Dedee F. Murrell , Giacomo Donati , Fiona M. Watt . 2015. “Innate Sensing of Microbial Products Promotes Wound‐Induced Skin Cancer.” Nature Communications 6: 5932. 10.1038/ncomms6932 PMC4338544 25575023

[498] Severn, Morgan M. , Alexander R. Horswill . 2023. “ *Staphylococcus epidermidis* and Its Dual Lifestyle in Skin Health and Infection.” Nature Reviews Microbiology 21: 97–111. 10.1038/s41579-022-00780-3 36042296 PMC9903335

[499] Yu, Jinlei , Yang Luo , Zhenlai Zhu , Yufeng Zhou , Licheng Sun , Jixin Gao , Jinlv Sun , et al. 2019. “A Tryptophan Metabolite of the skin Microbiota Attenuates Inflammation in Patients With Atopic Dermatitis Through the Aryl Hydrocarbon Receptor.” Journal of Allergy and Clinical Immunology 143: 2108–2119.e12. 10.1016/j.jaci.2018.11.036 30578876

[500] Axelrod, Margaret L. , Douglas B. Johnson , Justin M. Balko . 2018. “Emerging Biomarkers for Cancer Immunotherapy Iin Melanoma.” Seminars in Cancer Biology 52: 207–215. 10.1016/j.semcancer.2017.09.004 28917578 PMC5851807

[501] Hoeijmakers, Lotte L. , Irene L. M. Reijers , Christian U. Blank . 2023. “Biomarker‐Driven Personalization of Neoadjuvant Immunotherapy in Melanoma.” Cancer Discovery 13: 2319–2338. 10.1158/2159-8290.CD-23-0352 37668337

[502] Long, Georgina V. , Alexander M. Menzies , Richard A. Scolyer . 2023. “Neoadjuvant Checkpoint Immunotherapy and Melanoma: The Time Is Now.” Journal of Clinical Oncology 41: 3236–3248. 10.1200/JCO.22.02575 37104746

[503] Ianiro, Gianluca . 2025. “Longitudinal Gut Microbiome Shifts Correlate With Clinical Outcomes of Immune Checkpoint Inhibitors‐Treated Melanoma: Time for Microbial Biomarkers in Oncology?” Gastroenterology 168: 177–178. 10.1053/j.gastro.2024.07.034 39094750

[504] Ballerini, Mattia , Serena Galiè , Punit Tyagi , Carlotta Catozzi , Hariam Raji , Amir Nabinejad , Angeli D. G. Macandog , et al. 2025, 9, 967–984. “A Gut‐on‐a‐Chip Incorporating Human Faecal Samples and Peristalsis Predicts Responses to Immune Checkpoint Inhibitors for Melanoma.” Nature Biomedical Engineering. 10.1038/s41551-024-01318-z PMC12176660 39939548

[505] Pinato, David J. , Daria Gramenitskaya , Daniel M. Altmann , Rosemary J. Boyton , Benjamin H. Mullish , Julian R. Marchesi , Mark Bower . 2019. “Antibiotic Therapy and Outcome From Immune‐Checkpoint Inhibitors.” Journal for Immunotherapy of Cancer 7: 287. 10.1186/s40425-019-0775-x 31694714 PMC6836427

[506] Chen, Y. Erin , Djenet Bousbaine , Alessandra Veinbachs , Katayoon Atabakhsh , Alex Dimas , Victor K. Yu , Aishan Zhao , et al. 2023. “Engineered Skin Bacteria Induce Antitumor T Cell Responses Against Melanoma.” Science 380: 203–210. 10.1126/science.abp9563 37053311 PMC12356174

[507] He, Mengying , Tao Yang , Yuhan Wang , Mengyuan Wang , Xingye Chen , Dawei Ding , Yiran Zheng , Huabing Chen . 2021. “Immune Checkpoint Inhibitor‐Based Strategies for Synergistic Cancer Therapy.” Advanced Healthcare Materials 10: e2002104. 10.1002/adhm.202002104 33709564

[508] Plaçais, Léo , Stéphane Dalle , Olivier Dereure , Sabiha Trabelsi , Sophie Dalac , Delphine Legoupil , Henri Montaudié , et al. 2022. “Risk of irAEs in Patients With Autoimmune Diseases Treated by Immune Checkpoint Inhibitors for Stage III or IV Melanoma: Results From a Matched Case‐Control Study.” Annals of the Rheumatic Diseases 81: 1445–1452. 10.1136/ard-2022-222186 35788496

[509] Pan, Catherina X. , Mofei Liu , Charles B. Lau , William C. Lau , Daniel Y. Kim , Shahin A. Saberi , Rachael Rowley , et al. 2024. “Histopathological Predictors of Immune‐Related Adverse Events Among Patients With Melanoma Treated With Immune Checkpoint Inhibitors.” Journal of the American Academy of Dermatology 90: 826–829. 10.1016/j.jaad.2023.11.037 38040339

[510] Otto, Grant . 2023. “Diverse Routes to Melanoma Metastasis and ICI Resistance.” Nature Cancer 4: 1643. 10.1038/s43018-023-00679-9 38102352

[511] Killock, David . 2020. “ICI for resected Stage IV Melanoma.” Nature Reviews Clinical Oncology 17: 450. 10.1038/s41571-020-0397-8 32472086

[512] Carlino, Matteo S. , James Larkin , Georgina V. Long . 2021. “Immune Checkpoint Inhibitors in Melanoma.” The Lancet 398: 1002–1014. 10.1016/S0140-6736(21)01206-X 34509219

[513] Yacouba, Abdourahamane , Maryam Tidjani Alou , Jean‐Christophe Lagier , Grégory Dubourg , Didier Raoult . 2022. “Urinary Microbiota and Bladder Cancer: A Systematic Review and a Focus on Uropathogens.” Seminars in Cancer Biology 86: 875–884. 10.1016/j.semcancer.2021.12.010 34979272

[514] Wahid, Mohd , Sajad A. Dar , Arshad Jawed , Raju Kumar Mandal , Naseem Akhter , Saif Khan , Farah Khan , et al. 2022. “Microbes in Gynecologic Cancers: Causes or Consequences and Therapeutic Potential.” Seminars in Cancer Biology 86: 1179–1189. 10.1016/j.semcancer.2021.07.013 34302959

[515] Lee, Seoho , Karen Sfanos , Nirmish Singla . 2025. “The Role of the Urinary Microbiome in Genitourinary Cancers.” Nature Reviews Urology 22: 544–561. 10.1038/s41585-025-01011-z 40082677

[516] Mansour, Bassel , Ádám Monyók , Márió Gajdács , Balázs Stercz , Nóra Makra , Kinga Pénzes , István Vadnay , Dóra Szabó , Eszter Ostorházi . 2022. “Bladder Tissue Microbiome Composition in Patients of Bladder Cancer or Benign Prostatic Hyperplasia and Related Human Beta Defensin Levels.” Biomedicines 10: 1758. 10.3390/biomedicines10071758 35885062 PMC9313236

[517] Yin, Zhaofa , Bohan Liu , Shijian Feng , Yushi He , Cai Tang , Pengan Chen , Xinyi Wang , Kunjie Wang . 2023. “A Large Genetic Causal Analysis of the Gut Microbiota and Urological Cancers: A Bidirectional Mendelian Randomization Study.” Nutrients 15: 4086. 10.3390/nu15184086 37764869 PMC10537765

[518] Porto, Joao G. , Maria Camila Suarez Arbelaez , Brandon Pena , Archan Khandekar , Ankur Malpani , Bruno Nahar , Sanoj Punnen , et al. 2023. “The Influence of the Microbiome on Urological Malignancies: A Systematic Review.” Cancers 15: 4984. 10.3390/cancers15204984 37894351 PMC10605095

[519] Shrestha, Eva , James R. White , Shu‐Han Yu , Ibrahim Kulac , Onur Ertunc , Angelo M. De Marzo , Srinivasan Yegnasubramanian , et al. 2018. “Profiling the Urinary Microbiome in Men With Positive Versus Negative Biopsies for Prostate Cancer.” Journal of Urology 199: 161–171. 10.1016/j.juro.2017.08.001 28797714 PMC5937117

[520] Qi, Xiaoyu , Jing Zhou , Xinyue Wang , Yan Shen , Yuxun Cao , Liangzi Jiang , Miaomiao Shen , et al. 2025. “HPV E6/E7‐Induced Acetylation of a Peptide Encoded by a Long Non‐Coding RNA Inhibits Ferroptosis to Promote the Malignancy of Cervical Cancer.” Advanced Science (Weinheim, Baden‐Wurttemberg, Germany) 12: e2414018. 10.1002/advs.202414018 39836502 PMC11905060

[521] Molina, Mariano A. , Renske D. M. Steenbergen , Anna Pumpe , Angelique N. Kenyon , Willem J. G. Melchers . 2024. “HPV Integration and Cervical Cancer: A Failed Evolutionary Viral Trait.” Trends in Molecular Medicine 30: 890–902. 10.1016/j.molmed.2024.05.009 38853085

[522] Dias Gonçalves Lima, Fernando , Mariano A. Molina . 2024. “Uncovering the HPV Types Causing Cervical Cancer.” Nature Microbiology 9: 2795–2796. 10.1038/s41564-024-01835-2 39390274

[523] Mitra, Anita , David A. MacIntyre , Julian R. Marchesi , Yun S. Lee , Phillip R. Bennett , Maria Kyrgiou . 2016. “The Vaginal Microbiota, Human Papillomavirus Infection and Cervical Intraepithelial Neoplasia: What Do We Know and Where Are We Going Next?” Microbiome 4: 58. 10.1186/s40168-016-0203-0 27802830 PMC5088670

[524] Wei, Zhen‐Tong , Hong‐Liang Chen , Chun‐Feng Wang , Gui‐Lian Yang , Shu‐Mei Han , Song‐Ling Zhang . 2020. “Depiction of Vaginal Microbiota in Women With High‐Risk Human Papillomavirus Infection.” Frontiers in Public Health 8: 587298. 10.3389/fpubh.2020.587298 33490017 PMC7820762

[525] Gillet, Evy , Joris Fa Meys , Hans Verstraelen , Carolyne Bosire , Philippe De Sutter , Marleen Temmerman , Davy Vanden Broeck . 2011. “Bacterial vaginosis Is Associated With Uterine Cervical Human Papillomavirus Infection: A Meta‐Analysis.” BMC Infectious Diseases 11: 10. 10.1186/1471-2334-11-10 21223574 PMC3023697

[526] Chen, Yulian , Xingdi Qiu , Wenjing Wang , Dong Li , Anyue Wu , Zubei Hong , Wen Di , Lihua Qiu . 2020. “Human Papillomavirus Infection and Cervical Intraepithelial Neoplasia Progression Are Associated With Increased Vaginal Microbiome Diversity in a Chinese Cohort.” BMC Infectious Diseases 20: 629. 10.1186/s12879-020-05324-9 32842982 PMC7449047

[527] Gong, Xiangjin , Hao Chi , Zhijia Xia , Guanhu Yang , Gang Tian . 2023. “Advances in HPV‐Associated Tumor Management: Therapeutic Strategies and Emerging Insights.” Journal of Medical Virology 95: e28950. 10.1002/jmv.28950 37465863

[528] Usyk, Mykhaylo , Christine P. Zolnik , Philip E. Castle , Carolina Porras , Rolando Herrero , Ana Gradissimo , Paula Gonzalez , et al. 2020. “Cervicovaginal Microbiome and Natural History of HPV in a Longitudinal Study.” PLoS Pathogens 16: e1008376. 10.1371/journal.ppat.1008376 32214382 PMC7098574

[529] Wu, Ke , Yaorong Li , Kangli Ma , Weiguang Zhao , Zhixian Yao , Zhong Zheng , Feng Sun , et al. 2024. “The Microbiota and Renal Cell Carcinoma.” Cellular Oncology (Dordrecht, Netherlands) 47: 397–413. 10.1007/s13402-023-00876-9 37878209 PMC12974013

[530] Jiang, Shuanghong , Shan Xie , Dan Lv , Pu Wang , Hanchang He , Ting Zhang , Youlian Zhou , et al. 2017. “Alteration of the Gut Microbiota in Chinese Population With Chronic Kidney Disease.” Scientific Reports 7: 2870. 10.1038/s41598-017-02989-2 28588309 PMC5460291

[531] Hurst, Rachel , Emma Meader , Abraham Gihawi , Ghanasyam Rallapalli , Jeremy Clark , Gemma L. Kay , Martyn Webb , et al. 2022. “Microbiomes of Urine and the Prostate Are Linked to Human Prostate Cancer Risk Groups.” European Urology Oncology 5: 412–419. 10.1016/j.euo.2022.03.006 35450835

[532] Marshall, Erin A. , Fernando S. L. Filho , Don D. Sin , Stephen Lam , Janice M. Leung , Wan L. Lam . 2022. “Distinct Bronchial Microbiome Precedes Clinical Diagnosis of Lung Cancer.” Molecular Cancer 21: 68. 10.1186/s12943-022-01544-6 35255902 PMC8900294

[533] Li, Bowen , Daoyun Wang , Chengye Zhang , Yadong Wang , Zhicheng Huang , Libing Yang , Huaxia Yang , et al. 2024. “Role of Respiratory System Microbiota in Development of Lung Cancer and Clinical Application.” iMeta 3: e232. 10.1002/imt2.232 39429871 PMC11488069

[534] Goto, Taichiro . 2022. “Microbiota and Lung Cancer.” Seminars in Cancer Biology 86: 1–10. 10.1016/j.semcancer.2022.07.006 35882258

[535] O'Shaughnessy, Megan , Orla Sheils , Anne‐Marie Baird . 2023. “The Lung Microbiome in COPD and Lung Cancer: Exploring the Potential of Metal‐Based Drugs.” International Journal of Molecular Sciences 24: 12296. 10.3390/ijms241512296 37569672 PMC10419288

[536] Tsay, Jun‐Chieh J. , Benjamin G. Wu , Michelle H. Badri , Jose C. Clemente , Nan Shen , Peter Meyn , Yonghua Li , et al. 2018. “Airway Microbiota Is Associated With Upregulation of the PI3K Pathway in Lung Cancer.” American Journal of Respiratory and Critical Care Medicine 198: 1188–1198. 10.1164/rccm.201710-2118OC 29864375 PMC6221574

[537] Gomes, Sílvia , Bruno Cavadas , Joana Catarina Ferreira , Patrícia Isabel Marques , Catarina Monteiro , Maria Sucena , Catarina Sousa , et al. 2019. “Profiling of Lung Microbiota Discloses Differences in Adenocarcinoma and Squamous Cell Carcinoma.” Scientific Reports 9: 12838. 10.1038/s41598-019-49195-w 31492894 PMC6731246

[538] Ashique, Sumel , Gabriele De Rubis , Ekta Sirohi , Neeraj Mishra , Mohd Rihan , Ashish Garg , Ruby‐Jean Reyes , et al. 2022. “Short Chain Fatty Acids: Fundamental Mediators of the Gut‐Lung Axis and Their Involvement in Pulmonary Diseases.” Chemico‐Biological Interactions 368: 110231. 10.1016/j.cbi.2022.110231 36288778

[539] Liu, Weici , Jingtong Xu , Zheshun Pi , Yundi Chen , Guanyu Jiang , Yuan Wan , Wenjun Mao . 2023. “Untangling the Web of Intratumor Microbiota in Lung Cancer.” Biochimica et Biophysica Acta (BBA)—Reviews on Cancer 1878: 189025. 10.1016/j.bbcan.2023.189025 37980944

[540] Yang, Junjie , Xiaofeng Mu , Ye Wang , Dequan Zhu , Jiaming Zhang , Cheng Liang , Chen, Bin , et al. 2018. “Dysbiosis of the Salivary Microbiome Is Associated With Non‐smoking Female Lung Cancer and Correlated With Immunocytochemistry Markers.” Frontiers in Oncology 8: 520. 10.3389/fonc.2018.00520 30524957 PMC6256243

[541] Czarnecka‐Chrebelska, Karolina H. , Jacek Kordiak , Ewa Brzeziańska‐Lasota , Dorota Pastuszak‐Lewandoska . 2023. “Respiratory Tract Oncobiome in Lung Carcinogenesis: Where Are We Now?” Cancers 15: 4935. 10.3390/cancers15204935 37894302 PMC10605430

[542] Otoshi, Takehiro , Tatsuya Nagano , Jonguk Park , Koji Hosomi , Tomoya Yamashita , Motoko Tachihara , Tokiko Tabata , et al. 2022. “The Gut Microbiome as a Biomarker of Cancer Progression Among Female Never‐Smokers With Lung Adenocarcinoma.” Anticancer Research 42: 1589–1598. 10.21873/anticanres.15633 35220256

[543] Gui, Qifeng , Hanyu Li , Ange Wang , Xinxiu Zhao , Zhongju Tan , Lufang Chen , Keying Xu , Chi Xiao . 2020. “The Association Between Gut Butyrate‐Producing Bacteria and Non‐Small‐Cell Lung Cancer.” Journal of Clinical Laboratory Analysis 34: e23318. 10.1002/jcla.23318 32227387 PMC7439349

[544] Upreti, Deepa , Susumu Ishiguro , Morgan Phillips , Ayaka Nakashima , Kengo Suzuki , Jeffrey Comer , Masaaki Tamura . 2023. “ *Euglena gracilis* Extract Protects From Tobacco Smoke Carcinogen‐Induced Lung Cancer by Altering Gut Microbiota Metabolome.” Integrative Cancer Therapies 22: 15347354231195323. 10.1177/15347354231195323 37646331 PMC10469252

[545] Shin, Yoonhwa , Sunhee Han , Juhui Kwon , Songhyun Ju , Tae Choi , Insug Kang , Sung Kim . 2023. “Roles of Short‐Chain Fatty Acids in Inflammatory Bowel Disease.” Nutrients 15: 4466. 10.3390/nu15204466 37892541 PMC10609902

[546] Xiong, Xin , Le‐Wei Zheng , Yu Ding , Yu‐Fei Chen , Yu‐Wen Cai , Lei‐Ping Wang , Liang Huang , et al. 2025. “Breast Cancer: Pathogenesis and Treatments.” Signal Transduction and Targeted Therapy 10: 49. 10.1038/s41392-024-02108-4 39966355 PMC11836418

[547] Chadha, Jatin , Deeptashree Nandi , Yama Atri , Alo Nag . 2021. “Significance of Human Microbiome in Breast Cancer: Tale of an Invisible and an Invincible.” Seminars in Cancer Biology 70: 112–127. 10.1016/j.semcancer.2020.07.010 32717337

[548] Thu, May Soe , Korn Chotirosniramit , Tanawin Nopsopon , Nattiya Hirankarn , Krit Pongpirul . 2023. “Human Gut, Breast, and Oral Microbiome in Breast Cancer: A Systematic Review and Meta‐Analysis.” Frontiers in Oncology 13: 1144021. 10.3389/fonc.2023.1144021 37007104 PMC10063924

[549] Tzeng, Alice , Naseer Sangwan , Margaret Jia , Chin‐Chih Liu , Karen S. Keslar , Erinn Downs‐Kelly , Robert L. Fairchild , et al. 2021. “Human Breast Microbiome Correlates With Prognostic Features and Immunological Signatures in Breast Cancer.” Genome Medicine 13: 60. 10.1186/s13073-021-00874-2 33863341 PMC8052771

[550] Parida, Sheetal , Julia L. Drewes . 2022. “Unwanted Passengers: Microbes Hitchiking in Breast Cancer Metastases.” Cell Host & Microbe 30: 875–877. 10.1016/j.chom.2022.05.010 35679824

[551] Hou, Ting , Xiaoling Huang , Jiahui Lai , Dongfang Zhou . 2025. “Intra‐Tumoral Bacteria in Breast Cancer and Intervention Strategies.” Advanced Drug Delivery Reviews 217: 115516. 10.1016/j.addr.2025.115516 39828126

[552] Arnone, Alana A. , Yu‐Ting Tsai , J. Mark Cline , Adam S. Wilson , Brian Westwood , Meghan E. Seger , Akiko Chiba , et al. 2025. “Endocrine‐Targeting Therapies Shift the Breast Microbiome to Reduce Estrogen Receptor‐α Breast Cancer Risk.” Cell Reports. Medicine 6: 101880. 10.1016/j.xcrm.2024.101880 39742868 PMC11866439

[553] Wang, Wenhui , Zihao Ou , Xixin Huang , Jingyu Wang , Qianbei Li , Minghui Wen , Lei Zheng . 2024. “Microbiota and Glioma: A New Perspective From Association to Clinical Translation.” Gut Microbes 16: 2394166. 10.1080/19490976.2024.2394166 39185670 PMC11352717

[554] Ge, Yanshan , Xinhui Wang , Yali Guo , Junting Yan , Aliya Abuduwaili , Kasimujiang Aximujiang , Jie Yan , Minghua Wu . 2021. “Gut Microbiota Influence Tumor Development and Alter Interactions With the Human Immune System.” Journal of Experimental & Clinical Cancer Research: CR 40: 42. 10.1186/s13046-021-01845-6 33494784 PMC7829621

[555] Chatterjee, Jit , Xuanhe Qi , Rui Mu , Xuanwei Li , Talia Eligator , Megan Ouyang , Stephanie L. Bozeman , et al. 2025. “Intestinal Bacteroides Drives Glioma Progression by Regulating CD8+ T Cell Tumor Infiltration.” Neuro‐Oncology 27(6): 1579–1593. 10.1093/neuonc/noaf024 PMC12309722 39868555

[556] Lin, Ben , Zhen Ye , Zhan Cao , Zhao Ye , Yifei Yu , Weiliang Jiang , Sichen Guo , et al. 2024. “Integrated Microbiome and Metabolome Analysis Reveals Hypothalamic‐Comorbidities Related Signatures in Craniopharyngioma.” Advanced Science (Weinheim, Baden‐Wurttemberg, Germany) 11: e2400684. 10.1002/advs.202400684 39225628 PMC11497089

[557] Chandra, Vidhi , Le, Li , Olivereen Le Roux , Yu Zhang , Rian M. Howell , Dhwani N. Rupani , Seyda Baydogan , et al. 2024. “Gut Epithelial Interleukin‐17 Receptor A Signaling Can Modulate Distant Tumors Growth Through Microbial Regulation.” Cancer Cell 42: 85–100.e6. 10.1016/j.ccell.2023.12.006 38157865 PMC11238637

[558] Mehrian‐Shai, Ruty , Juergen K. V. Reichardt , Curtis C. Harris , Amos Toren . 2019. “The Gut–Brain Axis, Paving the Way to Brain Cancer.” Trends in Cancer 5: 200–207. 10.1016/j.trecan.2019.02.008 30961828 PMC6734924

[559] Zhu, Gengjun , Lifang Jin , Weizhang Shen , Meng Zhao , Ning Liu . 2023. “Intratumor Microbiota: Occult Participants in the Microenvironment of Multiple Myeloma.” Biochimica et Biophysica Acta (BBA)—Reviews on Cancer 1878: 188959. 10.1016/j.bbcan.2023.188959 37488050

[560] Shah, Urvi A. , Richa Parikh , Francesca Castro , Matteo Bellone , Alexander M. Lesokhin . 2023. “Dietary and Microbiome Evidence in Multiple Myeloma and Other Plasma Cell Disorders.” Leukemia 37: 964–980. 10.1038/s41375-023-01874-4 36997677 PMC10443185

[561] Fernandez Sanchez, Josaura , Arushana A. Maknojia , Katherine Y. King . 2024. “Blood and Guts: How the Intestinal Microbiome Shapes Hematopoiesis and Treatment of Hematologic Disease.” Blood 143: 1689–1701. 10.1182/blood.2023021174 38364184 PMC11103099

[562] Manzo, Veronica E. , Ami S. Bhatt . 2015. “The Human Microbiome in Hematopoiesis and Hematologic Disorders.” Blood 126: 311–318. 10.1182/blood-2015-04-574392 26012569 PMC4504946

[563] Manohar, Sonal M. 2024. “At the Crossroads of TNF α Signaling and Cancer.” Current Molecular Pharmacology 17: e060923220758. 10.2174/1874467217666230908111754 37691196

[564] Li, Guoqing , Yuxuan Wan , Alan Jiao , Ke Jiang , Gaoyuan Cui , Jinxin Tang , Simin Yu , et al. “Breaking Boundaries: Chronic Diseases and the Frontiers of Immune Microenvironments.” Med Research 1: 62–102. 10.1002/mdr2.70007

[565] Zhang, Sheng , Cheng Kong , Yongzhi Yang , Sanjun Cai , Xinxiang Li , Guoxiang Cai , Yanlei Ma . 2020. “Human Oral Microbiome Dysbiosis as a Novel Non‐Invasive Biomarker in Detection of Colorectal Cancer.” Theranostics 10: 11595–11606. 10.7150/thno.49515 33052235 PMC7545992

[566] Xian, Rena R. , Richard F. Ambinder . 2023. “Cell‐Free Circulating Tumor DNA and Epstein–Barr Virus DNA for Early Diagnosis of Epstein–Barr Virus‐Associated Cancers.” Journal of Clinical Oncology 41: 4290–4292. 10.1200/JCO.23.00687 37478392

[567] Wang, Ni , Jing‐Yuan Fang . 2023. “ *Fusobacterium nucleatum*, a Key Pathogenic Factor and Microbial Biomarker for Colorectal Cancer.” Trends in Microbiology 31: 159–172. 10.1016/j.tim.2022.08.010 36058786

[568] Li, Chiao‐Ling , Ming‐Chih Ho , You‐Yu Lin , Sheng‐Tai Tzeng , Yun‐Ju Chen , Hsin‐Yung Pai , Ya‐Chun Wang , et al. 2020. “Cell‐Free Virus‐Host Chimera DNA From Hepatitis B Virus Integration Sites as a Circulating Biomarker of Hepatocellular Cancer.” Hepatology 72: 2063–2076. 10.1002/hep.31230 32171027

[569] Hakozaki, Taiki , Kentaro Tanaka , Yoshimasa Shiraishi , Yuta Sekino , Noriko Mitome , Yusuke Okuma , Tomoiki Aiba , et al. 2025, 20, 912–927. “Gut Microbiota in Advanced NSCLC Receiving Chemoimmunotherapy: An Ancillary Biomarker Study From the Phase III Trial JCOG2007 (NIPPON).” Journal of Thoracic Oncology. 10.1016/j.jtho.2025.02.026 40058642

[570] Kwong, Thomas N. Y. , Xiansong Wang , Geicho Nakatsu , Tai Cheong Chow , Timothy Tipoe , Rudin Z. W. Dai , Kelvin K. K. Tsoi , et al. 2018. “Association Between Bacteremia From Specific Microbes and Subsequent Diagnosis of Colorectal Cancer.” Gastroenterology 155: 383–90.e8. 10.1053/j.gastro.2018.04.028 29729257

[571] Coker, Olabisi Oluwabukola , Changan Liu , William Ka Kei Wu , Sunny Hei Wong , Wei Jia , Joseph?J. Y. Sung , Jun Yu . 2022. “Altered Gut Metabolites and Microbiota Interactions Are Implicated in Colorectal Carcinogenesis and Can Be Non‐Invasive Diagnostic Biomarkers.” Microbiome 10: 35. 10.1186/s40168-021-01208-5 35189961 PMC8862353

[572] Zhang, Jia , Yangting He , Lu Xia , Jing Yi , Zhen Wang , Yingying Zhao , Xuemei Song , et al. 2022. “Expansion of Colorectal Cancer Biomarkers Based on Gut Bacteria and Viruses.” Cancers 14: 4662. 10.3390/cancers14194662 36230584 PMC9563090

[573] Zhao, Liuyang , Yu, Shi , Harry Cheuk‐Hay Lau , Weixin Liu , Guangwen Luo , Guoping Wang , Changan Liu , et al. 2022. “Uncovering 1058 Novel Human Enteric DNA Viruses Through Deep Long‐Read Third‐Generation Sequencing and Their Clinical Impact.” Gastroenterology 163: 699–711. 10.1053/j.gastro.2022.05.048 35679948

[574] Yunus, Aisyah , Norfilza Mohd Mokhtar , Raja Affendi Ali , Siti Maryam Ahmad Kendong , Hajar Fauzan Ahmad . 2024. “Methods for Identification of the Opportunistic Gut Mycobiome From Colorectal Adenocarcinoma Biopsy Tissues.” MethodsX 12: 102623. 10.1016/j.mex.2024.102623 38435637 PMC10907193

[575] Chen, Jijun , Siru Nie , Xunan Qiu , Shuwen Zheng , Chuxuan Ni , Yuan Yuan , Yuehua Gong . 2023. “Leveraging Existing 16S rRNA Microbial Data to Identify Diagnostic Biomarker in Chinese Patients With Gastric Cancer: A Systematic Meta‐Analysis.” mSystems 8: e0074723. 10.1128/msystems.00747-23 37787561 PMC10654077

[576] Xu, Shuo , Chunjie Xiang , Juan Wu , Yuhao Teng , Zhenfeng Wu , Ruiping Wang , Bin Lu , et al. 2021. “Tongue Coating Bacteria as a Potential Stable Biomarker for Gastric Cancer Independent of Lifestyle.” Digestive Diseases and Sciences 66: 2964–2980. 10.1007/s10620-020-06637-0 33044677

[577] Schinzari, V. , V. Barnaba , S. Piconese . 2015. “Chronic Hepatitis B Virus and Hepatitis C Virus Infections and Cancer: Synergy Between Viral and Host Factors.” Clinical Microbiology and Infection 21: 969–974. 10.1016/j.cmi.2015.06.026 26163104

[578] Han, Kathy , Jinfeng Zou , Zhen Zhao , Zeynep Baskurt , Yangqiao Zheng , Elizabeth Barnes , Jennifer Croke , et al. 2024. “Clinical Validation of Human Papilloma Virus Circulating Tumor DNA for Early Detection of Residual Disease After Chemoradiation in Cervical Cancer.” Journal of Clinical Oncology 42: 431–440. 10.1200/JCO.23.00954 37972346 PMC10824379

[579] Mendez, Roberto , Kousik Kesh , Nivedita Arora , Leá Di Martino , Florencia McAllister , Nipun Merchant , Sulagna Banerjee , Santanu Banerjee . 2020. “Microbial Dysbiosis and Polyamine Metabolism as Predictive Markers for Early Detection of Pancreatic Cancer.” Carcinogenesis 41: 561–570. 10.1093/carcin/bgz116 31369062 PMC7350554

[580] Yazdi, Hamid Reza , Abolfazl Movafagh , Fateme Fallah , Shohreh Alizadeh Shargh , Neda Mansouri , Atefeh Heidary Pour , Mehrdad Hashemi . 2016. “Evaluation of Methylobacterium Radiotolerance and *Sphyngomonas yanoikoaie* in Sentinel Lymph?Nodes of Breast Cancer Cases.” Asian Pacific Journal of Cancer Prevention 17: 279–285. 10.7314/apjcp.2016.17.s3.279 27165239

[581] Liang, Te , Hao Chen , Lei Liu , Yongqiang Zheng , Zhaoen Ma , Ling Min , Jiahui Zhang , et al. 2024. “Antibody Profiling of Pan‐Cancer Viral Proteome Reveals Biomarkers for Nasopharyngeal Carcinoma Diagnosis and Prognosis.” Molecular & Cellular Proteomics: MCP 23: 100729. 10.1016/j.mcpro.2024.100729 38309569 PMC10933552

[582] Wang, Na , Si Wu , Lanxiang Huang , Yue Hu , Xin He , Jourong He , Ben Hu , et al. 2025. “Intratumoral Microbiome: Implications for Immune Modulation and Innovative Therapeutic Strategies in Cancer.” Journal of Biomedical Science 32: 23. 10.1186/s12929-025-01117-x 39966840 PMC11837407

[583] Jiang, Zhengting , Wenjie Zhang , Zhilin Zhang , Gengyu Sha , Daorong Wang , Dong Tang . 2023. “Intratumoral Microbiota: A New Force in Diagnosing and Treating Pancreatic Cancer.” Cancer Letters 554: 216031. 10.1016/j.canlet.2022.216031 36481214

[584] Liu, Lanxiang , Haiyang Wang , Hanping Zhang , Xueyi Chen , Yangdong Zhang , Ji Wu , Libo Zhao , et al. 2022. “Toward a Deeper Understanding of Gut Microbiome in Depression: The Promise of Clinical Applicability.” Advanced Science (Weinheim, Baden‐Wurttemberg, Germany) 9: e2203707. 10.1002/advs.202203707 36285702 PMC9762301

[585] Kang, Xing , Harry Cheuk‐Hay Lau , Jun Yu . 2024. “Modulating Gut Microbiome in Cancer Immunotherapy: Harnessing Microbes to Enhance Treatment Efficacy.” Cell Reports. Medicine 5: 101478. 10.1016/j.xcrm.2024.101478 38631285 PMC11031381

[586] Han, Eui‐Jeong , Ji‐Seon Ahn , Yu‐Jin Choi , Da‐Hye Kim , Jong‐Soon Choi , Hea‐Jong Chung . 2025. “Exploring the Gut Microbiome: A Potential Biomarker for Cancer Diagnosis, Prognosis, and Therapy.” Biochimica et Biophysica Acta (BBA)—Reviews on Cancer 1880: 189251. 10.1016/j.bbcan.2024.189251 39719176

[587] Liu, Yali , Harry Cheuk‐Hay Lau , Wing Yin Cheng , Jun Yu . 2023. “Gut Microbiome in Colorectal Cancer: Clinical Diagnosis and Treatment.” Genomics, Proteomics & Bioinformatics 21: 84–96. 10.1016/j.gpb.2022.07.002 PMC10372906 35914737

[588] Zhang, Limin , Ziying Feng , Yinghua Li , Cuiting Lv , Chunchun Li , Yue Hu , Mingsheng Fu , Liang Song . 2023. “Salivary and Fecal Microbiota: Potential New Biomarkers for Early Screening of Colorectal Polyps.” Frontiers in Microbiology 14: 1182346. 10.3389/fmicb.2023.1182346 37655344 PMC10467446

[589] Che, Shusheng , Zhiyong Yan , Yugong Feng , Hai Zhao . 2024. “Unveiling the Intratumoral Microbiota Within Cancer Landscapes.” iScience 27: 109893. 10.1016/j.isci.2024.109893 38799560 PMC11126819

[590] Li, Xuebo , Xuelian Yuan , Xiumin Zhu , Changjun Li , Lei Ji , Kebo Lv , Geng Tian , Kang Ning , Jialiang Yang . 2023. “A Meta‐Analysis Of Tissue Microbial Biomarkers for Recurrence and Metastasis in Multiple Cancer Types.” Journal of Medical Microbiology 72. 10.1099/jmm.0.001744 37624368

[591] Ren, Zhigang , Ang Li , Jianwen Jiang , Lin Zhou , Zujiang Yu , Haifeng Lu , Haiyang Xie , et al. 2019. “Gut Microbiome Analysis as a Tool Towards Targeted Non‐Invasive Biomarkers for Early Hepatocellular Carcinoma.” Gut 68: 1014–1023. 10.1136/gutjnl-2017-315084 30045880 PMC6580753

[592] Cho, Eun Ju , Sangseob Leem , Sun Ah Kim , Jinho Yang , Yun Bin Lee , Soon Sun Kim , Jae Youn Cheong , et al. 2019. “Circulating Microbiota‐Based Metagenomic Signature for Detection of Hepatocellular Carcinoma.” Scientific Reports 9: 7536. 10.1038/s41598-019-44012-w 31101866 PMC6525191

[593] Loomba, Rohit , Victor Seguritan , Weizhong Li , Tao Long , Niels Klitgord , Archana Bhatt , Parambir Singh Dulai , et al. 2017. “Gut Microbiome‐Based Metagenomic Signature for Non‐invasive Detection of Advanced Fibrosis in Human Nonalcoholic Fatty Liver Disease.” Cell Metabolism 25: 1054–62.e5. 10.1016/j.cmet.2017.04.001 28467925 PMC5502730

[594] Liu, Ying , Hao Ai . 2024. “Comprehensive Insights Into Human Papillomavirus and Cervical Cancer: Pathophysiology, Screening, and Vaccination Strategies.” Biochimica et Biophysica Acta (BBA)—Reviews on Cancer 1879: 189192. 10.1016/j.bbcan.2024.189192 39349261

[595] Malpica, Luis , Mario L. Marques‐Piubelli , Brady E. Beltran , Julio C. Chavez , Roberto N. Miranda , Jorge J. Castillo . 2024. “EBV‐Positive Diffuse Large B‐Cell Lymphoma, Not Otherwise Specified: 2024 Update on the Diagnosis, Risk‐Stratification, and Management.” American Journal of Hematology 99: 2002–2015. 10.1002/ajh.27430 38957951

[596] Zoulim, Fabien , Pei‐Jer Chen , Maura Dandri , Patrick T. Kennedy , Christoph Seeger . 2024. “Hepatitis B Virus DNA Integration: Implications for Diagnostics, Therapy, and Outcome.” Journal of Hepatology 81: 1087–1099. 10.1016/j.jhep.2024.06.037 38971531

[597] Arbyn, Marc , Marie Simon , Eliana Peeters , Lan Xu , Chris J. L. M. Meijer , Johannes Berkhof , Kate Cuschieri , et al. 2021. “2020 List of Human Papillomavirus Assays Suitable for Primary Cervical Cancer Screening.” Clinical Microbiology and Infection 27: 1083–1095. 10.1016/j.cmi.2021.04.031 33975008

[598] Wolf, Jonas , Lucas Felipe Kist , Samanta Brangel Pereira , Marilze Alves Quessada , Helena Petek , Arthur Pille , Juçara Gasparetto Maccari , Mohamed Parrini Mutlaq , Luiz Antonio Nasi . 2024. “Human Papillomavirus Infection: Epidemiology, Biology, Host Interactions, Cancer Development, Prevention, and Therapeutics.” Reviews in Medical Virology 34: e2537. 10.1002/rmv.2537 38666757

[599] Emlet, Cade , Mack Ruffin , Regina Lamendella . 2020. “Enteric Virome and Carcinogenesis in the Gut.” Digestive Diseases and Sciences 65: 852–864. 10.1007/s10620-020-06126-4 32060814

[600] Zhang, Zhemei , Yongqing Yang , Lei Zhang , Yang Wu , Pengxia Jia , Qingmei Ma , Danni Wang . 2023. “Relationship Between Cervicovaginal Microecological Changes and HPV16/18 Infection and Cervical Cancer in Women of Childbearing Age.” Annals of Clinical and Laboratory Science 53: 825–834. 38182150

[601] Elbehi, Attia M. , R. I. Anu , Bene Ekine‐Afolabi , Elizabeth Cash . 2020. “Emerging Role of Immune Checkpoint Inhibitors and Predictive Biomarkers in Head and Neck Cancers.” Oral Oncology 109: 104977. 10.1016/j.oraloncology.2020.104977 32853912

[602] Xu, Jian‐Hua . 2013. “Hepatitis B or C Viral Infection and Risk of Pancreatic Cancer: A Meta‐Analysis of Observational Studies.” World Journal of Gastroenterology 19: 4234–4241. 10.3748/wjg.v19.i26.4234 23864789 PMC3710428

[603] Saftien, Aurelia , Jens Puschhof , Eran Elinav . 2023. “Fungi and Cancer.” Gut 72: 1410–1425. 10.1136/gutjnl-2022-327952 37147013

[604] Xu, Jing , Chunjie Xiang , Cong Zhang , Boqi Xu , Juan Wu , Ruiping Wang , Yaping Yang , et al. 2019. “Microbial Biomarkers of Common Tongue Coatings in Patients With Gastric Cancer.” Microbial Pathogenesis 127: 97–105. 10.1016/j.micpath.2018.11.051 30508628

[605] Kaźmierczak‐Siedlecka, Karolina , Aleš Dvořák , Marcin Folwarski , Agnieszka Daca , Katarzyna Przewłócka , Wojciech Makarewicz . 2020. “Fungal Gut Microbiota Dysbiosis and Its Role in Colorectal, Oral, and Pancreatic Carcinogenesis.” Cancers 12: 1326. 10.3390/cancers12051326 32455985 PMC7281455

[606] Chung, Li‐Min , Ji‐An Liang , Cheng‐Li Lin , Li‐Min Sun , Chia‐Hung Kao . 2017. “Cancer Risk in Patients With Candidiasis: A Nationwide Population‐Based Cohort Study.” Oncotarget 8: 63562–63573. 10.18632/oncotarget.18855 28969011 PMC5609943

[607] Kashyap, Sheetal , Soumya Pal , Gourav Chandan , Vipin Saini , Sasanka Chakrabarti , Neeraj K. Saini , Amit Mittal , et al. 2022. “Understanding the Cross‐Talk Between Human Microbiota and Gastrointestinal Cancer for Developing Potential Diagnostic and Prognostic Biomarkers.” Seminars in Cancer Biology 86: 643–651. 10.1016/j.semcancer.2021.04.020 33971261

[608] Malhotra, Pratibha , Ranjith Palanisamy , Jose A. Caparros‐Martin , Marco Falasca . 2023. “Bile Acids and Microbiota Interplay in Pancreatic Cancer.” Cancers 15: 3573. 10.3390/cancers15143573 37509236 PMC10377396

[609] Chalova, Petra , Anton Tazky , Ludovit Skultety , Lenka Minichova , Michal Chovanec , Sona Ciernikova , Peter Mikus , Juraj Piestansky . 2023. “Determination of Short‐Chain Fatty Acids as Putative Biomarkers of Cancer Diseases by Modern Analytical Strategies and Tools: A Review.” Frontiers in Oncology 13: 1110235. 10.3389/fonc.2023.1110235 37441422 PMC10334191

[610] Fang, Chuan‐Yin , Jung‐Sheng Chen , Bing‐Mu Hsu , Bashir Hussain , Jagat Rathod , Kuo‐Hsin Lee . 2021. “Colorectal Cancer Stage‐Specific Fecal Bacterial Community Fingerprinting of the Taiwanese Population and Underpinning of Potential Taxonomic Biomarkers.” Microorganisms 9: 1548. 10.3390/microorganisms9081548 34442626 PMC8401100

[611] Chen, Feng , Xudong Dai , Chang‐Chun Zhou , Ke‐Xin Li , Yu‐Juan Zhang , Xiao‐Ying Lou , Yuan‐Min Zhu , et al. 2022. “Integrated Analysis of the Faecal Metagenome and Serum Metabolome Reveals the Role of Gut Microbiome‐Associated Metabolites in the Detection of Colorectal Cancer and Adenoma.” Gut 71: 1315–1325. 10.1136/gutjnl-2020-323476 34462336 PMC9185821

[612] Liu, Bo , Yige Li , Lijun Suo , Wei Zhang , Hongyun Cao , Ruicai Wang , Jiahui Luan , et al. 2022. “Characterizing Microbiota and Metabolomics Analysis to Identify Candidate Biomarkers in Lung Cancer.” Frontiers in Oncology 12: 1058436. 10.3389/fonc.2022.1058436 36457513 PMC9705781

[613] Louis, Petra , Georgina L. Hold , Harry J. Flint . 2014. “The Gut Microbiota, Bacterial Metabolites and Colorectal Cancer.” Nature Reviews Microbiology 12: 661–672. 10.1038/nrmicro3344 25198138

[614] Hatta, Muhammad Nur Adam , Ezanee Azlina Mohamad Hanif , Siok‐Fong Chin , Teck Yew Low , Hui‐Min Neoh . 2023. “ *Parvimonas micra* Infection Enhances Proliferation, Wound Healing, and Inflammation of a Colorectal Cancer Cell Line.” Bioscience Reports 43: BSR20230609. 10.1042/BSR20230609 37218575 PMC10272962

[615] Ding, Ruojun , ShengYang Bertrand Lian , Yew Chong Tam , Choon Chiat Oh . 2024. “The Cutaneous Microbiome in Skin Cancer—A Systematic Review.” Journal Der Deutschen Dermatologischen Gesellschaft = Journal of the German Society of Dermatology: JDDG 22: 177–184. 10.1111/ddg.15294 38243841

[616] Wu, Peng , Guihao Zhang , Jie Zhao , Jiawei Chen , Yang Chen , Weina Huang , Jialei Zhong , Jiarong Zeng . 2018. “Profiling the Urinary Microbiota in Male Patients With Bladder Cancer in China.” Frontiers in Cellular and Infection Microbiology 8: 167. 10.3389/fcimb.2018.00167 29904624 PMC5990618

[617] Derosa, Lisa , Bertrand Routy , Andrew Maltez Thomas , Valerio Iebba , Gerard Zalcman , Sylvie Friard , Julien Mazieres , et al. 2022. “Intestinal *Akkermansia muciniphila* Predicts Clinical Response to PD‐1 Blockade in Patients With Advanced Non‐Small‐Cell Lung Cancer.” Nature Medicine 28: 315–324. 10.1038/s41591-021-01655-5 PMC9330544 35115705

[618] Liu, Yuting , Huiwen Wang , Haiyang Jiang , Zhumei Sun , Anyang Sun . 2023. “Alloprevotella Can be Considered as a Potential Oral Biomarker in Intestinal Metaphase of Gastric Patients.” Studies in Health Technology and Informatics 308: 155–167. 10.3233/SHTI230836 38007737

[619] Lee, Wei‐Hsiang , Hui‐Mei Chen , Shun‐Fa Yang , Chao Liang , Chih‐Yu Peng , Feng‐Mao Lin , Lo‐Lin Tsai , et al. 2017. “Bacterial Alterations in Salivary Microbiota and Their Association in Oral Cancer.” Scientific Reports 7: 16540. 10.1038/s41598-017-16418-x 29184122 PMC5705712

[620] Mai, Guoqin , Limei Chen , Ran Li , Quan Liu , Haoran Zhang , Yingfei Ma . 2019. “Common Core Bacterial Biomarkers of Bladder Cancer Based on Multiple Datasets.” BioMed Research International 2019: 4824909. 10.1155/2019/4824909 31321235 PMC6607711

[621] Law, Helen Ka Wai , Howard Chi Ho Yim . 2024. “Early Diagnosis of Cancer Using Circulating Microbial DNA.” Cell Reports. Medicine 5: 101502. 10.1016/j.xcrm.2024.101502 38631290 PMC11031418

[622] Kataria, Radhika , Saeed Shoaie , Anita Grigoriadis , Jonathan C. M. Wan . 2023. “Leveraging Circulating Microbial DNA for Early Cancer Detection.” Trends in Cancer 9: 879–882. 10.1016/j.trecan.2023.08.001 37659908 PMC10873208

[623] Dzutsev, Amiran , Giorgio Trinchieri . 2020. “Microbial DNA Signature in Plasma Enables Cancer Diagnosis.” Nature Reviews Clinical Oncology 17: 453–454. 10.1038/s41571-020-0391-1 32424197

[624] Xiao, Qian , Wei Lu , Xiangxing Kong , Yang W. Shao , Yeting Hu , Ao Wang , Hua Bao , et al. 2021. “Alterations of Circulating Bacterial DNA in Colorectal Cancer and Adenoma: A Proof‐of‐Concept Study.” Cancer Letters 499: 201–208. 10.1016/j.canlet.2020.11.030 33249197

[625] Ahn, Sun M. , Jason Y. K. Chan , Zhe Zhang , Hao Wang , Zubair Khan , Justin A. Bishop , William Westra , Wayne M. Koch , Joseph?A. Califano . 2014. “Saliva and Plasma Quantitative Polymerase Chain Reaction‐Based Detection and Surveillance of Human Papillomavirus‐Related Head and Neck Cancer.” JAMA Otolaryngology—Head & Neck Surgery 140: 846–854. 10.1001/jamaoto.2014.1338 25078109 PMC4313904

[626] Yang, Jinho , Hyo Eun Moon , Hyung Woo Park , Andrea McDowell , Tae‐Seop Shin , Young‐Koo Jee , Sungmin Kym , Sun Ha Paek , Yoon‐Keun Kim . 2020. “Brain Tumor Diagnostic Model and Dietary Effect Based on Extracellular Vesicle Microbiome Data in Serum.” Experimental & Molecular Medicine 52: 1602–1613. 10.1038/s12276-020-00501-x 32939014 PMC8080813

[627] Melo, Sonia A. , Linda B. Luecke , Christoph Kahlert , Agustin F. Fernandez , Seth T. Gammon , Judith Kaye , Valerie S. LeBleu , et al. 2015. “Glypican‐1 Identifies Cancer Exosomes and Detects Early Pancreatic Cancer.” Nature 523: 177–182. 10.1038/nature14581 26106858 PMC4825698

[628] Barteneva, Natasha S. , Yeldar Baiken , Elizaveta Fasler‐Kan , Kenneth Alibek , Sheng Wang , Natalia Maltsev , Eugene D. Ponomarev , et al. 2017. “Extracellular Vesicles in Gastrointestinal Cancer in Conjunction With Microbiota: On the Border of Kingdoms.” Biochimica et Biophysica Acta (BBA)—Reviews on Cancer 1868: 372–393. 10.1016/j.bbcan.2017.06.005 28669749

[629] Gilbert, Jack A. , Martin J. Blaser , J. Gregory Caporaso , Janet K. Jansson , Susan V. Lynch , Rob Knight . 2018. “Current Understanding of the Human Microbiome.” Nature Medicine 24: 392–400. 10.1038/nm.4517 PMC7043356 29634682

[630] Baron, Ellen Jo , J. Michael Miller , Melvin P. Weinstein , Sandra S. Richter , Peter H. Gilligan , Richard B. Thomson , Paul Bourbeau , et al. 2013. “A Guide to Utilization of the Microbiology Laboratory for Diagnosis of Infectious Diseases: 2013 Recommendations by the Infectious Diseases Society of America (IDSA) and the American Society for Microbiology (ASM)(a).” Clinical Infectious Diseases: An Official Publication of the Infectious Diseases Society of America 57: e22–e121. 10.1093/cid/cit278 23845951 PMC3719886

[631] Sánchez‐Romero, M. Isabel, Juan Manuel García‐Lechuz Moya, Juan José González López, Nieves Orta Mira . 2019. “Collection, Transport and General Processing of Clinical Specimens in Microbiology Laboratory.” Enfermedades Infecciosas Y Microbiologia Clinica (English Ed.) 37: 127–134. 10.1016/j.eimc.2017.12.002 29426791

[632] Bharti, Richa , Dominik G. Grimm . 2021. “Current Challenges and Best‐Practice Protocols for Microbiome Analysis.” Briefings in Bioinformatics 22: 178–193. 10.1093/bib/bbz155 31848574 PMC7820839

[633] Fabre, Valeria , Angelina Davis , Daniel J. Diekema , Bruno Granwehr , Mary K. Hayden , Christopher F. Lowe , Christopher D. Pfeiffer , et al. 2023. “Principles of Diagnostic Stewardship: A Practical Guide From the Society for Healthcare Epidemiology of America Diagnostic Stewardship Task Force.” Infection Control & Hospital Epidemiology 44: 178–185. 10.1017/ice.2023.5 36786646

[634] Derosa, Lisa , Bertrand Routy , Antoine Desilets , Romain Daillère , Safae Terrisse , Guido Kroemer , Laurence Zitvogel . 2021. “Microbiota‐Centered Interventions: The Next Breakthrough in Immuno‐Oncology?” Cancer Discovery 11: 2396–2412. 10.1158/2159-8290.CD-21-0236 34400407

[635] Guerrero‐Preston, Rafael , Filipa Godoy‐Vitorino , Anne Jedlicka , Arnold Rodríguez‐Hilario , Herminio González , Jessica Bondy , Fahcina Lawson , et al. 2016. “16S rRNA Amplicon Sequencing Identifies Microbiota Associated With Oral Cancer, Human Papilloma Virus Infection and Surgical Treatment.” Oncotarget 7: 51320–51334. 10.18632/oncotarget.9710 27259999 PMC5239478

[636] Ren, Shengnan , Lingxin Feng , Haoran Liu , Yuke Mao , Zhuang Yu . 2024. “Gut Microbiome Affects the Response to Immunotherapy in Non‐Small Cell Lung Cancer.” Thoracic Cancer 15: 1149–1163. 10.1111/1759-7714.15303 38572783 PMC11091776

[637] Lee, Pei‐Chang , Wu, Chi‐Jung Hung, Ya‐Wen Lee, Chieh Ju Chi, Chen‐Ta Lee, I‐Cheng .‐ Kuo Yu‐Lun , et al. 2022. “Gut Microbiota and Metabolites Associate With Outcomes of Immune Checkpoint Inhibitor‐Treated Unresectable Hepatocellular Carcinoma.” Journal for Immunotherapy of Cancer 10: e004779. 10.1136/jitc-2022-004779 35738801 PMC9226985

[638] Zhou, Xin , Lili Chen , Wanrun Lin , Wenxin Zheng , Huijuan Zhang , Feng Zhou . 2024. “Diagnostic and Prognostic Potential of the Intra‐Tumoral Microbiota Profile in HPV‐Independent Endocervical Adenocarcinoma.” Frontiers in Cellular and Infection Microbiology 14: 1440017. 10.3389/fcimb.2024.1440017 39220287 PMC11362085

[639] Dora, David , Glen J. Weiss , Zsolt Megyesfalvi , Gabriella Gállfy , Edit Dulka , Anna Kerpel‐Fronius , Judit Berta , et al. 2023. “Computed Tomography‐Based Quantitative Texture Analysis and Gut Microbial Community Signatures Predict Survival in Non‐Small Cell Lung Cancer.” Cancers 15: 5091. 10.3390/cancers15205091 37894458 PMC10605408

[640] Zhu, Chengpei , Chenchen Zhang , Shanshan Wang , Ziyu Xun , Dongya Zhang , Zhou Lan , Longhao Zhang , et al. 2024. “Characterizations of Multi‐Kingdom Gut Microbiota in Immune Checkpoint Inhibitor‐Treated Hepatocellular Carcinoma.” Journal for Immunotherapy of Cancer 12: e008686. 10.1136/jitc-2023-008686 38844407 PMC11163665

[641] Zhu, Xinhai , Ke Li , Guichao Liu , Ruan Wu , Yan Zhang , Siying Wang , Meng Xu , Ligong Lu , Peng Li . 2023. “Microbial Metabolite Butyrate Promotes Anti‐PD‐1 Antitumor Efficacy by Modulating T Cell Receptor Signaling of Cytotoxic CD8 T Cell.” Gut Microbes 15: 2249143. 10.1080/19490976.2023.2249143 37635362 PMC10464552

[642] Geller, Leore T. , Michal Barzily‐Rokni , Tal Danino , Oliver H. Jonas , Noam Shental , Deborah Nejman , Nancy Gavert , et al. 2017. “Potential Role of Intratumor Bacteria in Mediating Tumor Resistance to the Chemotherapeutic Drug Gemcitabine.” Science 357: 1156–1160. 10.1126/science.aah5043 28912244 PMC5727343

[643] Coutzac, Clélia , Jean‐Mehdi Jouniaux , Angelo Paci , Julien Schmidt , Domenico Mallardo , Atmane Seck , Vahe Asvatourian , et al. 2020. “Systemic Short Chain Fatty Acids Limit Antitumor Effect of CTLA‐4 Blockade in Hosts With Cancer.” Nature Communications 11: 2168. 10.1038/s41467-020-16079-x PMC7195489 32358520

[644] Wallace, Bret D. , Hongwei Wang , Kimberly T. Lane , John E. Scott , Jillian Orans , Ja Seol Koo , Madhukumar Venkatesh , et al. 2010. “Alleviating Cancer Drug Toxicity by Inhibiting a Bacterial Enzyme.” Science 330: 831–835. 10.1126/science.1191175 21051639 PMC3110694

[645] Richardson, Brianna N. , Jolinta Lin , Zachary S. Buchwald , Jinbing Bai . 2022. “Skin Microbiome and Treatment‐Related Skin Toxicities in Patients With Cancer: A Mini‐Review.” Frontiers in Oncology 12: 924849. 10.3389/fonc.2022.924849 35912217 PMC9334917

[646] Porcari, Serena , Nicolas Benech , Mireia Valles‐Colomer , Nicola Segata , Antonio Gasbarrini , Giovanni Cammarota , Harry Sokol , Gianluca Ianiro . 2023. “Key Determinants of Success in Fecal Microbiota Transplantation: From Microbiome to Clinic.” Cell Host & Microbe 31: 712–733. 10.1016/j.chom.2023.03.020 37167953

[647] Frey‐Furtado, Leonor , Inês Magalhães , Benedita Sampaio‐Maia , Maria João Azevedo . 2023. “Oral Microbiome Characterization in Oral Mucositis Patients—A Systematic Review.” Journal of Oral Pathology & Medicine: Official Publication of the International Association of Oral Pathologists and the American Academy of Oral Pathology 52: 911–918. 10.1111/jop.13492 37839408

[648] Gui, Q.‐F. , H.‐F. Lu , C.‐X. Zhang , Z.‐R. Xu , Y.‐H. Yang . 2015. “Well‐Balanced Commensal Microbiota Contributes to Anti‐Cancer Response in a Lung Cancer Mouse Model.” Genetics and Molecular Research 14: 5642–5651. 10.4238/2015.May.25.16 26125762

[649] Stolzenberg‐Solomon, Rachael Z. , Kevin W. Dodd , Martin J. Blaser , Jarmo Virtamo , Philip R. Taylor , Demetrius Albanes . 2003. “Tooth Loss, Pancreatic Cancer, and *Helicobacter pylori* .” The American Journal of Clinical Nutrition 78: 176–181. 10.1093/ajcn/78.1.176 12816788

[650] Farrell, James J. , Lei Zhang , Hui Zhou , David Chia , David Elashoff , David Akin , Bruce J. Paster , Kaumudi Joshipura , David T. W. Wong . 2012. “Variations of Oral Microbiota Are Associated With Pancreatic Diseases Including Pancreatic Cancer.” Gut 61: 582–588. 10.1136/gutjnl-2011-300784 21994333 PMC3705763

[651] Peters, Brandilyn A. , Richard B. Hayes , Chandra Goparaju , Christopher Reid , Harvey I. Pass , Jiyoung Ahn . 2019. “The Microbiome in Lung Cancer Tissue and Recurrence‐Free Survival.” Cancer Epidemiology, Biomarkers & Prevention: A Publication of the American Association for Cancer Research, Cosponsored by the American Society of Preventive Oncology 28: 731–740. 10.1158/1055-9965.EPI-18-0966 PMC6449216 30733306

[652] Hu, Min , Samuel Coleman , Muhammad Zaki Hidayatullah Fadlullah , Daniel Spakowicz , Christine H. Chung , Aik Choon Tan . 2023. “Deciphering the Tumor‐Immune‐Microbe Interactions in HPV‐Negative Head and Neck Cancer.” Genes 14: 1599. 10.3390/genes14081599 37628651 PMC10454300

[653] Garajová, Ingrid , Rita Balsano , Heling Wang , Francesco Leonardi , Elisa Giovannetti , Dongmei Deng , Godefridus J. Peters . 2021. “The Role of the Microbiome in Drug Resistance in Gastrointestinal Cancers.” Expert Review of Anticancer Therapy 21: 165–176. 10.1080/14737140.2021.1844007 33115280

[654] Wang, Feng , Qian Yin , Liang Chen , Mark M. Davis . 2018. “Bifidobacterium Can Mitigate Intestinal Immunopathology in the Context Of CTLA‐4 Blockade.” Proceedings of the National Academy of Sciences 115: 157–161. 10.1073/pnas.1712901115 PMC5776803 29255057

[655] Klufa, Jörg , Thomas Bauer , Buck Hanson , Craig Herbold , Philipp Starkl , Beate Lichtenberger , Dagmar Srutkova , et al. 2019. “Hair Eruption Initiates and Commensal Skin Microbiota Aggravate Adverse Events of Anti‐EGFR Therapy.” Science Translational Medicine 11: eaax2693. 10.1126/scitranslmed.aax2693 31826981

[656] Pal, Sumanta K. , Sierra M. Li , Xiwei Wu , Hanjun Qin , Marcin Kortylewski , JoAnn Hsu , Courtney Carmichael , Paul Frankel . 2015. “Stool Bacteriomic Profiling in Patients With Metastatic Renal Cell Carcinoma Receiving Vascular Endothelial Growth Factor‐Tyrosine Kinase Inhibitors.” Clinical Cancer Research: An Official Journal of the American Association for Cancer Research 21: 5286–5293. 10.1158/1078-0432.CCR-15-0724 26152743

[657] Bawaneh, Alaa , Adam S. Wilson , Nicole Levi , Marissa M. Howard‐McNatt , Akiko Chiba , David R. Soto‐Pantoja , Katherine L. Cook . 2022. “Intestinal Microbiota Influence Doxorubicin Responsiveness in Triple‐Negative Breast Cancer.” Cancers 14: 4849. 10.3390/cancers14194849 36230772 PMC9563306

[658] Geng, Hong‐Wei , Feng‐Yi Yin , Zhi‐Fa Zhang , Xu Gong , Yun Yang . 2021. “Butyrate Suppresses Glucose Metabolism of Colorectal Cancer Cells via GPR109a‐AKT Signaling Pathway and Enhances Chemotherapy.” Frontiers in Molecular Biosciences 8: 634874. 10.3389/fmolb.2021.634874 33855046 PMC8039130

[659] Geller, Leore T. , Ravid Straussman . 2018. “Intratumoral Bacteria May Elicit Chemoresistance by Metabolizing Anticancer Agents.” Molecular & Cellular Oncology 5: e1405139. 10.1080/23723556.2017.1405139 29404397 PMC5791857

[660] Wang, Xinyi , Xicai Sun , Jinjin Chu , Wenchang Sun , Shushan Yan , Yaowen Wang . 2023. “Gut Microbiota and Microbiota‐Derived Metabolites in Colorectal Cancer: Enemy or Friend.” World Journal of Microbiology & Biotechnology 39: 291. 10.1007/s11274-023-03742-w 37653349

[661] Zheng, Di‐Wei , Xue Dong , Pei Pan , Ke‐Wei Chen , Jin‐Xuan Fan , Si‐Xue Cheng , Xian‐Zheng Zhang . 2019. “Phage‐Guided Modulation of the Gut Microbiota of Mouse Models of Colorectal Cancer Augments Their Responses to Chemotherapy.” Nature Biomedical Engineering 3: 717–728. 10.1038/s41551-019-0423-2 31332342

[662] Lindel, Katja , Karl T. Beer , Jean Laissue , Richard H. Greiner , Daniel M. Aebersold . 2001. “Human Papillomavirus Positive Squamous Cell Carcinoma of the Oropharynx: A Radiosensitive Subgroup of Head and Neck Carcinoma.” Cancer 92: 805–813. 10.1002/1097-0142(20010815)92:4<805::AID-CNCR1386>3.0.CO;2-9 11550151

[663] Chen, Allen M. , Carol Felix , Pin‐Chieh Wang , Sophia Hsu , Vincent Basehart , Jordan Garst , Phillip Beron , et al. 2017. “Reduced‐Dose Radiotherapy for Human Papillomavirus‐Associated Squamous‐Cell Carcinoma of the Oropharynx: A Single‐Arm, Phase 2 Study.” The Lancet. Oncology 18: 803–811. 10.1016/S1470-2045(17)30246-2 28434660 PMC6488353

[664] Qu, Junxing , Zhiheng Sun , Chen Peng , Daoqian Li , Wenyue Yan , Zhen Xu , Yayi Hou , et al. 2021. “ *C. tropicalis* Promotes Chemotherapy Resistance in Colon Cancer Through Increasing Lactate Production to Regulate the Mismatch Repair System.” International Journal of Biological Sciences 17: 2756–2769. 10.7150/ijbs.59262 34345205 PMC8326116

[665] Kumpitsch, Christina , Christine Moissl‐Eichinger , Jakob Pock , Dietmar Thurnher , Axel Wolf . 2020. “Preliminary Insights Into the Impact of Primary Radiochemotherapy on the Salivary Microbiome in Head and Neck Squamous Cell Carcinoma.” Scientific Reports 10: 16582. 10.1038/s41598-020-73515-0 33024215 PMC7538973

[666] Shuwen, Han , Yang Xi , Pan Yuefen , Xu Jiamin , Qi Quan , Liao Haihong , Jiang Yizhen , Wu Wei . 2020. “Effects of Postoperative Adjuvant Chemotherapy and Palliative Chemotherapy on the Gut Microbiome in Colorectal Cancer.” Microbial Pathogenesis 149: 104343. 10.1016/j.micpath.2020.104343 32562813

[667] Panebianco, Concetta , Kaarel Adamberg , Madis Jaagura , Massimiliano Copetti , Andrea Fontana , Signe Adamberg , Kaia Kolk , et al. 2018. “Influence of Gemcitabine Chemotherapy on the Microbiota of Pancreatic Cancer Xenografted Mice.” Cancer Chemotherapy and Pharmacology 81: 773–782. 10.1007/s00280-018-3549-0 29473096

[668] Loman, B. R. , K. R. Jordan , B. Haynes , M. T. Bailey , L. M. Pyter . 2019. “Chemotherapy‐Induced Neuroinflammation Is Associated With Disrupted Colonic and Bacterial Homeostasis in Female Mice.” Scientific Reports 9: 16490. 10.1038/s41598-019-52893-0 31712703 PMC6848141

[669] Stringer, Andrea M. , Rachel J. Gibson , Richard M. Logan , Joanne M. Bowen , Ann S. J. Yeoh , Juliette Hamilton , Dorothy M. K. Keefe . 2009. “Gastrointestinal Microflora and Mucins May Play a Critical Role in the Development of 5‐Fluorouracil‐Induced Gastrointestinal Mucositis.” Exp Biol Med (Maywood) 234: 430–441. 10.3181/0810-RM-301 19176868

[670] Stojanovska, Vanesa , Rachel M. McQuade , Sarah Fraser , Monica Prakash , Shakuntla Gondalia , Rhian Stavely , Enzo Palombo , et al. 2018. “Oxaliplatin‐Induced Changes in Microbiota, TLR4+ Cells and Enhanced HMGB1 Expression in the Murine Colon.” PloS One 13: e0198359. 10.1371/journal.pone.0198359 29894476 PMC5997344

[671] Kaźmierczak‐Siedlecka, Karolina , Nikola Bulman , Paweł Ulasiński , Bartosz Kamil Sobocki , Karol Połom , Luigi Marano , Leszek Kalinowski , Karolina Skonieczna‐Żydecka . 2023. “Pharmacomicrobiomics of Cell‐Cycle Specific Anti‐Cancer Drugs—Is it a New Perspective for Personalized Treatment of Cancer Patients?” Gut Microbes 15: 2281017. 10.1080/19490976.2023.2281017 37985748 PMC10730203

[672] Chiba, Akiko , Alaa Bawaneh , Christine Velazquez , Kenysha Y. J. Clear , Adam S. Wilson , Marissa Howard‐McNatt , Edward A. Levine , et al. 2020. “Neoadjuvant Chemotherapy Shifts Breast Tumor Microbiota Populations to Regulate Drug Responsiveness and the Development of Metastasis.” Molecular Cancer Research: MCR 18: 130–139. 10.1158/1541-7786.MCR-19-0451 31628201 PMC9153322

[673] Forsgård, Richard A. , Vannina G. Marrachelli , Katri Korpela , Rafael Frias , Maria Carmen Collado , Riitta Korpela , Daniel Monleon , Thomas Spillmann , Pia Österlund . 2017. “Chemotherapy‐Induced Gastrointestinal Toxicity Is Associated With Changes in Serum and Urine Metabolome and Fecal Microbiota in Male Sprague‐Dawley Rats.” Cancer Chemotherapy and Pharmacology 80: 317–332. 10.1007/s00280-017-3364-z 28646338 PMC5532424

[674] Bowen, Joanne M. , Andrea M. Stringer , Rachel J. Gibson , Ann S. J. Yeoh , Sarah Hannam , Dorothy M. K. Keefe . 2007. “VSL#3 Probiotic Treatment Reduces Chemotherapy‐Induced Diarrhea and Weight Loss.” Cancer Biology & Therapy 6: 1449–1454. 10.4161/cbt.6.9.4622 17881902

[675] Shen, Shiqian , Grewo Lim , Zerong You , Weihua Ding , Peigen Huang , Chongzhao Ran , Jason Doheny , et al. 2017. “Gut Microbiota Is Critical for the Induction of Chemotherapy‐Induced Pain.” Nature Neuroscience 20: 1213–1216. 10.1038/nn.4606 28714953 PMC5575957

[676] Ramakrishna, Chandran , Jose Corleto , Paul M. Ruegger , Geoffrey D. Logan , Beth B. Peacock , Stacee Mendonca , Shanni Yamaki , et al. 2019. “Dominant Role of the Gut Microbiota in Chemotherapy Induced Neuropathic Pain.” Scientific Reports 9: 20324. 10.1038/s41598-019-56832-x 31889131 PMC6937259

[677] Li, Shiyu , Shuangli Zhu , Jun Yu . 2024. “The Role of Gut Microbiota and Metabolites in Cancer Chemotherapy.” Journal of Advanced Research 64: 223–235. 10.1016/j.jare.2023.11.027 38013112 PMC11464465

[678] Mirzaei, Sara , Milad Iranshahy , Hamid Gholamhosseinian , Maryam M. Matin , Fatemeh B. Rassouli . 2022. “Urolithins Increased Anticancer Effects of Chemical Drugs, Ionizing Radiation and Hyperthermia on Human Esophageal Carcinoma Cells In Vitro.” Tissue & Cell 77: 101846. 10.1016/j.tice.2022.101846 35679683

[679] Teng, Huajing , Yan Wang , Xin Sui , Jiawen Fan , Shuai Li , Xiao Lei , Chen Shi , et al. 2023. “Gut Microbiota‐Mediated Nucleotide Synthesis Attenuates the Response to Neoadjuvant Chemoradiotherapy in Rectal Cancer.” Cancer Cell 41: 124–38.e6. 10.1016/j.ccell.2022.11.013 36563680

[680] Linn, Ye Htut , K. Khine Thu , Nang Hla Hla Win . 2019. “Effect of Probiotics for the Prevention of Acute Radiation‐Induced Diarrhoea Among Cervical Cancer Patients: A Randomized Double‐Blind Placebo‐Controlled Study.” Probiotics and Antimicrobial Proteins 11: 638–647. 10.1007/s12602-018-9408-9 29550911

[681] Blake, Stephen J. , Yochai Wolf , Ben Boursi , Lynn, David J . 2024. “Role of the Microbiota in Response to and Recovery From Cancer Therapy.” Nature Reviews Immunology 24: 308–325. 10.1038/s41577-023-00951-0 37932511

[682] Li, Zongjuan , Yang Zhang , Weifeng Hong , Biao Wang , Yixing Chen , Ping Yang , Jian Zhou , et al. 2022. “Gut Microbiota Modulate Radiotherapy‐Associated Antitumor Immune Responses Against Hepatocellular Carcinoma via STING Signaling.” Gut Microbes 14: 2119055. 10.1080/19490976.2022.2119055 36093568 PMC9467592

[683] Shi, Wei , Lijun Shen , Wei Zou , Jingwen Wang , Jianing Yang , Yuezhu Wang , Bingdong Liu , et al. 2020. “The Gut Microbiome Is Associated With Therapeutic Responses and Toxicities of Neoadjuvant Chemoradiotherapy in Rectal Cancer Patients—A Pilot Study.” Frontiers in Cellular and Infection Microbiology 10: 562463. 10.3389/fcimb.2020.562463 33363048 PMC7756020

[684] Yi, Yuxi , Lijun Shen , Wei Shi , Fan Xia , Hui Zhang , Yan Wang , Jing Zhang , et al. 2021. “Gut Microbiome Components Predict Response to Neoadjuvant Chemoradiotherapy in Patients With Locally Advanced Rectal Cancer: A Prospective, Longitudinal Study.” Clinical Cancer Research: An Official Journal of the American Association for Cancer Research 27: 1329–1340. 10.1158/1078-0432.CCR-20-3445 33298472

[685] Chen, Linda , Nadeem Riaz , Nancy Lee , Sean McBride . 2021. “Current Considerations for Radiotherapy in HPV‐Associated Head and Neck Cancer.” Journal of Surgical Oncology 124: 945–951. 10.1002/jso.26689 34617275 PMC12206517

[686] Shiao, Stephen L. , Kathleen M. Kershaw , Jose J. Limon , Sungyong You , Junhee Yoon , Emily Y. Ko , Jlenia Guarnerio , et al. 2021. “Commensal Bacteria and Fungi Differentially Regulate Tumor Responses to Radiation Therapy.” Cancer Cell 39: 1202–13.e6. 10.1016/j.ccell.2021.07.002 34329585 PMC8830498

[687] Mougeot, Jean‐Luc C. , Craig B. Stevens , Kathryn G. Almon , Bruce J. Paster , Rajesh V. Lalla , Michael T. Brennan , Farah Bahrani Mougeot . 2019. “Caries‐Associated Oral Microbiome in Head and Neck Cancer Radiation Patients: A Longitudinal Study.” Journal of Oral Microbiology 11: 1586421. 10.1080/20002297.2019.1586421 30891159 PMC6419625

[688] Arrifin, Azirrawani , Ellie Heidari , Mary Burke , Michael R. Fenlon , Avijit Banerjee . 2018. “The Effect of Radiotherapy for Treatment of Head and Neck Cancer on Oral Flora and Saliva.” Oral Health & Preventive Dentistry 16: 425–429. 10.3290/j.ohpd.a41364 30460355

[689] Danckaert, Willeke , Mathieu Spaas , Nora Sundahl , Aurélie De Bruycker , Valérie Fonteyne , Ellen De Paepe , Carlos De Wagter , Lynn Vanhaecke , Piet Ost . 2023. “Microbiome and Metabolome Dynamics During Radiotherapy for Prostate Cancer.” Radiotherapy and Oncology 189: 109950. 10.1016/j.radonc.2023.109950 37827280

[690] Reis Ferreira, Miguel , H. Jervoise N. Andreyev , Kabir Mohammed , Lesley Truelove , Sharon M. Gowan , Jia Li , Sarah L. Gulliford , Julian R. Marchesi , David P. Dearnaley . 2019. “Microbiota‐ and Radiotherapy‐Induced Gastrointestinal Side‐Effects (MARS) Study: A Large Pilot Study of the Microbiome in Acute and Late‐Radiation Enteropathy.” Clinical Cancer Research: An Official Journal of the American Association for Cancer Research 25: 6487–6500. 10.1158/1078-0432.CCR-19-0960 31345839

[691] Olivas, Andrea D. , Benjamin D. Shogan , Vesta Valuckaite , Alexander Zaborin , Natalya Belogortseva , Mark Musch , Folker Meyer , et al. 2012. “Intestinal Tissues Induce an SNP Mutation in *Pseudomonas aeruginosa* That Enhances Its Virulence: Possible Role in Anastomotic Leak.” PloS One 7: e44326. 10.1371/journal.pone.0044326 22952955 PMC3432121

[692] Bahig, Houda , Clifton D. Fuller , Aparna Mitra , Kyoko Yoshida‐Court , Travis Solley , Sweet Ping Ng , Ibrahim Abu‐Gheida , et al. 2021. “Longitudinal Characterization of the Tumoral Microbiome During Radiotherapy in HPV‐Associated Oropharynx Cancer.” Clinical and Translational Radiation Oncology 26: 98–103. 10.1016/j.ctro.2020.11.007 33367119 PMC7749292

[693] Reis Ferreira, Miguel , H. Jervoise N. Andreyev , Kabir Mohammed , Lesley Truelove , Sharon M. Gowan , Jia Li , Sarah L. Gulliford , Julian R. Marchesi , David P. Dearnaley . 2019. “Microbiota‐ and Radiotherapy‐Induced Gastrointestinal Side‐Effects (MARS) Study: A Large Pilot Study of the Microbiome in Acute and Late‐Radiation Enteropathy.” Clinical Cancer Research: An Official Journal of the American Association for Cancer Research 25: 6487–6500. 10.1158/1078-0432.CCR-19-0960 31345839

[694] Ciorba, Matthew A. , Terrence E. Riehl , M. Suprada Rao , Clara Moon , Xueping Ee , Gerardo M. Nava , Monica R. Walker , et al. 2012. “Lactobacillus Probiotic Protects Intestinal Epithelium From Radiation Injury in a TLR‐2/Cyclo‐Oxygenase‐2‐Dependent Manner.” Gut 61: 829–838. 10.1136/gutjnl-2011-300367 22027478 PMC3345937

[695] Zhao, Zhenguo , Wei Cheng , Wei Qu , Guoyi Shao , Shuanghai Liu . 2020. “Antibiotic Alleviates Radiation‐Induced Intestinal Injury by Remodeling Microbiota, Reducing Inflammation, and Inhibiting Fibrosis.” ACS Omega 5: 2967–2977. 10.1021/acsomega.9b03906 32095719 PMC7033964

[696] Abdollahi, Elham , Thomas P. Johnston , Zahra Ghaneifar , Parviz Vahedi , Pouya Goleij , Sara Azhdari , Abbas Shapouri Moghaddam . 2023. “Immunomodulatory Therapeutic Effects of Curcumin on M1/M2 Macrophage Polarization in Inflammatory Diseases.” Current Molecular Pharmacology 16: 2–14. 10.2174/1874467215666220324114624 35331128

[697] Jia, Dingjiacheng , Qiwen Wang , Yadong Qi , Yao Jiang , Jiamin He , YIFENG Lin , Yong Sun , et al. 2024. “Microbial Metabolite Enhances Immunotherapy Efficacy by Modulating T Cell Stemness in Pan‐Cancer.” Cell 187: 1651–65.e21. 10.1016/j.cell.2024.02.022 38490195

[698] McCulloch, John A. , Diwakar Davar , Richard R. Rodrigues , Jonathan H. Badger , Jennifer R. Fang , Alicia M. Cole , Ascharya K. Balaji , et al. 2022. “Intestinal Microbiota Signatures of Clinical Response and Immune‐Related Adverse Events in Melanoma Patients Treated With Anti‐PD‐1.” Nature Medicine 28: 545–556. 10.1038/s41591-022-01698-2 PMC10246505 35228752

[699] Gunjur, Ashray , Yan Shao , Timothy Rozday , Oliver Klein , Andre Mu , Bastiaan W. Haak , Ben Markman , et al. 2024. “A Gut Microbial Signature For Combination Immune Checkpoint Blockade Across Cancer Types.” Nature Medicine 30: 797–809. 10.1038/s41591-024-02823-z PMC10957475 38429524

[700] Björk, Johannes R. , Laura A. Bolte , Andrew Maltez Thomas , Karla A. Lee , Niccolo Rossi , Thijs T. Wind , Lotte M. Smit , et al. 2024. “Longitudinal Gut Microbiome Changes in Immune Checkpoint Blockade‐Treated Advanced Melanoma.” Nature Medicine 30: 785–796. 10.1038/s41591-024-02803-3 PMC10957474 38365950

[701] Lee, Karla A. , Andrew Maltez Thomas , Laura A. Bolte , Johannes R. Björk , Laura Kist De Ruijter , Federica Armanini , Francesco Asnicar , et al. 2022. “Cross‐Cohort Gut Microbiome Associations With immune Checkpoint Inhibitor Response in Advanced Melanoma.” Nature Medicine 28: 535–544. 10.1038/s41591-022-01695-5 PMC8938272 35228751

[702] Chaput, N. , P. Lepage , C. Coutzac , E. Soularue , K. Le Roux , C. Monot , L. Boselli , et al. 2017. “Baseline Gut Microbiota Predicts Clinical Response and Colitis in Metastatic Melanoma Patients Treated With Ipilimumab.” Annals of Oncology 28: 1368–1379. 10.1093/annonc/mdx108 28368458

[703] Dubin, Krista , Margaret K. Callahan , Boyu Ren , Raya Khanin , Agnes Viale , Lilan Ling , Daniel No , et al. 2016. “Intestinal Microbiome Analyses Identify Melanoma patients at Risk for Checkpoint‐Blockade‐Induced Colitis.” Nature Communications 7: 10391. 10.1038/ncomms10391 PMC4740747 26837003

[704] Frankel, Arthur E. , Laura A. Coughlin , Jiwoong Kim , Thomas W. Froehlich , Yang Xie , Eugene P. Frenkel , Andrew Y. Koh . 2017. “Metagenomic Shotgun Sequencing and Unbiased Metabolomic Profiling Identify Specific Human Gut Microbiota and Metabolites Associated With Immune Checkpoint Therapy Efficacy in Melanoma Patients.” Neoplasia 19: 848–855. 10.1016/j.neo.2017.08.004 28923537 PMC5602478

[705] Huang, Xiaowen , Muni Hu , Tiantian Sun , Jiantao Li , Yilu Zhou , Yuqing Yan , Baoqin Xuan , et al. 2023. “Multi‐Kingdom Gut Microbiota Analyses Define Bacterial–Fungal Interplay and Microbial Markers of Pan‐Cancer Immunotherapy Across Cohorts.” Cell host & microbe 31: 1930–43.e4. 10.1016/j.chom.2023.10.005 37944495

[706] Deng, Weiwei , Zhen Su , Panpan Liang , Yubo Ma , Yufang Liu , Kai Zhang , Yi Zhang , et al. 2021. “Single‐Cell Immune Checkpoint Landscape of PBMCs Stimulated With *Candida albicans* .” Emerging Microbes & Infections 10: 1272–1283. 10.1080/22221751.2021.1942228 34120578 PMC8238073

[707] Prasad, Rishika , Abdur Rehman , Lubna Rehman , Faezeh Darbaniyan , Viktoria Blumenberg , Maria‐Luisa Schubert , Uria Mor , et al. 2025. “Antibiotic‐Induced Loss of Gut Microbiome Metabolic Output Correlates With Clinical Responses to CAR T‐Cell Therapy.” Blood 145: 823–839. 10.1182/blood.2024025366 39441941

[708] DeFilipp, Zachariah , Marcela V. Maus . 2023. “Linking the Microbiome to CAR‐T Cell Responses.” Nature Medicine 29: 785–786. 10.1038/s41591-023-02272-0 36973411

[709] CD19 CAR T‐Cell Clinical Outcome Is Associated With the Gut Microbiome. 2022. Cancer Discovery 12: 1182. 10.1158/2159-8290.CD-RW2022-052 35491638

[710] Smith, Melody , Anqi Dai , Guido Ghilardi , Kimberly V. Amelsberg , Sean M. Devlin , Raymone Pajarillo , John B. Slingerland , et al. 2022. “Gut Microbiome Correlates of Response and Toxicity Following Anti‐CD19 CAR T Cell Therapy.” Nature Medicine 28: 713–723. 10.1038/s41591-022-01702-9 PMC9434490 35288695

[711] Abid, Muhammad Bilal , Nirav N. Shah , Theresa C. Maatman , Parameswaran N. Hari . 2019. “Gut Microbiome and CAR‐T Therapy.” Experimental Hematology & Oncology 8: 31. 10.1186/s40164-019-0155-8 31827982 PMC6862813

[712] Shen, Junyi , Rong Hu , Anqi Lin , Aimin Jiang , Bufu Tang , Zaoqu Liu , Quan Cheng , et al. 2024. “Characterization of?Second Primary Malignancies Post CAR T‐Cell Therapy: Real‐World Insights From the Two Global Pharmacovigilance Databases of FAERS and VigiBase.” EClinicalMedicine 73: 102684. 10.1016/j.eclinm.2024.102684 39007060 PMC11245995

[713] Hu, Yongxian , Jingjing Li , Fang Ni , Zhongli Yang , Xiaohua Gui , Zhiwei Bao , Houli Zhao , et al. 2022. “CAR‐T Cell Therapy‐Related Cytokine Release Syndrome and Therapeutic Response Is Modulated by the Gut Microbiome in Hematologic Malignancies.” Nature Communications 13: 5313. 10.1038/s41467-022-32960-3 PMC9461447 36085303

[714] Stergiopoulos, Georgios M. , Ianko Iankov , Evanthia Galanis . 2024. “Personalizing Oncolytic Immunovirotherapy Approaches.” Molecular Diagnosis & Therapy 28: 153–168. 10.1007/s40291-023-00689-4 38150172

[715] Sivan, Ayelet , Leticia Corrales , Nathaniel Hubert , Jason B. Williams , Keston Aquino‐Michaels , Zachary M. Earley , Franco W. Benyamin , et al. 2015. “Commensal Bifidobacterium Promotes Antitumor Immunity and Facilitates Anti‐PD‐L1 Efficacy.” Science 350: 1084–1089. 10.1126/science.aac4255 26541606 PMC4873287

[716] Vétizou, Marie , Jonathan M. Pitt , Romain Daillère , Patricia Lepage , Nadine Waldschmitt , Caroline Flament , Sylvie Rusakiewicz , et al. 2015. “Anticancer Immunotherapy by CTLA‐4 Blockade Relies on the Gut Microbiota.” Science 350: 1079–1084. 10.1126/science.aad1329 26541610 PMC4721659

[717] Mager, Lukas F. , Regula Burkhard , Nicola Pett , Noah C. A. Cooke , Kirsty Brown , Hena Ramay , Seungil Paik , et al. 2020. “Microbiome‐Derived Inosine Modulates Response to Checkpoint Inhibitor Immunotherapy.” Science 369: 1481–1489. 10.1126/science.abc3421 32792462

[718] Ravilla, Rahul , Hannah N. Coleman , Cheryl‐Emiliane Chow , Luisa Chan , Barbara J. Fuhrman , William W. Greenfield , Michael Scott Robeson , et al. 2019. “Cervical Microbiome and Response to a Human Papillomavirus Therapeutic Vaccine for Treating High‐Grade Cervical Squamous Intraepithelial Lesion.” Integrative Cancer Therapies 18: 1534735419893063. 10.1177/1534735419893063 31833799 PMC6913049

[719] Guardamagna, Mora , Miguel‐Angel Berciano‐Guerrero , Beatriz Villaescusa‐González , Elisabeth Perez‐Ruiz , Javier Oliver , Rocío Lavado‐Valenzuela , Antonio Rueda‐Dominguez , Isabel Barragán , María Isabel Queipo‐Ortuño . 2022. “Gut Microbiota and Therapy in Metastatic Melanoma: Focus on MAPK Pathway Inhibition.” International Journal of Molecular Sciences 23: 11990. 10.3390/ijms231911990 36233289 PMC9569448

[720] Mu, Wenxin , Yiqun Jia , Xiaobing Chen , Haoyu Li , Zhi Wang , Bin Cheng 2020. “Intracellular *Porphyromonas gingivalis* Promotes the Proliferation of Colorectal Cancer Cells via the MAPK/ERK Signaling Pathway.” Frontiers in Cellular and Infection Microbiology 10: 584798. 10.3389/fcimb.2020.584798 33425779 PMC7785964

[721] Shan, Khine S. , Tauseef U. Rehman , Stan Ivanov , Gelenis Domingo , Luis E. Raez . 2024. “Molecular Targeting of the BRAF Proto‐Oncogene/Mitogen‐Activated Protein Kinase (MAPK) Pathway Across Cancers.” International Journal of Molecular Sciences 25: 624. 10.3390/ijms25010624 38203795 PMC10779188

[722] Kumar, Mukesh , Ramanpreet Kaur , Shruthi Kanthaje , Radha K. Dhiman , Anuradha Chakraborti . 2023. “Bacterial Metabolite Butyrate in Modulating Sorafenib‐Targeted MicroRNAs to Curtail Its Resistance in Hepatocellular Carcinoma.” Journal of Cancer Research and Clinical Oncology 149: 5823–5839. 10.1007/s00432-022-04544-7 36583742 PMC11798150

[723] Yu, Jingjing , Xiaoping Chen , Xiangliang Yang , Bixiang Zhang . 2024. “Understanding Gut Dysbiosis for Hepatocellular Carcinoma Diagnosis and Treatment.” Trends in Endocrinology & Metabolism 35: 1006–1020. 10.1016/j.tem.2024.06.003 38969601

[724] Ahmed, Lamiaa A. , Khaled F. Al‐Massri . 2023. “Gut Microbiota Modulation for Therapeutic Management of Various Diseases: A New Perspective Using Stem Cell Therapy.” Current Molecular Pharmacology 16: 43–59. 10.2174/1874467215666220222105004 35196976

[725] Hahn, Andrew W. , Camryn Froerer , Sidney VanAlstine , Nityam Rathi , Erin B. Bailey , David D. Stenehjem , Neeraj Agarwal . 2018. “Targeting Bacteroides in Stool Microbiome and Response to Treatment With First‐Line VEGF Tyrosine Kinase Inhibitors in Metastatic Renal‐Cell Carcinoma.” Clinical Genitourinary Cancer 16: 365–368. 10.1016/j.clgc.2018.05.001 29858123

[726] Ianiro, Gianluca , Ernesto Rossi , Andrew M. Thomas , Giovanni Schinzari , Luca Masucci , Gianluca Quaranta , Carlo Romano Settanni , et al. 2020. “Faecal Microbiota Transplantation for the Treatment of Diarrhoea Induced by Tyrosine‐Kinase Inhibitors in Patients With Metastatic Renal Cell Carcinoma.” Nature Communications 11: 4333. 10.1038/s41467-020-18127-y PMC7455693 32859933

[727] Van Praagh, Jasper B. , Marcus C. De Goffau , Ilsalien S. Bakker , Harry Van Goor , Hermie J. M. Harmsen , Peter Olinga , Klaas Havenga . 2019. “Mucus Microbiome of Anastomotic Tissue During Surgery Has Predictive Value for Colorectal Anastomotic Leakage.” Annals of Surgery 269: 911–916. 10.1097/SLA.0000000000002651 29303807

[728] Rollins, Katie E. , Hannah Javanmard‐Emamghissi , Austin G. Acheson , Dileep N. Lobo . 2019. “The Role of Oral Antibiotic Preparation in Elective Colorectal Surgery: A Meta‐analysis.” Annals of Surgery 270: 43–58. 10.1097/SLA.0000000000003145 30570543 PMC6570620

[729] Darbandi, Atieh , Maryam Mirshekar , Aref Shariati , Majid Taati Moghadam , Vahid Lohrasbi , Parisa Asadolahi , Malihe Talebi . 2020. “The Effects of Probiotics on Reducing the Colorectal Cancer Surgery Complications: A Periodic Review During 2007–2017.” Clinical Nutrition 39: 2358–2367. 10.1016/j.clnu.2019.11.008 31831184

[730] Kiran, Ravi Pokala , Alice C. A. Murray , Cody Chiuzan , David Estrada , Kenneth Forde . 2015. “Combined Preoperative Mechanical Bowel Preparation With Oral Antibiotics Significantly Reduces Surgical Site Infection, Anastomotic Leak, and Ileus After Colorectal Surgery.” Annals of Surgery 262: 416–425; discussion 423–5. 10.1097/SLA.0000000000001416 26258310

[731] Cong, Jing , Hua Zhu , Dong Liu , Tianjun Li , Chuantao Zhang , Jingjuan Zhu , Hongying Lv , et al. 2018. “A Pilot Study: Changes of Gut Microbiota in Post‐Surgery Colorectal Cancer Patients.” Frontiers in Microbiology 9: 2777. 10.3389/fmicb.2018.02777 30515141 PMC6255893

[732] Lapthorne, Susan . 2015. “Changes in the Colon Microbiota and Intestinal Cytokine Gene Expression Following Minimal Intestinal Surgery.” World Journal of Gastroenterology 21: 4150–4158. 10.3748/wjg.v21.i14.4150 25892864 PMC4394075

[733] Kong, Cheng , Renyuan Gao , Xuebing Yan , Linsheng Huang , Jide He , Hao Li , Jie You , Huanlong Qin . 2019. “Alterations in Intestinal Microbiota of Colorectal Cancer Patients Receiving Radical Surgery Combined With Adjuvant CapeOx Therapy.” Science China. Life Sciences 62: 1178–1193. 10.1007/s11427-018-9456-x 30796721

[734] Peng, Yu‐Chong , Xin‐Hua Zhao , Chuan‐Fa Zeng , Jing‐Xuan Xu , Lu‐Nan Qi , Le‐Qun Li . 2022. “Integrated Omics Analysis: The Relationship Between Significantly Increased Klebsiella Post‐Hepatectomy and Decreased Hub‐Metabolite 3‐Methyl‐2‐Oxobutanoic Acid Is Associated With Induced Liver Failure.” Journal of Gastrointestinal Oncology 13: 326–343. 10.21037/jgo-21-906 35284109 PMC8899754

[735] Yang, Jie , Yuhua He , Xi Liao , Jiankun Hu , Ka Li . 2023. “Does Postoperative Pulmonary Infection Correlate With Intestinal Flora Following Gastric Cancer Surgery?—A Nested Case‐Control Study.” Frontiers in Microbiology 14: 1267750. 10.3389/fmicb.2023.1267750 38029086 PMC10658784

[736] Xu, Fusheng , Zhiming Yu , Yaru Liu , Ting Du , Leilei Yu , Fengwei Tian , Wei Chen , Qixiao Zhai . 2023. “A High‐Fat, High‐Cholesterol Diet Promotes Intestinal Inflammation by Exacerbating Gut Microbiome Dysbiosis and Bile Acid Disorders in Cholecystectomy.” Nutrients 15: 3829. 10.3390/nu15173829 37686860 PMC10489946

[737] Poutahidis, Theofilos , Sean M. Kearney , Tatiana Levkovich , Peimin Qi , Bernard J. Varian , Jessica R. Lakritz , Yassin M. Ibrahim , et al. 2013. “Microbial Symbionts Accelerate Wound Healing via the Neuropeptide Hormone Oxytocin.” PloS One 8: e78898. 10.1371/journal.pone.0078898 24205344 PMC3813596

[738] Obermüller, Beate , Georg Singer , Bernhard Kienesberger , Barbara Mittl , Vanessa Stadlbauer , Angela Horvath , Wolfram Miekisch , et al. 2023. “Probiotic OMNi‐BiOTiC® 10 AAD Reduces Cyclophosphamide‐Induced Inflammation and Adipose Tissue Wasting in Mice.” Nutrients 15: 3655. 10.3390/nu15163655 37630845 PMC10458463

[739] Yazdi, Mohammad Hossein , Mehdi Mahdavi , Neda Setayesh , Mohammad Esfandyar , Ahmad Reza Shahverdi . 2013. “Selenium Nanoparticle‐Enriched *Lactobacillus brevis* Causes More Efficient Immune Responses In Vivo and Reduces the Liver Metastasis in Metastatic Form of Mouse Breast Cancer.” DARU Journal of Pharmaceutical Sciences 21: 33. 10.1186/2008-2231-21-33 23631392 PMC3658950

[740] Xu, Fuqiang , Qiaoqiao Li , Shuyang Wang , Miaoyin Dong , Guoqing Xiao , Jin Bai , Junkai Wang , Xisi Sun . 2023. “The Efficacy of Prevention for Colon Cancer Based on the Microbiota Therapy and the Antitumor Mechanisms With Intervention of Dietary Lactobacillus.” Microbiology Spectrum 11: e0018923. 10.1128/spectrum.00189-23 37655887 PMC10581183

[741] Zeighamy Alamdary, Shabnam , Shahnaz Halimi , Akram Rezaei , Roghayeh Afifirad . 2023. “Association Between Probiotics and Modulation of Gut Microbial Community Composition in Colorectal Cancer Animal Models: A Systematic Review (2010–2021).” The Canadian Journal of Infectious Diseases & Medical Microbiology = Journal Canadien Des Maladies Infectieuses Et De La Microbiologie Medicale 2023: 3571184. 10.1155/2023/3571184 37719797 PMC10505085

[742] Wei, Hao , Zhiying Yue , Jialong Han , Ping Chen , Ke Xie , Yu Sun , Jiang Zhu . 2024. “Oral Compound Probiotic Supplements Can Improve the Quality of Life for Patients With Lung Cancer During Chemotherapy: A Randomized Placebo‐Controlled Study.” Thoracic Cancer 15: 182–191. 10.1111/1759-7714.15177 38018652 PMC10788472

[743] Shi, Qi , Jia Wang , Mengnan Zhou , Rui Zheng , Xiaoli Zhang , Beixing Liu . 2023. “Gut Lactobacillus Contribute to the Progression of Breast Cancer by Affecting the Anti‐Tumor Activities of Immune Cells in the TME of Tumor‐Bearing Mice.” International Immunopharmacology 124: 111039. 10.1016/j.intimp.2023.111039 37862739

[744] Jang, Albert , Jake N. Lichterman , Jeffrey Y. Zhong , Jonathan E. Shoag , Jorge A. Garcia , Tian Zhang , Pedro C. Barata . 2023. “Immune Approaches Beyond Traditional Immune Checkpoint Inhibitors for Advanced Renal Cell Carcinoma.” Human Vaccines & Immunotherapeutics 19: 2276629. 10.1080/21645515.2023.2276629 37947202 PMC10653627

[745] Lin, Anqi , Aimin Jiang , Lihaoyun Huang , Yu Li , Chunyanx Zhang , Lingxuan Zhu , Weiming Mou , et al. 2025. “From Chaos to Order: Optimizing Fecal Microbiota Transplantation for Enhanced Immune Checkpoint Inhibitors Efficacy.” Gut Microbes 17: 2452277. 10.1080/19490976.2025.2452277 39826104 PMC12716052

[746] Wang, Xinjun , Di Zhao , Dexi Bi , Long Li , Hongliang Tian , Fang Yin , Tao Zuo , et al. 2025. “Fecal Microbiota Transplantation: Transitioning From Chaos and Controversial Realm to Scientific Precision Era.” Science Bulletin 70: 970‐985. 10.1016/j.scib.2025.01.029 39855927

[747] Kim, Do‐Yeon , So‐Yeon Lee , Jae‐Yun Lee , Tae Woong Whon , June‐Young Lee , Che Ok Jeon , Jin‐Woo Bae . 2024. “Gut Microbiome Therapy: Fecal Microbiota Transplantation vs Live Biotherapeutic Products.” Gut Microbes 16: 2412376. 10.1080/19490976.2024.2412376 39377231 PMC11469438

[748] Yang, Yunwei , Yaping An , Yue Dong , Qiao Chu , Jingge Wei , Bangmao Wang , Hailong Cao . 2024. “Fecal Microbiota Transplantation: No Longer Cinderella in Tumour Immunotherapy.” EBioMedicine 100: 104967. 10.1016/j.ebiom.2024.104967 38241975 PMC10831174

[749] Yadegar, Abbas , Haggai Bar‐Yoseph , Tanya Marie Monaghan , Sepideh Pakpour , Andrea Severino , Ed J. Kuijper , Wiep Klaas Smits , et al. 2024. “Fecal Microbiota Transplantation: Current Challenges and Future Landscapes.” Clinical Microbiology Reviews 37: e0006022. 10.1128/cmr.00060-22 38717124 PMC11325845

[750] Cao, Yanyan , Lijie Zhang , Fu Xiong , Xiaopeng Guo , Xuefeng Kan , Songlin Song , Bo Liang , et al. 2024. “Effect of Probiotics and Fecal Microbiota Transplantation in Dirty Rats With Established Primary Liver Cancer.” Future Microbiology 19: 117–129. 10.2217/fmb-2022-0234 37934064

[751] Cheng, Xiaoshuo , Xiaozheng Li , Xudong Yang , Shaojun Fang , Zhenyu Wang , Tingting Liu , Mengyao Zheng , et al. 2023. “Successful Treatment of pMMR MSS IVB Colorectal Cancer Using Anti‐VEGF and Anti‐PD‐1 Therapy in Combination of Gut Microbiota Transplantation: A Case Report.” Cureus 15: e42347. 10.7759/cureus.42347 37621810 PMC10445052

[752] Cui, Ming , Huiwen Xiao , Yuan Li , Lixin Zhou , Shuyi Zhao , Dan Luo , Qisheng Zheng , et al. 2017. “Faecal Microbiota Transplantation Protects Against Radiation‐Induced Toxicity.” EMBO Molecular Medicine 9: 448–461. 10.15252/emmm.201606932 28242755 PMC5376756

[753] Lythgoe, Mark P. , Rohma Ghani , Benjamin H. Mullish , Julian R. Marchesi , Jonathan Krell . 2022. “The Potential of Fecal Microbiota Transplantation in Oncology.” Trends in Microbiology 30: 10–12. 10.1016/j.tim.2021.10.003 34711461

[754] Ninkov, Marina , Crystal L. Schmerk , Manoosh Moradizadeh , Seema N. Parvathy , Rene Figueredo , Jeremy P. Burton , Michael S. Silverman , et al. 2023. “Improved MAIT Cell Functions Following Fecal Microbiota Transplantation for Metastatic Renal Cell Carcinoma.” Cancer Immunology, Immunotherapy 72: 1247–1260. 10.1007/s00262-022-03329-8 36396738 PMC9672546

[755] Hefazi, Mehrdad , Mrinal M. Patnaik , William J. Hogan , Mark R. Litzow , Darrell S. Pardi , Sahil Khanna . 2017. “Safety and Efficacy of Fecal Microbiota Transplant for Recurrent Clostridium Difficile Infection in Patients With Cancer Treated With Cytotoxic Chemotherapy: A Single‐Institution Retrospective Case Series.” Mayo Clinic Proceedings 92: 1617–1624. 10.1016/j.mayocp.2017.08.016 29101931

[756] Halsey, Taylor M. , Anusha S. Thomas , Tomo Hayase , Weijie Ma , Hamzah Abu‐Sbeih , Baohua Sun , Edwin Roger Parra , et al. 2023. “Microbiome Alteration via Fecal Microbiota Transplantation Is Effective for Refractory Immune Checkpoint Inhibitor‐Induced Colitis.” Science Translational Medicine 15: eabq4006. 10.1126/scitranslmed.abq4006 37315113 PMC10759507

[757] Baruch, Erez N. , Ilan Youngster , Guy Ben‐Betzalel , Rona Ortenberg , Adi Lahat , Lior Katz , Katerina Adler , et al. 2021. “Fecal Microbiota Transplant Promotes Response in Immunotherapy‐Refractory Melanoma Patients.” Science 371: 602–609. 10.1126/science.abb5920 33303685

[758] Davar, Diwakar , Amiran K. Dzutsev , John A. McCulloch , Richard R. Rodrigues , Joe‐Marc Chauvin , Robert M. Morrison , Richelle N. Deblasio , et al. 2021. “Fecal Microbiota Transplant Overcomes Resistance to Anti‐PD‐1 Therapy in Melanoma Patients.” Science 371: 595–602. 10.1126/science.abf3363 33542131 PMC8097968

[759] Routy, Bertrand , John G. Lenehan , Wilson H. Miller , Rahima Jamal , Meriem Messaoudene , Brendan A. Daisley , Cecilia Hes , et al. 2023. “Fecal Microbiota Transplantation Plus Anti‐PD‐1 Immunotherapy in Advanced Melanoma: A phase I Trial.” Nature Medicine 29: 2121–2132. 10.1038/s41591-023-02453-x 37414899

[760] Wang, Yinghong , Diana H. Wiesnoski , Beth A. Helmink , Vancheswaran Gopalakrishnan , Kati Choi , Hebert L. DuPont , Zhi‐Dong Jiang , et al. 2018. “Fecal Microbiota Transplantation for Refractory Immune Checkpoint Inhibitor‐Associated Colitis.” Nature Medicine 24: 1804–1808. 10.1038/s41591-018-0238-9 PMC6322556 30420754

[761] Simin, Johanna , Rulla M. Tamimi , Lars Engstrand , Steven Callens , Nele Brusselaers . 2020. “Antibiotic Use and the Risk of Breast Cancer: A Systematic Review and Dose–Response Meta‐Analysis.” Pharmacological Research 160: 105072. 10.1016/j.phrs.2020.105072 32679181

[762] Lurienne, Lise , Julie Cervesi , Lola Duhalde , Jean De Gunzburg , Antoine Andremont , Gérard Zalcman , Renaud Buffet , Pierre‐Alain Bandinelli . 2020. “NSCLC Immunotherapy Efficacy and Antibiotic Use: A Systematic Review and Meta‐Analysis.” Journal of Thoracic Oncology 15: 1147–1159. 10.1016/j.jtho.2020.03.002 32173463

[763] Imlay, Hannah , Nicholas C. Laundy , Graeme N. Forrest , Monica A. Slavin . 2023. “Shorter Antibiotic Courses in the Immunocompromised: The Impossible Dream?” Clinical Microbiology and Infection 29: 143–149. 10.1016/j.cmi.2022.08.007 35988852

[764] Singh, Vishal , Beng San Yeoh , Ahmed A. Abokor , Rachel M. Golonka , Yuan Tian , Andrew D. Patterson , Bina Joe , Mathias Heikenwalder , Matam Vijay‐Kumar . 2020. “Vancomycin Prevents Fermentable Fiber‐Induced Liver Cancer in Mice With Dysbiotic Gut Microbiota.” Gut Microbes 11: 1077–1091. 10.1080/19490976.2020.1743492 32223398 PMC7524287

[765] Pinato, David J. , Xiaoxue Li , Pallavi Mishra‐Kalyani , Antonio D'Alessio , Claudia A. M. Fulgenzi , Bernhard Scheiner , Matthias Pinter , et al. 2023. “Association Between Antibiotics and Adverse Oncological Outcomes in Patients Receiving Targeted or Immune‐Based Therapy for Hepatocellular Carcinoma.” JHEP Reports 5: 100747. 10.1016/j.jhepr.2023.100747 37197442 PMC10183666

[766] Iida, Noriho , Eishiro Mizukoshi , Tatsuya Yamashita , Takeshi Terashima , Kuniaki Arai , Jun Seishima , Shuichi Kaneko . 2019. “Overuse of Antianaerobic Drug Is Associated With Poor Postchemotherapy Prognosis of Patients With Hepatocellular Carcinoma.” International Journal of Cancer 145: 2701–2711. 10.1002/ijc.32339 30980680 PMC6766885

[767] López‐Bucio, José . 2025. “Dietary Auxin May Help Patients to Fight Cancer.” Trends in Plant Science 30: 134–136. 10.1016/j.tplants.2024.10.016 39510947

[768] Arifuzzaman, Mohammad , Nicholas Collins , Chun‐Jun Guo , David Artis . 2024. “Nutritional Regulation of Microbiota‐Derived Metabolites: Implications for Immunity and Inflammation.” Immunity 57: 14–27. 10.1016/j.immuni.2023.12.009 38198849 PMC10795735

[769] Ilerhunmwuwa, Nosakhare Paul , Abul Hasan Shadali Abdul Khader , Calvin Smith , Edward R Scheffer Cliff , Christopher M. Booth , Evevanne Hottel , Muhammad Aziz , et al. 2024. “Dietary Interventions in Cancer: A Systematic Review of All Randomized Controlled Trials.” Journal of the National Cancer Institute 116: 1026–1034. 10.1093/jnci/djae051 38429997 PMC11223872

[770] Xiao, Yu‐Ling , Yue Gong , Ying‐Jia Qi , Zhi‐Ming Shao , Yi‐Zhou Jiang . 2024. “Effects of Dietary Intervention on Human Diseases: Molecular Mechanisms and Therapeutic Potential.” Signal Transduction and Targeted Therapy 9: 59. 10.1038/s41392-024-01771-x 38462638 PMC10925609

[771] Bolte, Laura A. , Karla A. Lee , Johannes R. Björk , Emily R. Leeming , Marjo J. E. Campmans‐Kuijpers , Jacco J. De Haan , Arnau Vich Vila , et al. 2023. “Association of a Mediterranean Diet With Outcomes for Patients Treated With Immune Checkpoint Blockade for Advanced Melanoma.” JAMA Oncology 9: 705–709. 10.1001/jamaoncol.2022.7753 36795408 PMC9936383

[772] Li, Ziyuan , Lei Qian , Jianghui Chu , Yuan Liu , Gusonghan Maitiniyazi , Yue Chen , Xinxin Cheng , et al. 2023. “Diet Is Associated With Frailty in Lung Cancer: A Possible Role of Gut Microbiota.” Nutrients 15: 4298. 10.3390/nu15194298 37836582 PMC10574134

[773] Li, Lili , Shuling Yan , Shuangjiang Liu , Ping Wang , Wenjun Li , Yuetao Yi , Song Qin . 2023. “In‐Depth Insight Into Correlations Between Gut Microbiota and Dietary Fiber Elucidates a Dietary Causal Relationship With Host Health.” Food Research International (Ottawa, Ont.) 172: 113133. 10.1016/j.foodres.2023.113133 37689844

[774] Wang, Chuhui , Tongtong Lan , Zhao Chen , Xiaowen Wang , Yisa Han , Ning Yang , Zhen Xu , et al. 2023. “The Preventive Effects of Inulin, Cellulose, and Their Mixture on Colorectal Cancer Liver Metastasis in Mice by Regulating Gut Microbiota.” Journal of Food Science 88: 4705–4717. 10.1111/1750-3841.16772 37815692

[775] Fang, Gaofeng , Shengquan Wang , Qianyao Chen , Han Luo , Xuemei Lian , Dan Shi . 2023. “Time‐Restricted Feeding Affects the Fecal Microbiome Metabolome and Its Diurnal Oscillations in Lung Cancer Mice.” Neoplasia 45: 100943. 10.1016/j.neo.2023.100943 37852131 PMC10590998

[776] Ronis, Martin J. , Kelly E. Mercer , Kartik Shankar , Casey Pulliam , Kim Pedersen , Magnus Ingelman‐Sundberg , Simonetta Friso , et al. 2020. “Potential Role of Gut Microbiota, the Proto‐Oncogene PIKE (Agap2) and Cytochrome P450 CYP2W1 in Promotion of Liver Cancer by Alcoholic and Nonalcoholic Fatty Liver Disease and Protection by Dietary Soy Protein.” Chemico‐Biological Interactions 325: 109131. 10.1016/j.cbi.2020.109131 32417163 PMC7370542

[777] Yu, Yinglan , Xinran Shen , Xin Xiao , Lian Li , Yuan Huang . 2023. “Butyrate Modification Promotes Intestinal Absorption and Hepatic Cancer Cells Targeting of Ferroptosis Inducer Loaded Nanoparticle for Enhanced Hepatocellular Carcinoma Therapy.” Small (Weinheim an Der Bergstrasse, Germany) 19: e2301149. 10.1002/smll.202301149 37165608

[778] Wang, Hongbin , Mia Borlongan , Howard L. Kaufman , Uyen Le , Hans J. Nauwynck , Samuel D. Rabkin , Dipongkor Saha . 2024. “Cytokine‐Armed Oncolytic Herpes Simplex Viruses: A Game‐Changer in Cancer Immunotherapy?” Journal for Immunotherapy of Cancer 12: e008025. 10.1136/jitc-2023-008025 38821716 PMC11149157

[779] Duan, Shijie , Shuhang Wang , Lei Qiao , Xinbo Yu , Nan Wang , Liting Chen , Xinyuan Zhang , et al. 2023. “Oncolytic Virus‐Driven Biotherapies From Bench to Bedside.” Small (Weinheim an Der Bergstrasse, Germany) 19: e2206948. 10.1002/smll.202206948 36879416

[780] Burchett, Rebecca , Scott Walsh , Yonghong Wan , Jonathan L. Bramson . 2020. “A Rational Relationship: Oncolytic Virus Vaccines as Functional Partners for Adoptive T Cell Therapy.” Cytokine & Growth Factor Reviews 56: 149–159. 10.1016/j.cytogfr.2020.07.003 32665126

[781] Nguyen, Hong‐My , Praveen K. Bommareddy , Ann W. Silk , Dipongkor Saha . 2022. “Optimal Timing of PD‐1 Blockade in Combination With Oncolytic Virus Therapy.” Seminars in Cancer Biology 86: 971–980. 10.1016/j.semcancer.2021.05.019 34033895

[782] DePeaux, Kristin , Greg M. Delgoffe . 2024. “Integrating Innate and Adaptive Immunity in Oncolytic Virus Therapy.” Trends in Cancer 10: 135–146. 10.1016/j.trecan.2023.09.012 37880008 PMC10922271

[783] Bahreyni, Amirhossein , Yasir Mohamud , Honglin Luo . 2024. “Oncolytic Virus‐Based Combination Therapy in Breast Cancer.” Cancer Letters 585: 216634. 10.1016/j.canlet.2024.216634 38309616

[784] Kaufman, Howard L. , Frederick J. Kohlhapp , Andrew Zloza . 2015. “Oncolytic Viruses: A New Class of Immunotherapy Drugs.” Nature Reviews Drug Discovery 14: 642–662. 10.1038/nrd4663 26323545 PMC7097180

[785] Shalhout, Sophia Z. , David M. Miller , Kevin S. Emerick , Howard L. Kaufman . 2023. “Therapy With Oncolytic Viruses: Progress and Challenges.” Nature Reviews Clinical Oncology 20: 160–177. 10.1038/s41571-022-00719-w 36631681

[786] Hennessy, Morgan L. , Praveen K. Bommareddy , Genevieve Boland , Howard L. Kaufman . 2019. “Oncolytic Immunotherapy.” Surgical Oncology Clinics of North America 28: 419–430. 10.1016/j.soc.2019.02.007 31079797

[787] Ma, Rui , Zhenlong Li , E. Antonio Chiocca , Michael A. Caligiuri , Jianhua Yu . 2023. “The Emerging Field of Oncolytic Virus‐Based Cancer Immunotherapy.” Trends in Cancer 9: 122–139. 10.1016/j.trecan.2022.10.003 36402738 PMC9877109

[788] Han, Zi‐Yi , Qi‐Wen Chen , Zhuang‐Jiong Fu , Si‐Xue Cheng , Xian‐Zheng Zhang . 2022. “Probiotic Spore‐Based Oral Drug Delivery System for Enhancing Pancreatic Cancer Chemotherapy by Gut‐Pancreas‐Axis‐Guided Delivery.” Nano Letters 22: 8608–8617. 10.1021/acs.nanolett.2c03131 36259687

[789] Nguyen, Vu H. , Hyung‐Seok Kim , Jung‐Min Ha , Yeongjin Hong , Hyon E. Choy , Jung‐Joon Min . 2010. “Genetically Engineered *Salmonella typhimurium* as an Imageable Therapeutic Probe for Cancer.” Cancer Research 70: 18–23. 10.1158/0008-5472.CAN-09-3453 20028866

[790] Ballister, Edward R. , Alexander Michels , Rosa L. Vincent , Lior Kreindler , Sreyan Chowdhury , Samik Upadhaya , Ana Rosa Saez‐Ibañez , et al. 2025. “The Emerging Landscape of Engineered Bacteria Cancer Therapies.” Nature Biotechnology 43: 672–676. 10.1038/s41587-025-02623-x 40169920

[791] Schmitz‐Winnenthal, Friedrich H. , Hohmann Nicolas , Andreas G. Niethammer , Tobias Friedrich , Heinz Lubenau , Marco Springer , Klaus M. Breiner , et al. 2015. “Anti‐Angiogenic Activity of VXM01, an Oral T‐Cell Vaccine Against VEGF Receptor 2, in Patients With Advanced Pancreatic Cancer: A Randomized, Placebo‐Controlled, Phase 1 Trial.” Oncoimmunology 4: e1001217. 10.1080/2162402X.2014.1001217 26137397 PMC4485742

[792] Le, Dung T. , Dirk G. Brockstedt , Ran Nir‐Paz , Johannes Hampl , Shruti Mathur , John Nemunaitis , Daniel H. Sterman , et al. 2012. “A Live‐Attenuated Listeria Vaccine (ANZ‐100) and a Live‐Attenuated Listeria Vaccine Expressing Mesothelin (CRS‐207) for Advanced Cancers: Phase I Studies of Safety and Immune Induction.” Clinical Cancer Research: An Official Journal of the American Association for Cancer Research 18: 858–868. 10.1158/1078-0432.CCR-11-2121 22147941 PMC3289408

[793] Wang, Heng , Fang Xu , Chao Wang . 2025. “Metabolic Reprogramming of Tumor Microenviroment by Engineered Bacteria.” Seminars in Cancer Biology 112: 58–70. 10.1016/j.semcancer.2025.03.003 40157514

[794] Zhang, Hao , Li Fu , Xinwen Leiliang , Chunrun Qu , Wantao Wu , Rong Wen , Ning Huang , et al. 2024. “Beyond the Gut: The Intratumoral Microbiome's Influence on Tumorigenesis and Treatment Response.” Cancer Communications 44: 1130–1167. 10.1002/cac2.12597 39087354 PMC11483591

[795] Sieow, Brendan Fu‐Long , Kwok Soon Wun , Wei Peng Yong , In Young Hwang , Matthew Wook Chang . 2021. “Tweak to Treat: Reprograming Bacteria for Cancer Treatment.” Trends in Cancer 7: 447–464. 10.1016/j.trecan.2020.11.004 33303401

[796] Li, Tong , Wenjing Wang . 2021. “New Strategy for Cancer Immunotherapy: Using Live Engineered Bacteria for Metabolic Modulation.” Signal Transduction and Targeted Therapy 6: 415. 10.1038/s41392-021-00829-4 34873144 PMC8648820

[797] Cubillos‐Ruiz, Juan R. , Andres Cubillos‐Ruiz . 2021. “Engineered Bacteria Recycle Tumor Metabolic Waste to Boost Immunotherapy.” Cell Host & Microbe 29: 1725–1727. 10.1016/j.chom.2021.11.008 34883059

[798] Wang, Yifan , Anqi Dong , Jianping Man , Hua Chen , Wenhao Shen , Lei Wang , Hongli Yang , Lin Hu , Kai Yang . 2025. “TREM2 scFv‐Engineering *Escherichia coli* Displaying Modulation of Macrophages to Boost Cancer Radio‐Immunotherapy.” Advanced Materials (Deerfield Beach, Fla.) 37: e2417920. 10.1002/adma.202417920 40103438

[799] Xie, Beibei , Linmiao Dong , Leo Wang , Ruibing Wang , Chunlai Li . 2024. “Supramolecularly Engineered Bacteria Mediated Calcium Overload and Immunotherapy of Tumors.” Theranostics 14: 6560–6570. 10.7150/thno.99931 39479452 PMC11519789

[800] Liu, Xuemeng , Mengyu Sun , Fang Pu , Jinsong Ren , Xiaogang Qu . 2023. “Transforming Intratumor Bacteria Into Immunopotentiators to Reverse Cold Tumors for Enhanced Immuno‐chemodynamic Therapy of Triple‐Negative Breast Cancer.” Journal of the American Chemical Society 145: 26296–26307. 10.1021/jacs.3c09472 37987621

[801] Zhang, Lilong , Dongqi Chai , Chen Chen , Chunlei Li , Zhendong Qiu , Tianrui Kuang , Mungur Parveena , et al. 2022. “Mycobiota and C‐Type Lectin Receptors in Cancers: Know Thy Neighbors.” Frontiers in Microbiology 13: 946995. 10.3389/fmicb.2022.946995 35910636 PMC9326027

[802] De Menezes, Ag‐Anne P. M. , Raí P. S. Aguiar , José V. O. Santos , Chandan Sarkar , Muhammad T. Islam , Antonio L. Braga , Mohammad M. Hasan , et al. 2023. “Citrinin as a Potential Anti‐Cancer Therapy: A Comprehensive Review.” Chemico‐Biological Interactions 381: 110561. 10.1016/j.cbi.2023.110561 37230156

[803] Jin, Jianshi , Reiko Yamamoto , Katsuyuki Shiroguchi . 2024. “High‐Throughput Identification and Quantification of Bacterial Cells in the Microbiota Based on 16S rRNA Sequencing With Single‐Base Accuracy Using BarBIQ.” Nature Protocols 19: 207–239. 10.1038/s41596-023-00906-8 38012397

[804] Galeano Niño, Jorge Luis , Hanrui Wu , Kaitlyn D. LaCourse , Andrew G. Kempchinsky , Alexander Baryiames , Brittany Barber , Neal Futran , et al. 2022. “Effect of the Intratumoral Microbiota on Spatial and Cellular Heterogeneity in Cancer.” Nature 611: 810–817. 10.1038/s41586-022-05435-0 36385528 PMC9684076

[805] Gerber, Georg K. 2024. “AI in Microbiome Research: Where Have We Been, Where Are We Going?” Cell Host & Microbe 32: 1230–1234. 10.1016/j.chom.2024.07.021 39146795

[806] Ghannam, Ryan B. , Stephen M. Techtmann . 2021. “Machine Learning Applications in Microbial Ecology, Human Microbiome Studies, and Environmental Monitoring.” Computational and Structural Biotechnology Journal 19: 1092–1107. 10.1016/j.csbj.2021.01.028 33680353 PMC7892807

[807] Pepke, Michael L. , Søren B. Hansen , Morten T. Limborg . 2024. “Unraveling Host Regulation of Gut Microbiota Through the Epigenome‐Microbiome Axis.” Trends in Microbiology 32: 1229–1240. 10.1016/j.tim.2024.05.006 38839511

[808] Ma, Yong‐Jing , Yuan‐Chen Sun , Lu Wang , Wan‐Xing Xu , Xiao‐Dan Fan , Jun Ding , Christopher Heeschen , Wen‐Juan Wu , Xiao‐Qi Zheng , Ning‐Ning Liu . 2024. Dissection of Intratumor Microbiome–Host Interactions at Single‐Cell Level in Lung Cancer.” hLife: in press. 10.1016/j.hlife.2024.09.001

[809] Ratiner, Karina , Dragos Ciocan , Suhaib K. Abdeen , Eran Elinav . 2024. “Utilization of the Microbiome in Personalized Medicine.” Nature Reviews Microbiology 22: 291–308. 10.1038/s41579-023-00998-9 38110694

[810] Redenti, Andrew , Jongwon Im , Benjamin Redenti , Fangda Li , Mathieu Rouanne , Zeren Sheng , William Sun , et al. 2024. “Probiotic Neoantigen Delivery Vectors for Precision Cancer Immunotherapy.” Nature 635: 453–461. 10.1038/s41586-024-08033-4 39415001 PMC11560847

[811] Li, Zhaoting , Yixin Wang , Jun Liu , Piper Rawding , Jiyoon Bu , Seungpyo Hong , Quanyin Hu . 2021. “Chemically and Biologically Engineered Bacteria‐Based Delivery Systems for Emerging Diagnosis and Advanced Therapy.” Advanced Materials (Deerfield Beach, Fla.) 33: e2102580. 10.1002/adma.202102580 34347325

[812] Shen, Haosheng , Nikhil Aggarwal , Kwok Soon Wun , Yung Seng Lee , In Young Hwang , Matthew Wook Chang . 2022. “Engineered Microbial Systems for Advanced Drug Delivery.” Advanced Drug Delivery Reviews 187: 114364. 10.1016/j.addr.2022.114364 35654214

[813] Krishnamurthy, Malathy , Richard T. Moore , Sathish Rajamani , Rekha G. Panchal . 2016. “Bacterial Genome Engineering and Synthetic Biology: Combating Pathogens.” BMC Microbiology 16: 258. 10.1186/s12866-016-0876-3 27814687 PMC5097395

[814] Cianci, Rossella , Mario Caldarelli , Paola Brani , Annalisa Bosi , Alessandra Ponti , Cristina Giaroni , Andreina Baj . 2025. “Cytokines Meet Phages: A Revolutionary Pathway to Modulating Immunity and Microbial Balance.” Biomedicines 13: 1202. 10.3390/biomedicines13051202 40427029 PMC12109214

[815] Lei, Lei , Yan, Jiayao , Kai Xin , Lin Li , Qi Sun , Ying Wang , Tianran Chen , et al. 2024. “Engineered Bacteriophage‐Based In?Situ Vaccine Remodels a Tumor Microenvironment and Elicits Potent Antitumor Immunity.” ACS Nano 18: 12194–12209. 10.1021/acsnano.4c00413 38689426

[816] Hou, Xiao‐Lin , Bin Zhang , Kai Cheng , Fang Zhang , Xiao‐Ting Xie , Wei Chen , Lin‐Fang Tan , et al. 2024. “Engineering Phage Nanocarriers Integrated With Bio‐Intelligent Plasmids for Personalized and Tunable Enzyme Delivery to Enhance Chemodynamic Therapy.” Advanced Science (Weinheim, Baden‐Wurttemberg, Germany) 11: e2308349. 10.1002/advs.202308349 38582522 PMC11199971

[817] Al‐Bahrani, Mariam , Paladd Asavarut , Sajee Waramit , Keittisak Suwan , Amin Hajitou . 2023. “Transmorphic Phage‐Guided Systemic Delivery of TNFα Gene for the Treatment of Human Pediatric Medulloblastoma.” The FASEB Journal 37: e23038. 10.1096/fj.202300045R 37331004 PMC10947044

[818] Li, Dengxiong , Ruicheng Wu , Qingxin Yu , Zhouting Tuo , Jie Wang , Koo Han Yoo , Wuran Wei , et al. 2024. “Microbiota and Urinary Tumor Immunity: Mechanisms, Therapeutic Implications, and Future Perspectives.” Chinese Journal of Cancer Research = Chung‐Kuo Yen Cheng Yen Chiu 36: 596–615. 10.21147/j.issn.1000-9604.2024.06.03 39802902 PMC11724181

[819] Zhao, Lihong , Mei Li , Chen Shen , Yurui Luo , Xiaoming Hou , Yu Qi , Ziwei Huang , et al. 2024. “Nano‐Assisted Radiotherapy Strategies: New Opportunities for Treatment of Non‐Small Cell Lung Cancer.” Research (Washington, D.C.) 7: 0429. 10.34133/research.0429 39045421 PMC11265788

[820] Fang, Yiran , Yuyun Kong , Guangda Rong , Qingcong Luo , Wangjun Liao , Dongqiang Zeng . “Systematic Investigation of Tumor Microenvironment and Antitumor Immunity With IOBR.” Med Research 1: 136–140. 10.1002/mdr2.70001

[821] Yu, Li , Ke Huang , Yixiang Liao , Lingzhi Wang , Gautam Sethi , Zhaowu Ma . 2024. “Targeting Novel Regulated Cell Death: Ferroptosis, Pyroptosis and Necroptosis in Anti‐PD‐1/PD‐L1 Cancer Immunotherapy.” Cell Proliferation 57: e13644. 10.1111/cpr.13644 38594879 PMC11294428

[822] Jiang, Aimin , Jinxin Li , Ziwei He , Ying Liu , Kun Qiao , Yu Fang , Le Qu et al. 2024. “Renal Cancer: Signaling Pathways and Advances in Targeted Therapies.” MedComm 5: e676. 10.1002/mco2.676 39092291 PMC11292401

[823] Zetrini, Abdulmottaleb E. , HoYin Lip , Azhar Z. Abbasi , Ibrahim Alradwan , Taksim Ahmed , Chunsheng He , Jeffrey T. Henderson , Andrew M. Rauth , Xiao Yu Wu . 2023. “Remodeling Tumor Immune Microenvironment by Using Polymer‐Lipid‐Manganese Dioxide Nanoparticles With Radiation Therapy to Boost Immune Response of Castration‐Resistant Prostate Cancer.” Research (Washington, D.C.) 6: 0247. 10.34133/research.0247 37795337 PMC10546607

[824] Li, Zizhuo , Anqi Lin , Zhifei Gao , Aimin Jiang , Minying Xiong , Jiapeng Song , Zaoqu Liu , et al. 2024. “B‐Cell Performance in Chemotherapy: Unravelling the Mystery of B‐Cell Therapeutic Potential.” Clinical and Translational Medicine 14: e1761. 10.1002/ctm2.1761 38997802 PMC11245406

[825] Lin, Anqi , Chang Qi , Ting Wei , Mengyao Li , Quan Cheng , Zaoqu Liu , Peng Luo , Jian Zhang . 2022. “CAMOIP: A Web Server for Comprehensive Analysis on Multi‐Omics of Immunotherapy in Pan‐Cancer.” Briefings in Bioinformatics 23: bbac129. 10.1093/bib/bbac129 35395670

[826] Zhang, Haonan , Ge Zhang , Pengyuan Xu , Fengyi Yu , Liwen Li , Runzhi Huang , Pengpeng Zhang , et al. “Optimized Dynamic Network Biomarker Deciphers a High‐Resolution Heterogeneity Within Thyroid Cancer Molecular Subtypes.” Med Research 1: 10‐31. 10.1002/mdr2.70004

[827] Zhu, Xiaoqiang , Muni Hu , Xiaowen Huang , Lingxi Li , Xiaolin Lin , Xiaoyan Shao , Jiantao Li , et al. 2025, 37, 806–823.e6. “Interplay Between Gut Microbial Communities and Metabolites Modulates Pan‐Cancer Immunotherapy Responses.” Cell Metabolism. 10.1016/j.cmet.2024.12.013 39909032

[828] Battaglia, Thomas W. , Iris L. Mimpen , Joleen J. H. Traets , Arne Van Hoeck , Laurien J. Zeverijn , Birgit S. Geurts , Gijs F. De Wit , et al. 2024. “A Pan‐Cancer Analysis of the Microbiome in Metastatic Cancer.” Cell 187: 2324–35.e19. 10.1016/j.cell.2024.03.021 38599211

[829] Liu, Yali , Chi Chun Wong , Yanqiang Ding , Mengxue Gao , Jun Wen , Harry Cheuk‐Hay Lau , Alvin Ho‐Kwan Cheung , Dan Huang , He Huang et al. 2024. “ *Peptostreptococcus anaerobius* Mediates Anti‐PD1 Therapy Resistance and Exacerbates Colorectal Cancer via Myeloid‐Derived Suppressor Cells in Mice.” Nature Microbiology 9: 1467–1482. 10.1038/s41564-024-01695-w PMC11153135 38750176

[830] Tian, Jinzhong , Chong Li , Zhixiang Dong , Yunpeng Yang , Jing Xing , Peijun Yu , Ying Xin , et al. 2023. “Inactivation of the Antidiabetic Drug Acarbose by Human Intestinal Microbial‐Mediated Degradation.” Nature Metabolism 5: 896–909. 10.1038/s42255-023-00796-w 37157031

[831] Schupack, Daniel A. , Ruben A. T. Mars , Dayne H. Voelker , Jithma P. Abeykoon , Purna C. Kashyap . 2022. “The Promise of the Gut Microbiome as Part of Individualized Treatment Strategies.” Nature Reviews Gastroenterology & Hepatology 19: 7–25. 10.1038/s41575-021-00499-1 34453142 PMC8712374

[832] Liu, Yafeng , Shujun Zhang , Kaijie Liu , Xinjun Hu , Xinyu Gu . 2024. “Advances in Drug Discovery Based on Network Pharmacology and Omics Technology. “Current Pharmaceutical Analysis 21: 33–43. 10.1016/j.cpan.2024.12.002

[833] Chen, Jinghong , Anqi Lin , Peng Luo . 2024. “Advancing Pharmaceutical Research.” Current Pharmaceutical Analysis 21: 1–19. 10.1016/j.cpan.2024.11.001

[834] Wang, Ziheng , Yang Zhao , Lin Zhang . 2024. “Emerging Trends and Hot Topics in the Application of Multi‐Omics in Drug Discovery.” Current Pharmaceutical Analysis 21: 20–32. 10.1016/j.cpan.2024.12.001

